# Efficacy of automated insulin delivery systems in people with type 1 diabetes: a systematic review and network meta-analysis of outpatient randomised controlled trials

**DOI:** 10.1016/j.eclinm.2025.103190

**Published:** 2025-04-11

**Authors:** Anna Stahl-Pehe, Nafiseh Shokri-Mashhadi, Marielle Wirth, Sabrina Schlesinger, Oliver Kuss, Reinhard W. Holl, Christina Bächle, Klaus-D. Warz, Jutta Bürger-Büsing, Olaf Spörkel, Joachim Rosenbauer

**Affiliations:** a Institute for Biometrics and Epidemiology, German Diabetes Center (DDZ), Leibniz Center for Diabetes Research at Heinrich Heine University Düsseldorf, Düsseldorf, Germany; b German Center for Diabetes Research (DZD), Partner Düsseldorf, Munich-Neuherberg, Germany; c Department of General Pediatrics, Neonatology and Pediatric Cardiology, Medical Faculty and University Hospital Düsseldorf, Heinrich Heine University Düsseldorf, Germany; d Centre for Health and Society, Medical Faculty and University Hospital Düsseldorf, Heinrich Heine University Düsseldorf, Germany; e Institute of Epidemiology and Medical Biometry, ZIBMT, University of Ulm, Germany; f Deutsche Diabetes Föderation e.V. (DDF), Berlin, Germany; g Bund Diabetischer Kinder und Jugendlicher e.V. (BdKJ), Kaiserslautern, Germany; h National Diabetes Information Center, German Diabetes Center (DDZ), Leibniz Center for Diabetes Research at Heinrich Heine University Düsseldorf, Düsseldorf, Germany

**Keywords:** Artificial pancreas system, Automated insulin delivery, Network meta-analysis, Systematic review, Type 1 diabetes

## Abstract

**Background:**

The comparative efficacy of automated insulin delivery (AID) systems and other treatment options for type 1 diabetes, accounting for the certainty of evidence (CoE), is unknown.

**Methods:**

We searched PubMed, EMBASE, the Cochrane Central Register of Controlled Trials and ClinicalTrials.gov and included outpatient randomised controlled trials (RCTs) published until January 8, 2025, in people with type 1 diabetes with a three-week or longer intervention of AID systems (PROSPERO registration number: CRD42023395492). We performed pairwise and network meta-analyses and used the Risk of Bias tool 2 and the Grading of Recommendations Assessment, Development and Evaluation methods to determine the CoE for each outcome.

**Findings:**

A total of 46 studies involving seven insulin treatment options and 4113 participants were included, of which 29 and 17 had low and moderate risks of bias, respectively. The intervention AID systems, including the hybrid closed-loop (HCL), advanced HCL (AHCL) and full closed-loop (FCL) systems, were evaluated in 20, 25 and 1 studies, respectively. The network meta-analysis did not indicate global inconsistencies but did indicate global publication bias for all glycaemic outcomes. The CoE varied between very low and high, depending on the treatment and outcome under consideration. Compared with pump therapy, the percentage of time in the range 70–180 mg/dl was greater with AID use (HCL: 19.7% [95% confidence interval 13.2%; 26.1%], moderate CoE; AHCL: 24.1% [18.2%; 29.9%], moderate CoE; FCL: 25.5% [11.1%; 39.9%], high CoE). Compared with pump therapy, the percentage of time above 180 mg/dl and 250 mg/dl was lower with AHCL, on average, by 19.6% (14.0%; 25.1%), moderate CoE, and 14.8% (8.8%; 20.8%), moderate CoE, respectively. The CoE was very uncertain regarding the overall effect of AID systems on the percentage of time below 70 mg/dl and 54 mg/dl and the HbA1c.

**Interpretation:**

AID systems improve glycaemic outcomes to varying degrees and with varying CoE.

**Funding:**

10.13039/501100002347German Federal Ministry of Education and Research (BMBF; grant 01KG2203).

**Research in context**

**Evidence before this study**

Automated insulin delivery (AID) systems represent constantly evolving options for the treatment of type 1 diabetes. According to a nonsystematic Pubmed search with variations of the search term ‘type 1 diabetes AND AID’ until March 31, 2023, most randomised controlled trials (RCTs) and pairwise meta-analyses compared a single AID system with a non-AID system and lacked direct comparisons between different AID systems. With respect to the efficacy of AID systems, the certainty of the evidence (CoE) was largely unknown. Network meta-analysis (NMA) with CoE assessment provides an effective strategy to address these issues. Limitations of previous NMAs were the short follow-up duration of the RCTs, small sample sizes, narrowly defined patient groups and the absence of a CoE assessment or very low CoE.

**Added value of this study**

To our knowledge, this is the first NMA with a CoE assessment that includes hybrid, advanced hybrid and full closed-loop systems, and focuses on longer-term interventions. A total of 29 and 17 RCTs had low and moderate risks of bias, respectively. The results showed that AID systems were beneficial to varying degrees with respect to the percentage of time in the range of 70–180 mg/dl and the percentage of time above the ranges of 180 mg/dl and 250 mg/dl. The CoE varied between very low and high, depending on the treatments and outcomes under consideration.

**Implications of all the available evidence**

This study includes a comprehensive comparison of the relative efficacy of various treatment options for type 1 diabetes through a systematic review and NMA. The findings update and add to the existing evidence and provide valuable information for the decision-making process between patients and clinicians on the actual achievable benefits of available treatment options without neglecting heterogeneity and uncertainties in this context.

## Introduction

Type 1 diabetes is a chronic autoimmune disease characterised by insulin deficiency that requires intensive insulin therapy to maintain optimal blood glucose levels. Most of the currently available treatment options impose a significant burden on daily life but do not result in optimal outcomes for many people with type 1 diabetes.^1^ The latest technologies are automated insulin delivery (AID) systems, also known as closed-loop or artificial pancreas systems, which combine continuous glucose monitoring with automated subcutaneous insulin delivery in a glucose-dependent manner. A variety of AID systems have been tested in randomised controlled trials (RCTs) and have been shown to be safe and beneficial for maintaining glucose levels within the target glycaemic range.^2^^,^^3^ Owing to the heterogeneity of AID systems with different degrees of automation and the control treatments tested in RCTs, network meta-analyses (NMAs) combining direct and indirect evidence are necessary to consider all evidence from RCTs and to enable a ranking of available systems based on efficacy. Previous NMAs had the following limitations: they involved systematic searches conducted before 2024 and thus before several longer-term RCTs were published; they had small sample sizes and narrow inclusion criteria; they evaluated few outcomes; and they were conducted without any patient or public involvement.^4^, ^5^, ^6^, ^7^ Therefore, this systematic review and NMA aimed to evaluate the comparative efficacy of AID systems and other currently available insulin treatment modalities based on outpatient RCTs of at least three weeks of intervention involving people with type 1 diabetes.

## Methods

This systematic review with NMA is reported according to the Preferred Reporting Items for Systematic Reviews and Meta-Analyses (PRISMA) guidelines specific for NMAs.^8^^,^^9^ The study protocol was registered with PROSPERO (CRD42023395492) and published in advance.^10^ Ethics approval was not obtained as this systematic review was secondary research using aggregated, anonymised data available in the public domain. Because limited data were available from identified RCTs, slight deviations from the protocol were necessary, which are reported in Appendix 1.3.

### Identification and selection of studies

For the original search, Medline via PubMed, Embase, the Cochrane Central Register of Controlled Trials (CENTRAL) and ClinicalTrials.gov were searched from database inception to April 17, 2023. The same databases were used to update the search on January 8, 2025. A predefined search strategy (Appendix 1.1) was used, without language, publication date or publication status restrictions. All the identified references were imported into EndNote (Clarivate, PA, USA) to remove duplicates and subsequently exported to Covidence systematic review software (Veritas Health Innovation, Melbourne, Australia). Two investigators (AS-P and NS-M) independently screened the titles and abstracts, followed by screening the full texts of the eligible publications. In accordance with the protocol,^10^ we used the following criteria to select studies for inclusion: (a) population: people with type 1 diabetes in their home and work environment, including people of all ages, pregnant women and individuals with comorbidities; (b) intervention and control: AID systems with hybrid (HCL), advanced hybrid (AHCL) or full closed-loop (FCL) technology including do-it-yourself systems used by participants day and night compared with any other type of insulin-based therapy, with a minimum intervention duration of three weeks; (c) outcomes: glycaemic outcomes and patient-reported outcomes; and (d) study design: outpatient RCTs. In addition, conference abstracts published since 2018 were searched, and forwards and backwards reference searches of the included studies and all relevant systematic reviews were performed via the Citationchaser^11^ application to identify additional full-text reports. The selection process is summarised in a flow chart.^12^

### Outcomes

As the primary outcome, we considered the proportion (%) of time that the blood glucose concentrations of people with type 1 diabetes were within the target range (time in range, TIR) of 70–180 mg/dl (3.9–10.0 mmol/l). The secondary outcomes included several levels of hypoglycaemia and hyperglycaemia, and haemoglobin A1c (HbA1c) levels.

### Data extraction

Two investigators (AS-P and NS-M) independently extracted relevant data on study characteristics and postintervention mean outcome values with corresponding standard deviations (SDs) or, alternatively, medians and interquartile ranges, at the study level using a piloted Microsoft Excel table. Treatments were grouped into seven categories: multiple daily injections (MDI), continuous subcutaneous insulin infusion (CSII), sensor augmented pump (SAP), predictive low glucose management (PLGM), hybrid closed-loop (HCL), advanced hybrid closed-loop (AHCL), and full closed-loop (FCL) as defined in Appendix 1.2. For treatment groups with more than one treatment, participants were assigned to the category with a percentage share above 50% and, in all other cases, to the category with the highest degree of automation. For both screening and data extraction, disagreements between the two investigators were resolved by discussion or, if necessary, through consultation with a third investigator (SS). The outcomes for which the data were extracted are shown in Appendix 1.3. Additional data were requested via email and provided by the authors of two studies.^13^^,^^14^

### Risk of bias

The risk of bias (RoB) of the included studies was assessed at the outcome level to evaluate intention-to-treat effects by means of the Cochrane Risk of Bias tool for RCTs (RoB 2). The results of the RoB evaluation for the primary outcome were applied to all glycaemic outcomes as they were all measured objectively by continuous glucose monitoring systems or laboratory analysis. Two investigators (AS-P and NS-M) independently used the RoB 2 Microsoft Excel tools for parallel and crossover study designs to judge the ‘randomisation process’, ‘deviations from intended interventions’, ‘missing outcome data’, ‘measurement of the outcome’ and ‘selection of the reported result’ bias domains, as well as the ‘bias arising from period and carryover effects’ bias domain for crossover studies. In addition, overall RoB was rated as “low”, “some concerns” or “high”.^15^ A third investigator (SS) was consulted to resolve discrepancies.

### Statistical analysis

All of the outcomes evaluated, including the TIR, time above ranges (TARs) (glucose concentrations >180 mg/dl and >250 mg/dl), time below ranges (TBRs) (glucose concentrations <70 mg/dl and <54 mg/dl), and HbA1c level, were continuous. We used the mean difference as the treatment effect accompanied by the corresponding standard error (SE) if reported or derived from the reported 95% confidence interval (CI) given. For the studies reporting only quartiles, we calculated the means and SEs via the Box–Cox method within the “estmeansd” package in R.

First, we performed pairwise meta-analyses for all AID systems versus control treatments if two or more studies were available within a frequentist framework via a random effects model (DerSimonian and Laird approach). Forest plots including heterogeneity estimates (I^2^ and τ^2^) were created to illustrate study-specific and overall effects. We assessed potential small study effects when ten or more trials were included in the meta-analysis by visual inspection of the funnel plots with respect to the criterion of symmetry, Egger's test (p value <0.1 indicates asymmetry) and the trim-and-fill method. Furthermore, we conducted the following sensitivity analyses to assess the robustness of the results: (a) subgroup analyses according to the characteristics of the study design and population (RoB [categorised as low risk, some concerns, high risk], study design [parallel, crossover], sample size [<100 participants, ≥100 participants], intervention duration [3 to <12 weeks, ≥12 weeks], age [<18 years, ≥18 years, age mixed population], study population [only women, men and women], diabetes duration [new-onset type 1 diabetes, longer-term type 1 diabetes], baseline HbA1c [≤7.5%, >7.5%]/[≤58 mmol/mol, >58 mmol/mol]); (b) leave-one-out meta-analysis to investigate the influence of each study on the overall effect estimate.

Second, we conducted a NMA with random effects within a frequentist framework to compare all interventions simultaneously, creating a connected network using all available evidence. The network geometry^16^ and additional geometry metrics provide an overview of the network relationships.^17^ For each outcome, direct, indirect and network estimates were computed and are provided in a side-split and league table, respectively. To identify the percentage contribution of each direct comparison to each network estimate and for the entire network, a contribution matrix^16^ was estimated. An additional diagram shows the RoB for each network estimate in pairwise comparisons based on the contributions of the direct evidence.^18^ We further present the network estimates of mean differences along with their 95% CIs and 95% predictive intervals (95% PrIs) in predictive interval plots to indicate the interval within which the relative treatment effect of a future study is expected to lie.^16^^,^^19^

To assess transitivity in the NMA, we visually inspected the similarity of the distributions of prespecified effect modifiers (intervention duration, sample size, RoB, age, sex, diabetes duration, and HbA1c) across treatment comparisons.^20^ The “design-by-treatment” interaction model and the side-splitting approach were applied to check for inconsistency in the entire network (globally) and in each individual loop (locally), respectively. We present the results for each outcome in a side-split table, a net heat plot and an inconsistency plot.^16^^,^^21^ In addition, a comparison-adjusted funnel plot was used to visually inspect the criterion of symmetry.^16^ We ranked all interventions per network under the consistency assumption via P-scores but also provided the mean rank, surface under the cumulative ranking curve (SUCRA), and SUCRA plots.^22^

Statistical evaluations and graph creation were performed via the network and network graph packages in Stata/SE, version 17.0 (StataCorp LLC).^16^ The net heat plot was produced with RStudio, version 4.3.2.^21^

### Certainty of evidence

We followed the Grading of Recommendations Assessment, Development, and Evaluation (GRADE) approach to rate and communicate the CoE of the results derived from the pairwise meta-analyses and NMAs.^23^^,^^24^ The GRADE framework distinguishes four levels of CoE: high, moderate, low, and very low. The detailed GRADE assessments, including the reasons for rating down are shown in Appendix 1.4. We used a minimally contextualised framework with a prespecified minimal important difference (MID) derived from the literature evidence (Appendix 1.4) as our decision threshold.^25^^,^^26^ For all outcomes, the CoE was rated independently by two investigators (AS-P and NS-M), who resolved discrepancies by consensus supported by a third investigator (SS).

An online tool, GRADEPro GDT (https://gdt.gradepro.org/app/), was used to grade the CoEs of pairwise meta-analyses. For NMAs, a spreadsheet that incorporates the automation of several of the steps for applying the GRADE framework was used.^27^

### Patient and public involvement

Two patient representatives were part of the research team at all stages of the study. They were involved in the identification of the patient-relevant research questions and the selection of the primary and secondary outcomes. They have participated throughout the duration of the study and will disseminate the results to lay people and relevant target groups.

### Role of the funding source

This work was supported by the German Federal Ministry of Education and Research (BMBF; grant 01KG2203). The funder had no role in study design, data collection, data analysis, interpretation, or writing of the report.

## Results

### Literature search and study characteristics

**Fig. 1:**
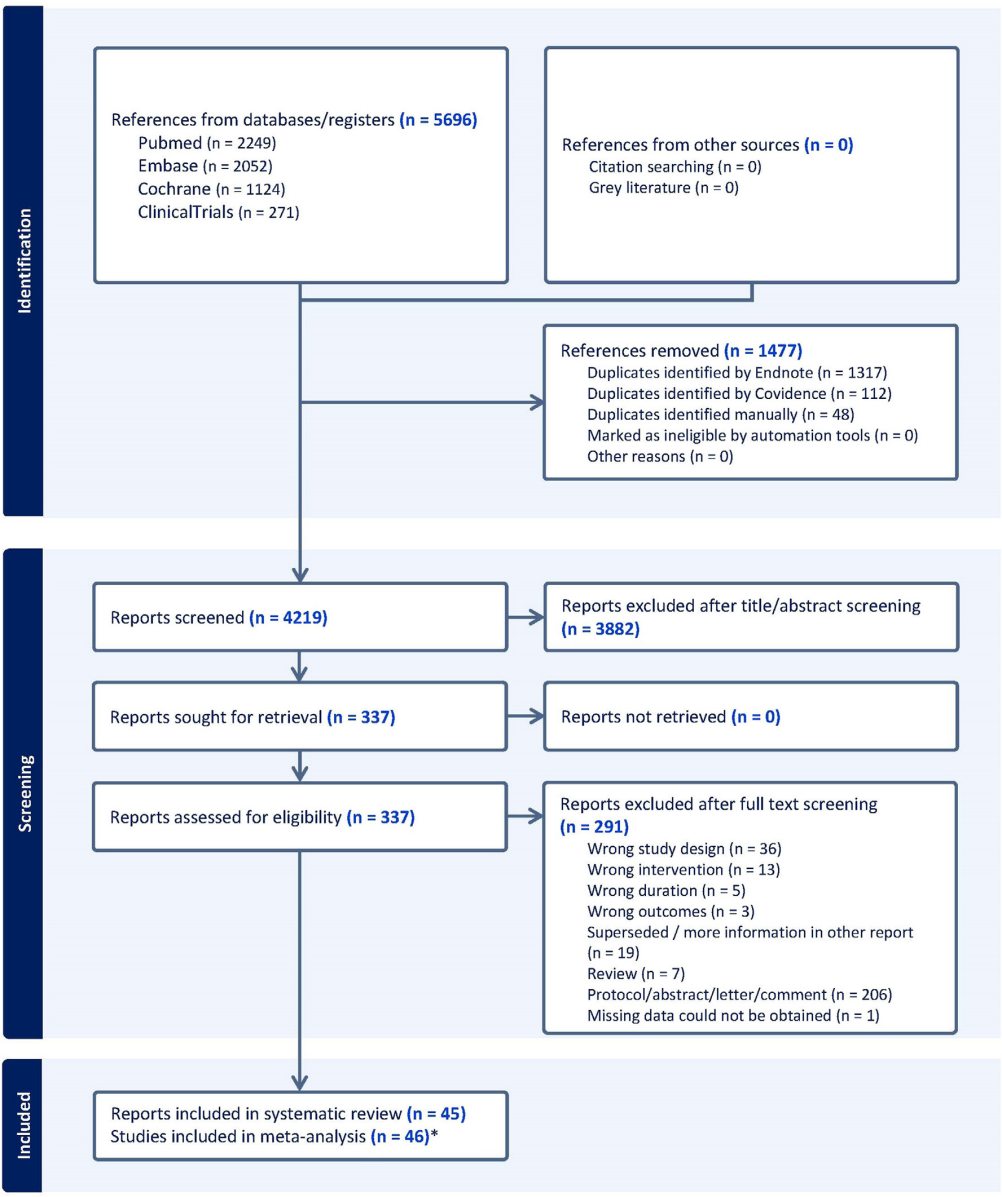
**Flow diagram showing identification, selection, and inclusion of studies**. ∗ One report provided data separately for age-groups and was treated as two studies.^28^

The flow diagram for trial screening and selection is provided in Fig. 1, and the reasons for exclusion after full-text screening are provided in Appendix 2.1. Of the 5696 identified articles, 337 were full-text screened, and 45 reports^13^^,^^14^^,^^28^, ^29^, ^30^, ^31^, ^32^, ^33^, ^34^, ^35^, ^36^, ^37^, ^38^, ^39^, ^40^, ^41^, ^42^, ^43^, ^44^, ^45^, ^46^, ^47^, ^48^, ^49^, ^50^, ^51^, ^52^, ^53^, ^54^, ^55^, ^56^, ^57^, ^58^, ^59^, ^60^, ^61^, ^62^, ^63^, ^64^, ^65^, ^66^, ^67^, ^68^, ^69^, ^70^, ^71^ corresponding to 46 studies (18 crossover studies and 28 parallel design studies) were eligible for inclusion. In 35 studies, the intervention lasted 12 weeks or longer. A total of 4113 participants were included, with most studies (n = 31) comprising fewer than 100 participants. The intervention AID systems, including the HCL, AHCL and FCL systems, were evaluated in 20, 25 and 1 studies respectively. The control treatments were MDI, CSII, SAP, PLGM and HCL in 6, 5, 24, 9 and 2 studies, respectively. Most studies were supported to some degree by industry (Appendix 2.2).

### Risk of bias of the included studies

Most of the studies (n = 29) had a low RoB, some (n = 17) had some concerns regarding RoB, and none had a high RoB. The primary reason for the RoB of the included studies was a lack of clarity in reporting the allocation sequence of the study participants (Appendix 3).

### Pairwise meta-analysis

The results of the pairwise meta-analyses are provided in detail in Appendices 4–7, and the results of the CoE assessment are provided in Appendix 8. All pairwise comparisons revealed substantial between-study heterogeneity, as measured by τ^2^ and I^2^ except for TIR when HCL and AHCL were compared with CSII and TBRs when AHCL and HCL were compared (Appendices 4.2–4.7). There was funnel plot asymmetry indicating small-study bias for the outcomes of TIR and TAR >180 mg/dl when HCL or AHCL was compared with SAP, for TAR >250 mg/dl when AHCL and SAP were compared, and for TBR <70 mg/dl when HCL and SAP were compared (Appendix 7).

### Network meta-analysis

**Fig. 2:**
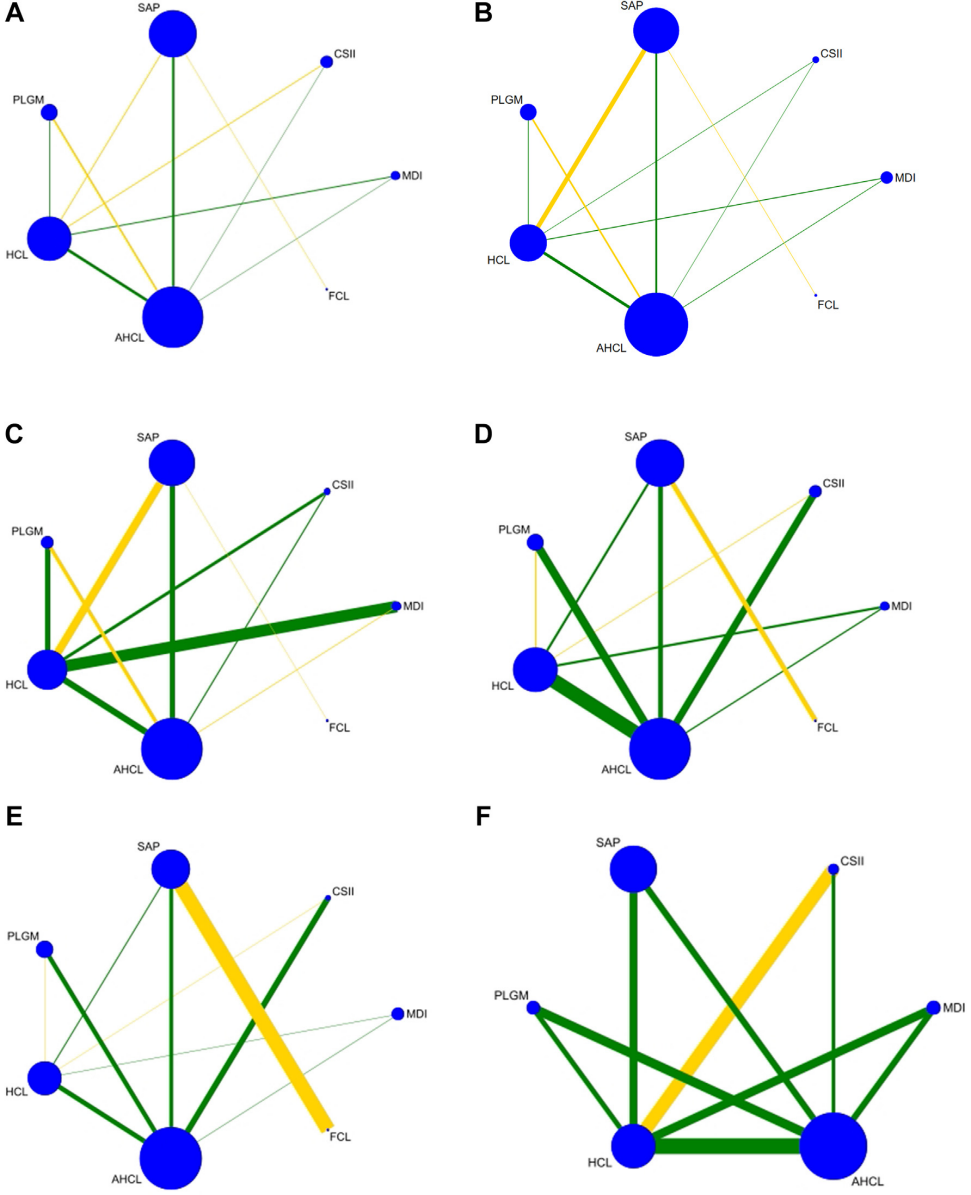
**Network plots for (A) time in range 70–180 mg/dl, (B) time above range >180 mg/dl, (C) time above range >250 mg/dl, (D) time below range <70 mg/dl, (E) time below range <54 mg/dl, and (F) Haemoglobin A1c**. Explanations: The node size in the network graph represents the number of participants with the respective intervention. The line width of the edge represents the mean of inverse variances (precision) of the treatment effect of the studies on which the direct comparison is based. The color of the edge corresponds to the average level of the RoB estimated as the precision-weighted mean of the trials of the direct comparison.^16^

Fig. 2 shows the network plots with connections between treatments separately for the glycaemic outcomes analysed. The six networks comprised 25 to 45 trials, each consisting of six to seven nodes (treatment categories) and nine to ten edges (direct comparisons), 56%–70% of which were based on strong edges with more than one study. Additional network metrics are given in Figure B of Appendices 9.1–9.6. Overall, the network structures reflected a low to moderate RoB. The figures with the study limitations for each network estimate illustrate that there was low RoB for the comparison of HCL or AHCL systems with non-AID therapies for most outcomes. FCL was only directly compared with SAP, and this comparison was based on a single study that had a moderate RoB (Appendices 9.1–9.6, Figure B).

For all outcomes, there was insufficient evidence to assess transitivity due to the small number of studies (Appendices 9.1–9.6, Figure A). The results regarding global network inconsistencies did not suggest any inconsistencies (Appendix 9.7). Local inconsistencies were identified in the loop CSII—HCL—AHCL for TBRs <70 mg/dl and < 54 mg/dl (Appendices 9.1–9.6, Figures E and F). The visual inspection of the comparison-adjusted funnel plots suggested global publication bias for all outcomes (Appendices 9.1–9.6, Figure H).

#### Time in range

All the network estimates consistently show that the use of an AID system was beneficial for increasing the TIR compared with other currently available treatments. However, the intervention with AID was not classified as effective in all cases due to wide confidence intervals that included the MID of a 5% TIR increase. The CoE for the results varied and ranged from very low to high (Table 1, Appendix 10.2). The greatest benefits were observed for HCL compared with CSII therapy (19.7% [13.2%; 26.1%] higher TIR; moderate CoE), AHCL compared with CSII (24.1% [18.2%; 29.9%] higher TIR; moderate CoE), and FCL compared with CSII therapy (25.5% [11.1%; 39.9%] higher TIR; high CoE) (Table 1, Table A in Appendix 9.1). The comparison of the 95% CIs and 95% PrIs revealed that a future study is not expected to substantially change these results (Appendix 9.1, Figure G). According to the P-scores, FCL therapy was the most effective therapy for increasing the TIR, followed by AHCL therapy (Table 2, Figure I and Table C in Appendix 9.1).

**Table 1:** Comparative efficacy of AID systems and other treatment options for type 1 diabetes.

AHCL: Advanced Hybrid Closed-Loop; AID: Automated Insulin Delivery; CSII: Continuous Subcutaneous Insulin Infusion; FCL: Full Closed-Loop; HbA1c: Haemoglobin A1c; HCL: Hybrid Closed-Loop; MDI: Multiple Daily Injection; MID: Minimal Important Difference; NMA: Network Meta-Analysis; PLGM: Predictive Low Glucose Management; SAP: Sensor-Augmented Pump.

Explanation:

**Table 2:** Treatment rankings of insulin treatments for glycaemic outcomes from network meta-analysis.

Treatment	P-score					
	Time in range 70–180 mg/dl	Time above range >180 mg/dl	Time above range >250 mg/dl	Time below range <70 mg/dl	Time below range <54 mg/dl	HbA1c
MDI	0.0	0.0	0.4	0.9	1.1	0.1
CSII	0.0	76.8	86.5	4.0	18.4	88.0
SAP	0.0	22.5	8.6	57.9	51.5	11.9
PLGM	0.0	0.6	3.5	4.3	9.0	0.0
HCL	0.4	0.0	0.0	0.0	0.0	0.0
AHCL	41.3	0.0	0.0	0.0	0.0	0.0
FCL	58.3	0.1	1.0	32.9	20.0	–

P-score: The probability for each intervention to be better than all competing interventions.

HbA1c: Haemoglobin A1c; MDI: Multiple Daily Injection; CSII: Continuous Subcutaneous Insulin Infusion; SAP: Sensor-Augmented Pump therapy; PLGM: Predictive Low Glucose Management; HCL: Hybrid Closed-Loop; AHCL: Advanced Hybrid Closed-Loop; FCL: Full Closed-Loop.

#### Time above range

Regarding TARs >180 mg/dl and >250 mg/dl, the network estimates indicated a benefit of AID systems over non-AID systems, which was not always clinically relevant, with very low to high CoE (Table 1, Appendices 10.3 and 10.4). Compared with CSII, HCL therapy reduced the TAR >180 mg/dl on average by 14.2% (7.8%; 20.6%; moderate CoE), and AHCL and FCL reduced it by 19.6% (14.0%; 25.1%; moderate CoE) and 23.3% (8.4%; 38.2%; high CoE), respectively (Table 1, Table A in Appendix 9.2). Compared with CSII, HCL, AHCL and FCL therapy reduced the TAR >250 mg/dl on average by 10.1% (3.6%; 16.5%; moderate CoE), 14.8% (8.8%; 20.8%; moderate CoE) and 17.7% (2.8%; 32.7%; high CoE), respectively (Table 1, Table A in Appendix 9.3). Based on the 95% PrIs, only AHCL therapy compared with CSII met the MIDs for TAR >180 mg/dl (Appendix 9.2, Figure G) and TAR >250 mg/dl (Appendix 9.3, Figure G). According to the P-scores, the most effective treatments for reducing TARs >180 mg/dl and >250 mg/dl were CSII and SAP therapy (Table 2, Figure I and Table C in Appendices 9.2 and 9.3).

#### Time below range

With respect to TBR <70 mg/dl, the comparisons of HCL and AHCL with non-AID therapies indicated benefits of AID technology, which were clinically relevant in a few cases, with very low to low CoE. Compared with CSII, HCL therapy reduced it on average by 3.6% (2.3%; 4.8%; low CoE) and AHCL by 3.4% (1.6%; 5.2%; low CoE), whereas FCL was not effective (Table 1, Appendix 10.5). With respect to TBR <54 mg/dl, neither HCL nor AHCL or FCL were convincingly different from the non-AID control treatments with very low to low CoE, respectively (Table 1, Appendix 10.6). Based on the 95% PrIs, only AHCL therapy compared with CSII met the MIDs for TBR <70 mg/dl (Appendix 9.4, Figure G). All 95% PrIs indicated nonsuperiority of a future study with respect to TBR <54 mg/dl (Appendix 9.5, Figure G). According to the P-scores, the most effective treatments for reducing the TBRs <70 mg/dl and <54 mg/dl were SAP and FCL therapy (Table 2, Figure I and Table C in Appendices 9.4 and 9.5).

#### HbA1c

The comparisons of HCL and AHCL with non-AID therapies indicated a reduction in HbA1c levels with AID therapy, with very low to moderate CoE. A clinically relevant HbA1c reduction was achieved only when AHCL was compared with CSII (−1.0% (−1.4%; −0.7; moderate CoE)) (Table 1, Appendix 10.7). Based on the 95% PrIs, we found no difference in efficacy (Appendix 9.6, Figure G). According to the P-scores, the most effective treatments for lowering HbA1c levels were CSII and SAP therapies (Table 2, Figure I and Table C in Appendix 9.6).

## Discussion

We systematically collected and analysed all available evidence from RCTs directly comparing currently available insulin treatments, including HCL, AHCL, and FCL therapy, on the key outcomes of time in glycaemic ranges in people with type 1 diabetes. To our knowledge, this is the first NMA that includes FCL systems and focuses on longer-term interventions with a detailed CoE assessment. Using MIDs defined a priori as decision thresholds, we found that the use of AHCL systems in particular was associated with improved glycaemic outcomes compared with CSII. Overall, the CoE varied between very low and high, depending on the treatment under consideration. There was no indication of possibly harmful effects.

Our findings are largely consistent with those of previous systematic reviews and meta-analyses. According to a guideline concerning insulin delivery in children and adolescents, HCL and AHCL therapy increased the TIR by approximately 10%–15% compared with CSII, SAP, PLGM, or AHCL to HCL therapy.^3^ Our results largely confirm this statement, but we observed greater effects of HCL and AHCL compared with CSII and a smaller difference between AHCL and HCL therapy in terms of the TIR achieved. An expert committee has estimated improvements in TIR through the use of AID of 9%–16% and reductions in HbA1c levels of 0.3%–0.5% without causing an increased risk of hypoglycaemia which is also supported by our findings.^2^ Similar to our results, previous NMAs estimated a mean TIR improvement by AID therapy compared with SAP of up to 20% depending on the algorithm.^4^^,^^7^ Another NMA reported that HCL therapy, compared with SAP therapy, was associated with a 0.6% (0.2%; 0.9%) lower mean HbA1c level (very low CoE).^5^

The strengths of our study include the extensive literature search and the broad search strategy. We followed the current guidelines for conducting and reporting systematic reviews and NMAs. Study selection, data extraction, RoB evaluation and GRADE assessment were performed by two reviewers independently according to best practices. Compared with other recently published systematic reviews with pairwise meta-analyses on the use of AID systems, our study included the largest number of RCTs.^72^, ^73^, ^74^ A key strength of our approach was the addition of indirect evidence to direct comparisons. The use of NMA enabled us to provide an integrated comparison of seven insulin treatment modalities and particularly to estimate the relative effects of treatments that were not directly compared in RCTs. To our knowledge, no previous NMA has included interventions with HCL, AHCL, and FCL. Another strength lies in the comprehensive GRADE assessment, which we presented transparently for both the standard pairwise meta-analyses and the NMAs. The assessment provides a differentiated picture of the evidence base for various insulin treatment alternatives that people with type 1 diabetes and clinicians can choose from.

Limitations of our study include the relatively small number of RCTs resulting in a low median number of studies for direct comparisons, a small number of participants except for the comparison of AHCL and SAP therapy, and primary and secondary outcomes characterised by heterogeneity of the effect estimates. We tried to address this issue via sensitivity analyses and did not find any major problems; however, important aspects, such as people's competence in using different technologies, nutrition, physical activity and psychosocial aspects, could not be accounted for. In addition, we categorised similar AID systems in terms of the degree of automation. As these systems also have important differences despite their great similarities (e.g., different glucose target levels of AHCL systems), this approach has probably contributed to the heterogeneity of the results. Owing to the lack of sufficient data, network meta-regression was not applied for further evaluation of effect modification. As the assumption of transitivity could not be clearly verified, the GRADE domain of intransitivity was downgraded conservatively by default for indirect comparisons. The impact of possible effect-modifying factors should be further investigated as more extensive RCTs become available.

Notably, the effects estimated have varying and often (very) low CoEs. We attributed the main reason for the low CoE in both the pairwise meta-analyses and the NMAs to the downgrading due to RoB and indirectness caused by mixed control groups for the primary outcome, and to additional imprecision for the secondary outcomes. Although the P-scores showed that FCL therapy performed better than AHCL therapy in terms of TIR and TBRs, we believe that these results have not yet been well established. FCL therapy was examined in only one of the included studies. The participants in this RCT had very low TIR (32%) at baseline and still low TIR at follow-up despite good improvement, and there were some concerns regarding the randomisation process because it was not reported whether the allocation sequence was concealed until participants were enrolled and assigned to interventions.^39^ In addition, the comparison-adjusted funnel plots suggested global publication bias, which limits the validity of our network estimates. Future high-quality studies with large sample sizes, nonmixed control treatments and long durations might help to fully clarify the glycaemic benefits resulting from the use of AID systems.

We wanted to provide a comprehensive overview and therefore did not exclude pregnant women or children with newly diagnosed type 1 diabetes. Nonetheless, the problem of generalising our results to all persons with type 1 diabetes is similar to that of all meta-analyses of RCTs. Participants in RCTs are selective study populations and may therefore be among the most motivated and active people, which may lead to an overestimation of efficacy. However, recent observational data reported similar results regarding TIR, HbA1c levels and TBR <70 mg/dl.^75^ In addition, the rapid pace of technological development means that systematic reviews are difficult to keep up to date. With respect to this review, to our knowledge, no studies have been published since the systematic search was completed, which could have significantly changed the results.

In particular, our results show the efficacy of AHCL systems compared with non-AID systems in terms of glycaemic control in people with type 1 diabetes. However, reducing the TBR and HbA1c remains a challenge for patients, regardless of which AID system they use. The results provide information for clinical decisions on the actual achievable benefits of HCL, AHCL and FCL therapy compared with other insulin treatment modalities without neglecting heterogeneity and uncertainties in this context. The scope and depth of our results may be particularly useful for advocacy and evidence-based recommendations.

This systematic review and NMA provides a comprehensive overview of the currently available evidence on the efficacy of AID systems. Despite the partially limited CoE, this review can guide the choice of diabetes technologies for physicians and people with type 1 diabetes. Overall, AID systems are superior to other currently available insulin treatments for type 1 diabetes in terms of TIR, but their superiority regarding other glycaemic outcomes has not yet been clearly demonstrated.

## Contributors

Study conception and design: ASP, JR. Funding acquisition: ASP. Data extraction and verification: ASP, NSM. Data curation and formal analysis: ASP, MW, JR, OK. Visualization: ASP, MW, JR. Supervision: SS, JR. Writing–original draft preparation: ASP. Writing–review & editing: all authors. All authors commented on previous versions of the manuscript, read and approved the final version of the manuscript.

## Data sharing statement

Data in this systematic review with meta-analysis were extracted from published studies available on the internet. Request for the meta-analysis data can be made by contacting the corresponding author.

## Declaration of interests

There are no potential conflicts relevant to this article.
