# Supplementary material for: Efficacy of automated insulin delivery systems in people with type 1 diabetes: a systematic review and network meta-analysis of outpatient randomised controlled trials
Source: eClinicalMedicine. 2025 Apr 11;82:103190. doi: 10.1016/j.eclinm.2025.103190 (PMC12017971; doi:10.1016/j.eclinm.2025.103190)
Supplement: Supplementary Figures and Tables [file mmc1.pdf]

Online-Only Supplemental Material

Appendix to:

Efficacy of automated insulin delivery systems in people with type 1 diabetes: A systematic review and network meta-analysis of outpatient randomised controlled trials

Table of contents

1 Supplement to methods ... 3
1.1 Search strategy for MEDLINE via PubMed ... 3
1.2 Summary of included interventions ... 4
1.3 Primary and secondary outcomes ... 5
1.4 GRADE assess reasons for rating down ... 6
2 Supplement to systematic review ... 8
2.1 Excluded studies ... 8
2.2 Included studies ... 26
3 Risk of bias evaluation ... 31
4 Supplement to pairwise meta-analysis ... 33
4.1 Summary results of pairwise meta-analyses ... 33
4.2 Pairwise meta-analyses TIR ... 33
4.3 Pairwise meta-analyses TAR >180 mg/dl ... 37
4.4 Pairwise meta-analyses TAR >250 mg/dl ... 40
4.5 Pairwise meta-analyses TBR <70 mg/dl ... 43
4.6 Pairwise meta-analyses TBR <54 mg/dl ... 47
4.7 Pairwise meta-analyses HbA1c ... 50
5 Supplement to sensitivity analysis ... 54
5.1 Sensitivity analyses TIR ... 54
5.2 Sensitivity analyses TAR >180 mg/dl ... 61
5.3 Sensitivity analyses TAR >250 mg/dl ... 68
5.4 Sensitivity analyses TBR <70 mg/dl ... 75
5.5 Sensitivity analyses TBR <54 mg/dl ... 82
5.6 Sensitivity analyses HbA1c ... 89
6 Supplement to leave-one-out analysis ... 94
6.1 Leave-one-out TIR ... 94

6.2 Leave-one-out TAR >180 mg/dl ... 96
6.3 Leave-one-out TAR >250 mg/dl ... 99
6.4 Leave-one-out TBR <70 mg/dl ... 101
6.5 Leave-one-out TBR <54 mg/dl ... 103
6.6 Leave-one-out HbA1c ... 106
7 Supplement to small-study bias assessment ... 108
7.1 Small-study bias TIR ... 108
7.2 Small-study bias TAR >180 mg/dl ... 109
7.3 Small-study bias TAR >250 mg/dl ... 110
7.4 Small-study bias TBR <70 mg/dl ... 110
7.5 Small-study bias TBR <54 mg/dl ... 111
7.6 Small-study bias HbA1c ... 112
8 Supplement to GRADE CoE assessment from pairwise meta-analysis ... 113
9 Supplement to network meta-analysis ... 120
9.1 Network meta-analysis TIR ... 120
9.2 Network meta-analysis TAR >180 mg/dl ... 129
9.3 Network meta-analysis TAR >250 mg/dl ... 138
9.4 Network meta-analysis TBR <70 mg/dl ... 147
9.5 Network meta-analysis TBR <54 mg/dl ... 156
9.6 Network meta-analysis HbA1c ... 165
9.7 Global consistencies ... 173
10 Supplement to GRADE CoE assessment from network meta-analysis ... 173
10.1 Applying GRADE to network meta-analysis ... 173
10.2 CoE assessment TIR network meta-analysis ... 174
10.3 CoE assessment TAR >180 mg/dl network meta-analysis ... 176
10.4 CoE assessment TAR >250 mg/dl network meta-analysis ... 178
10.5 CoE assessment TBR <70 mg/dl network meta-analysis ... 180
10.6 CoE assessment TBR <54 mg/dl network meta-analysis ... 182
10.7 CoE assessment HbA1c network meta-analysis ... 184
11 References ... 186

1 Supplement to methods

1.1 Search strategy for MEDLINE via PubMed

Search number	Query	Description	Results (April 17, 2023)	Results (January 8, 2025)
#1	"diabetes mellitus, type 1"[mh] OR "diabetes mellitus type 1"[tw] OR "diabetes type 1"[tw] OR "type 1"[tw] OR T1D[tw] OR T1DM[tw]	Population: type 1 diabetes	101,481	298,926
#2	"Pancreas, Artificial*"[mh] OR "automated insulin"[tw] AND (delivery[tw] OR dosing[tw]) OR ("bioartificial organs"[mh] AND (pancrea*[tw] OR insulin[tw] OR diabet*[tw])) OR ("Bionics"[mh] AND (pancrea*[tw] OR insulin[tw] OR diabet*[tw])) OR "artificial pancrea*"[tw] OR ("artificial endocrine"[tw] AND "pancrea*"[tw]) OR "bioartificial pancrea*"[tw] OR "bionic pancrea*"[tw] OR "automated pancrea*"[tw] OR "artificial beta cell*"[tw] OR "artificial b-cell*"[tw] OR ((closed-loop*[tw] OR closed loop[tw]) AND (pancrea*[tw] OR insulin[tw] OR diabet*[tw]))	Intervention: AID (version A)	3,597	4,301
#3	"Insulin Infusion Systems"[mh] OR "Insulin/administration and dosage"[mh] OR "Infusion pumps, implantable"[mh] OR "Insulin/therapeutic use"[mh] OR ("continuous subcutaneous"[tw] OR automated[tw] AND "insulin infusion"[tw]) OR CSII[tw] OR pump[tw] OR delivery [tw]	Infusion pump systems	876,377	971,131
#4	"blood glucose self monitoring"[mh] OR "Glycemic Control/methods"[mh] OR (glucose [tw] AND (monitor*[tw] OR meter*[tw] OR sensor*[tw] OR augment*[tw])) OR "glucose measurement"[tw] OR glucometer[tw] OR CGM[tw]	Glucose sensor	61,539	68,600
#5	algorithm[tw] OR program*[tw] OR "mobile application"[tw] OR "mobile phone"[tw] OR computer[tw] OR smartphone[tw] OR "connected device"[tw] OR "smart watch"[tw] OR wearable[tw]	Algorithm	1,915,883	2,385,313
#6	#3 AND #4 AND #5	Intervention: AID (version B)	1,541	1,769
#7	#1 AND (#2 OR #6)	Type 1 diabetes, AID	2,586	3,277
#8	"randomized controlled trial"[pt] OR "controlled clinical trial"[pt] OR randomized[tiab] OR placebo[tiab] OR "drug therapy"[sh] OR randomly[tiab] OR trial[ti] OR groups[tiab]	RCTs in humans	5,535,048	6,055,789
#9	"animals"[mh] NOT "humans"[mh]		5,111,128	5,293,496
#10	#8 NOT #9		4,827,661	5,302,489
#11	#7 AND #10	Type 1 diabetes, AID, RCTs in humans	1,735	2,249

1.2 Summary of included interventions

Abbreviation	Terminology	Description *
MDI	Multiple Daily Injection	User injects a long acting insulin once or twice daily as a basal dose and has further injections of rapid acting insulin at each meal time via an insulin pen
CSII	Continuous Subcutaneous Insulin Infusion	Insulin pump delivers insulin at pre-selected basal rates, with additional user-initiated bolus infusions
SAP	Sensor-Augmented Pump therapy	Insulin pump with use of a continuous glucose measurement (CGM) either on a separate device or displayed directly on the pump; insulin delivery is not altered on the basis of sensor glucose values
(P)LGM	(Predictive) Low Glucose Management	Insulin pump system that suspends insulin delivery for actual hypoglycaemia due to sensor glucose value or for predicted hypoglycaemia
HCL	Hybrid Closed-Loop technology, Hybrid AID	Insulin pump systems that automatically adjust basal insulin delivery in response to sensor glucose values; user needs to dose prandial insulin manually
AHCL	Advanced Hybrid Closed-Loop technology, Advanced Hybrid AID	Insulin pump systems that automatically adjust basal insulin delivery, but also have the capacity to deliver automatic correction boluses; user needs to dose prandial insulin manually
FCL	Full Closed-Loop technology, Full AID	AID system that automatically adjusts all insulin delivery, including prandial insulin without interaction between the user and the system

* The definitions of current treatment options for type 1 diabetes were adapted from [1]

1.3 Primary and secondary outcomes

Pre-defined primary outcome:

- Time in range (TIR): percent of time spent with glucose concentrations within the target range of 70–180 mg/dl (3.9–10.0 mmol/L).

Pre-defined secondary outcome:

- Haemoglobin A_{1c} (HbA_{1c});
- Hypoglycaemia:
    - Level 1: percent of time spent with a glucose concentration of <70 mg/dl (3.9 mmol/L) and ≥54 mg/dl (3.0 mmol/L);
    - Level 2 time below range (TBR): percent of time spent with a glucose concentration of <54 mg/dl (3.0 mmol/L);
    - Level 3: number of severe events (per time unit) characterised by altered mental and/or physical status requiring assistance;
- Hyperglycaemia:
    - Level 1 time above range (TAR): percent of time spent with a glucose concentration of >180 mg/dl (10 mmol/L) and ≤250 mg/dl (13.9 mmol/L);
    - Level 2 time above range (TAR): percent of time spent with a glucose concentration of >250 mg/dl (13.9 mmol/L);
- Diabetic ketoacidosis: number of events (per time unit) of elevated serum or urine ketone concentrations and serum bicarbonate concentrations of <15 mmol/L or a blood pH of <7.3; and
- Patient-reported outcomes:
    - quality of life;
    - diabetes distress;
    - fear of hypoglycaemia.

Deviations from the protocol:
Some predefined outcomes could not be meta-analytically summarised. Patient-reported outcomes were not assessed or inconsistently reported, so no similarity between studies could be assumed for these outcomes. Definitions of severe hypoglycaemic events and diabetic ketoacidosis were inconsistently reported as safety outcomes without sufficient duration or study power to detect significant differences. Hypoglycaemia level 1 and hyperglycaemia level 1 were reported by only a small number of studies. However, the percentage of time spent at a glucose concentration of <70 mg/dl or >180 mg/dl was reported in more studies. Therefore, we evaluated hypoglycaemia level 1/2 (<70 mg/dl) and hyperglycaemia level 1/2 (>180 mg/dl) instead of hypoglycaemia level 1 and hyperglycaemia level 1.

1.4 GRADE assess reasons for rating down

GRADE for direct estimates (≥ 2 studies)	
**Risk of bias (RoB):**	We used the RoB2 tool and considered the weight of each study in the pairwise comparisons and in the direct estimates of the NMA, respectively.
not serious	Most studies had a low RoB (<50% some concerns, 0-25% high RoB)
serious	RoB was unclear (≥50% some concerns)
	>25-50% of the studies had a high RoB
very serious	>50% of the studies had a high RoB
**Inconsistency:**	We evaluated the forest plots of the pairwise meta-analysis (similarity of point estimates, overlap of confidence intervals). I^2 and p value for statistical heterogeneity were used as supportive information.
not serious	Estimates pointed to same directions and the 95% CIs overlapped.
serious	Estimates pointed to same directions but were appreciably different and the 95% CIs did not overlap.
	Estimates pointed to different directions but the 95% CIs overlapped.
	One of two estimates was zero and the 95% CIs did not overlap.
very serious	Estimates pointed to different directions and the 95% CIs did not overlap.
**Indirectness:**	We considered differences in population, outcome and intervention.
not serious	Population differences, unless we had a compelling reason to suspect that the biology of the population of interest differed from the population tested
	Outcome differences, unless we used surrogate outcomes different from the prespecified outcomes
	Most studies were clearly attributable to a single intervention (≤50% mixed intervention and/or control group).
serious	>50% of the studies had mixed intervention and/or control groups.
very serious	We did not expect a very serious degree of indirectness, as all included studies met the PICOSs.
**Imprecision:**	We evaluated the 95% confidence intervals (95% CIs) regarding crossing the threshold values for the minimally important difference (MID) and inclusion of the null value, and considered the sample size in the control and intervention groups. MID:<br>• TIR 70-180 mg/dl: +/-5% [2]<br>• TAR >180 mg/dl: +/-5% [3]<br>• TAR >250 mg/dl: +/-1%<br>• TBR <70 mg/dl: +/-1% [4]<br>• TBR <54 mg/dl: +/-0.5% [2]<br>• HbA1c [%]: +/-0.5% [2,5,6]
not serious	Null value and MID not in the 95% CI and ≥400 participants per group
serious	Null value not in the 95% CI, MID not in the 95% CI and <400 participants per group
	Null value not in the 95% CI, MID in the 95% CI and ≥400 participants per group
	Null value in the 95% CI and MID not in the 95% CI and ≥400 participants per group
very serious	Null value not in the 95% CI, MID in the 95% CI and <400 participants per group
	Null value in the 95% CI and <400 participants per group
extremely serious	Extreme values included in the 95% CI
**Publication bias:**	Visual inspection of funnel plots of the pairwise meta-analyses, supported by Egger's test and the trim-and-fill-method
not serious	<10 studies
	≥10 studies and no publication bias was detected
serious	≥10 studies and publication bias was detected
**GRADE for direct estimates (single study)**	
**RoB:**	We used the RoB2 tool.
not serious	low RoB
serious	RoB was unclear (some concerns)
very serious	high RoB

**Inconsistency:**	Not applicable (not serious)
**Indirectness:**	We considered differences in the population, outcome and intervention.
not serious	Population differences, unless we had a compelling reason to suspect that the biology of the population of interest differed from the population tested
	Outcome differences, unless we used surrogate outcomes different from the prespecified outcomes
	Study was clearly attributable to the intervention and control group
serious	mixed intervention and/or control groups
very serious	We did not expect a very serious degree of indirectness, as all included studies met the PICOSs.
**Imprecision:**	See GRADE for direct estimates (≥ 2 studies)
**Publication bias:**	Not applicable (not serious)
**GRADE for indirect estimates**	
Starting point is the lowest of the preliminary certainty ratings for the direct comparisons that constitute the most dominant (in terms of number of studies and participants included) loop.	
**Intransitivity:**	Joint consideration of indirectness and intransitivity with more weight given to the assessments from direct comparisons that contribute more information (see contributions matrix)
not serious	Transitivity assessment suggests that distribution of effect modifiers is similar for all sources of direct evidence.
serious	No convincing evidence for the plausibility of the transitivity assumption, similarity of effect modifiers cannot be assured
	Insufficient evidence to judge transitivity (e.g., because of small number of studies)
**Imprecision:**	See GRADE for network estimates
**GRADE for network estimates (direct and/or indirect estimates available)**	
Starting point is the certainty rating of the dominating direct or indirect estimate (algorithm in [7]).	
**Incoherence:**	We evaluated the network side-split table. When direct and indirect estimates were incoherent, imprecision of direct and indirect estimates was assessed.
not serious	The direct and indirect estimates and the corresponding 95% CIs are coherent.
	The p value of network side-split analysis is >5%
serious	The p value of network side-split analysis is <5%
	Direct and indirect estimates imply different clinical decisions.
**Imprecision:**	We evaluated the width of the 95% CI.
not serious	The 95% CI did not contain the MID and/or null value.
serious	The 95% CI contained the MID and/or null value
very serious	The 95% CI was divided by the null value into two parts with a comparable ratio, or the mean was close to null.
	The point estimate reflects an important benefit, and the boundary of the CI least favorable to the intervention includes the possibility of harm.
Extremely serious	The 95% CI is very wide with the two boundaries of the 95% CI suggesting very different inferences.
**GRADE for network estimates (no direct and/or indirect estimates available)**	
**Incoherence:**	Not applicable (not serious)
**Imprecision:**	See GRADE for network estimates with direct and/or indirect estimates available

2 Supplement to systematic review

2.1 Excluded studies

First author	Year	Title	DOI	Exclusion reason
Franc, S.	2020	The Official Journal of ATTD Advanced Technologies & Treatments for Diabetes Conference Madrid, Spain-February 19-22, 2020	10.1089/dia.2020.2525.abstracts	Wrong study design
Buckingham, B.	2021	ATTD 2021 Invited Speaker Abstracts	10.1089/dia.2021.2525.abstracts	Wrong study design
Medtronic Diabetes	2020	MiniMed™ 670G System China Study for Type I Diabetic		Wrong study design
Tandem Diabetes Care Inc	2022	Adaptation of Insulin Delivery Settings to Improve Clinical Outcomes With AID Use		Wrong study design
Coutant, R.	2023	Hybrid Closed Loop Overcomes the Impact of Missed or Suboptimal Meal Boluses on Glucose Control in Children with Type 1 Diabetes Compared to Sensor-Augmented Pump Therapy	10.1089/dia.2022.0518	Wrong study design
Beck, R. W.	2023	The Relationship Between Percent Time <70 mg/dl and Percent Time <54 mg/dl Measured by Continuous Glucose Monitoring	10.1089/dia.2022.0462	Wrong study design
Garg, S. K.	2017	Glucose Outcomes with the In-Home Use of a Hybrid Closed-Loop Insulin Delivery System in Adolescents and Adults with Type 1 Diabetes	10.1089/dia.2016.0421	Wrong study design
Gawrecki, A.	2021	Safety and glycemic outcomes of do-it-yourself AndroidAPS hybrid closed-loop system in adults with type 1 diabetes	10.1371/journal.pone.0248965	Wrong study design
Pinsker, J. E.	2021	Use of the Interoperable Artificial Pancreas System for Type 1 Diabetes Management During Psychological Stress	10.1177/1932296820948566	Wrong study design
Bergenstal, R. M.	2016	Safety of a Hybrid Closed-Loop Insulin Delivery System in Patients With Type 1 Diabetes	10.1001/jama.2016.11708	Wrong study design
Steil, G. M.	2013	Algorithms for a closed-loop artificial pancreas: the case for proportional-integral-derivative control	10.1177/193229681300700623	Wrong study design
Petrovski	2022	Glycemic outcomes of Advanced Hybrid Closed Loop system in children and adolescents with Type 1 Diabetes, previously treated with Multiple Daily Injections (MiniMed 780G system in T1D individuals, previously treated with MDI)	10.1186/s12902-022-00996-7	Wrong study design
Gianini, A.	2022	Patient reported outcome measures in children and adolescents with type 1 diabetes using advanced hybrid closed loop insulin delivery	10.3389/fendo.2022.967725	Wrong study design
Bisio, A.	2021	Sleep and diabetes-specific psycho-behavioral outcomes of a new automated insulin delivery system in young children with type 1 diabetes and their parents	10.1111/pedi.13164	Wrong study design

Bisio, A.	2022	The Impact of a Recently Approved Automated Insulin Delivery System on Glycemic, Sleep, and Psychosocial Outcomes in Older Adults With Type 1 Diabetes: A Pilot Study	10.1177/1932296820986879	Wrong study design
Carlson, A. L.	2022	Safety and Glycemic Outcomes During the MiniMed Advanced Hybrid Closed-Loop System Pivotal Trial in Adolescents and Adults with Type 1 Diabetes	10.1089/dia.2021.0319	Wrong study design
Forlenza, G. P.	2022	Glycemic outcomes of children 2-6 years of age with type 1 diabetes during the pediatric MiniMed 670G system trial	10.1111/pedi.13312	Wrong study design
Nimri, R.	2021	Feasibility Study of a Hybrid Closed-Loop System with Automated Insulin Correction Boluses	10.1089/dia.2020.0448	Wrong study design
Ozaslan, B.	2022	Feasibility of Closed-Loop Insulin Delivery with a Pregnancy-Specific Zone Model Predictive Control Algorithm	10.1089/dia.2021.0521	Wrong study design
Benhamou, P. Y.	2022	The beneficial effects of closed-loop insulin delivery in patients with highly unstable type 1 diabetes eligible for islet transplantation are maintained over 6 months: An extension study of the DBLHU-WP10 trial	10.1111/dom.14654	Wrong study design
Kanapka, L. G.	2021	Extended Use of the Control-IQ Closed-Loop Control System in Children With Type 1 Diabetes	10.2337/dc20-1729	Wrong study design
Mauras, N.	2023	Utility and Safety of Backup Insulin Regimens Generated by the Bionic Pancreas: A Randomized Study	10.1089/dia.2022.0461	Wrong study design
Leelarathna, L.	2019	115-LB: Optimal Sampling Duration of Hybrid Closed-Loop Therapy to Determine Long-Term Glycemic Control in Adults with Type 1 Diabetes	10.2337/db19-115-LB	Wrong study design
Levy, C.	2023	ATTD 2023 Abstract Author Index	10.1089/dia.2023.2526.abstracts.index	Wrong study design
Gardner, D. S.	2023	ATTD 2023 Abstract Author Index	10.1089/dia.2023.2526.abstracts.index	Wrong study design
Schoelwer, M. J.	2022	Assessment for Predictors of Rise in Hemoglobin A1c During Extended Use of a Closed-Loop Control System	10.1089/dia.2021.0405	Wrong study design
Benhamou, P. Y.	2022	Efficacy of a Hybrid Closed-Loop Solution in Patients With Excessive Time in Hypoglycaemia: A Post Hoc Analysis of Trials With DBLG1 System	10.1177/19322968221128565	Wrong study design
Nct	2018	The International Diabetes Closed Loop (iDCL) Trial: clinical Acceptance of the Artificial Pancreas (DCLP3 Extension)		Wrong study design
Farhy, L.	2020	The Official Journal of ATTD Advanced Technologies & Treatments for Diabetes Conference Madrid, Spain-February 19-22, 2020	10.1089/dia.2020.2525.abstracts	Wrong study design
Bode, B.	2021	ATTD 2021 Invited Speaker Abstracts	10.1089/dia.2021.2525.abstracts	Wrong study design
Buckingham, B.	2018	The Official Journal of ATTD Advanced Technologies & Treatments for Diabetes Conference Austria, Vienna-February 14-17, 2018	10.1089/dia.2018.2525.abstracts	Wrong study design

Lynch, J.	2022	The Insulin-Only Bionic Pancreas Pivotal Trial Extension Study: A Multi-Center Single-Arm Evaluation of the Insulin-Only Configuration of the Bionic Pancreas in Adults and Youth with Type 1 Diabetes	10.1089/dia.2022.0341	Wrong study design
Beunen, K.	2024	Advanced Hybrid Closed-Loop Therapy Compared With Standard Insulin Therapy Intrapartum and Early Postpartum in Women With Type 1 Diabetes: A Secondary Observational Analysis From the CRISTAL Randomized Controlled Trial	10.2337/dc24-1320	Wrong study design
Elhenawy, Y. I.	2024	Performance of the MiniMed 780G system on mitigating menstrual cycle-dependent glycaemic variability	10.1111/dom.15891	Wrong study design
Al-Sofiani, M. E.	2024	A Real-World Prospective Study of the Effectiveness and Safety of Automated Insulin Delivery Compared With Other Modalities of Type 1 Diabetes Treatment During Ramadan Intermittent Fasting	10.2337/dc23-1968	Wrong study design
Pei, Y.	2023	Safety Event Outcomes and Glycemic Control with a Hybrid Closed-Loop System Used by Chinese Adolescents and Adults with Type 1 Diabetes Mellitus	10.1089/dia.2023.0234	Wrong study design
Miller, K. M.	2022	Benefit of Continuous Glucose Monitoring in Reducing Hypoglycemia Is Sustained Through 12 Months of Use Among Older Adults with Type 1 Diabetes	10.1089/dia.2021.0503	Wrong intervention
Air Liquide Santé International	2021	Evaluation of Satisfaction Regarding Home Healthcare Provider (HHP) Management of Type 1 Diabetic Patients Using a Closed-loop Automatic Insulin Delivery System		Wrong intervention
McGill University	2020	Alleviating Carbohydrate Counting for Patients With Type 1 Diabetes Using a Novel Insulin-plus-pramlintide Artificial Pancreas		Wrong intervention
Lawton, J.	2019	Ambassadors of hope, research pioneers and agents of change-individuals' expectations and experiences of taking part in a randomised trial of an innovative health technology: longitudinal qualitative study	10.1186/s13063-019-3373-9	Wrong intervention
Briganti, S. I.	2021	ATTD 2021 Invited Speaker Abstracts	10.1089/dia.2021.2525.abstracts	Wrong intervention
Forlenza, G. P.	2018	Predictive Low-Glucose Suspend Reduces Hypoglycemia in Adults, Adolescents, and Children With Type 1 Diabetes in an At-Home Randomized Crossover Study: Results of the PROLOG Trial	10.2337/dc18-0771	Wrong intervention
Petrovski, G.	2023	Simplified Meal Announcement Versus Precise Carbohydrate Counting in Adolescents With Type 1 Diabetes Using the MiniMed 780G Advanced Hybrid Closed Loop System: A Randomized Controlled Trial Comparing Glucose Control	10.2337/dc22-1692	Wrong intervention
Kropff, J.	2017	Psychological outcomes of evening and night closed-loop insulin delivery under free living conditions in people with Type 1 diabetes: a 2-month randomized crossover trial	10.1111/dme.13268	Wrong intervention

Balliro, C.	2018	The Official Journal of ATTD Advanced Technologies & Treatments for Diabetes Conference Austria, Vienna-February 14-17, 2018	10.1089/dia.2018.2525.abstracts	Wrong intervention
Stewart, Z. A.	2016	Closed-Loop Insulin Delivery during Pregnancy in Women with Type 1 Diabetes	10.1056/NEJMoa1602494	Wrong intervention
Thabit, H.	2014	Home use of closed-loop insulin delivery for overnight glucose control in adults with type 1 diabetes: a 4-week, multicentre, randomised crossover study	10.1016/S2213-8587(14)70114-7	Wrong intervention
Hirsch, I. B.	2024	A Randomized Comparison of Postprandial Glucose Excursion Using Inhaled Insulin Versus Rapid-Acting Analog Insulin in Adults With Type 1 Diabetes Using Multiple Daily Injections of Insulin or Automated Insulin Delivery	10.2337/dc24-0838	Wrong intervention
Kaiserman, K.	2023	1805-PUB: Results of a Two-Part Feasibility Study Assessing Inhaled Insulin for Meals with MDI or Automated Insulin Delivery (AID) Compared with Standard AID Therapy	10.2337/db23-1805-PUB	Wrong intervention
Sansum Diabetes Research Institute	2019	Artificial Pancreas With Different Stress Assessments in the Outpatient Setting		Wrong duration
Steno Diabetes Center Copenhagen	2021	Dual-Hormone Closed-Loop Glucose Control in Adolescents With Type 1 Diabetes		Wrong duration
Clarke, W. L.	2009	Closed-loop artificial pancreas using subcutaneous glucose sensing and insulin delivery and a model predictive control algorithm: the Virginia experience	10.1177/193229680900300506	Wrong duration
Garcia-Tirado, J.	2021	Anticipation of Historical Exercise Patterns by a Novel Artificial Pancreas System Reduces Hypoglycemia During and After Moderate-Intensity Physical Activity in People with Type 1 Diabetes	10.1089/dia.2020.0516	Wrong duration
Tsoukas, M. A.	2021	A fully artificial pancreas versus a hybrid artificial pancreas for type 1 diabetes: a single-centre, open-label, randomised controlled, crossover, non-inferiority trial	10.1016/S2589-7500(21)00139-4	Wrong duration
Abraham, M. B.	2019	2407-PUB: Glycemic Patterns Derived from Masked Continuous Glucose Monitoring in Adolescents with Type 1 Diabetes on Contemporary Management	10.2337/db19-2407-PUB	Wrong outcomes
Roberts, A.	2022	Hybrid closed-loop therapy with a first-generation system increases confidence and independence in diabetes management in youth with type 1 diabetes	10.1111/dme.14907	Wrong outcomes
Farrington, C.	2018	Women's Experiences of Day-and-Night Closed-Loop Insulin Delivery During Type 1 Diabetes Pregnancy	10.1177/1932296818800065	Wrong outcomes
Barnard, K. D.	2015	Psychosocial aspects of closed- and open-loop insulin delivery: closing the loop in adults with Type 1 diabetes in the home setting	10.1111/dme.12706	Superseded / more information in other report

Castellanos, L. E.	2023	The Insulin-Only Bionic Pancreas Improves Glycemic Control in Non-Hispanic White and Minority Adults and Children With Type 1 Diabetes	10.2337/dc22-1478	Superseded / more information in other report
Cordero, T. L.	2017	The Effect of Prior Continuous Glucose Monitoring Use on Glycemic Outcomes in the Pivotal Trial of the MiniMed() 670G Hybrid Closed-Loop System	10.1089/dia.2017.0208	Superseded / more information in other report
Emami, A.	2017	Behavioral Patterns and Associations with Glucose Control During 12-Week Randomized Free-Living Clinical Trial of Day and Night Hybrid Closed-Loop Insulin Delivery in Adults with Type 1 Diabetes	10.1089/dia.2016.0307	Superseded / more information in other report
O'Malley, G.	2021	Clinical Management and Pump Parameter Adjustment of the Control-IQ Closed-Loop Control System: Results from a 6-Month, Multicenter, Randomized Clinical Trial	10.1089/dia.2020.0472	Superseded / more information in other report
Kubilay, E.	2023	Lived experience of older adults with type 1 diabetes using closed-loop automated insulin delivery in a randomised trial	10.1111/dme.15020	Superseded / more information in other report
Lee, M. H.	2024	Hybrid Closed-Loop Versus Manual Insulin Delivery in Adults With Type 1 Diabetes: A Post Hoc Analysis Using the Glycemia Risk Index	10.1177/19322968241231307	Superseded / more information in other report
Thabit, H.	2024	Impact of hybrid closed-loop insulin delivery on cardiac rhythm in older adults with type 1 diabetes: A post hoc analysis of trial data	10.1111/dom.15366	Superseded / more information in other report
Hood, K. K.	2024	Patient reported outcomes (PROs) and user experiences of young children with type 1 diabetes using t:slim X2 insulin pump with control-IQ technology	10.1016/j.diabres.2024.111114	Superseded / more information in other report
Weissberg-Benchell, J.	2023	Psychosocial Impact of the Insulin-Only iLet Bionic Pancreas for Adults, Youth, and Caregivers of Youth with Type 1 Diabetes	10.1089/dia.2023.0238	Superseded / more information in other report
Ware, J.	2024	Effect of 48 Months of Closed-Loop Insulin Delivery on Residual C-Peptide Secretion and Glycemic Control in Newly Diagnosed Youth With Type 1 Diabetes: A Randomized Trial	10.2337/dc24-0360	Superseded / more information in other report
Schneider-Utaka, A. K.	2023	Patient-reported outcomes for older adults on CamAPS FX closed loop system	10.1111/dme.15126	Superseded / more information in other report
Boughton, C. K.	2024	Efficacy and Mechanism Evaluation	10.3310/KTFR5698	Superseded / more information in other report
Roberts, A.	2024	"I Think I Could Have Used It Better□": Experiences of Youth with High HbA1c Commencing Advanced Hybrid Closedâ€□Loop Therapy in a Clinical Trial Settingâ€”A Qualitative Research	10.1155/2024/6260002	Superseded / more information in other report

Madrid-Valero, J. J.	2023	Sleep Quality and Quantity in Caregivers of Children with Type 1 Diabetes Using Closed-Loop Insulin Delivery or a Sensor-Augmented Pump	10.1155/2023/7937007	Superseded / more information in other report
Lai, S. T.	2024	Parental perspectives following the implementation of advanced hybrid closed-loop therapy in children and adolescents with type 1 diabetes and elevated glycaemia	10.1111/dme.15448	Superseded / more information in other report
Edd, S. N.	2023	Twelve-month results of the ADAPT randomized controlled trial: Reproducibility and sustainability of advanced hybrid closed-loop therapy outcomes versus conventional therapy in adults with type 1 diabetes	10.1111/dom.15217	Superseded / more information in other report
Lee, M. H.	2024	Hybrid Closed Loop in Adults With Type 1 Diabetes and Severely Impaired Hypoglycemia Awareness	10.1177/19322968241245627	Superseded / more information in other report
Quintanilha, M.	2024	Women's and Partners' Experiences With a Closed-loop Insulin Delivery System to Manage Type 1 Diabetes in the Postpartum Period	10.1016/j.jcjd.2024.08.005	Superseded / more information in other report
Zeng, B.	2022	Dual-hormone artificial pancreas for glucose control in type 1 diabetes: A meta-analysis	10.1111/dom.14781	Review
Pease, A.	2020	Time in Range for Multiple Technologies in Type 1 Diabetes: A Systematic Review and Network Meta-analysis	10.2337/dc19-1785	Review
Garg, S. K.	2019	Closed-loop insulin delivery systems for patients with diabetes	10.1016/S2589-7500(19)30007-X	Review
Jiao, X.	2022	Better TIR, HbA1c, and less hypoglycemia in closed-loop insulin system in patients with type 1 diabetes: a meta-analysis	10.1136/bmjdrc-2021-002633	Review
Fang, Z.	2022	Efficacy and safety of closed-loop insulin delivery versus sensor-augmented pump in the treatment of adults with type 1 diabetes: a systematic review and meta-analysis of randomized-controlled trials	10.1007/s40618-021-01674-6	Review
Kumareswaran, K.	2011	Meta-analysis of overnight closed-loop randomized studies in children and adults with type 1 diabetes: the Cambridge cohort	10.1177/193229681100500606	Review
Zhang, G.	2023	Advances in Insulin Infusion Set in the New Era of Automated Insulin Delivery: A Systematic Review	10.1177/19322968221145731	Review
Graf, A.	2017	Overnight Counter-Regulatory Hormone Levels and Next Day Glycemia in Adults with Type 1 Diabetes During Closed-Loop Insulin Delivery Versus Sensor-Augmented Pump with Low-Glucose Suspend	10.1089/dia.2017.0049	Protocol/abstract/letter/comment/editorial
Lee, S. W.	2019	The Official Journal of ATTD Advanced Technologies & Treatments for Diabetes Conference Berlin, Germany—February 20–23, 2019	10.1089/dia.2019.2525.abstracts	Protocol/abstract/letter/comment/editorial
Lockhart, M.	2023	In T1DM, open-source automated insulin delivery increased glucose time in target vs. sensor-augmented pumps	10.7326/J22-0108	Protocol/abstract/letter/comment/editorial
Actrn	2016	The effect of Hybrid Closed Loop insulin delivery on glucose control in patients with type 1 diabetes aged 12 - 25 years	10.1001/jamapediatrics.2021.3965	Protocol/abstract/letter/comment/editorial

Fuchs, J.	2021	57(th) EASD Annual Meeting of the European Association for the Study of Diabetes	10.1007/s00125-021-05519-y	Protocol/abstract/letter/comment/editorial
de Bock, M.	2018	Effect of 6 months hybrid closed-loop insulin delivery in young people with type 1 diabetes: a randomised controlled trial protocol	10.1136/bmjopen-2017-020275	Protocol/abstract/letter/comment/editorial
Stewart, Z. A.	2016	Young Diabetologist and Endocrinologist Travel Award	10.1111/dme.4_13047	Protocol/abstract/letter/comment/editorial
Sherwood, J. S.	2018	Poster Session Abstracts	10.1002/ppul.24152	Protocol/abstract/letter/comment/editorial
Koshmeleva, M.	2018	Poster Abstracts	10.1111/pedi.12746	Protocol/abstract/letter/comment/editorial
Gonder-Frederick, L.	2019	79-LB: The Relationship between Diabetes Distress and Technology Experience in a Closed-Loop Control (CLC) Trial	10.2337/db19-79-LB	Protocol/abstract/letter/comment/editorial
Charpentier, G.	2019	55(th) EASD Annual Meeting of the European Association for the Study of Diabetes : Barcelona, Spain, 16 - 20 September 2019	10.1007/s00125-019-4946-6	Protocol/abstract/letter/comment/editorial
Levy, C. J.	2020	100-LB: Closed-Loop Control Reduces Hypoglycemia without Increased Hyperglycemia in Subjects with Increased Prestudy Hypoglycemia: Results from the iDCL DCLP3 Randomized Trial	10.2337/db20-100-LB	Protocol/abstract/letter/comment/editorial
Snaith, J. R.	2021	Technologies in the management of type 1 diabetes	10.5694/mja2.50946	Protocol/abstract/letter/comment/editorial
Ng, S. M.	2021	Poster Abstract	10.1111/pedi.13269	Protocol/abstract/letter/comment/editorial
Balliro, C. A.	2022	96-LB: Safety of Glucose Regulation by the Bionic Pancreas without Continuous Glucose Monitoring Input	10.2337/db22-96-LB	Protocol/abstract/letter/comment/editorial
Desalvo, D.	2022	33-OR: ADA Presidents' Select Abstract: Glycemic Outcomes over 12 Months in Very Young Children with the Omnipod 5 Automated Insulin Delivery (AID) System	10.2337/db22-33-OR	Protocol/abstract/letter/comment/editorial
Criego, A.	2022	58(th) EASD Annual Meeting of the European Association for the Study of Diabetes : Stockholm, Sweden, 19 - 23 September 2022	10.1007/s00125-022-05755-w	Protocol/abstract/letter/comment/editorial
Renard, E.	2023	ATTD 2023 Abstract Author Index	10.1089/dia.2023.2526.abstracts.index	Protocol/abstract/letter/comment/editorial
Tandem Diabetes Care Inc	2021	Control-IQ Technology 2.0 Adult and Adolescent Feasibility Study		Protocol/abstract/letter/comment/editorial
Medtronic Diabetes	2020	Feasibility Studies of Personalized Closed Loop		Protocol/abstract/letter/comment/editorial
Centre d'Etudes et de Recherche pour l'Intensification du Traitement du Diabète	2019	Diabeloop for Kids		Protocol/abstract/letter/comment/editorial
Sidra Medical	2021	Simple Initiation of Advanced Hybrid Closed Loop System		Protocol/abstract/letter/comment/editorial

Novo Nordisk	2019	Research Study to Look at Fast-acting Insulin Aspart With the Insulin Pump System 'iLet™' in Adults With Type 1 Diabetes	Protocol/abstract/letter/comment/editorial
Kinderkrankenhaus auf der Bult	2018	Comparison of Two Management Systems in Patients With Type 1 Diabetes (Pediatric SmartHome)	Protocol/abstract/letter/comment/editorial
University of Virginia	2018	The VRIF Trial: Hypoglycemia Reduction With Automated-Insulin Delivery System	Protocol/abstract/letter/comment/editorial
University of Virginia	2019	A Study of t:Slim X2 With Control-IQ Technology	Protocol/abstract/letter/comment/editorial
University of Virginia	2019	The International Diabetes Closed Loop (iDCL) Trial: Clinical Acceptance of the Artificial Pancreas (DCLP3 Extension)	Protocol/abstract/letter/comment/editorial
Sansum Diabetes Research Institute	2019	Model Predictive Control (MPC) Artificial Pancreas vs. Sensor Augmented Pump (SAP)/Predictive Low Glucose Suspend (PLGS) With Different Food Choices in the Outpatient Setting	Protocol/abstract/letter/comment/editorial
University of Virginia	2021	Adaptive Biobehavioral Control (ABC) of Automated Insulin Delivery	Protocol/abstract/letter/comment/editorial
Sheba Medical Center	2020	Unannounced Meal Handling of Advanced Closed Loop Insulin Delivery in Monitored Condition	Protocol/abstract/letter/comment/editorial
Oregon, Health	2021	Two Way Crossover Closed Loop Study R-AP vs MPC	Protocol/abstract/letter/comment/editorial
Oregon, Health	2021	Two Way Crossover Closed Loop Study MPC vs FMPD	Protocol/abstract/letter/comment/editorial
Sansum Diabetes Research Institute	2018	Safety and Feasibility Evaluation of the APS APP	Protocol/abstract/letter/comment/editorial
University of Virginia	2018	The International Diabetes Closed Loop (iDCL) Trial: Clinical Acceptance of the Artificial Pancreas	Protocol/abstract/letter/comment/editorial
Sheba Medical Center	2021	Meal Handling of Advanced Closed Loop Insulin Delivery	Protocol/abstract/letter/comment/editorial
Massachusetts General Hospital	2018	The Insulin-Only Bionic Pancreas Bridging Study	Protocol/abstract/letter/comment/editorial
Massachusetts General Hospital	2019	The Bihormonal iLet Bionic Pancreas Feasibility Study	Protocol/abstract/letter/comment/editorial
Jaeb Center for Health Research	2020	The Insulin-Only Bionic Pancreas Pivotal Trial	Protocol/abstract/letter/comment/editorial
Massachusetts General Hospital	2018	The Insulin-Only Bionic Pancreas Bridging Study- Pediatric Transitional Study	Protocol/abstract/letter/comment/editorial
Centre d'Etudes et de Recherche pour l'Intensification du Traitement du Diabète	2019	Diabeloop for Highly Unstable Type 1 Diabetes	Protocol/abstract/letter/comment/editorial

University of Cambridge	2019	The Artificial Pancreas in Very Young Children With T1D		Protocol/abstract/letter/comment/editorial
University of Cambridge	2019	24/7 Closed-loop in Older Subjects With Type 1 Diabetes		Protocol/abstract/letter/comment/editorial
University of Virginia	2020	Fully Automated Closed Loop Control in Adolescents With Type 1 Diabetes		Protocol/abstract/letter/comment/editorial
Rabin Medical Center	2020	An Open-label, Two-center, Randomized, Cross-over Study to Evaluate the Safety and Efficacy of Glycemic Control Using Hybrid-closed Loop vs. Advanced Hybrid Closed-loop in Young Subjects With Type 1 Diabetes		Protocol/abstract/letter/comment/editorial
Hospital Italiano de Buenos Aires	2021	Is the Artificial Pancreas, Without Carbohydrate Counting, Efficient and Safe in an Outpatient Setting		Protocol/abstract/letter/comment/editorial
Breton, M	2021	The Pediatric Artificial Pancreas (PEDAP) Trial of Control-IQ Technology in Young Children in Type 1 Diabetes		Protocol/abstract/letter/comment/editorial
Eli Lilly Company	2019	A Study of an Automated Insulin Delivery System in Adult Participants With Type 1 Diabetes Mellitus During Meal Challenges		Protocol/abstract/letter/comment/editorial
Eli Lilly Company	2018	A Study of an Automated Insulin Delivery System in Adult Participants With Type 1 Diabetes Mellitus (T1D)		Protocol/abstract/letter/comment/editorial
Eli Lilly Company	2019	A Study of an Automated Insulin Delivery System in Adult Participants With Type 1 Diabetes Mellitus		Protocol/abstract/letter/comment/editorial
Eli Lilly Company	2019	A Study of an Automated Insulin Delivery System in Adult Participants With Type 1 Diabetes		Protocol/abstract/letter/comment/editorial
University of Cambridge	2019	Closing the Loop in Adults With Type 1 Diabetes Under Free Living Conditions		Protocol/abstract/letter/comment/editorial
University of Virginia	2021	Hybrid Closed-Loop Control With Prandial Insulin Dosing Informed by Insulin Sensitivity in Adolescents With Type 1 Diabetes		Protocol/abstract/letter/comment/editorial
University of Virginia	2021	Diabetes Closed-Loop Project 6 (DCLP6): Fully Automated Closed-Loop Control in Type 1 Diabetes Using Meal Anticipation		Protocol/abstract/letter/comment/editorial
Sansum Diabetes Research Institute	2020	Automated Insulin Delivery in Pregnant Patients With Type 1 Diabetes With Extension Into Outpatient at Home		Protocol/abstract/letter/comment/editorial
Hospital Clinic of Barcelona	2021	Artificial Pancreas With Carbohydrate Suggestion for Patients With Type 1 Diabetes Prone to Hypoglycemia		Protocol/abstract/letter/comment/editorial
Iacobucci, G.	2023	Type 1 diabetes: NICE recommends new "artificial pancreas" technology	10.1136/bmj.p55	Protocol/abstract/letter/comment/editorial
Chen, N. S.	2021	User Engagement With the CamAPS FX Hybrid Closed-Loop App According to Age and User Characteristics	10.2337/dc20-2762	Protocol/abstract/letter/comment/editorial
Choudhary, P.	2016	Implications of Predictive Low-Glucose Management System in Hybrid of Full Closed Loop System	10.1089/dia.2016.0227	Protocol/abstract/letter/comment/editorial
Jacobs, P. G.	2022	Improving HbA(1c) levels with advanced hybrid closed-loop therapy	10.1016/S2213-8587(22)00245-5	Protocol/abstract/letter/comment/editorial

N. N.	2022	Closed-Loop System Improves Glycemic Control but Not Hypoglycemia for Young Children With Type 1 Diabetes Mellitus		Protocol/abstract/letter/comment/editorial
Holdstock, V.	2023	Hybrid closed-loop system improves glycaemic control in young people with type 1 diabetes compared with conventional management	10.1136/archdischild-2021-323658	Protocol/abstract/letter/comment/editorial
Delageniere, J.	2021	Prospective analysis of satisfaction and usability of closed-loop Diabeloop DBLHU treatment in patients with highly unstable type 1 diabetes	10.1007/s00592-021-01769-9	Protocol/abstract/letter/comment/editorial
Slomski, A.	2022	Large Treatment Effects With Automated Insulin Delivery System	10.1001/jama.2022.15487	Protocol/abstract/letter/comment/editorial
Vambergue, A.	2022	Management of Pregnancy in a Patient with Type 1 Diabetes and Hypoglycemia Unawareness by DBL-hu Closed-Loop Insulin Delivery System	10.1089/dia.2022.0043	Protocol/abstract/letter/comment/editorial
Tysoe, O.	2022	Cognitive function in T1DM improved by hybrid closed-loop insulin delivery	10.1038/s41574-022-00752-w	Protocol/abstract/letter/comment/editorial
Han, J. J.	2021	Pivotal trial of the Omnipod 5 Automated Insulin Delivery System shows promising results	10.1111/aor.14036	Protocol/abstract/letter/comment/editorial
Takita, M.	2020	Multicenter Trial of Closed-Loop Control in Type 1 Diabetes	10.1056/NEJMc1915995	Protocol/abstract/letter/comment/editorial
Beunen, K.	2023	Closed-loop insulin delivery in pregnant women with type 1 diabetes (CRISTAL): a multicentre randomized controlled trial - study protocol	10.1186/s12884-023-05481-0	Protocol/abstract/letter/comment/editorial
Zuniga-Hernandez, J. A.	2020	Multicenter Trial of Closed-Loop Control in Type 1 Diabetes	10.1056/NEJMc1915995	Protocol/abstract/letter/comment/editorial
Larkin, H. D.	2022	Bionic Pancreas Outperforms Standard Care for Type 1 Diabetes in Trial	10.1001/jama.2022.18449	Protocol/abstract/letter/comment/editorial
Kovatchev, B.	2021	ATTD 2021 Invited Speaker Abstracts	10.1089/dia.2021.2525.abstracts	Protocol/abstract/letter/comment/editorial
Wu, Z.	2022	ICIMH 2022 Abstracts	10.1177/2164957x221096590	Protocol/abstract/letter/comment/editorial
Actrn	2020	Optimising self-management in youth with type 1 diabetes to improve short-term glycaemic control: OPTIMISE Study		Protocol/abstract/letter/comment/editorial
Nct	2019	The Impact of a Predictive Hypoglycaemia Alert Function in Physical Activity for People With T1DM		Protocol/abstract/letter/comment/editorial
Nct	2020	Maximising Time With a Normal Blood Glucose to Restore the Glucagon Response in Type 1 Diabetes		Protocol/abstract/letter/comment/editorial
Nct	2023	Use of Control-IQ Technology 2.0 in Adults, Children, and Preschoolers With Type 1 Diabetes		Protocol/abstract/letter/comment/editorial
Burnside, M. J.	2022	58(th) EASD Annual Meeting of the European Association for the Study of Diabetes : Stockholm, Sweden, 19 - 23 September 2022	10.1007/s00125-022-05755-w	Protocol/abstract/letter/comment/editorial
Burnside, M.	2020	CREATE (Community deRivEd AutomaTEd insulin delivery) trial. Randomised parallel arm open label clinical trial comparing automated	10.1007/s40200-020-00547-8	Protocol/abstract/letter/comment/editorial

		insulin delivery using a mobile controller (AnyDANA-loop) with an open-source algorithm with sensor augmented pump therapy in type 1 diabetes		
Actrn	2020	Assessing efficacy and safety of Automated Insulin Delivery utilizing open source technology in children and adults with Type 1 Diabetes		Protocol/abstract/letter/comment/editorial
Nct	2022	Feasibility of Automated Insulin Delivery With an Interoperable Algorithm Using an Alternative Insulin Pump		Protocol/abstract/letter/comment/editorial
Nct	2021	MANATEE-T1D: metformin ANd AutomaTEd Insulin Delivery System Effects on Renal Vascular Resistance, Insulin Sensitivity, and Cardiometabolic Function in Youth With Type 1 Diabetes		Protocol/abstract/letter/comment/editorial
Nct	2019	Artificial Pancreas With Different Stress Assessments in the Outpatient Setting		Protocol/abstract/letter/comment/editorial
Nct	2020	Artificial Pancreas - Adolescent Physiology & Psychology Longitudinal Evaluation		Protocol/abstract/letter/comment/editorial
Actrn	2022	Randomised controlled trial investigating the safety and efficacy of an open source automated insulin delivery system without manual mealtime boluses, in people with type 1 diabetes		Protocol/abstract/letter/comment/editorial
Nct	2022	Artificial Pancreas Technology to Reduce Glycemic Variability and Improve Cardiovascular Health in Type 1 Diabetes		Protocol/abstract/letter/comment/editorial
Shin, J.	2018	The Official Journal of ATTD Advanced Technologies & Treatments for Diabetes Conference Austria, Vienna-February 14-17, 2018	10.1089/dia.2018.2525.abstracts	Protocol/abstract/letter/comment/editorial
Nct	2023	Efficacy and Safety of Android Artificial Pancreas System in Adult Patients With Type 1 Diabetes Mellitus in China		Protocol/abstract/letter/comment/editorial
Choudhary, P.	2022	The Official Journal of ATTD Advanced Technologies & Treatments for Diabetes Conference 27-30 April 2022 I Barcelona & Online	10.1089/dia.2022.2527.abstracts	Protocol/abstract/letter/comment/editorial
Nct	2016	Multi-center Trial in Adult and Pediatric Patients With Type 1 Diabetes Using Hybrid Closed Loop System at Home		Protocol/abstract/letter/comment/editorial
Brown, S. A.	2020	101-LB: Eighteen-Month Use of Closed-Loop Control (CLC): A Randomized, Controlled Trial	10.2337/db20-101-LB	Protocol/abstract/letter/comment/editorial
Nct	2020	ADvanced Hybrid Closed Loop Study in Adult Population With Type 1 Diabetes		Protocol/abstract/letter/comment/editorial
Nct	2021	Simple Initiation of Advanced Hybrid Closed Loop System		Protocol/abstract/letter/comment/editorial
Paldus, B.	2021	First Randomized Controlled Trial of Hybrid Closed Loop Versus Multiple Daily Injections or Insulin Pump Using Self-Monitoring of Blood Glucose in Free-Living Adults with Type 1 Diabetes Undertaking Exercise	10.1177/19322968211035110	Protocol/abstract/letter/comment/editorial
Von Dem Berge, T.	2021	ATTD 2021 Invited Speaker Abstracts	10.1089/dia.2021.2525.abstracts	Protocol/abstract/letter/comment/editorial
Nct	2020	The International Diabetes Closed Loop (iDCL) Trial: protocol 4		Protocol/abstract/letter/comment/editorial

Fernandes, N.	2020	A Randomized Trial of Closed-Loop Control in Children with Type 1 Diabetes	10.1056/NEJMc2030417	Protocol/abstract/letter/comment/editorial
Hooper, J.	2022	ICIMH 2022 Abstracts	10.1177/2164957x221096590	Protocol/abstract/letter/comment/editorial
Murata, Y.	2023	Randomized Trial of a Bionic Pancreas in Type 1 Diabetes	10.1056/NEJMc2213988	Protocol/abstract/letter/comment/editorial
Khoo, T. K.	2023	Randomized Trial of a Bionic Pancreas in Type 1 Diabetes	10.1056/NEJMc2213988	Protocol/abstract/letter/comment/editorial
McAuley, S. A.	2021	212-OR: Closed-Loop Increases Time-in-Range in Older Adults with Type 1 Diabetes Compared with Sensor-Augmented Pump Therapy: A Randomized Crossover Trial	10.2337/db21-212-OR	Protocol/abstract/letter/comment/editorial
Matejko, B.	2023	ATTD 2023 Abstract Author Index	10.1089/dia.2023.2526.abstracts.index	Protocol/abstract/letter/comment/editorial
Cyranka, K.	2023	ATTD 2023 Abstract Author Index	10.1089/dia.2023.2526.abstracts.index	Protocol/abstract/letter/comment/editorial
Cyranka, K.	2022	ICIMH 2022 Abstracts	10.1177/2164957x221096590	Protocol/abstract/letter/comment/editorial
Matejko, B.	2022	ICIMH 2022 Abstracts	10.1177/2164957x221096590	Protocol/abstract/letter/comment/editorial
Schoelwer, M. J.	2021	Predictors of Time-in-Range (70-180 mg/dl) Achieved Using a Closed-Loop Control System	10.1089/dia.2020.0646	Protocol/abstract/letter/comment/editorial
Adenis, A.	2021	57(th) EASD Annual Meeting of the European Association for the Study of Diabetes	10.1007/s00125-021-05519-y	Protocol/abstract/letter/comment/editorial
Boughton, C. K.	2022	288-OR: Effect of 24 Months of Optimised Glucose Control on Residual C-Peptide Secretion in Youth with New Onset Type 1 Diabetes (T1D)	10.2337/db22-288-OR	Protocol/abstract/letter/comment/editorial
Nct	2022	Evaluation of the MiniMed 780 System in Paediatric Subjects		Protocol/abstract/letter/comment/editorial
Nct	2020	AHCL System Initiation in T1D Patients naïve to Technology		Protocol/abstract/letter/comment/editorial
Actrn	2021	Postprandial Glucose Excursions with Difficult Foods in Children with Type 1 Diabetes on Hybrid Closed Loop Therapy: a Pilot Study		Protocol/abstract/letter/comment/editorial
McAuley, S. A.	2020	999-P: Six Months At-Home Hybrid Closed-Loop vs. Manual Insulin Delivery with Finger-Stick Blood Glucose Monitoring in Adults with Type 1 Diabetes: A Randomized Controlled Trial	10.2337/db20-999-P	Protocol/abstract/letter/comment/editorial
Nct	2018	Pregnancy Intervention With a Closed-Loop System (PICLS) Study		Protocol/abstract/letter/comment/editorial
Nct	2018	Efficacy of Closed-loop Insulin Therapy in Prepubertal Child in Free-life		Protocol/abstract/letter/comment/editorial

Nct	2021	Closing the Loop in Adults With Type 1 Diabetes (CLEAR)		Protocol/abstract/letter/comment/editorial
Nct	2022	Closing the Loop in People With Type 1 Diabetes		Protocol/abstract/letter/comment/editorial
Bisio, A.	2020	The Official Journal of ATTD Advanced Technologies & Treatments for Diabetes Conference Madrid, Spain-February 19-22, 2020	10.1089/dia.2020.2525.abstracts	Protocol/abstract/letter/comment/editorial
Van Den Heuvel, T.	2023	ATTD 2023 Abstract Author Index	10.1089/dia.2023.2526.abstracts.index	Protocol/abstract/letter/comment/editorial
Jendle, J.	2023	ATTD 2023 Abstract Author Index	10.1089/dia.2023.2526.abstracts.index	Protocol/abstract/letter/comment/editorial
Jancev, M.	2023	ATTD 2023 Abstract Author Index	10.1089/dia.2023.2526.abstracts.index	Protocol/abstract/letter/comment/editorial
Nct	2020	Closed-loop Insulin Delivery in Pregnant Women With Type 1 Diabetes		Protocol/abstract/letter/comment/editorial
Nct	2019	A Trial Evaluating Automated Insulin Delivery Technologies on Hypoglycemia and Quality of Life in Older Adults With Type 1 Diabetes		Protocol/abstract/letter/comment/editorial
Schierloh, U.	2021	Abstracts for the 47th Annual Conference of the International Society for Pediatric and Adolescent Diabetes (ISPAD), Virtual, October 13-15, 2021	10.1111/pedi.13268	Protocol/abstract/letter/comment/editorial
Kariyawasam, D.	2021	Abstracts	10.1159/000518849	Protocol/abstract/letter/comment/editorial
Allen, J. M.	2022	Poster Abstracts	10.1111/pedi.13400	Protocol/abstract/letter/comment/editorial
Actrn	2019	The Use of a Hybrid Closed Loop System in the Management of Individuals with Type 1 Diabetes and Poor Glycaemic Control Aged 12-25		Protocol/abstract/letter/comment/editorial
Mauras, N.	2022	101-LB: Utility and Safety of Backup Insulin Regimens Generated by the Bionic Pancreas—A Randomized Study	10.2337/db22-101-LB	Protocol/abstract/letter/comment/editorial
Nct	2021	Diabeloop For Teens		Protocol/abstract/letter/comment/editorial
Collyns, O.	2020	979-P: Improved Technology Satisfaction and Sleep Quality with Medtronic Minimed Advanced Hybrid Closed-Loop Delivery Compared with Predictive Low Glucose Suspend in People with Type 1 Diabetes in a Randomized Crossover Trial	10.2337/db20-979-P	Protocol/abstract/letter/comment/editorial
Tubiana-Rufi, N.	2019	Oral Sessions	10.1111/pedi.12923	Protocol/abstract/letter/comment/editorial
MacLeish, S. A.	2021	Poster Abstract	10.1111/pedi.13269	Protocol/abstract/letter/comment/editorial
Kimbell, B.	2021	Poster Abstract	10.1111/pedi.13269	Protocol/abstract/letter/comment/editorial

Burckhardt, M.-A.	2019	282-OR: Impact of a Hybrid Closed-Loop Insulin Delivery System on Hypoglycemia Awareness in Individuals with Type 1 Diabetes	10.2337/db19-282-OR	Protocol/abstract/letter/comment/editorial
Isganaitis, E.	2019	Late Breaker - Oral Abstracts	10.1111/pedi.12925	Protocol/abstract/letter/comment/editorial
Fuchs, J.	2021	Assessing the efficacy, safety and utility of closed-loop insulin delivery compared with sensor-augmented pump therapy in very young children with type 1 diabetes (KidsAP02 study): an open-label, multicentre, multinational, randomised cross-over study protocol	10.1136/bmjopen-2020-042790	Protocol/abstract/letter/comment/editorial
Madrid-Valero, J.	2022	ICIMH 2022 Abstracts	10.1177/2164957x221096590	Protocol/abstract/letter/comment/editorial
Petrovski, G.	2022	ICIMH 2022 Abstracts	10.1177/2164957x221096590	Protocol/abstract/letter/comment/editorial
Baagar, K.	2022	ICIMH 2022 Abstracts	10.1177/2164957x221096590	Protocol/abstract/letter/comment/editorial
Garg, S.	2022	ICIMH 2022 Abstracts	10.1177/2164957x221096590	Protocol/abstract/letter/comment/editorial
Franc, S.	2022	ICIMH 2022 Abstracts	10.1177/2164957x221096590	Protocol/abstract/letter/comment/editorial
Nct	2023	Clinical Trial to Evaluate the Efficacy of the Smart Insulin Pen Compared to a Closed Loop System in Patients With Type 1 Diabetes (EBIACE-1)		Protocol/abstract/letter/comment/editorial
Ekhlaspour, L.	2023	ATTD 2023 Abstract Author Index	10.1089/dia.2023.2526.abstracts.index	Protocol/abstract/letter/comment/editorial
Crocket, H.	2023	ATTD 2023 Abstract Author Index	10.1089/dia.2023.2526.abstracts.index	Protocol/abstract/letter/comment/editorial
Forlenza, G.	2023	ATTD 2023 Abstract Author Index	10.1089/dia.2023.2526.abstracts.index	Protocol/abstract/letter/comment/editorial
Ware, J.	2023	ATTD 2023 Abstract Author Index	10.1089/dia.2023.2526.abstracts.index	Protocol/abstract/letter/comment/editorial
Franc, S.	2023	ATTD 2023 Abstract Author Index	10.1089/dia.2023.2526.abstracts.index	Protocol/abstract/letter/comment/editorial
Nct	2019	Safety Assessment of DBLUS System in Adolescent and Adult Patients With Type 1 Diabetes and Assessment of Its Clinical Efficacy (DIABELOOP SP8)		Protocol/abstract/letter/comment/editorial
Renard, E.	2021	ATTD 2021 Invited Speaker Abstracts	10.1089/dia.2021.2525.abstracts	Protocol/abstract/letter/comment/editorial
Actrn	2022	Hybrid Closed Loop in Advanced Renal Disease		Protocol/abstract/letter/comment/editorial
Nct	2021	Automated Insulin Delivery Amongst Pregnant Women With Type 1 Diabetes		Protocol/abstract/letter/comment/editorial

Nct	2019	Diabeloop for Highly Unstable Type 1 Diabetes		Protocol/abstract/letter/comment/editorial
Nct	2021	Closed-loop Insulin Delivery In Type 1 Diabetes Pregnancies (CIRCUIT) - Internal Pilot		Protocol/abstract/letter/comment/editorial
Actrn	2022	Assessment of closed loop technology in young children with Type 1 Diabetes Aged 2-7		Protocol/abstract/letter/comment/editorial
Nct	2020	An Open-label, Two-center, Randomized, Cross-over Study to Evaluate the Safety and Efficacy of Glycemic Control Using Hybrid-closed Loop vs. Advanced Hybrid Closed-loop in Young Subjects With Type 1 Diabetes		Protocol/abstract/letter/comment/editorial
Fuchs, J.	2021	214-OR: Cambridge Hybrid Closed-Loop in Children and Adolescents with T1D: A Multicentre Six-Month Randomised Trial	10.2337/db21-214-OR	Protocol/abstract/letter/comment/editorial
de Portu, S.	2022	Randomised controlled trial of Advanced Hybrid Closed Loop in an Adult Population with Type 1 Diabetes (ADAPT): study protocol and rationale	10.1136/bmjopen-2021-050635	Protocol/abstract/letter/comment/editorial
O'Neal, D. N.	2020	56(th) EASD Annual Meeting of the European Association for the Study of Diabetes : 21-25 September 2020	10.1007/s00125-020-05221-5	Protocol/abstract/letter/comment/editorial
Breton, M. D.	2020	56(th) EASD Annual Meeting of the European Association for the Study of Diabetes : 21-25 September 2020	10.1007/s00125-020-05221-5	Protocol/abstract/letter/comment/editorial
Isganaitis, E.	2020	2019 Diabetes Technology Meeting Abstracts	10.1177/1932296819897652	Protocol/abstract/letter/comment/editorial
Kudva, Y. C.	2020	2019 Diabetes Technology Meeting Abstracts	10.1177/1932296819897652	Protocol/abstract/letter/comment/editorial
Ekhlaspour, L.	2020	2019 Diabetes Technology Meeting Abstracts	10.1177/1932296819897652	Protocol/abstract/letter/comment/editorial
Portillo, K.	2020	2019 Diabetes Technology Meeting Abstracts	10.1177/1932296819897652	Protocol/abstract/letter/comment/editorial
Nct	2018	The Artificial Pancreas in Very Young Children With T1D		Protocol/abstract/letter/comment/editorial
Isrctn	2016	Closed-loop in pregnancy day and night home feasibility study (CLIP 24/7)		Protocol/abstract/letter/comment/editorial
Pasqua, M. R.	2023	ATTD 2023 Abstract Author Index	10.1089/dia.2023.2526.abstracts.index	Protocol/abstract/letter/comment/editorial
Ware, J.	2022	58(th) EASD Annual Meeting of the European Association for the Study of Diabetes : Stockholm, Sweden, 19 - 23 September 2022	10.1007/s00125-022-05755-w	Protocol/abstract/letter/comment/editorial
Dat, D. Q.	2022	58(th) EASD Annual Meeting of the European Association for the Study of Diabetes : Stockholm, Sweden, 19 - 23 September 2022	10.1007/s00125-022-05755-w	Protocol/abstract/letter/comment/editorial
Forlenza, G. P.	2022	58(th) EASD Annual Meeting of the European Association for the Study of Diabetes : Stockholm, Sweden, 19 - 23 September 2022	10.1007/s00125-022-05755-w	Protocol/abstract/letter/comment/editorial
Ekhlaspour, L.	2020	387-P: Glycemic Outcomes in Baseline Hemoglobin A1c Subgroups in the International Diabetes Closed-Loop (IDCL) Trial	10.2337/db20-387-P	Protocol/abstract/letter/comment/editorial

Nct	2023	Dual Hormone Closed Loop in Type 1 Diabetes		Protocol/abstract/letter/comment/editorial
Musolino, G.	2019	Assessing the efficacy, safety and utility of 6-month day-and-night automated closed-loop insulin delivery under free-living conditions compared with insulin pump therapy in children and adolescents with type 1 diabetes: an open-label, multicentre, multinational, single-period, randomised, parallel group study protocol	10.1136/bmjopen-2018-027856	Protocol/abstract/letter/comment/editorial
Actrn	2022	The Co-Pilot trial: closed loop in children and youth with type 1 diabetes and high-risk glycaemic control		Protocol/abstract/letter/comment/editorial
Tauschmann, M.	2016	Poster Tours	10.1111/pedi.12451	Protocol/abstract/letter/comment/editorial
Burnside, M.	2020	The Official Journal of ATTD Advanced Technologies & Treatments for Diabetes Conference Madrid, Spain-February 19-22, 2020	10.1089/dia.2020.2525.abstracts	Protocol/abstract/letter/comment/editorial
Actrn	2019	Older Adult Closed Loop (ORACL) Study for Type 1 Diabetes		Protocol/abstract/letter/comment/editorial
Nct	2018	The International Diabetes Closed Loop (iDCL) Trial: clinical Acceptance of the Artificial Pancreas		Protocol/abstract/letter/comment/editorial
Tauschmann, M.	2018	Oral Sessions	10.1111/pedi.12745	Protocol/abstract/letter/comment/editorial
Nct	2017	The Artificial Pancreas in Very Young Children With T1D - Pilot (KidsAP01)		Protocol/abstract/letter/comment/editorial
Kovatchev, B.	2019	Abstracts	10.1159/000501868	Protocol/abstract/letter/comment/editorial
Sherr, J.	2021	ATTD 2021 Invited Speaker Abstracts	10.1089/dia.2021.2525.abstracts	Protocol/abstract/letter/comment/editorial
Nct	2019	24/7 Closed-loop in Older Subjects With Type 1 Diabetes		Protocol/abstract/letter/comment/editorial
Isrctn	2018	Automated insulin delivery among pregnant women with type 1 diabetes		Protocol/abstract/letter/comment/editorial
Boughton, C. K.	2021	57(th) EASD Annual Meeting of the European Association for the Study of Diabetes	10.1007/s00125-021-05519-y	Protocol/abstract/letter/comment/editorial
Actrn	2019	Randomized cross over trial comparing advanced hybrid closed loop mode with sensor augmented pump therapy in type 1 diabetes		Protocol/abstract/letter/comment/editorial
Collyns, O.	2020	199-OR: Improved Glycemic Outcomes with Medtronic Minimed Advanced Hybrid Closed-Loop Delivery: Results from a Randomized Crossover Trial Comparing Automated Insulin Delivery with Predictive Low Glucose Suspend in People with Type 1 Diabetes	10.2337/db20-199-OR	Protocol/abstract/letter/comment/editorial
McAuley, S. A.	2018	Effect of 6 months of hybrid closed-loop insulin delivery in adults with type 1 diabetes: a randomised controlled trial protocol	10.1136/bmjopen-2017-020274	Protocol/abstract/letter/comment/editorial

Buckingham, B.	2018	The Official Journal of ATTD Advanced Technologies & Treatments for Diabetes Conference Austria, Vienna-February 14-17, 2018	10.1089/dia.2018.2525.abstracts	Protocol/abstract/letter/comment/editorial
Ruan, Y.	2018	The Official Journal of ATTD Advanced Technologies & Treatments for Diabetes Conference Austria, Vienna-February 14-17, 2018	10.1089/dia.2018.2525.abstracts	Protocol/abstract/letter/comment/editorial
Tauschmann, M.	2018	The Official Journal of ATTD Advanced Technologies & Treatments for Diabetes Conference Austria, Vienna-February 14-17, 2018	10.1089/dia.2018.2525.abstracts	Protocol/abstract/letter/comment/editorial
Carlson, A.	2020	56(th) EASD Annual Meeting of the European Association for the Study of Diabetes : 21-25 September 2020	10.1007/s00125-020-05221-5	Protocol/abstract/letter/comment/editorial
Renard, E.	2020	56(th) EASD Annual Meeting of the European Association for the Study of Diabetes : 21-25 September 2020	10.1007/s00125-020-05221-5	Protocol/abstract/letter/comment/editorial
Franc, S.	2020	The Official Journal of ATTD Advanced Technologies & Treatments for Diabetes Conference Madrid, Spain-February 19-22, 2020	10.1089/dia.2020.2525.abstracts	Protocol/abstract/letter/comment/editorial
Gonder-Frederick, L.	2020	The Official Journal of ATTD Advanced Technologies & Treatments for Diabetes Conference Madrid, Spain-February 19-22, 2020	10.1089/dia.2020.2525.abstracts	Protocol/abstract/letter/comment/editorial
Breton, M.	2020	The Official Journal of ATTD Advanced Technologies & Treatments for Diabetes Conference Madrid, Spain-February 19-22, 2020	10.1089/dia.2020.2525.abstracts	Protocol/abstract/letter/comment/editorial
Tauschmann, M.	2018	54(th) EASD Annual Meeting of the European Association for the Study of Diabetes : Berlin, Germany, 1 - 5 October 2018	10.1007/s00125-018-4693-0	Protocol/abstract/letter/comment/editorial
Franc, S.	2018	Twelve-week home use of hybrid closed-loop insulin delivery system vs. sensor-assisted pump therapy in adults with type 1 diabetes-intermediate results of the multicenter randomised crossover diabeloop WP7 trial		Protocol/abstract/letter/comment/editorial
Anderson, S.	2018	The Official Journal of ATTD Advanced Technologies & Treatments for Diabetes Conference Austria, Vienna-February 14-17, 2018	10.1089/dia.2018.2525.abstracts	Protocol/abstract/letter/comment/editorial
Kovatchev, B.	2018	The Official Journal of ATTD Advanced Technologies & Treatments for Diabetes Conference Austria, Vienna-February 14-17, 2018	10.1089/dia.2018.2525.abstracts	Protocol/abstract/letter/comment/editorial
Brown, S.	2018	The Official Journal of ATTD Advanced Technologies & Treatments for Diabetes Conference Austria, Vienna-February 14-17, 2018	10.1089/dia.2018.2525.abstracts	Protocol/abstract/letter/comment/editorial
Ghatak, A.	2023	Closed-Loop from Diagnosis of Type 1 Diabetes in Children and Young People	10.1089/dia.2023.0217	Protocol/abstract/letter/comment/editorial
Buschur, E.	2023	175-OR: Pregnancy Intervention with a Closed-Loop System (PICLS) Study	10.2337/db23-175-OR	Protocol/abstract/letter/comment/editorial
Biostatem Axonal	2024	Evaluation of Security and Efficacy of Medtrum Hybrid Closed Loop System in Children, Adolescents and Adults With Type 1 Diabetes		Protocol/abstract/letter/comment/editorial
Murphy, H. R.	2023	108-LB: Randomized Trial of Automated Insulin Delivery in Pregnant Women with Type 1 Diabetes	10.2337/db23-108-LB	Protocol/abstract/letter/comment/editorial
King, J.	2024	Glycemic Variability in Pregnant Individuals Using Assisted Hybrid Closed-Loop Therapy Versus Sensor-Augmented Pump Therapy	10.1177/19322968241260050	Protocol/abstract/letter/comment/editorial
Boucsein, A.	2024	Protocol for a prospective, multicenter, parallel-group, open-label randomized controlled trial comparing standard care with Closed lOoP In	10.1007/s40200-024-01397-4	Protocol/abstract/letter/comment/editorial

		chiLdren and yOuth with Type 1 diabetes and high-risk glycemic control: the CO-PILOT trial		
Wilkinson, T.	2024	Study protocol for a randomised open-label clinical trial examining the safety and efficacy of the Android Artificial Pancreas System (AAPS) with advanced bolus-free features in adults with type 1 diabetes: the 'CLOSE IT' (Closed Loop Open SourcE In Type 1 diabetes) trial	10.1136/bmjopen-2023-078171	Protocol/abstract/letter/comment/editorial
Christensen, M. B.	2023	59th EASD Annual Meeting of the European Association for the Study of Diabetes	10.1007/s00125-023-05969-6	Protocol/abstract/letter/comment/editorial
Reiss, A L.	2022	A Pilot randomized trial to examine effects of a hybrid closed-loop insulin delivery system on neurodevelopmental and cognitive outcomes in adolescents with type 1 diabetes	10.1038/s41467-022-32289-x	Missing data could not be obtained

2.2 Included studies

First author, publication year	Trial registration	Country	Design	Intervention duration [weeks]	Main inclusion criteria	N completed study	Intervention (pump, algorithm, sensor)	Control	Funding / support
Abraham 2021 [8]	ACTRN12616000753459	Australia	parallel	26	Age 12-<25y, HbA1c ≤10.5%	111	HCL (MiniMed 670G + Guardian Sensor 3)	MDI (19%), CSII (28%), SAP (24%), PLGM (30%)	commercial and non-commercial
Abraham 2025 [9]	ACTRN12619001452189	Australia	parallel	24	Age 12-<25y, HbA1c >8.5%	38	AHCL (MiniMed 670G 4.0 + Guardian Sensor 3)	CSII (33%), SAP (67%)	commercial and non-commercial
Anderson 2019 [10]	NCT02302963	United States	parallel	4	Age 12-70y, HbA1c <10.0%, risk of hypoglycaemia or hypoglycaemia unawareness	42	HCL (AccuChek Spirit Combo + USS Virginia algorithm + Dexcom G4 Platinum)	SAP	commercial and non-commercial
Bally 2017 [11]	NCT02727231	United Kingdom, Austria	cross-over	4	Age ≥18y, HbA1c <7.5%	29	HCL (DANA-R Diabecare + Florence D2A + FreeStyle Navigator II)	CSII	commercial and non-commercial
Benhalima 2024 [12]	NCT04520971	Belgium, Netherlands	parallel	before 14 weeks' gestation until 33–36 weeks of gestation	Age 18-45y, pregnant women, HbA1c ≤10%	93	AHCL (MiniMed 780G + SmartGuard + Guardian Sensor 4)	MDI + CGM (4 %), SAP (15 %), PLGM (81 %), HCL (4 %)	commercial and non-commercial
Benhamou 2019 [13]	NCT02987556	France	cross-over	12	Age ≥18y, HbA1c ≤10.0%, and preserved hypoglycaemia awareness	63	AHCL (Cellnovo Generation 1 and Kaleido + Diabeloop Generation 1 (DBLG1) + Dexcom G5)	SAP	commercial and non-commercial
Benhamou 2021 [14]	NCT04042207	France	cross-over	8	Age ≥22y, severe glucose instability	5	AHCL (Kaleido + adapted DBLG1 + Dexcom G6)	PLGM	commercial and non-commercial
Bergenstal 2021 [15]	NCT03040414	United States, Germany, Israel, Slovenia	cross-over	12	Age 14-29y, HbA1c 7.0%-11.0%	113	AHCL (MiniMed 670G + investigational algorithm + Guardian Sensor 3)	HCL (MiniMed 670G + Guardian Sensor 3)	commercial and non-commercial
Boucsein 2024 [16]	ACTRN12622001454763	New Zealand	parallel	13	Age 7-25y, HbA1c ≥8.5%	80	AHCL (MiniMed 780G + SmartGuard + Guardian Sensor 4)	MDI (84%) CSII (9%), SAP (7%)	commercial and non-commercial

Boughton 2022a [17]	NCT02871089	United Kingdom	parallel	104	Age 10-<17y, T1D diagnosed within the previous 21 days	85	AHCL (modified MiniMed 640G + CamAPS FX + Guardian Sensor 3 or Dana Diabecare RS + CamAPS FX + Dexcom G6)	MDI (at beginning, later change allowed)	commercial and non-commercial
Boughton 2022b [18]	NCT04025762	United Kingdom, Austria	cross-over	16	Age ≥60y, HbA1c ≤10.0%	36	AHCL (Dana Diabecare RS + CamAPS FX + Dexcom G6)	SAP	commercial and non-commercial
Boughton 2023 [19]	NCT04977908	United Kingdom	cross-over	8	Age ≥18y, HbA1c ≥8.0%	26	FCL (Dana Diabecare RS + CamAPS HX + Dexcom G6)	SAP	commercial and non-commercial
Breton 2020 [20]	NCT03844789	United States	parallel	16	Age 6-13y	101	AHCL (t:slim X2 + Control-IQ + Dexcom G6)	SAP (80%), PLGM	commercial and non-commercial
Brown 2019 [21]	NCT03563313	United States	parallel	26	Age ≥14y, without a restriction on HbA1c	168	AHCL (t:slim X2 + Control-IQ + Dexcom G6)	SAP	commercial and non-commercial
Brown 2020 [22]	NCT03591354	United States	parallel	13	participants in the AID-group in the preceding study (Brown 2019)	109	AHCL (t:slim X2 + Control-IQ + Dexcom G6)	PLGM	commercial and non-commercial
Burckhardt 2021 [23]	ACTRN12616000909426	Australia	cross-over	8	Age >12-55y, hypoglycaemia unawareness (Gold score ≥4)	17	HCL (MiniMed 670G + Guardian Sensor 3)	CSII (66%), SAP (n=2), PLGM (n=3)	commercial and non-commercial
Burnside 2022 <16 [24]	ACTRN12620000034932	New Zealand	parallel	24	Age 7-<16y, HbA1c <10.5%	47	HCL (preproduction DANA-i + modified AndroidAPS 2.8 + Dexcom G6)	SAP	commercial and non-commercial
Burnside 2022 ≥16 [24]	ACTRN12620000034932	New Zealand	parallel	24	Age 16-70y, HbA1c <10.5%	48	HCL (preproduction DANA-i + modified AndroidAPS 2.8 + Dexcom G6)	SAP	commercial and non-commercial
Choudhary 2022 [25]	NCT04235504	France, Germany, United Kingdom	parallel	24	Age ≥18y, HbA1c ≥8.0%	82	AHCL (MiniMed 670G 4.0 similar to MiniMed 780G + Guardian Sensor 3)	MDI	commercial
Christensen 2024 [26]	NCT04914910	Denmark	parallel	14	Age 18-75y, HbA1c ≥7.5%	40	AHCL (MiniMed 780G + SmartGuard +	SAP	commercial and non-commercial

							Guardian Sensor 3 link / Guardian Sensor 4)		
Collyns 2021 [27]	ANZCTR12619000007134 and NCT04073576	New Zealand	cross-over	4	Age 7-80y, HbA1c ≤10.0%	59	AHCL (MiniMed 670G 4.0 + Guardian Sensor 3)	PLGM	commercial
Donovan 2023 [28]	NCT04420728	Canada	parallel	12	Age 18-45y, pregnant women, HbA1c <9.9%	18	HCL (MiniMed 670G or 770G + Guardian Sensor 3)	SAP, PLGM (n=1)	commercial and non-commercial
Garg 2023 [29]	NCT02748018	United States, Canada	parallel	26	Age 2-80y, HbA1c <10.0%	278	HCL (MiniMed 670G + Guardian Sensor 3)	CSII	commercial
Kariyawasam 2022 [30]	NCT03671915	France, Belgium	cross-over	6	Age 6-12y, Tanner stage I, HbA1c ≤9.0%	17	AHCL (Kaleido + adapted DBLG1 + Dexcom G6)	SAP	commercial
Kim 2024 [31]	KCT0008398	South Korea	parallel	12	Age 19–69y, HbA1c <10.0%	104	HCL (EOPatch X +TypeZero inControl + Dexcom G6)	SAP	non-commercial
Kovatchev 2020 [32]	NCT02985866	United States	parallel	13	Age ≥14y, HbA1c <10.5%	125	HCL (AccuChek Spirit Combo + inControlAP + Dexcom G4 or G5)	SAP	commercial and non-commercial
Kudva 2025 [33]	NCT04016662	United States	cross-over	12	Age ≥65y	81	AHCL (t:slim X2 + Control-IQ + Dexcom G6)	SAP<br>PLGM	commercial and non-commercial
Lee 2023 [34]	ISRCTN56898625	United Kingdom	parallel	from 16 weeks' gestation until delivery	Age 18-45y, pregnant women, HbA1c <10.0%	120	AHCL (Dana Diabecare RS + CamAPS FX + Dexcom G6)	MDI (59%), CSII	commercial and non-commercial
Matejko 2022 [35]	NCT04616391	Poland	parallel	12	Age 26-60y, HbA1c <10.0%	37	AHCL (MiniMed 780G + advanced HCL algorithm + Guardian Sensor 3)	MDI	commercial
McAuley 2020 [36]	ACTRN12617000520336	Australia	parallel	26	Age 25-75y; HbA1c ≤10.5%	110	HCL (MiniMed 670G + Enlite 3)	MDI (51%), CSII	commercial and non-commercial
McAuley 2022 [37]	ACTRN126190000515190	Australia	cross-over	16	Age ≥60y, HbA1c ≤10.5%	30	HCL (MiniMed 670G + Guardian Sensor 3)	SAP	commercial and non-commercial
McVean 2023 [38]	NCT04233034	United States	parallel	52	Age 7-17y, T1D diagnosed within 31 days of randomization	108	AHCL t:slim X2 + Control-IQ + Dexcom G6 or MiniMed 670G 4.0 + Guardian Sensor 3 or	CSII (56%), MDI	commercial and non-commercial

							MiniMed 780G + Guardian Sensor 4		
Nanayakkara 2023 [39]	ACTRN12620001191987	Australia	cross-over	4	Adults, HbA1c <10.0%	20	HCL (YpsoPump OPN + AAPS source code 2.5 + Dexcom G5)	CSII	commercial
Pinsker 2022 [40]	NCT04436796	United States	cross-over	13	Age ≥18y	35	HCL (Tandem t:AP + Zone-MPC + Dexcom G6)	SAP (89%), PLGM	commercial and non-commercial
Polsky 2024 [41]	NCT03774186	United States	parallel	from 14-18 weeks gestation until 4-6 weeks postpartum	Age 18-45y, pregnant women, HbA1c 5.5%-9%	23	HCL (MiniMed 670G + Guardian Sensor 3)	SAP	commercial and non-commercial
Renard 2023 [42]	NCT04266379	France	parallel	12	Age >18y, HbA1c <10.5%, Clarke score >3, and/or experience of severe hypoglycaemia during the previous 6 months.	71	AHCL (t:slim X2 + Control-IQ + Dexcom G6)	SAP	commercial and non-commercial
Renard 2024 [43]	NCT05409131	United States, France	parallel	13	Age 18-70y, HbA1c 7.0-11.0% (≥80% of participants ≥8.0%), ≥50% were using an Omnipod pump	193	AHCL (Omnipod 5 + Dexcom G6)	SAP	commercial
Russell 2022 [44]	NCT04200313	United States	parallel	13	Age ≥6y, without a restriction on HbA1c	326	AHCL (iLet + bionic-pancreas insulin-dosing algorithm + Dexcom G6)	MDI (36%), CSII (4%), SAP (25%), PLGM (5%), HCL (30%)	commercial and non-commercial
Stewart 2018 [45]	IRCTN83316328	United Kingdom	cross-over	4	Age 18-45y, pregnant women, HbA1c 6.5%-10.0%	16	HCL (Dana Diabecare R + Florence D2A + FreeStyle Navigator II)	SAP	commercial and non-commercial
Tauschmann 2016 [46]	NCT01873066	United Kingdom, United States	cross-over	3	Age 10-18y, HbA1c ≤11.0%	12	HCL (Dana Diabecare R + Florence D2A + FreeStyle Navigator II)	SAP	commercial and non-commercial
Tauschmann 2018 [47]	NCT02523131	United Kingdom	parallel	12	Age ≥6y, HbA1c 7.5%-10.0%	86	HCL (modified MiniMed 640G + MPC version 0.3.46 + Enlite 3)	SAP	commercial and non-commercial
Thabit 2015 [48]	NCT01961622	United Kingdom,	cross-over	12	Age ≥18y, HbA1c 7.5%-10.0%,	33	HCL (Dana Diabecare R + Florence D2A +	SAP	commercial and non-commercial

		Germany, Austria					FreeStyle Navigator II)		
Von dem Berge 2022 [49]	NCT03815487	Germany	cross-over	8	Age 2-14y, HbA1c <12.0%	37	HCL (MiniMed 670G + Guardian Sensor 3)	PLGM	commercial
Wadwa 2023 [50]	NCT04796779	United States	parallel	13	Age 2-<6y	101	AHCL (t:slim X2 + Control-IQ + Dexcom G6)	SAP (71%), MDI plus CGM	commercial and non-commercial
Ware 2022a [51]	NCT02925299	United Kingdom, United States	parallel	24	Age 6-18y, HbA1c 7.0-10.0%	123	AHCL (modified MiniMed 640G + Florence M + Guardian Sensor 3 or Dana Diabecare RS + CamAPS FX + Dexcom G6)	SAP (65%), CSII	commercial and non-commercial
Ware 2022b [52]	NCT03784027	Austria, Germany, Luxembourg, United Kingdom	cross-over	16	Age 1-7y, HbA1c ≤11.0%	74	AHCL (Dana Diabecare RS + CamAPS FX + Dexcom G6)	SAP	commercial and non-commercial

3 Risk of bias evaluation

Reference	Randomization process	Bias arising from period and carryover effects *	Deviations from intended interventions	Missing outcome data	Measurement of the outcome	Selection of the reported result	Overall Bias
Abraham 2021	Low		Low	Low	Low	Low	Low
Abraham 2025	Low		Low	Low	Low	Low	Low
Anderson 2019	Some concerns		Low	Low	Low	Some concerns	Some concerns
Bally 2017	Low	Low	Low	Low	Low	Low	Low
Benhalima 2024	Low	Low	Low	Low	Low	Low	Low
Benhamou 2019	Low	Low	Low	Low	Low	Low	Low
Benhamou 2021	Some concerns	Low	Low	Low	Low	Low	Some concerns
Bergenstal 2021	Low	Low	Low	Low	Low	Low	Low
Boucsin 2024	Low	Low	Low	Low	Low	Low	Low
Boughton 2022a	Low		Low	Low	Low	Low	Low
Boughton 2022b	Low	Low	Low	Low	Low	Low	Low
Boughton 2023	Some concerns	Low	Low	Low	Low	Low	Some concerns
Breton 2020	Low		Low	Low	Low	Low	Low
Brown 2019	Low		Low	Low	Low	Low	Low
Brown 2020	Low		Low	Low	Low	Low	Low
Burckhardt 2021	Some concerns	Low	Low	Low	Low	Low	Some concerns
Burnside 2022 <16 years	Low		Low	Low	Low	Low	Low
Burnside 2022 ≥16 years	Low		Low	Low	Low	Low	Low
Choudhary 2022	Low		Low	Low	Low	Low	Low
Christensen 2024	Low	Low	Low	Low	Low	Some concerns	Some concerns
Collyns 2021	Some concerns	Low	Low	Low	Low	Low	Some concerns
Donovan 2023	Low		Low	Low	Low	Low	Low
Garg 2023	Some concerns		Low	Low	Low	Low	Some concerns
Kariyawasam 2022	Low	Low	Low	Low	Low	Low	Low
Kim 2024	Low	Low	Low	Low	Low	Some concerns	Some concerns
Kovatchev 2020	Low		Low	Low	Low	Low	Low
Kudva	Low	Low	Low	Low	Low	Low	Low
Lee 2023	Low		Low	Low	Low	Low	Low
Matejko 2022	Low		Some concerns	Low	Low	Low	Some concerns

McAuley 2020	Low		Low	Low	Low	Low	Low
McAuley 2022	Low	Low	Low	Low	Low	Low	Low
McVean 2023	Low		Low	Low	Low	Low	Low
Nanayakkara 2023	Some concerns	Low	Low	Low	Low	Low	Some concerns
Pinsker 2022	Some concerns	Low	Low	Low	Low	Low	Some concerns
Polsky 2024	Some concerns	Low	Low	Low	Low	Low	Some concerns
Renard 2023	Low		Some concerns	Low	Low	Low	Some concerns
Renard 2024	Some concerns		Low	Low	Low	Some concerns	Some concerns
Russel 2022	Low		Low	Low	Low	Low	Low
Stewart 2018	Low	Low	Low	Low	Low	Some concerns	Some concerns
Tauschmann 2016	Some concerns	Low	Low	Low	Low	Low	Some concerns
Tauschmann 2018	Low		Low	Low	Low	Low	Low
Thabit 2015	Low	Low	Low	Low	Low	Low	Low
Von dem Berge 2022	Some concerns	Low	Low	Low	Low	Some concerns	Some concerns
Wadwa 2023	Low		Low	Low	Low	Low	Low
Ware 2022a	Low		Low	Low	Low	Low	Low
Ware 2022b	Low	Low	Low	Low	Low	Low	Low

* assessed for crossover studies

4 Supplement to pairwise meta-analysis

4.1 Summary results of pairwise meta-analyses

The RCTs could be pooled in random effects meta-analyses and summarised to clinically meaningful results, as the studies were reasonably comparable. All direct comparisons that were included in the network meta-analyses are provided in detail in Appendices 4.2 to 4.7 and the CoE assessment is provided in Appendix 8. Compared with SAP therapy, AHCL was associated with a greater TIR (11.4 [9.2; 13.6], low CoE), a lower TAR >180 mg/dl (-9.8 [-12.2; -7.4], low CoE), a lower TAR >250 mg/dl (-6.5 [-8.4; -4.7], low CoE), a lower TBR <70 mg/dl (-1.1 [-1.7; -0.5], low CoE), a lower TBR <54 mg/dl (-0.2 [-0.3; -0.0], very low CoE), and a lower HbA1c (-0.5 [-0.7; -0.3], low CoE). None of the GRADE evaluations of the other comparisons of AID and non-AID systems had a moderate or high CoE; CoE was low in some cases and very low in most cases.

Subgroup sensitivity analyses revealed a lower AID efficacy regarding several outcomes in studies that included only pregnant women (e.g., in terms of TARs and TBRs when HCL and SAP were compared). Partially, AID efficacy was related to HbA1c at baseline, sample size and risk of bias (Appendix 5). The leave-one-out meta-analyses did not identify influential studies. However, the studies comparing AHCL and HCL showed divergent results for TIR and TARs, and studies comparing HCL and PLGM showed divergent results for TBRs (Appendix 6).

4.2 Pairwise meta-analyses TIR

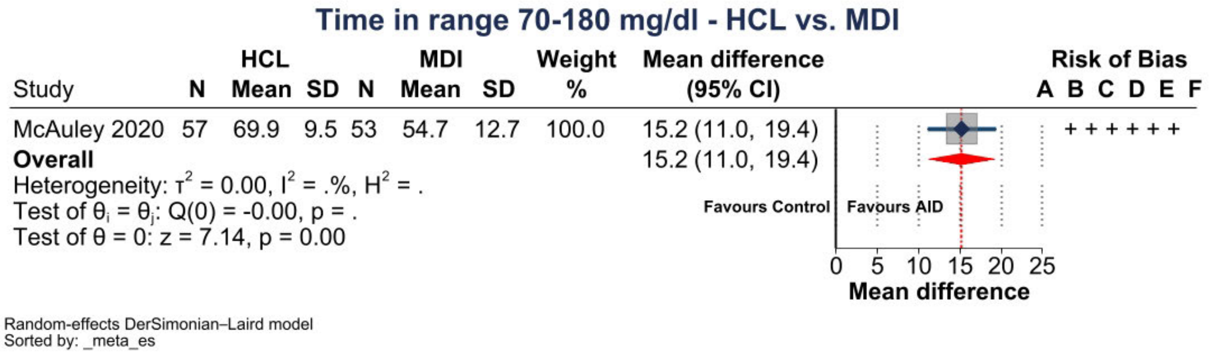

Time in range 70-180 mg/dl - HCL vs. CSII

Study	HCL N	HCL Mean	HCL SD	CSII N	CSII Mean	CSII SD	Weight %	Mean difference (95% CI)	Risk of Bias A B C D E F
Bally 2017	29	76.2	6.4	28	65.6	8.1	35.0	10.6 (6.8, 14.4)	+ + + + + +
Burckhardt 2021	16	69.8	8.7	16	57.9	18.2	5.1	11.9 (2.0, 21.8)	? + + + + ?
Garg 2023	140	67.4	10.8	138	55.4	15.0	53.1	12.0 (8.9, 15.1)	? + + + + ?
Nanayakkara 2023	20	76.6	11.7	20	58.0	15.6	6.8	18.6 (10.1, 27.1)	? + + + + ?
**Overall**								12.0 (9.7, 14.2)	

Heterogeneity: $\tau^2 = 0.00$, $I^2 = 0.00\%$, $H^2 = 1.00$
Test of $\theta_i = \theta_j$: Q(3) = 2.82, p = 0.42
Test of $\theta = 0$: z = 10.48, p = 0.00

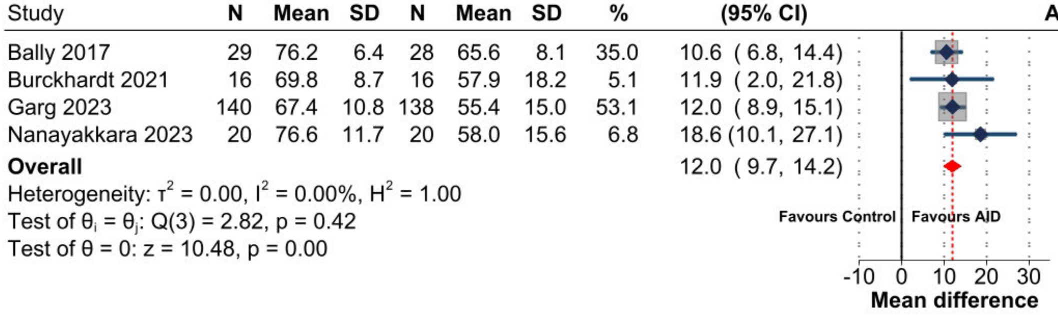

Random-effects DerSimonian–Laird model
Sorted by: _meta_es

Time in range 70-180 mg/dl - HCL vs. SAP

Study	HCL N	HCL Mean	HCL SD	SAP N	SAP Mean	SAP SD	Weight %	Mean difference (95% CI)	Risk of Bias A B C D E F
Polsky 2024	11	75.1	2.4	12	76.5	2.4	10.0	-1.4 (-3.4, 0.6)	? + + + + ?
Donovan 2023	9	79.2	8.7	9	78.2	6.0	8.0	1.0 (-5.9, 7.9)	+ + + + + +
Pinsker 2022	35	69.0	14.0	35	66.0	15.0	8.0	3.0 (-3.8, 9.8)	? + + + + ?
Kim 2024	53	71.5	10.7	51	66.9	15.0	8.9	4.6 (-0.4, 9.6)	+ + + + ? ?
McAuley 2022	30	75.2	6.3	30	69.0	9.1	9.3	6.2 (2.2, 10.2)	+ + + + + +
Kovatchev 2020	64	64.0	11.0	61	57.0	14.0	9.2	7.0 (2.6, 11.4)	+ + + + + +
Thabit 2015	32	67.7	10.6	33	56.8	14.2	8.4	10.9 (4.8, 17.0)	+ + + + + +
Tauschmann 2018	46	65.0	8.0	40	54.0	9.0	9.5	11.0 (7.4, 14.6)	+ + + + + +
Burnside 2022 <16y	20	67.5	11.5	27	52.5	17.5	7.0	15.0 (6.2, 23.8)	+ + + + + +
Burnside 2022 ≥16y	22	74.5	11.9	26	56.5	14.2	7.7	18.0 (10.5, 25.5)	+ + + + + +
Anderson 2019	21	78.2	10.0	21	59.6	16.5	7.3	18.6 (10.3, 26.9)	? + + + ? ?
Tauschmann 2016	12	66.6	7.9	12	47.7	14.4	6.8	18.9 (9.6, 28.2)	? + + + + ?
**Overall**								8.8 (4.7, 13.0)	

Heterogeneity: $\tau^2 = 44.18$, $I^2 = 88.16\%$, $H^2 = 8.45$
Test of $\theta_i = \theta_j$: Q(11) = 92.94, p = 0.00
Test of $\theta = 0$: z = 4.16, p = 0.00

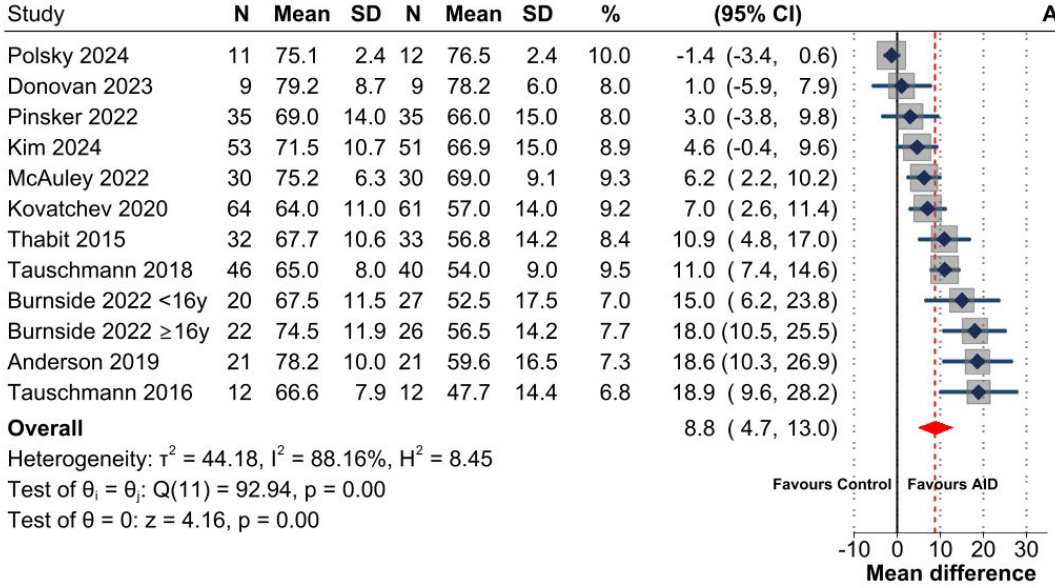

Random-effects DerSimonian–Laird model
Sorted by: _meta_es

Time in range 70-180 mg/dl - HCL vs. PLGM

Study	HCL N	HCL Mean	HCL SD	PLGM N	PLGM Mean	PLGM SD	Weight %	Mean difference (95% CI)	Risk of Bias A B C D E F
Abraham 2021	58	62.5	12.0	63	56.1	12.2	55.3	6.4 (2.1, 10.7)	+ + + + + +
von dem Berge 2022	37	70.8	7.2	37	60.3	13.9	44.7	10.5 (5.5, 15.5)	? + + + ? ?
**Overall**								8.2 (4.2, 12.2)	

Heterogeneity: $\tau^2 = 2.67$, $I^2 = 31.74\%$, $H^2 = 1.46$
Test of $\theta_i = \theta_j$: Q(1) = 1.46, p = 0.23
Test of $\theta = 0$: z = 4.04, p = 0.00

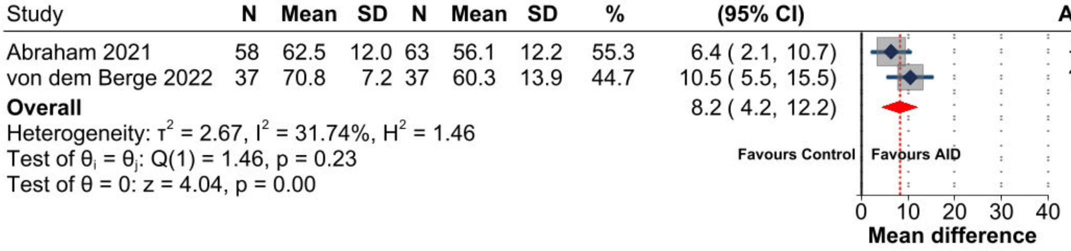

Random-effects DerSimonian–Laird model
Sorted by: _meta_es

Time in range 70-180 mg/dl - AHCL vs. MDI

Study	AHCL N	AHCL Mean	AHCL SD	MDI N	MDI Mean	MDI SD	Weight %	Mean difference (95% CI)	Risk of Bias A B C D E F
Boughton 2022a	42	64.0	15.0	30	49.0	18.0	23.3	15.0 (7.4, 22.6)	+ + + + + +
Matejko 2022	20	85.0	6.3	17	61.5	11.2	26.0	23.5 (17.8, 29.2)	+ ? + + + ?
Choudhary 2022	36	70.6	9.7	31	43.6	15.4	25.6	27.0 (20.9, 33.1)	+ + + + + +
Boucsein 2024	37	57.0	17.0	43	22.0	12.0	25.1	35.0 (28.6, 41.4)	+ + + + + +
**Overall**								25.3 (17.8, 32.8)	

Heterogeneity: $\tau^2 = 47.59$, $I^2 = 81.73\%$, $H^2 = 5.47$

Test of $\theta_i = \theta_j$: $Q(3) = 16.42$, $p = 0.00$

Test of $\theta = 0$: $z = 6.62$, $p = 0.00$

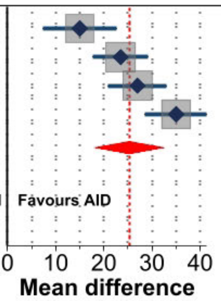

Random-effects DerSimonian–Laird model
Sorted by: _meta_es

Time in range 70-180 mg/dl - AHCL vs. CSII

Study	AHCL N	AHCL Mean	AHCL SD	CSII N	CSII Mean	CSII SD	Weight %	Mean difference (95% CI)	Risk of Bias A B C D E F
McVean 2023	59	78.0	11.0	47	64.0	22.0	100.0	14.0 (7.6, 20.4)	+ + + + + +
**Overall**								14.0 (7.6, 20.4)	

Heterogeneity: $\tau^2 = 0.00$, $I^2 = .\%$, $H^2 = .$

Test of $\theta_i = \theta_j$: $Q(0) = 0.00$, $p = .$

Test of $\theta = 0$: $z = 4.27$, $p = 0.00$

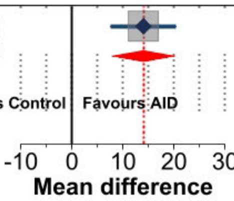

Random-effects DerSimonian–Laird model
Sorted by: _meta_es

Time in range 70-180 mg/dl - AHCL vs. SAP

Study	AHCL N	AHCL Mean	AHCL SD	SAP N	SAP Mean	SAP SD	Weight %	Mean difference (95% CI)	Risk of Bias A B C D E F
Ware 2022a	52	54.0	17.0	62	47.0	12.0	6.8	7.0 (1.7, 12.3)	+ + + + + +
Kariywasam 2022	17	66.2	1.5	17	58.7	1.5	11.0	7.5 (6.5, 8.5)	+ + + + + +
Kudva 2025	81	74.0	10.0	78	66.0	12.0	8.9	8.0 (4.6, 11.4)	+ + + + + +
Boughton 2022b	20	79.9	7.9	17	71.4	13.2	5.4	8.5 (1.6, 15.4)	+ + + + + +
Ware 2022b	73	71.6	5.9	74	62.9	9.0	9.9	8.7 (6.2, 11.2)	+ + + + + +
Benhamou 2019	63	68.5	9.4	63	59.4	10.2	8.9	9.1 (5.7, 12.5)	+ + + + + +
Renard 2023	49	74.4	8.9	22	64.2	13.3	6.9	10.2 (5.0, 15.4)	+ ? + + + ?
Breton 2020	78	67.0	10.0	23	55.0	13.0	7.2	12.0 (7.0, 17.0)	+ + + + + +
Brown 2019	112	71.0	12.0	56	59.0	14.0	8.2	12.0 (7.9, 16.1)	+ + + + + +
Wadwa 2023	68	69.3	11.1	34	55.9	12.6	7.4	13.4 (8.6, 18.2)	+ + + + + +
Renard 2024	132	61.2	11.2	62	43.8	14.5	8.6	17.4 (13.7, 21.1)	? + + + ? ?
Abraham 2024	19	51.9	11.5	19	31.4	14.0	4.5	20.5 (12.4, 28.6)	+ + + + + +
Christensen 2024	20	75.1	5.4	20	54.1	12.8	6.1	21.0 (14.9, 27.1)	+ + + + ? ?
**Overall**								11.4 (9.2, 13.6)	

Heterogeneity: $\tau^2 = 11.44$, $I^2 = 79.46\%$, $H^2 = 4.87$

Test of $\theta_i = \theta_j$: $Q(12) = 58.42$, $p = 0.00$

Test of $\theta = 0$: $z = 10.04$, $p = 0.00$

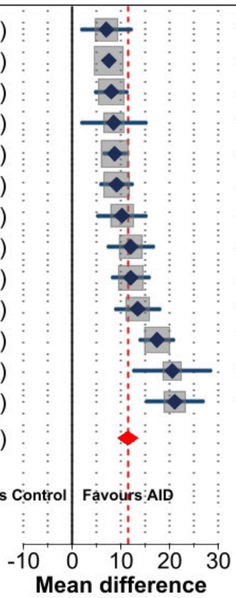

Random-effects DerSimonian–Laird model
Sorted by: _meta_es

Time in range 70-180 mg/dl - AHCL vs. PLGM

Study	AHCL N	AHCL Mean	AHCL SD	PLGM N	PLGM Mean	PLGM SD	Weight %	Mean difference (95% CI)	Risk of Bias A B C D E F
Benhalima 2024	48	84.2	7.0	45	80.7	9.1	20.1	3.5 (0.2, 6.8)	+ + + + + +
Kudva 2025	81	74.0	10.0	79	67.0	14.0	20.0	7.0 (3.2, 10.8)	+ + + + + +
Brown 2020	54	67.6	12.6	55	60.4	17.1	19.5	7.2 (1.6, 12.8)	+ + + + + +
Collyns 2021	59	70.4	8.1	59	57.9	11.7	20.0	12.5 (8.9, 16.1)	? + + + + ?
Benhamou 2021	5	73.3	1.7	5	43.5	1.7	20.3	29.8 (27.7, 31.9)	? + + + + ?
**Overall**								12.1 (0.1, 24.0)	

Heterogeneity: $\tau^2 = 182.16$, $I^2 = 98.40\%$, $H^2 = 62.63$
Test of $\theta_i = \theta_j$: Q(4) = 250.51, p = 0.00
Test of $\theta = 0$: z = 1.98, p = 0.05

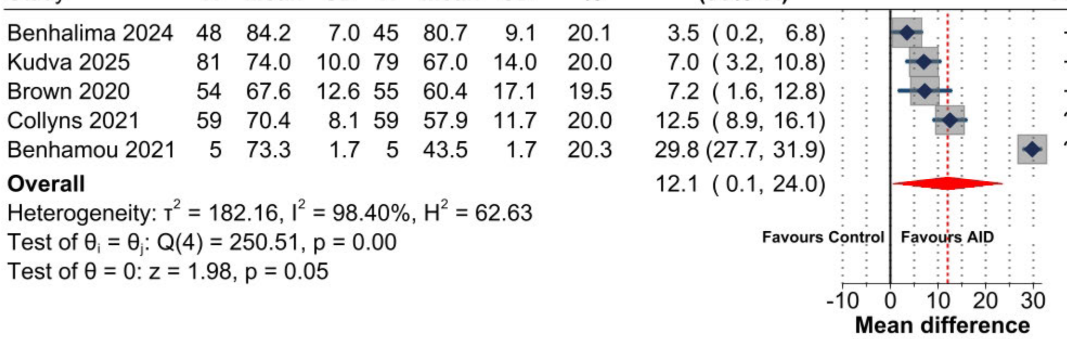

Random-effects DerSimonian–Laird model
Sorted by: _meta_es

Time in range 70-180 mg/dl - AHCL vs. HCL

Study	AHCL N	AHCL Mean	AHCL SD	HCL N	HCL Mean	HCL SD	Weight %	Mean difference (95% CI)	Risk of Bias A B C D E F
Bergenstal 2021	112	67.0	8.0	112	63.0	8.0	50.9	4.0 (1.9, 6.1)	+ + + + + +
Russell 2022	219	65.0	9.0	107	54.0	17.0	49.1	11.0 (8.2, 13.8)	+ + + + + +
**Overall**								7.4 (0.6, 14.3)	

Heterogeneity: $\tau^2 = 22.89$, $I^2 = 93.44\%$, $H^2 = 15.23$
Test of $\theta_i = \theta_j$: Q(1) = 15.23, p = 0.00
Test of $\theta = 0$: z = 2.12, p = 0.03

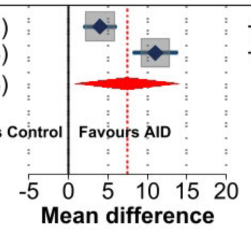

Random-effects DerSimonian–Laird model
Sorted by: _meta_es

Time in range 70-180 mg/dl - FCL vs. SAP

Study	FCL N	FCL Mean	FCL SD	SAP N	SAP Mean	SAP SD	Weight %	Mean difference (95% CI)	Risk of Bias A B C D E F
Boughton 2023	26	50.0	9.6	24	36.2	12.2	100.0	13.8 (7.7, 19.9)	? + + + + ?
**Overall**								13.8 (7.7, 19.9)	

Heterogeneity: $\tau^2 = 0.00$, $I^2 = .\%$, $H^2 = .$
Test of $\theta_i = \theta_j$: Q(0) = 0.00, p = .
Test of $\theta = 0$: z = 4.46, p = 0.00

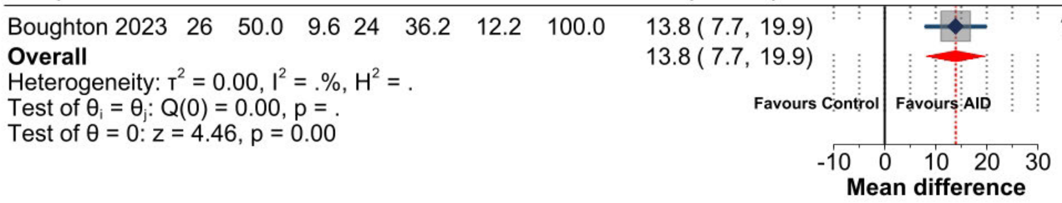

Random-effects DerSimonian–Laird model
Sorted by: _meta_es

TIR 70-180 mg/dl – FCL vs. MDI/CSII/PLGM/HCL/AHCL: no study

4.3 Pairwise meta-analyses TAR >180 mg/dl

Time above range >180 mg/dl - HCL vs. MDI

Study	HCL N	HCL Mean	HCL SD	MDI N	MDI Mean	MDI SD	Weight %	Mean difference (95% CI)	Risk of Bias A B C D E F
McAuley 2020	57	27.6	9.5	53	40.3	14.4	100.0	-12.7 (-17.2, -8.2)	+ + + + + +
**Overall**								-12.7 (-17.2, -8.2)	

Heterogeneity: $\tau^2 = 0.00$, $I^2 = .\%$, $H^2 = .$
Test of $\theta_i = \theta_j$: Q(0) = -0.00, p = .
Test of θ = 0: z = -5.50, p = 0.00

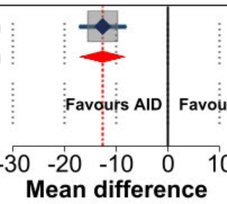

Random-effects DerSimonian–Laird model
Sorted by: _meta_es

Time above range >180 mg/dl - HCL vs. CSII

Study	HCL N	HCL Mean	HCL SD	CSII N	CSII Mean	CSII SD	Weight %	Mean difference (95% CI)	Risk of Bias A B C D E F
Nanayakkara 2023	20	21.3	11.5	20	37.9	14.3	33.6	-16.6 (-24.6, -8.6)	? + + + + ?
Burckhardt 2021	16	30.0	9.5	16	41.7	28.7	16.1	-11.7 (-26.5, 3.1)	? + + + + ?
Bally 2017	29	20.4	6.3	28	27.4	9.6	50.3	-7.0 (-11.2, -2.8)	+ + + + + +
**Overall**								-11.0 (-17.9, -4.1)	

Heterogeneity: $\tau^2 = 20.00$, $I^2 = 54.71\%$, $H^2 = 2.21$
Test of $\theta_i = \theta_j$: Q(2) = 4.42, p = 0.11
Test of θ = 0: z = -3.12, p = 0.00

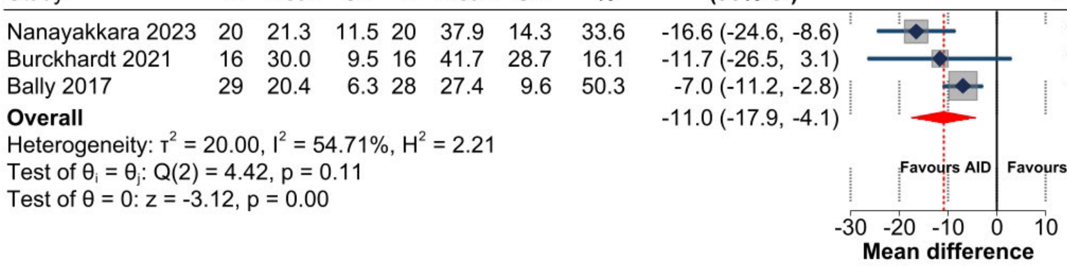

Random-effects DerSimonian–Laird model
Sorted by: _meta_es

Time above range >180 mg/dl - HCL vs. SAP

Study	HCL N	HCL Mean	HCL SD	SAP N	SAP Mean	SAP SD	Weight %	Mean difference (95% CI)	Risk of Bias A B C D E F
Tauschmann 2016	12	29.7	9.2	12	49.1	16.5	5.7	-19.4 (-30.1, -8.7)	? + + + + ?
Anderson 2019	21	19.8	10.1	21	35.6	17.6	6.9	-15.8 (-24.5, -7.1)	? + + + ? ?
Tauschmann 2018	46	32.0	8.0	40	42.0	10.0	10.5	-10.0 (-13.8, -6.2)	+ + + + + +
Thabit 2015	32	29.2	11.4	33	38.9	16.6	8.2	-9.7 (-16.6, -2.8)	+ + + + + +
McAuley 2022	30	23.6	6.6	30	29.0	9.8	10.2	-5.4 (-9.6, -1.2)	+ + + + + +
Kovatchev 2020	64	34.0	11.0	61	39.0	15.0	10.0	-5.0 (-9.6, -0.4)	+ + + + + +
Kim 2024	53	27.0	11.4	51	30.8	15.7	9.5	-3.8 (-9.1, 1.5)	+ + + + ? ?
Pinsker 2022	35	29.0	15.0	35	30.0	16.0	7.9	-1.0 (-8.3, 6.3)	? + + + + ?
Stewart 2018	16	14.6	1.0	16	14.8	1.0	12.0	-0.2 (-0.9, 0.5)	+ + + + ? ?
Donovan 2023	9	18.7	8.8	9	15.9	7.7	7.6	2.8 (-4.8, 10.4)	+ + + + + +
Polsky 2024	11	21.1	2.4	12	15.2	2.4	11.6	5.9 (3.9, 7.9)	? + + + + ?
**Overall**								-4.7 (-8.2, -1.1)	

Heterogeneity: $\tau^2 = 26.56$, $I^2 = 90.64\%$, $H^2 = 10.69$
Test of $\theta_i = \theta_j$: Q(10) = 106.86, p = 0.00
Test of θ = 0: z = -2.60, p = 0.01

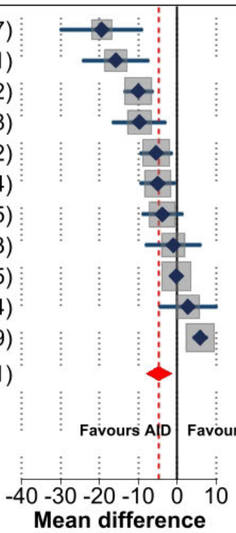

Random-effects DerSimonian–Laird model
Sorted by: _meta_es

Time above range >180 mg/dl - HCL vs. PLGM

Study	HCL N	HCL Mean	HCL SD	PLGM N	PLGM Mean	PLGM SD	Weight %	Mean difference (95% CI)	Risk of Bias A B C D E F
von dem Berge 2022	37	25.8	8.1	37	36.5	15.2	48.0	-10.7 (-16.2, -5.2)	? + + + ? ?
Abraham 2021	58	34.4	13.0	63	37.9	13.8	52.0	-3.5 (-8.3, 1.3)	+ + + + + +
**Overall**								-7.0 (-14.0, 0.1)	

Heterogeneity: $\tau^2 = 18.93$, $I^2 = 73.03\%$, $H^2 = 3.71$
Test of $\theta_i = \theta_j$: Q(1) = 3.71, p = 0.05
Test of θ = 0: z = -1.93, p = 0.05

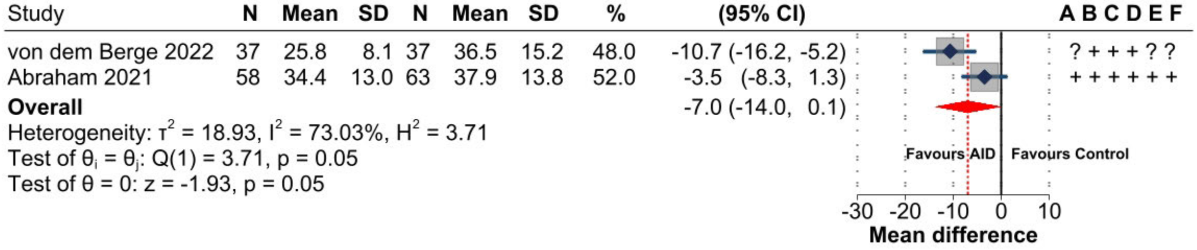

Random-effects DerSimonian–Laird model
Sorted by: _meta_es

Time above range >180 mg/dl - AHCL vs. MDI

Study	AHCL N	AHCL Mean	AHCL SD	MDI N	MDI Mean	MDI SD	Weight %	Mean difference (95% CI)	Risk of Bias A B C D E F
Boucsein 2024	37	42.0	18.0	43	74.0	15.0	19.6	-32.0 (-39.2, -24.8)	+ + + + + +
Choudhary 2022	36	26.7	10.4	31	53.8	16.5	19.9	-27.1 (-33.6, -20.6)	+ + + + + +
Boughton 2022a	42	22.0	11.0	30	42.0	19.0	19.7	-20.0 (-27.0, -13.0)	+ + + + + +
Matejko 2022	20	12.9	5.8	17	30.5	14.2	19.8	-17.6 (-24.4, -10.8)	+ ? + + + ?
Lee 2023	59	11.0	9.0	61	17.0	11.0	21.1	-6.0 (-9.6, -2.4)	+ + + + + +
**Overall**								-20.3 (-30.7, -10.0)	

Heterogeneity: $\tau^2 = 129.61$, $I^2 = 93.43\%$, $H^2 = 15.22$
Test of $\theta_i = \theta_j$: Q(4) = 60.87, p = 0.00
Test of θ = 0: z = -3.84, p = 0.00

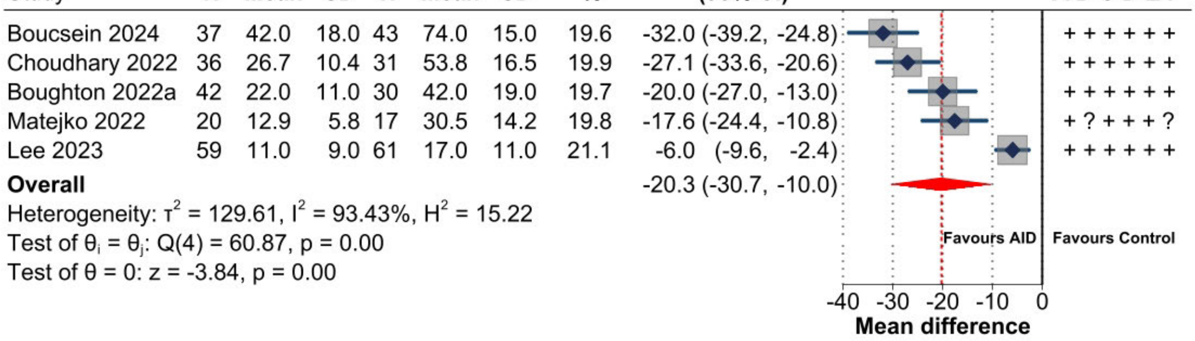

Random-effects DerSimonian–Laird model
Sorted by: _meta_es

Time above range >180 mg/dl - AHCL vs. CSII

Study	AHCL N	AHCL Mean	AHCL SD	CSII N	CSII Mean	CSII SD	Weight %	Mean difference (95% CI)	Risk of Bias A B C D E F
McVean 2023	59	20.0	11.0	47	34.0	22.0	100.0	-14.0 (-20.4, -7.6)	+ + + + + +
**Overall**								-14.0 (-20.4, -7.6)	

Heterogeneity: $\tau^2 = 0.00$, $I^2 = .\%$, $H^2 = .$
Test of $\theta_i = \theta_j$: Q(0) = 0.00, p = .
Test of θ = 0: z = -4.27, p = 0.00

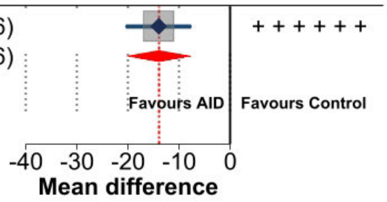

Random-effects DerSimonian–Laird model
Sorted by: _meta_es

Time above range >180 mg/dl - AHCL vs. SAP

Study	AHCL N	AHCL Mean	AHCL SD	SAP N	SAP Mean	SAP SD	Weight %	Mean difference (95% CI)	Risk of Bias A B C D E F
Abraham 2024	19	46.0	11.8	19	65.3	16.0	4.4	-19.3 (-28.2, -10.4)	+ + + + + +
Renard 2024	132	37.6	11.4	62	54.5	15.4	8.6	-16.9 (-20.8, -13.0)	? + + + ? ?
Wadwa 2023	68	28.0	11.0	34	41.0	13.0	7.7	-13.0 (-17.8, -8.2)	+ + + + + +
Breton 2020	78	31.0	10.0	23	43.0	14.0	7.4	-12.0 (-17.1, -6.9)	+ + + + + +
Brown 2019	112	27.0	12.0	56	38.0	15.0	8.3	-11.0 (-15.2, -6.8)	+ + + + + +
Christensen 2024	20	18.4	4.0	20	28.9	7.8	8.7	-10.5 (-14.3, -6.7)	+ + + + ? ?
Boughton 2022b	20	19.3	10.9	17	29.6	21.0	3.6	-10.3 (-20.8, 0.2)	+ + + + + +
Ware 2022a	52	38.0	20.0	62	46.0	15.0	6.2	-8.0 (-14.4, -1.6)	+ + + + + +
Ware 2022b	73	23.8	6.4	74	31.6	12.0	9.4	-7.8 (-10.9, -4.7)	+ + + + + +
Kudva 2025	81	24.0	10.0	78	31.0	13.0	8.9	-7.0 (-10.6, -3.4)	+ + + + + +
Benhamou 2019	63	29.5	10.2	63	36.3	10.2	8.9	-6.8 (-10.4, -3.2)	+ + + + + +
Renard 2023	49	22.8	8.9	22	28.9	13.6	7.2	-6.1 (-11.4, -0.8)	+ ? + + + ?
Kariywasam 2022	17	31.1	1.8	17	36.1	1.8	10.8	-5.0 (-6.2, -3.8)	+ + + + + +
**Overall**								-9.8 (-12.2, -7.4)	

Heterogeneity: $\tau^2 = 13.66$, $I^2 = 79.90\%$, $H^2 = 4.98$
Test of $\theta_i = \theta_j$: Q(12) = 59.71, p = 0.00
Test of $\theta = 0$: z = -7.98, p = 0.00

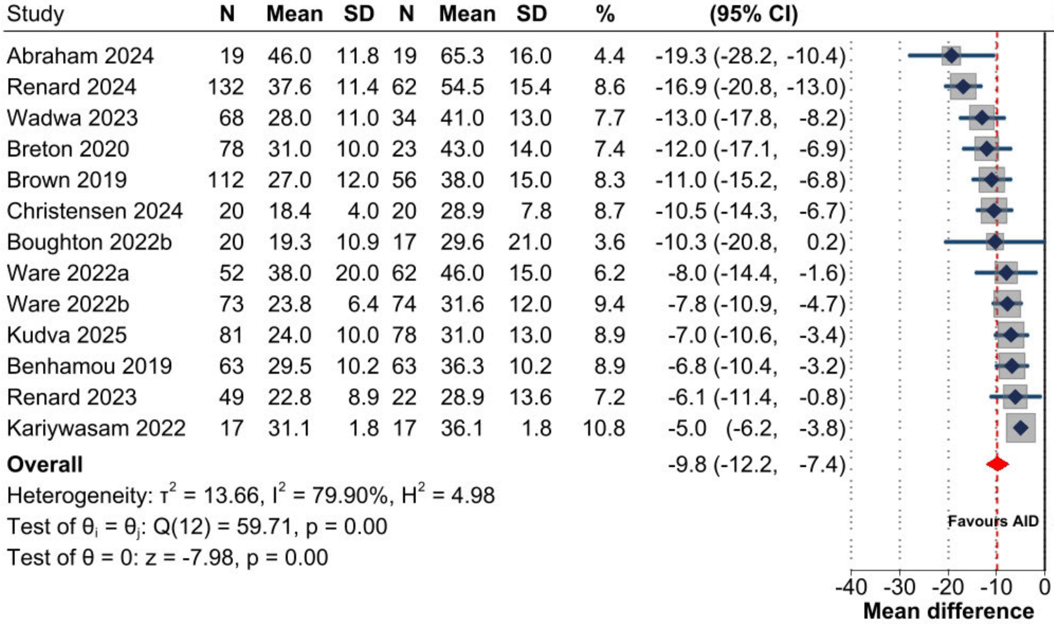

Random-effects DerSimonian–Laird model
Sorted by: _meta_es

Time above range >180 mg/dl - AHCL vs. PLGM

Study	AHCL N	AHCL Mean	AHCL SD	PLGM N	PLGM Mean	PLGM SD	Weight %	Mean difference (95% CI)	Risk of Bias A B C D E F
Benhamou 2021	5	25.8	1.9	5	52.8	1.9	20.3	-27.0 (-29.4, -24.6)	? + + + + ?
Collyns 2021	59	27.5	8.1	59	39.6	12.1	20.1	-12.1 (-15.8, -8.4)	? + + + + ?
Brown 2020	54	31.0	13.0	55	38.0	18.0	19.4	-7.0 (-12.9, -1.1)	+ + + + + +
Kudva 2025	81	24.0	10.0	79	31.0	14.0	20.0	-7.0 (-10.8, -3.2)	+ + + + + +
Benhalima 2024	48	11.3	6.9	45	12.3	9.0	20.2	-1.0 (-4.2, 2.2)	+ + + + + +
**Overall**								-10.9 (-21.9, 0.1)	

Heterogeneity: $\tau^2 = 153.09$, $I^2 = 97.98\%$, $H^2 = 49.41$
Test of $\theta_i = \theta_j$: Q(4) = 197.64, p = 0.00
Test of $\theta = 0$: z = -1.94, p = 0.05

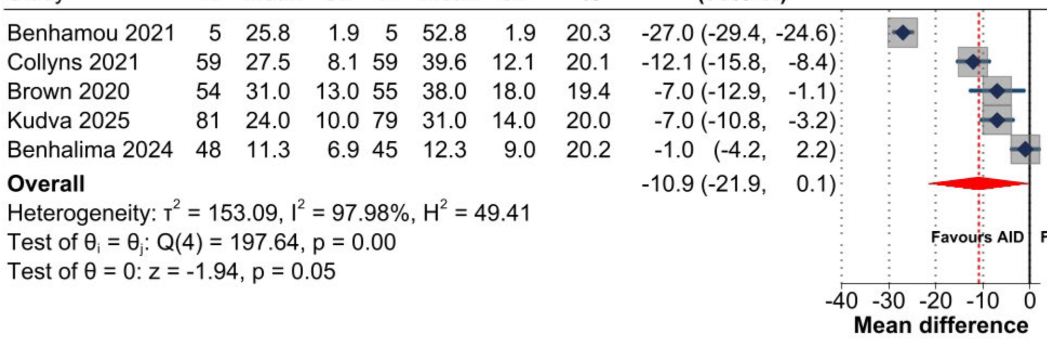

Random-effects DerSimonian–Laird model
Sorted by: _meta_es

Time above range >180 mg/dl - AHCL vs. HCL

Study	AHCL N	AHCL Mean	AHCL SD	HCL N	HCL Mean	HCL SD	Weight %	Mean difference (95% CI)	Risk of Bias A B C D E F
Russell 2022	219	33.0	9.0	107	44.0	18.0	49.1	-11.0 (-13.9, -8.1)	+ + + + + +
Bergenstal 2021	112	31.0	8.0	112	34.0	8.0	50.9	-3.0 (-5.1, -0.9)	+ + + + + +
**Overall**								-6.9 (-14.8, 0.9)	

Heterogeneity: $\tau^2 = 30.31$, $I^2 = 94.73\%$, $H^2 = 18.96$
Test of $\theta_i = \theta_j$: Q(1) = 18.96, p = 0.00
Test of $\theta = 0$: z = -1.73, p = 0.08

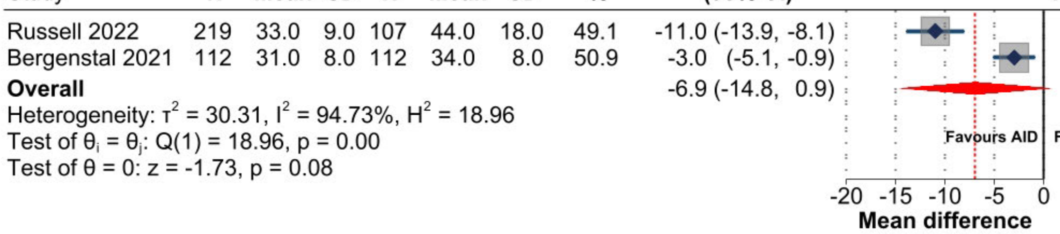

Random-effects DerSimonian–Laird model
Sorted by: _meta_es

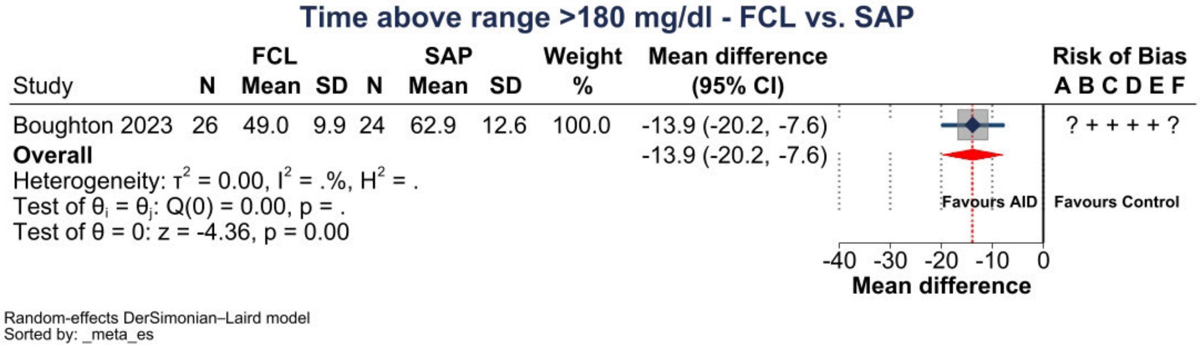

TAR >180 mg/dl – FCL vs. MDI/CSII/PLGM/HCL/AHCL: no study

4.4 Pairwise meta-analyses TAR >250 mg/dl

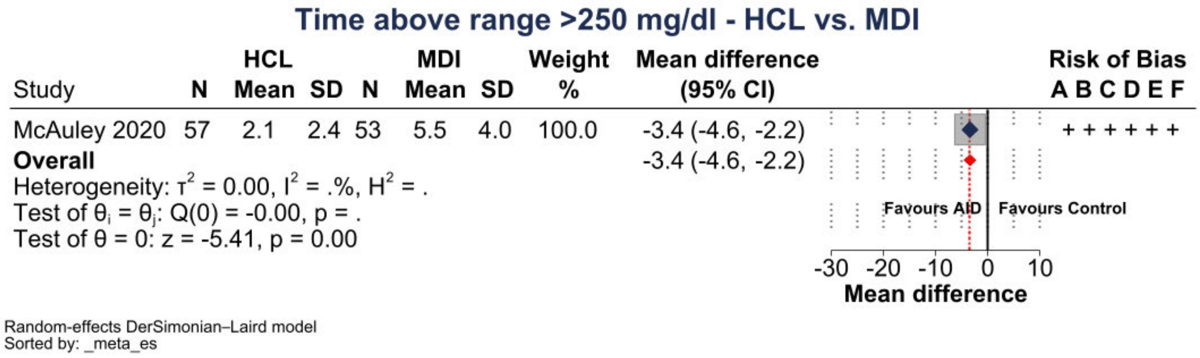

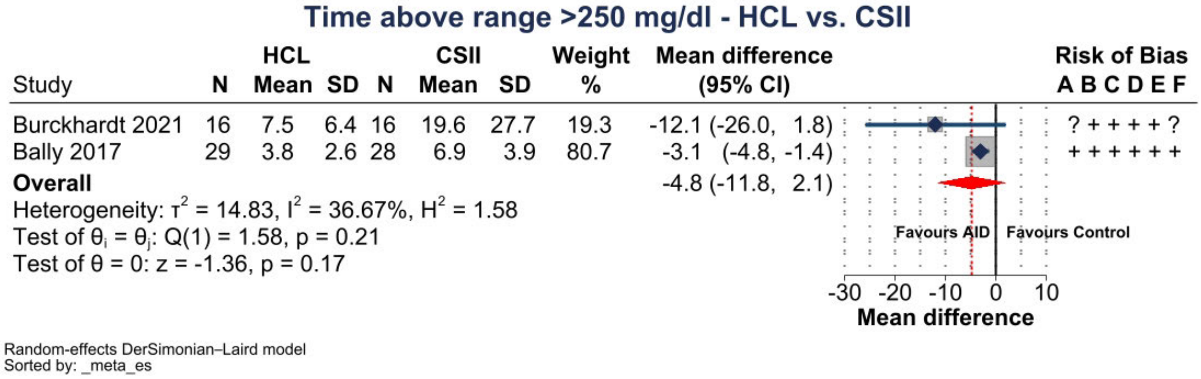

Time above range >250 mg/dl - HCL vs. SAP

Study	HCL N	HCL Mean	HCL SD	SAP N	SAP Mean	SAP SD	Weight %	Mean difference (95% CI)	Risk of Bias A B C D E F
Burnside 2022 <16y	20	9.3	6.0	27	18.8	14.7	9.1	-9.5 (-16.3, -2.7)	+ + + + + +
Burnside 2022 ≥16y	22	5.6	4.9	26	13.8	9.3	12.2	-8.2 (-12.5, -3.9)	+ + + + + +
Anderson 2019	21	3.6	3.9	21	11.2	9.4	12.2	-7.6 (-12.0, -3.2)	? + + + ? ?
McAuley 2022	30	4.4	2.6	30	8.9	15.1	10.7	-4.6 (-10.1, 0.9)	+ + + + + +
Kovatchev 2020	64	11.2	7.4	61	13.7	11.4	13.4	-2.5 (-5.9, 0.9)	+ + + + + +
Kim 2024	53	7.7	6.2	51	9.9	8.9	13.9	-2.2 (-5.1, 0.7)	+ + + + ? ?
Pinsker 2022	35	9.0	8.0	35	8.0	8.0	12.9	1.0 (-2.7, 4.7)	? + + + + ?
Polsky 2024	11	4.3	0.7	12	2.6	0.7	15.6	1.7 (1.1, 2.3)	? + + + + ?
**Overall**								-3.5 (-6.7, -0.3)	

Heterogeneity: $\tau^2 = 17.01$, $I^2 = 88.31\%$, $H^2 = 8.55$
Test of $\theta_i = \theta_j$: Q(7) = 59.87, p = 0.00
Test of $\theta = 0$: z = -2.16, p = 0.03

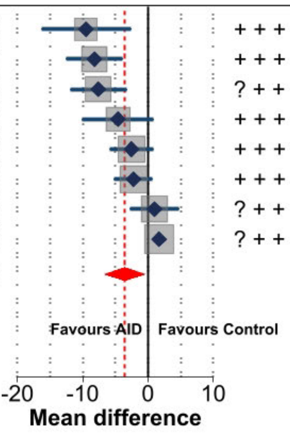

Random-effects DerSimonian–Laird model
Sorted by: _meta_es

Time above range >250 mg/dl - HCL vs. PLGM

Study	HCL N	HCL Mean	HCL SD	PLGM N	PLGM Mean	PLGM SD	Weight %	Mean difference (95% CI)	Risk of Bias A B C D E F
Abraham 2021	58	12.3	4.0	63	12.6	6.3	100.0	-0.4 (-2.3, 1.5)	+ + + + + +
**Overall**								-0.4 (-2.3, 1.5)	

Heterogeneity: $\tau^2 = 0.00$, $I^2 = .\%$, $H^2 = .$
Test of $\theta_i = \theta_j$: Q(0) = -0.00, p = .
Test of $\theta = 0$: z = -0.37, p = 0.71

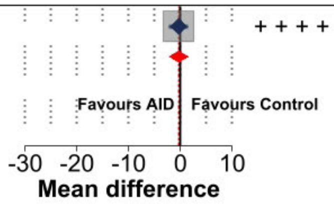

Random-effects DerSimonian–Laird model
Sorted by: _meta_es

Time above range >250 mg/dl - AHCL vs. MDI

Study	AHCL N	AHCL Mean	AHCL SD	MDI N	MDI Mean	MDI SD	Weight %	Mean difference (95% CI)	Risk of Bias A B C D E F
Boucsein 2024	37	19.0	18.0	43	53.0	20.0	30.8	-34.0 (-42.4, -25.6)	+ + + + + +
Choudhary 2022	36	6.6	5.0	31	22.5	13.2	34.3	-15.9 (-20.6, -11.2)	+ + + + + +
Matejko 2022	20	1.6	1.6	17	9.3	8.3	34.9	-7.7 (-11.4, -4.0)	+ ? + + + ?
**Overall**								-18.6 (-30.8, -6.4)	

Heterogeneity: $\tau^2 = 107.49$, $I^2 = 94.00\%$, $H^2 = 16.66$
Test of $\theta_i = \theta_j$: Q(2) = 33.31, p = 0.00
Test of $\theta = 0$: z = -2.99, p = 0.00

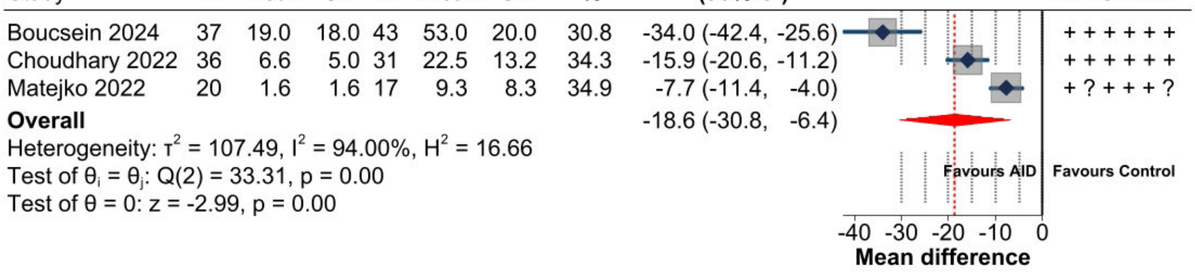

Random-effects DerSimonian–Laird model
Sorted by: _meta_es

Time above range >250 mg/dl - AHCL vs. CSII

Study	AHCL N	AHCL Mean	AHCL SD	CSII N	CSII Mean	CSII SD	Weight %	Mean difference (95% CI)	Risk of Bias A B C D E F
McVean 2023	59	4.0	4.0	47	10.0	13.0	100.0	-6.0 (-9.5, -2.5)	+ + + + + +
**Overall**								-6.0 (-9.5, -2.5)	

Heterogeneity: $\tau^2 = 0.00$, $I^2 = .\%$, $H^2 = .$
Test of $\theta_i = \theta_j$: $Q(0) = 0.00$, $p = .$
Test of $\theta = 0$: $z = -3.35$, $p = 0.00$

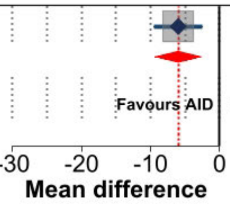

Random-effects DerSimonian–Laird model
Sorted by: _meta_es

Time above range >250 mg/dl - AHCL vs. SAP

Study	AHCL N	AHCL Mean	AHCL SD	SAP N	SAP Mean	SAP SD	Weight %	Mean difference (95% CI)	Risk of Bias A B C D E F
Abraham 2024	19	18.9	9.1	19	41.2	17.7	3.2	-22.3 (-31.2, -13.4)	+ + + + + +
Renard 2024	132	13.5	8.2	62	25.7	14.3	10.0	-12.2 (-15.4, -9.0)	? + + + ? ?
Christensen 2024	20	4.5	2.5	20	14.9	8.5	8.7	-10.4 (-14.3, -6.5)	+ + + + ? ?
Wadwa 2023	68	8.4	7.2	34	15.0	10.9	9.3	-6.6 (-10.1, -3.1)	+ + + + + +
Breton 2020	78	11.1	9.1	23	17.2	6.3	8.5	-6.1 (-10.1, -2.1)	+ + + + + +
Brown 2019	112	7.0	6.7	56	12.3	10.2	11.2	-5.3 (-7.9, -2.7)	+ + + + + +
Kariywasam 2022	17	8.1	1.1	17	12.9	1.1	14.1	-4.9 (-5.6, -4.2)	+ + + + + +
Benhamou 2019	63	7.4	6.3	63	11.7	6.3	11.9	-4.3 (-6.5, -2.1)	+ + + + + +
Renard 2023	49	5.5	3.9	22	9.2	8.7	10.5	-3.7 (-6.6, -0.8)	+ ? + + + ?
Kudva 2025	81	5.6	5.2	78	8.6	7.0	12.4	-3.0 (-4.9, -1.1)	+ + + + + +
**Overall**								-6.5 (-8.4, -4.7)	

Heterogeneity: $\tau^2 = 6.00$, $I^2 = 81.15\%$, $H^2 = 5.31$
Test of $\theta_i = \theta_j$: $Q(9) = 47.75$, $p = 0.00$
Test of $\theta = 0$: $z = -7.04$, $p = 0.00$

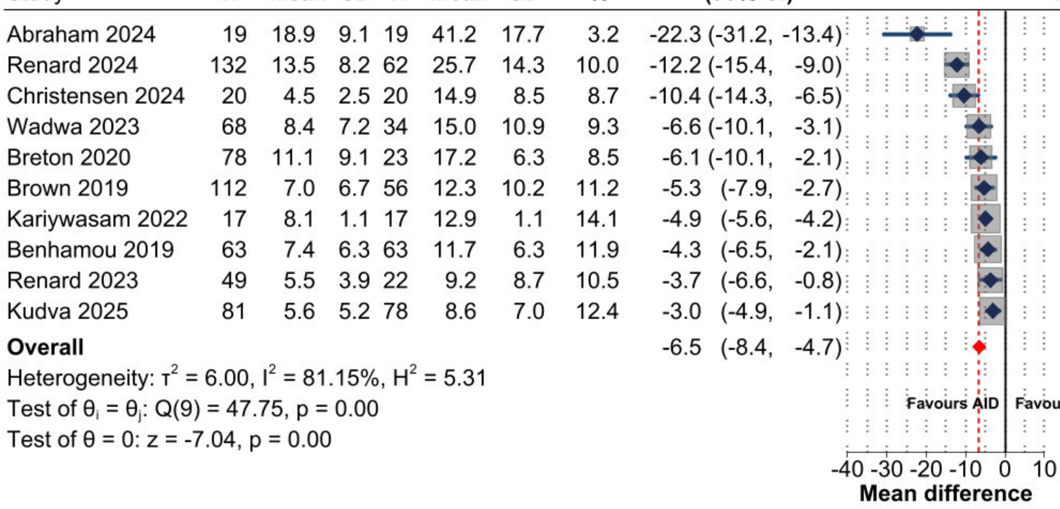

Random-effects DerSimonian–Laird model
Sorted by: _meta_es

Time above range >250 mg/dl - AHCL vs. PLGM

Study	AHCL N	AHCL Mean	AHCL SD	PLGM N	PLGM Mean	PLGM SD	Weight %	Mean difference (95% CI)	Risk of Bias A B C D E F
Benhamou 2021	5	3.9	1.4	5	23.0	1.4	33.7	-19.1 (-20.8, -17.4)	? + + + + ?
Brown 2020	54	8.4	7.1	55	12.8	12.2	32.8	-4.4 (-8.2, -0.6)	+ + + + + +
Kudva 2025	81	5.6	5.2	79	8.7	8.8	33.5	-3.1 (-5.3, -0.9)	+ + + + + +
**Overall**								-8.9 (-20.6, 2.8)	

Heterogeneity: $\tau^2 = 104.67$, $I^2 = 98.58\%$, $H^2 = 70.51$
Test of $\theta_i = \theta_j$: $Q(2) = 141.03$, $p = 0.00$
Test of $\theta = 0$: $z = -1.50$, $p = 0.13$

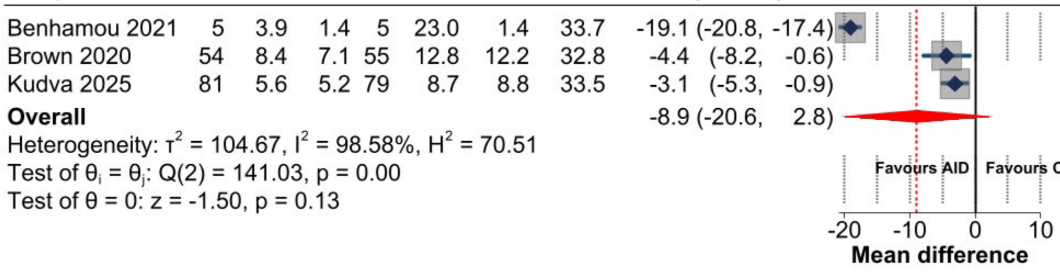

Random-effects DerSimonian–Laird model
Sorted by: _meta_es

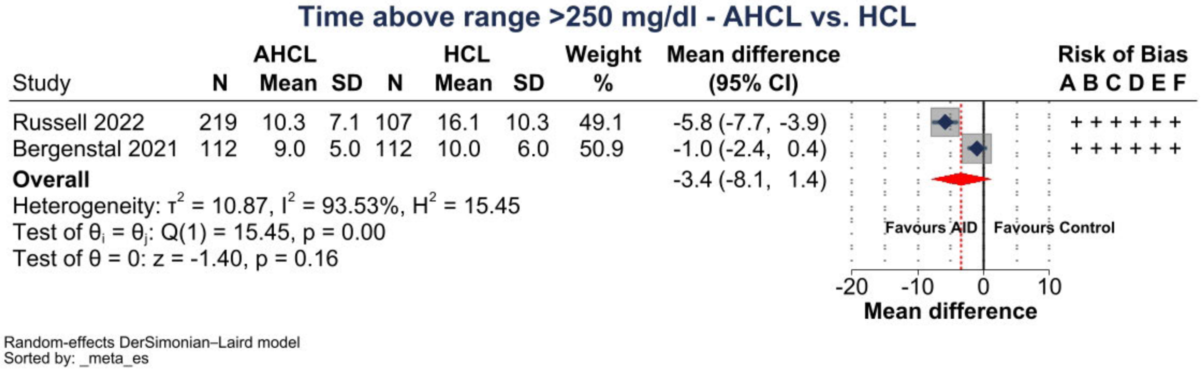

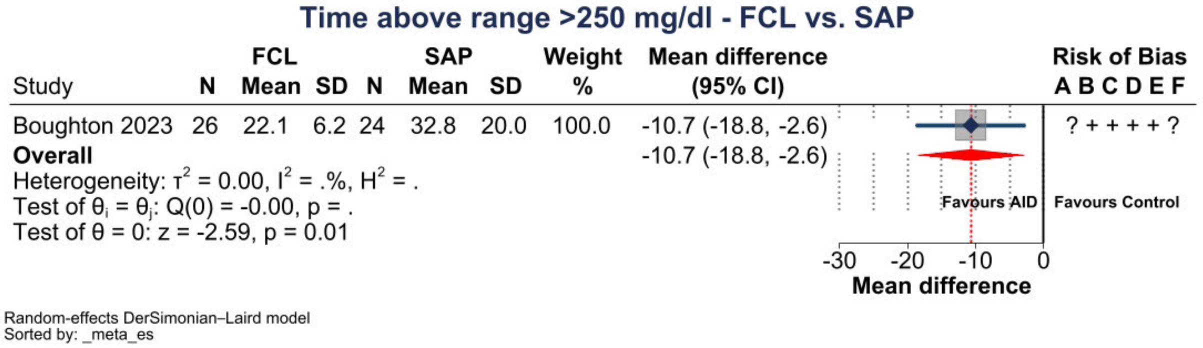

TAR >250 mg/dl – FCL vs. MDI/CSII/PLGM/HCL/AHCL: no study

4.5 Pairwise meta-analyses TBR <70 mg/dl

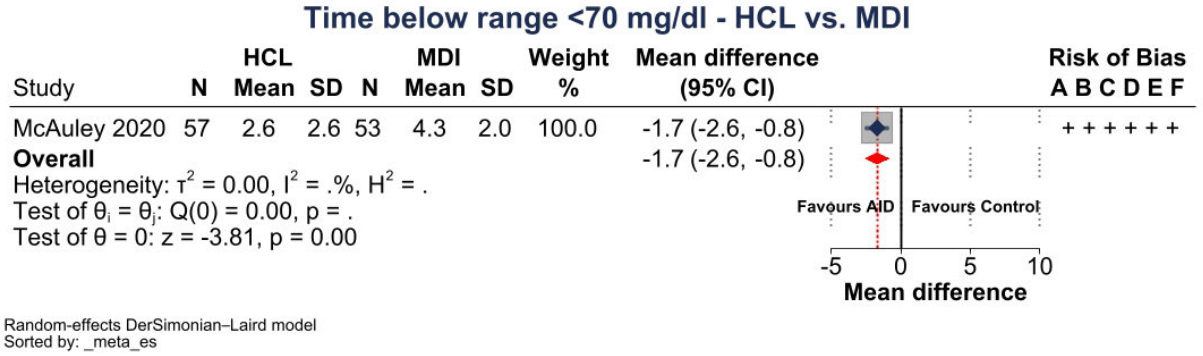

Time below range <70 mg/dl - HCL vs. CSII

Study	HCL N	HCL Mean	HCL SD	CSII N	CSII Mean	CSII SD	Weight %	Mean difference (95% CI)	Risk of Bias A B C D E F
Burckhardt 2021	16	1.7	0.9	16	7.5	7.3	10.4	-5.8 (-9.4, -2.2)	? + + + + ?
Bally 2017	29	3.3	1.5	28	8.4	8.7	12.5	-5.1 (-8.3, -1.8)	+ + + + + +
Garg 2023	140	2.4	2.1	138	6.0	5.4	49.0	-3.6 (-4.6, -2.6)	? + + + + ?
Nanayakkara 2023	20	2.1	1.7	20	4.2	3.8	28.2	-2.1 (-3.9, -0.3)	? + + + + ?
**Overall**								-3.6 (-4.8, -2.3)	

Heterogeneity: $\tau^2 = 0.61$, $I^2 = 36.37\%$, $H^2 = 1.57$

Test of $\theta_i = \theta_j$: Q(3) = 4.71, p = 0.19

Test of $\theta = 0$: z = -5.56, p = 0.00

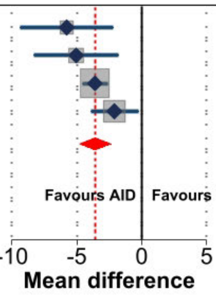

Random-effects DerSimonian–Laird model
Sorted by: _meta_es

Time below range <70 mg/dl - HCL vs. SAP

Study	HCL N	HCL Mean	HCL SD	SAP N	SAP Mean	SAP SD	Weight %	Mean difference (95% CI)	Risk of Bias A B C D E F
Polsky 2024	11	4.5	1.8	12	9.2	1.7	7.6	-4.7 (-6.1, -3.3)	? + + + + ?
Donovan 2023	9	1.7	0.8	9	5.5	3.3	5.0	-3.8 (-6.0, -1.6)	+ + + + + +
Anderson 2019	21	2.0	1.4	21	4.8	4.5	5.5	-2.8 (-4.8, -0.8)	? + + + ? ?
Thabit 2015	32	3.0	1.6	33	4.6	5.3	5.9	-1.7 (-3.6, 0.2)	+ + + + + +
Kovatchev 2020	64	2.4	1.7	61	4.0	3.4	9.7	-1.6 (-2.5, -0.7)	+ + + + + +
Pinsker 2022	35	2.1	1.7	35	3.5	3.1	8.7	-1.4 (-2.6, -0.2)	? + + + + ?
Tauschmann 2018	46	2.9	1.5	40	3.7	1.3	11.0	-0.8 (-1.4, -0.2)	+ + + + + +
Kim 2024	53	1.6	1.6	51	2.3	1.5	11.0	-0.7 (-1.3, -0.1)	+ + + + ? ?
McAuley 2022	30	1.2	0.4	30	1.9	1.2	11.5	-0.7 (-1.1, -0.2)	+ + + + + +
Burnside 2022 <16y	20	2.1	1.5	27	2.7	2.8	7.9	-0.6 (-2.0, 0.8)	+ + + + + +
Burnside 2022 ≥16y	22	1.6	2.1	26	1.8	2.5	8.0	-0.2 (-1.5, 1.1)	+ + + + + +
Tauschmann 2016	12	3.9	1.0	12	2.7	2.0	8.2	1.2 (-0.1, 2.5)	? + + + + ?
**Overall**								-1.3 (-1.9, -0.6)	

Heterogeneity: $\tau^2 = 0.88$, $I^2 = 79.29\%$, $H^2 = 4.83$

Test of $\theta_i = \theta_j$: Q(11) = 53.12, p = 0.00

Test of $\theta = 0$: z = -3.93, p = 0.00

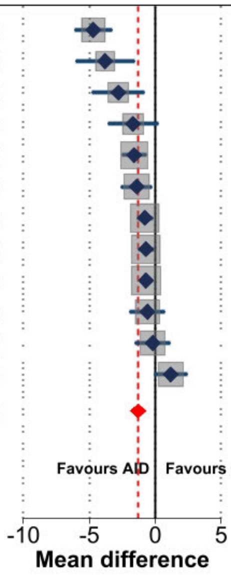

Random-effects DerSimonian–Laird model
Sorted by: _meta_es

Time below range <70 mg/dl - HCL vs. PLGM

Study	HCL N	HCL Mean	HCL SD	PLGM N	PLGM Mean	PLGM SD	Weight %	Mean difference (95% CI)	Risk of Bias A B C D E F
Abraham 2021	58	3.2	3.5	63	6.3	6.3	46.9	-3.1 (-4.9, -1.2)	+ + + + + +
von dem Berge 2022	37	3.4	2.1	37	3.2	1.9	53.1	0.2 (-0.7, 1.1)	? + + + ? ?
**Overall**								-1.3 (-4.6, 1.9)	

Heterogeneity: $\tau^2 = 4.91$, $I^2 = 89.95\%$, $H^2 = 9.95$

Test of $\theta_i = \theta_j$: Q(1) = 9.95, p = 0.00

Test of $\theta = 0$: z = -0.81, p = 0.42

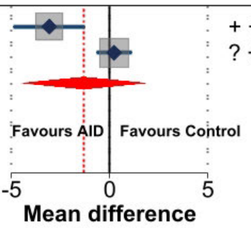

Random-effects DerSimonian–Laird model
Sorted by: _meta_es

Time below range <70 mg/dl mg/dl - AHCL vs. MDI

Study	AHCL N	AHCL Mean	AHCL SD	MDI N	MDI Mean	MDI SD	Weight %	Mean difference (95% CI)	Risk of Bias A B C D E F
Matejko 2022	20	2.1	1.7	17	8.1	7.1	17.8	-6.0 (-9.2, -2.8)	+ ? + + + ?
Boucsein 2024	37	1.6	1.6	43	2.4	1.5	33.6	-0.8 (-1.5, -0.1)	+ + + + + +
Choudhary 2022	36	2.6	2.0	31	2.6	2.5	31.5	0.0 (-1.1, 1.1)	+ + + + + +
Boughton 2022a	42	11.2	7.3	30	7.5	6.7	17.2	3.7 (0.4, 7.0)	+ + + + + +
**Overall**								-0.7 (-2.6, 1.2)	

Heterogeneity: $\tau^2 = 2.75$, $I^2 = 84.03\%$, $H^2 = 6.26$
Test of $\theta_i = \theta_j$: Q(3) = 18.79, p = 0.00
Test of $\theta = 0$: z = -0.70, p = 0.48

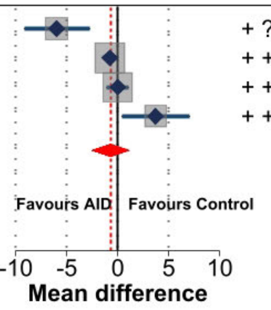

Random-effects DerSimonian–Laird model
Sorted by: _meta_es

Time below range <70 mg/dl mg/dl - AHCL vs. CSII

Study	AHCL N	AHCL Mean	AHCL SD	CSII N	CSII Mean	CSII SD	Weight %	Mean difference (95% CI)	Risk of Bias A B C D E F
McVean 2023	59	1.8	1.2	47	1.4	1.4	100.0	0.4 (-0.1, 0.9)	+ + + + + +
**Overall**								0.4 (-0.1, 0.9)	

Heterogeneity: $\tau^2 = 0.00$, $I^2 = .\%$, $H^2 = .$
Test of $\theta_i = \theta_j$: Q(0) = 0.00, p = .
Test of $\theta = 0$: z = 1.58, p = 0.11

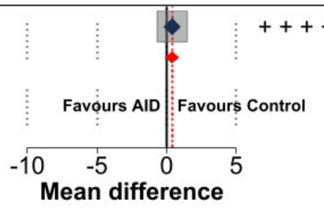

Random-effects DerSimonian–Laird model
Sorted by: _meta_es

Time below range <70 mg/dl mg/dl - AHCL vs. SAP

Study	AHCL N	AHCL Mean	AHCL SD	SAP N	SAP Mean	SAP SD	Weight %	Mean difference (95% CI)	Risk of Bias A B C D E F
Renard 2023	49	2.8	1.4	22	6.8	3.5	7.5	-4.0 (-5.1, -2.9)	+ ? + + + ?
Abraham 2024	19	1.9	1.1	19	5.6	12.1	1.1	-3.7 (-9.2, 1.7)	+ + + + + +
Kariywasam 2022	17	2.6	0.5	17	5.2	0.5	9.6	-2.6 (-3.0, -2.3)	+ + + + + +
Benhamou 2019	63	2.0	2.4	63	4.3	2.4	8.4	-2.3 (-3.1, -1.5)	+ + + + + +
Kudva 2025	81	1.6	0.9	78	2.6	1.5	9.6	-1.0 (-1.4, -0.6)	+ + + + + +
Breton 2020	78	1.6	0.8	23	2.4	2.0	9.2	-0.8 (-1.4, -0.3)	+ + + + + +
Brown 2019	112	1.6	1.1	56	2.3	1.5	9.5	-0.7 (-1.1, -0.3)	+ + + + + +
Renard 2024	132	1.2	0.9	62	1.8	1.7	9.6	-0.6 (-0.9, -0.2)	? + + + ? ?
Ware 2022b	73	5.0	2.4	74	5.5	4.1	7.6	-0.5 (-1.6, 0.5)	+ + + + + +
Christensen 2024	20	1.7	1.0	20	1.7	1.1	9.0	0.0 (-0.7, 0.7)	+ + + + ? ?
Wadwa 2023	68	3.0	1.8	34	3.0	2.2	8.5	0.0 (-0.8, 0.8)	+ + + + + +
Boughton 2022b	20	2.0	1.0	17	1.9	1.3	8.7	0.1 (-0.7, 0.8)	+ + + + + +
Ware 2022a	52	9.9	13.6	62	8.9	10.4	1.6	1.0 (-3.4, 5.4)	+ + + + + +
**Overall**								-1.1 (-1.7, -0.5)	

Heterogeneity: $\tau^2 = 1.00$, $I^2 = 91.71\%$, $H^2 = 12.07$
Test of $\theta_i = \theta_j$: Q(12) = 144.84, p = 0.00
Test of $\theta = 0$: z = -3.46, p = 0.00

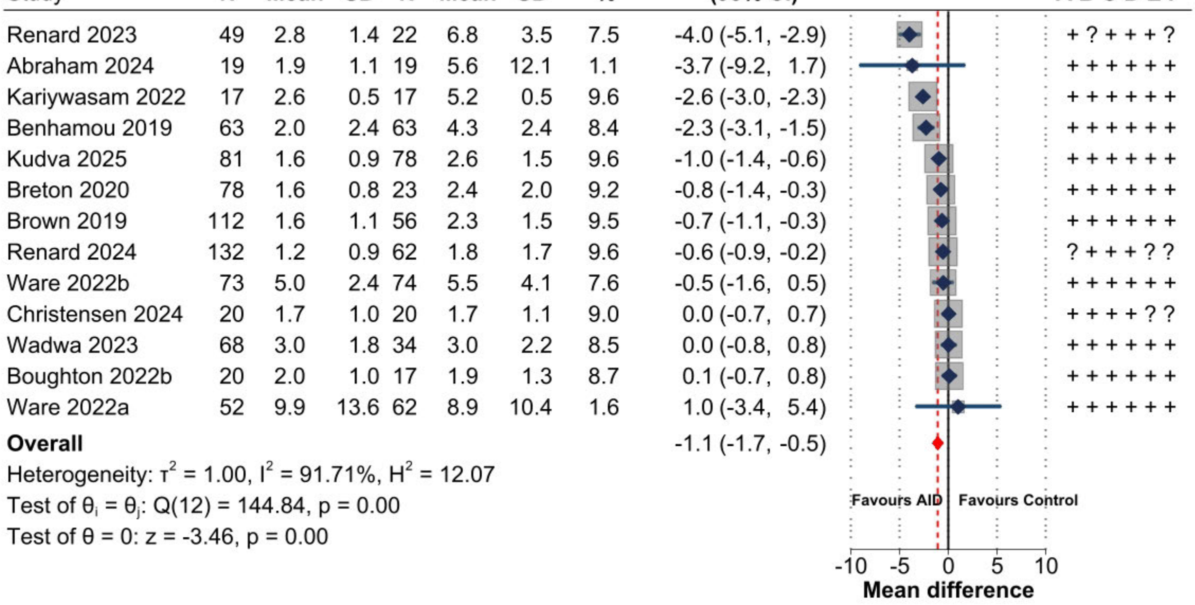

Random-effects DerSimonian–Laird model
Sorted by: _meta_es

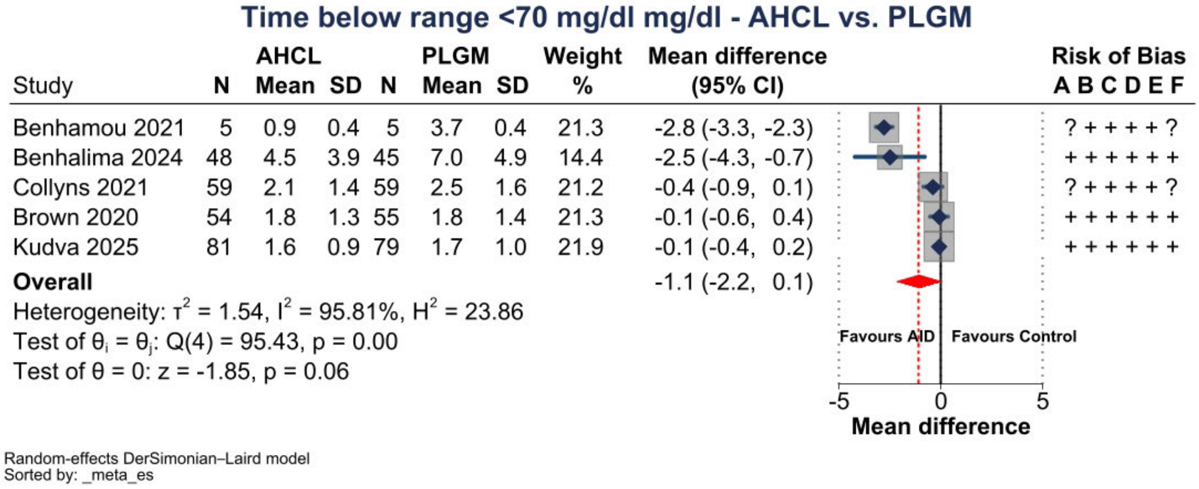

Study	AHCL N	AHCL Mean	AHCL SD	PLGM N	PLGM Mean	PLGM SD	Weight %	Mean difference (95% CI)	Risk of Bias A B C D E F
Benhamou 2021	5	0.9	0.4	5	3.7	0.4	21.3	-2.8 (-3.3, -2.3)	? + + + + ?
Benhalima 2024	48	4.5	3.9	45	7.0	4.9	14.4	-2.5 (-4.3, -0.7)	+ + + + + +
Collyns 2021	59	2.1	1.4	59	2.5	1.6	21.2	-0.4 (-0.9, 0.1)	? + + + + ?
Brown 2020	54	1.8	1.3	55	1.8	1.4	21.3	-0.1 (-0.6, 0.4)	+ + + + + +
Kudva 2025	81	1.6	0.9	79	1.7	1.0	21.9	-0.1 (-0.4, 0.2)	+ + + + + +
**Overall**								-1.1 (-2.2, 0.1)	

Heterogeneity: $\tau^2 = 1.54$, $I^2 = 95.81\%$, $H^2 = 23.86$
Test of $\theta_i = \theta_j$: Q(4) = 95.43, p = 0.00
Test of $\theta = 0$: z = -1.85, p = 0.06

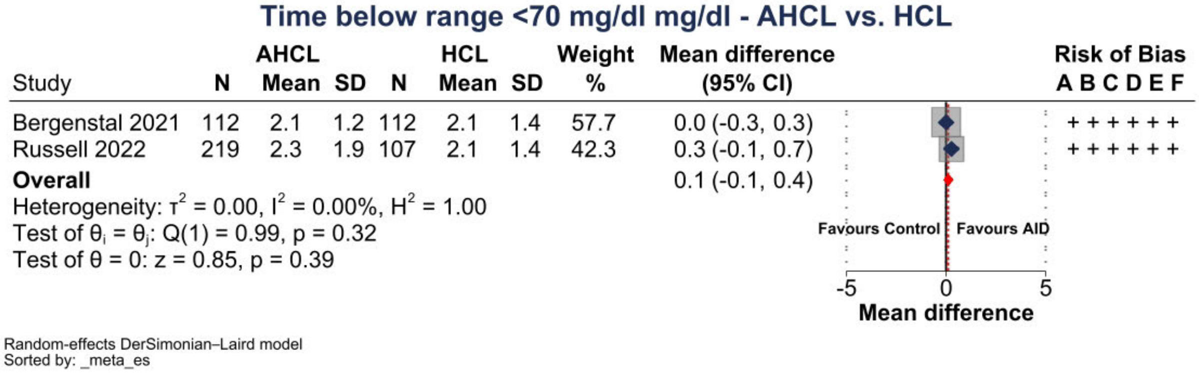

Study	AHCL N	AHCL Mean	AHCL SD	HCL N	HCL Mean	HCL SD	Weight %	Mean difference (95% CI)	Risk of Bias A B C D E F
Bergenstal 2021	112	2.1	1.2	112	2.1	1.4	57.7	0.0 (-0.3, 0.3)	+ + + + + +
Russell 2022	219	2.3	1.9	107	2.1	1.4	42.3	0.3 (-0.1, 0.7)	+ + + + + +
**Overall**								0.1 (-0.1, 0.4)	

Heterogeneity: $\tau^2 = 0.00$, $I^2 = 0.00\%$, $H^2 = 1.00$
Test of $\theta_i = \theta_j$: Q(1) = 0.99, p = 0.32
Test of $\theta = 0$: z = 0.85, p = 0.39

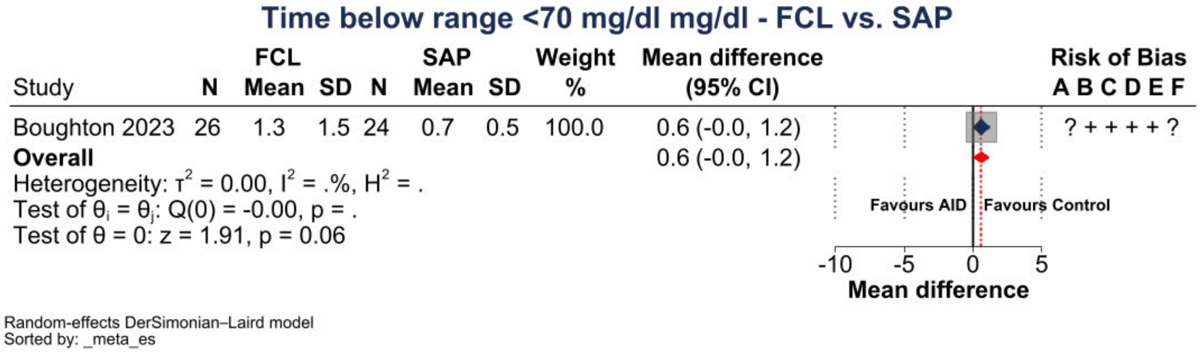

Study	FCL N	FCL Mean	FCL SD	SAP N	SAP Mean	SAP SD	Weight %	Mean difference (95% CI)	Risk of Bias A B C D E F
Boughton 2023	26	1.3	1.5	24	0.7	0.5	100.0	0.6 (-0.0, 1.2)	? + + + + ?
**Overall**								0.6 (-0.0, 1.2)	

Heterogeneity: $\tau^2 = 0.00$, $I^2 = .\%$, $H^2 = .$
Test of $\theta_i = \theta_j$: Q(0) = -0.00, p = .
Test of $\theta = 0$: z = 1.91, p = 0.06

TBR <70 mg/dl – FCL vs. MDI/CSII/PLGM/HCL/AHCL: no study

4.6 Pairwise meta-analyses TBR <54 mg/dl

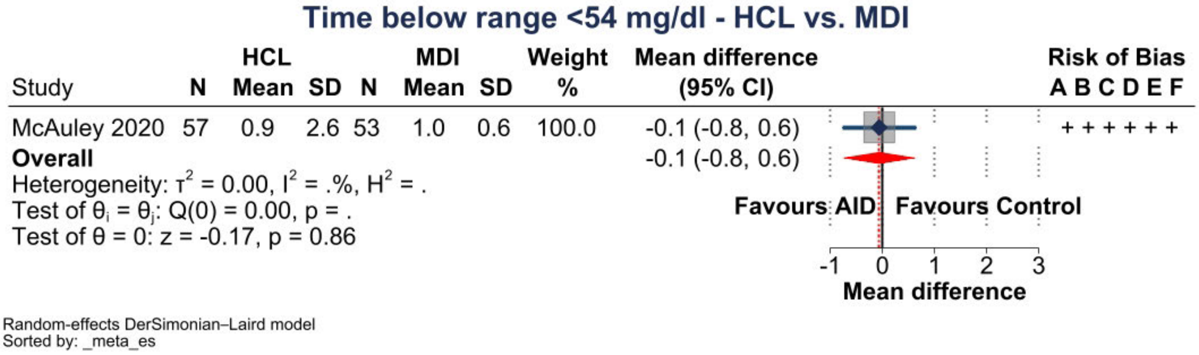

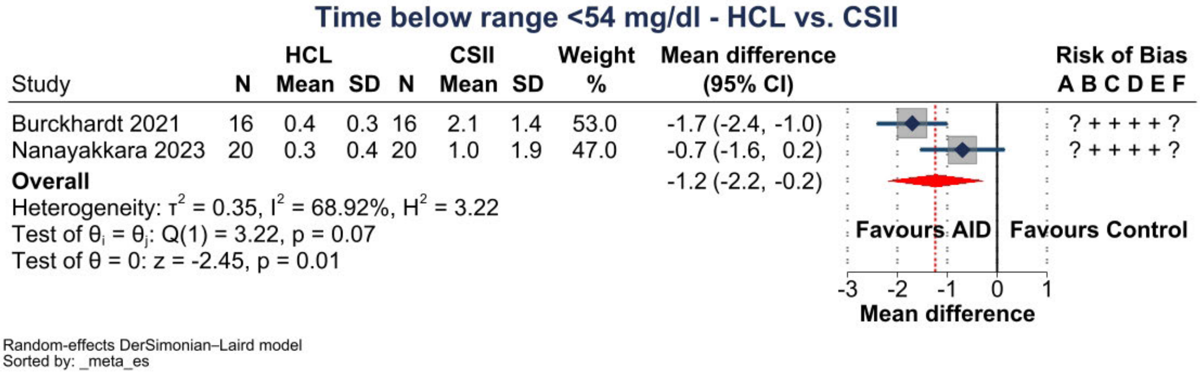

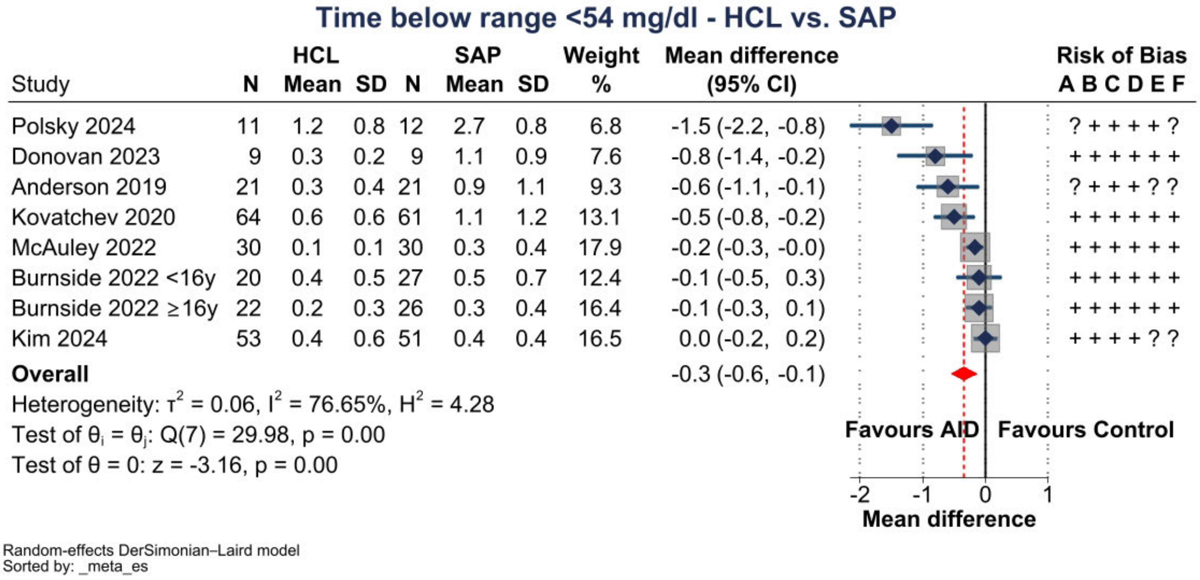

Time below range <54 mg/dl - HCL vs. PLGM

Study	HCL N	HCL Mean	HCL SD	PLGM N	PLGM Mean	PLGM SD	Weight %	Mean difference (95% CI)	Risk of Bias A B C D E F
Abraham 2021	58	0.8	1.4	63	2.0	2.9	45.6	-1.1 (-2.0, -0.3)	+ + + + + +
von dem Berge 2022	37	0.8	0.7	37	0.6	0.5	54.4	0.2 (-0.1, 0.5)	? + + + ? ?
**Overall**								-0.4 (-1.7, 0.9)	

Heterogeneity: $\tau^2 = 0.79$, $I^2 = 88.93\%$, $H^2 = 9.04$
Test of $\theta_i = \theta_j$: Q(1) = 9.04, p = 0.00
Test of $\theta = 0$: z = -0.61, p = 0.54

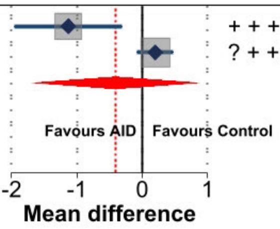

Random-effects DerSimonian–Laird model
Sorted by: _meta_es

Time below range <54 mg/dl - AHCL vs. MDI

Study	AHCL N	AHCL Mean	AHCL SD	MDI N	MDI Mean	MDI SD	Weight %	Mean difference (95% CI)	Risk of Bias A B C D E F
Matejko 2022	20	0.3	0.4	17	2.6	3.9	5.8	-2.3 (-4.0, -0.6)	+ ? + + + ?
Boucsein 2024	37	0.2	0.1	43	0.8	0.6	34.1	-0.6 (-0.8, -0.4)	+ + + + + +
Lee 2023	59	1.0	0.7	61	1.4	1.9	24.7	-0.4 (-0.9, 0.1)	+ + + + + +
Choudhary 2022	36	0.6	0.7	31	0.7	1.2	26.9	-0.1 (-0.5, 0.3)	+ + + + + +
Boughton 2022a	42	3.1	3.0	30	2.3	2.7	8.5	0.8 (-0.5, 2.1)	+ + + + + +
**Overall**								-0.4 (-0.9, 0.0)	

Heterogeneity: $\tau^2 = 0.14$, $I^2 = 67.54\%$, $H^2 = 3.08$
Test of $\theta_i = \theta_j$: Q(4) = 12.32, p = 0.02
Test of $\theta = 0$: z = -1.76, p = 0.08

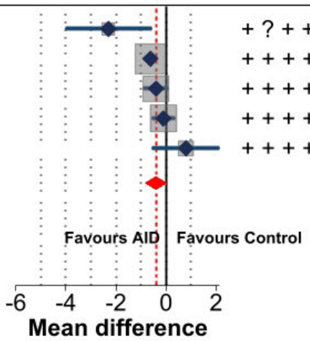

Random-effects DerSimonian–Laird model
Sorted by: _meta_es

Time below range <54 mg/dl - AHCL vs. CSII

Study	AHCL N	AHCL Mean	AHCL SD	CSII N	CSII Mean	CSII SD	Weight %	Mean difference (95% CI)	Risk of Bias A B C D E F
McVean 2023	59	0.3	0.3	47	0.2	0.2	100.0	0.1 (-0.0, 0.2)	+ + + + + +
**Overall**								0.1 (-0.0, 0.2)	

Heterogeneity: $\tau^2 = 0.00$, $I^2 = .\%$, $H^2 = .$
Test of $\theta_i = \theta_j$: Q(0) = 0.00, p = .
Test of $\theta = 0$: z = 1.94, p = 0.05

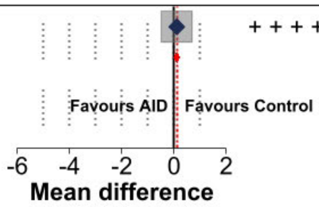

Random-effects DerSimonian–Laird model
Sorted by: _meta_es

Time below range <54 mg/dl - AHCL vs. SAP

Study	AHCL N	AHCL Mean	AHCL SD	SAP N	SAP Mean	SAP SD	Weight %	Mean difference (95% CI)	Risk of Bias A B C D E F
Renard 2023	49	0.6	0.5	22	1.5	1.2	4.9	-0.9 (-1.3, -0.5)	+ ? + + + ?
Kariywasam 2022	17	0.6	0.2	17	1.0	0.2	11.0	-0.4 (-0.6, -0.3)	+ + + + + +
Abraham 2024	19	0.5	0.4	19	0.8	0.6	6.5	-0.3 (-0.6, 0.0)	+ + + + + +
Kudva 2025	81	0.3	0.2	78	0.5	0.4	11.2	-0.2 (-0.3, -0.1)	+ + + + + +
Renard 2024	132	0.2	0.2	62	0.4	0.5	11.1	-0.1 (-0.2, -0.0)	? + + + ? ?
Brown 2019	112	0.3	0.3	56	0.3	0.3	11.3	-0.1 (-0.2, 0.0)	+ + + + + +
Ware 2022b	73	1.0	0.5	74	1.1	0.8	8.5	-0.1 (-0.3, 0.2)	+ + + + + +
Breton 2020	78	0.4	0.6	23	0.4	0.3	7.9	-0.0 (-0.3, 0.2)	+ + + + + +
Christensen 2024	20	0.4	0.4	20	0.4	0.5	7.0	0.0 (-0.3, 0.3)	+ + + + ? ?
Boughton 2022b	20	0.2	0.1	17	0.2	0.1	11.8	0.0 (-0.1, 0.1)	+ + + + + +
Wadwa 2023	68	0.6	0.5	34	0.5	0.5	8.8	0.1 (-0.1, 0.3)	+ + + + + +
**Overall**								-0.2 (-0.3, -0.0)	

Heterogeneity: $\tau^2 = 0.03$, $I^2 = 85.31\%$, $H^2 = 6.81$
Test of $\theta_i = \theta_j$: Q(10) = 68.09, p = 0.00
Test of $\theta = 0$: z = -2.61, p = 0.01

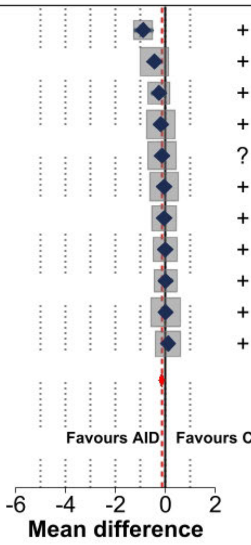

Random-effects DerSimonian–Laird model
Sorted by: _meta_es

Time below range <54 mg/dl - AHCL vs. PLGM

Study	AHCL N	AHCL Mean	AHCL SD	PLGM N	PLGM Mean	PLGM SD	Weight %	Mean difference (95% CI)	Risk of Bias A B C D E F
Benhamou 2021	5	0.2	0.2	5	1.3	0.2	20.7	-1.1 (-1.3, -0.9)	? + + + + ?
Benhalima 2024	48	1.0	1.5	45	1.7	2.0	10.4	-0.7 (-1.4, 0.0)	+ + + + + +
Brown 2020	54	0.3	0.3	55	0.3	0.3	23.2	-0.0 (-0.1, 0.1)	+ + + + + +
Collyns 2021	59	0.5	0.5	59	0.5	0.5	22.1	0.0 (-0.2, 0.2)	? + + + + ?
Kudva 2025	81	0.3	0.2	79	0.3	0.2	23.7	0.0 (-0.0, 0.1)	+ + + + + +
**Overall**								-0.3 (-0.6, 0.0)	

Heterogeneity: $\tau^2 = 0.10$, $I^2 = 94.79\%$, $H^2 = 19.21$
Test of $\theta_i = \theta_j$: Q(4) = 76.83, p = 0.00
Test of $\theta = 0$: z = -1.92, p = 0.05

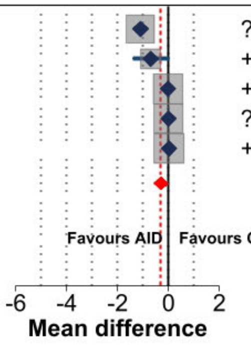

Random-effects DerSimonian–Laird model
Sorted by: _meta_es

Time below range <54 mg/dl - AHCL vs. HCL

Study	AHCL N	AHCL Mean	AHCL SD	HCL N	HCL Mean	HCL SD	Weight %	Mean difference (95% CI)	Risk of Bias A B C D E F
Russell 2022	219	0.5	0.5	107	0.6	1.1	21.5	-0.1 (-0.3, 0.0)	+ + + + + +
Bergenstal 2021	112	0.5	0.3	112	0.5	0.3	78.5	-0.0 (-0.1, 0.0)	+ + + + + +
**Overall**								-0.1 (-0.1, 0.0)	

Heterogeneity: $\tau^2 = 0.00$, $I^2 = 0.00\%$, $H^2 = 1.00$
Test of $\theta_i = \theta_j$: Q(1) = 0.74, p = 0.39
Test of $\theta = 0$: z = -1.44, p = 0.15

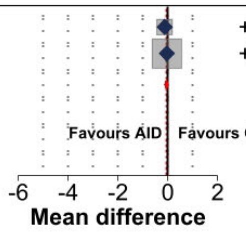

Random-effects DerSimonian–Laird model
Sorted by: _meta_es

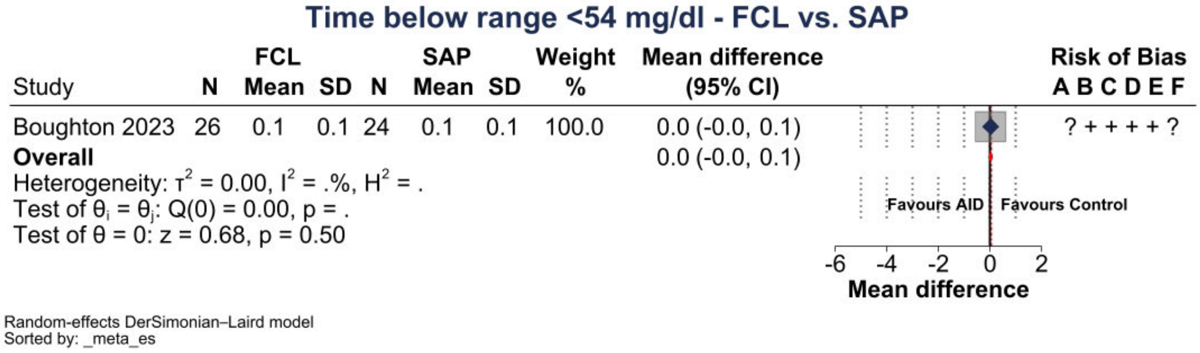

TBR <54 mg/dl – FCL vs. MDI/CSII/PLGM/HCL/AHCL: no study

4.7 Pairwise meta-analyses HbA1c

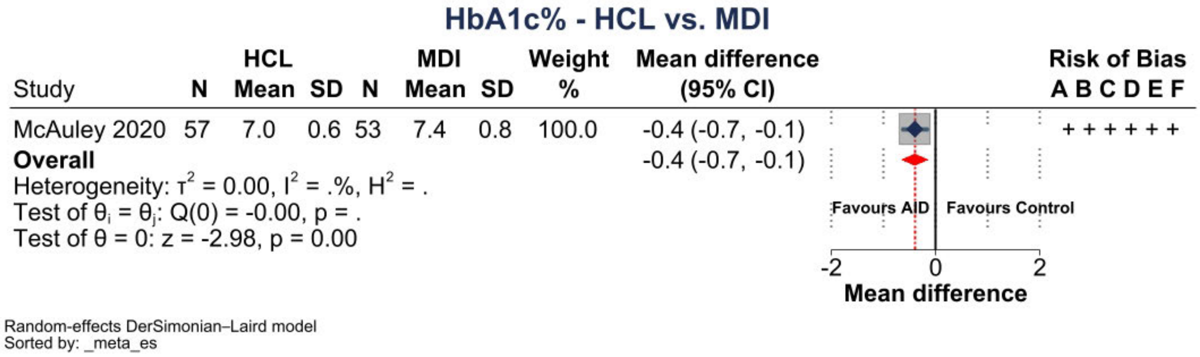

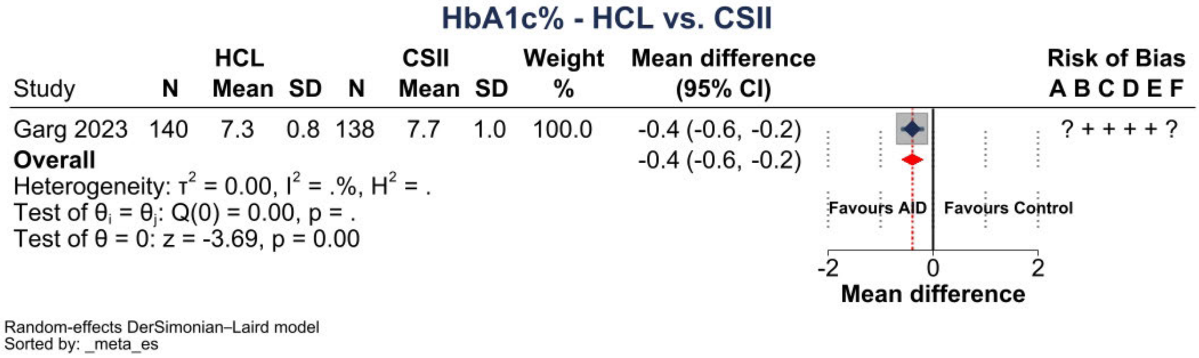

HbA1c% - HCL vs. SAP

Study	HCL N	HCL Mean	HCL SD	SAP N	SAP Mean	SAP SD	Weight %	Mean difference (95% CI)	Risk of Bias A B C D E F
Burnside 2022 ≥16y	22	6.8	0.8	26	7.5	0.8	9.9	-0.7 (-1.2, -0.2)	+ + + + + +
Burnside 2022 <16y	20	7.0	0.9	27	7.6	1.0	8.7	-0.6 (-1.2, -0.0)	+ + + + + +
Tauschmann 2018	46	7.4	0.6	40	7.7	0.5	12.3	-0.3 (-0.5, -0.1)	+ + + + + +
Thabit 2015	32	7.3	0.8	33	7.6	1.1	9.7	-0.3 (-0.8, 0.2)	+ + + + + +
McAuley 2022	30	7.3	0.3	30	7.5	0.7	12.1	-0.2 (-0.5, 0.0)	+ + + + + +
Kovatchev 2020	63	7.2	0.7	59	7.2	0.9	11.8	0.0 (-0.3, 0.3)	+ + + + + +
Pinsker 2022	35	6.8	0.8	35	6.8	0.9	10.5	0.0 (-0.4, 0.4)	? + + + + ?
Kim 2024	53	6.4	0.7	51	6.3	0.7	12.0	0.1 (-0.2, 0.4)	+ + + + ? ?
Polsky 2024	11	6.7	0.2	12	6.2	0.2	13.0	0.5 (0.3, 0.7)	? + + + + ?
**Overall**								-0.1 (-0.4, 0.1)	

Heterogeneity: $\tau^2 = 0.14$, $I^2 = 86.60\%$, $H^2 = 7.46$

Test of $\theta_i = \theta_j$: Q(8) = 59.71, p = 0.00

Test of $\theta = 0$: z = -0.98, p = 0.33

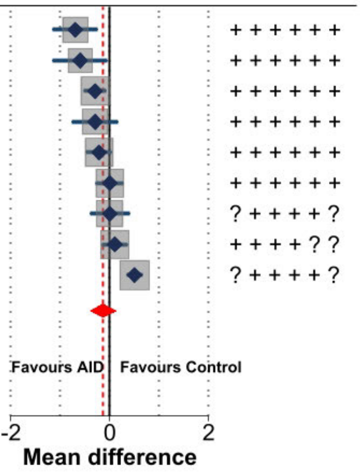

Random-effects DerSimonian–Laird model
Sorted by: _meta_es

HbA1c% - HCL vs. PLGM

Study	HCL N	HCL Mean	HCL SD	PLGM N	PLGM Mean	PLGM SD	Weight %	Mean difference (95% CI)	Risk of Bias A B C D E F
Abraham 2021	58	7.5	1.1	63	7.6	0.8	100.0	-0.1 (-0.4, 0.2)	+ + + + + +
**Overall**								-0.1 (-0.4, 0.2)	

Heterogeneity: $\tau^2 = 0.00$, $I^2 = .\%$, $H^2 = .$

Test of $\theta_i = \theta_j$: Q(0) = 0.00, p = .

Test of $\theta = 0$: z = -0.58, p = 0.57

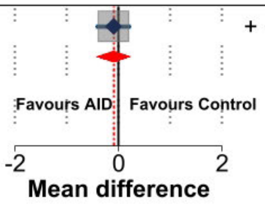

Random-effects DerSimonian–Laird model
Sorted by: _meta_es

HbA1c% - AHCL vs. MDI

Study	AHCL N	AHCL Mean	AHCL SD	MDI N	MDI Mean	MDI SD	Weight %	Mean difference (95% CI)	Risk of Bias A B C D E F
Boucsein 2024	37	8.1	1.8	43	10.6	1.8	16.7	-2.5 (-3.3, -1.7)	+ + + + + +
Choudhary 2022	36	7.3	0.6	38	8.9	0.8	21.2	-1.6 (-1.9, -1.3)	+ + + + + +
Boughton 2022a	47	6.9	1.0	36	8.0	1.4	19.5	-1.1 (-1.6, -0.6)	+ + + + + +
Matejko 2022	20	6.7	0.4	17	7.4	0.8	20.6	-0.7 (-1.1, -0.3)	+ ? + + + ?
Lee 2023	59	6.0	0.5	61	6.4	0.5	22.0	-0.4 (-0.6, -0.2)	+ + + + + +
**Overall**								-1.2 (-1.8, -0.6)	

Heterogeneity: $\tau^2 = 0.48$, $I^2 = 93.51\%$, $H^2 = 15.42$

Test of $\theta_i = \theta_j$: Q(4) = 61.67, p = 0.00

Test of $\theta = 0$: z = -3.66, p = 0.00

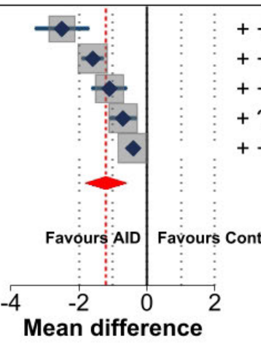

Random-effects DerSimonian–Laird model
Sorted by: _meta_es

HbA1c% - AHCL vs. CSII

Study	AHCL N	AHCL Mean	AHCL SD	CSII N	CSII Mean	CSII SD	Weight %	Mean difference (95% CI)	Risk of Bias A B C D E F
McVean 2023	59	6.5	0.7	48	7.1	1.3	100.0	-0.6 (-1.0, -0.2)	+ + + + + +
**Overall**								-0.6 (-1.0, -0.2)	

Heterogeneity: $\tau^2 = 0.00$, $I^2 = .\%$, $H^2 = .$
Test of $\theta_i = \theta_j$: $Q(0) = 0.00$, $p = .$
Test of $\theta = 0$: $z = -3.05$, $p = 0.00$

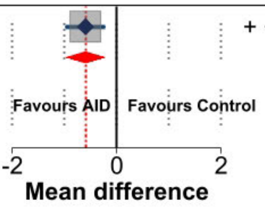

Random-effects DerSimonian–Laird model
Sorted by: _meta_es

HbA1c% - AHCL vs. SAP

Study	AHCL N	AHCL Mean	AHCL SD	SAP N	SAP Mean	SAP SD	Weight %	Mean difference (95% CI)	Risk of Bias A B C D E F
Abraham 2024	19	8.8	1.1	19	9.9	1.2	4.5	-1.1 (-1.8, -0.4)	+ + + + + +
Christensen 2024	20	7.3	0.4	20	8.3	0.4	10.9	-1.0 (-1.2, -0.8)	+ + + + ? ?
Breton 2020	78	7.0	0.8	23	7.6	0.9	8.6	-0.6 (-1.0, -0.2)	+ + + + + +
Renard 2024	132	7.3	0.8	62	7.8	0.8	11.1	-0.6 (-0.8, -0.4)	? + + + ? ?
Wadwa 2023	68	7.0	0.8	34	7.5	0.9	9.3	-0.5 (-0.8, -0.2)	+ + + + + +
Ware 2022a	57	7.6	1.1	62	8.1	0.8	9.3	-0.5 (-0.8, -0.2)	+ + + + + +
Ware 2022b	73	6.6	0.6	74	7.0	0.7	11.5	-0.4 (-0.6, -0.2)	+ + + + + +
Brown 2019	112	7.1	0.8	56	7.4	0.9	10.6	-0.3 (-0.6, -0.1)	+ + + + + +
Boughton 2022b	20	6.7	0.7	17	6.9	0.9	6.7	-0.2 (-0.7, 0.3)	+ + + + + +
Kudva 2025	81	6.9	0.7	78	7.0	0.8	11.1	-0.1 (-0.3, 0.1)	+ + + + + +
Renard 2023	49	7.0	0.7	22	6.9	1.6	6.5	0.1 (-0.4, 0.6)	+ ? + + + ?
**Overall**								-0.5 (-0.7, -0.3)	

Heterogeneity: $\tau^2 = 0.07$, $I^2 = 74.01\%$, $H^2 = 3.85$
Test of $\theta_i = \theta_j$: $Q(10) = 38.48$, $p = 0.00$
Test of $\theta = 0$: $z = -4.84$, $p = 0.00$

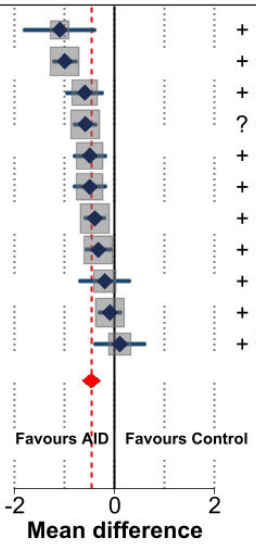

Random-effects DerSimonian–Laird model
Sorted by: _meta_es

HbA1c% - AHCL vs. PLGM

Study	AHCL N	AHCL Mean	AHCL SD	PLGM N	PLGM Mean	PLGM SD	Weight %	Mean difference (95% CI)	Risk of Bias A B C D E F
Brown 2020	54	7.2	0.8	55	7.5	1.1	25.2	-0.4 (-0.7, 0.0)	+ + + + + +
Kudva 2025	81	6.9	0.7	79	7.1	0.8	37.0	-0.2 (-0.4, 0.0)	+ + + + + +
Benhalima 2024	48	6.2	0.6	45	6.1	0.5	37.8	0.1 (-0.1, 0.3)	+ + + + + +
**Overall**								-0.1 (-0.4, 0.1)	

Heterogeneity: $\tau^2 = 0.03$, $I^2 = 63.51\%$, $H^2 = 2.74$
Test of $\theta_i = \theta_j$: $Q(2) = 5.48$, $p = 0.06$
Test of $\theta = 0$: $z = -0.95$, $p = 0.34$

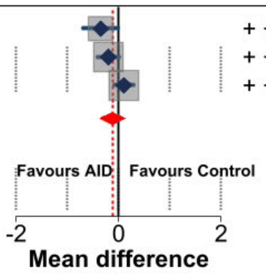

Random-effects DerSimonian–Laird model
Sorted by: _meta_es

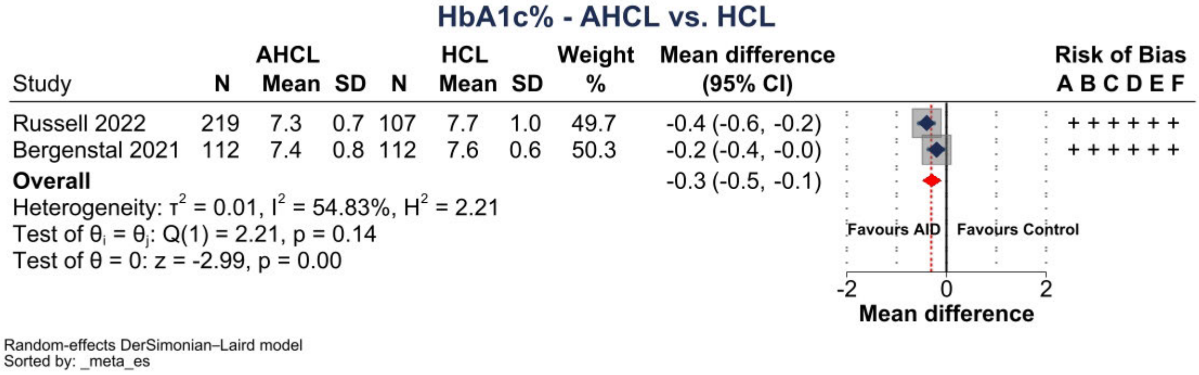

HbA1c – FCL vs. MDI/CSII/SAP/PLGM/HCL/AHCL: no study

5 Supplement to sensitivity analysis

5.1 Sensitivity analyses TIR

Sensitivity Analyses - time in range 70-180 mg/dl - HCL vs. CSII

Study	K	Mean difference (95% CI)	p-value
**Intervention duration**			
<12 weeks	3	12.6 (8.0, 17.2)	0.000
≥12 weeks	1	12.0 (8.9, 15.1)	0.000
Test of group differences: $Q_b(1) = 0.05$, $p = 0.82$			
**Study design**			
parallel	1	12.0 (8.9, 15.1)	0.000
crossover	3	12.6 (8.0, 17.2)	0.000
Test of group differences: $Q_b(1) = 0.05$, $p = 0.82$			
**Sample size**			
<100 participants	3	12.6 (8.0, 17.2)	0.000
≥100 participants	1	12.0 (8.9, 15.1)	0.000
Test of group differences: $Q_b(1) = 0.05$, $p = 0.82$			
**Study population**			
men and women	4	12.0 (9.7, 14.2)	0.000
Test of group differences: $Q_b(0) = 0.00$, p = .			
**Age**			
≥18 years	2	13.6 (6.0, 21.3)	0.000
mixed population	2	12.0 (9.1, 14.9)	0.000
Test of group differences: $Q_b(1) = 0.16$, $p = 0.69$			
**Diabetes duration**			
longer-term T1D	4	12.0 (9.7, 14.2)	0.000
Test of group differences: $Q_b(0) = 0.00$, p = .			
**HbA1c**			
≤7.5%	2	13.6 (6.0, 21.3)	0.000
>7.5%	2	12.0 (9.1, 14.9)	0.000
Test of group differences: $Q_b(1) = 0.16$, $p = 0.69$			
**Risk of bias**			
low	1	10.6 (6.8, 14.4)	0.000
some concerns	3	12.8 (9.8, 15.7)	0.000
Test of group differences: $Q_b(1) = 0.78$, $p = 0.38$			
**Overall**		12.0 (9.7, 14.2)	0.000
Heterogeneity: $\tau^2 = 0.00$, $I^2 = 0.00\%$, $H^2 = 1.00$			
Test of $\theta_i = \theta_j$: $Q(3) = 2.82$, $p = 0.42$			

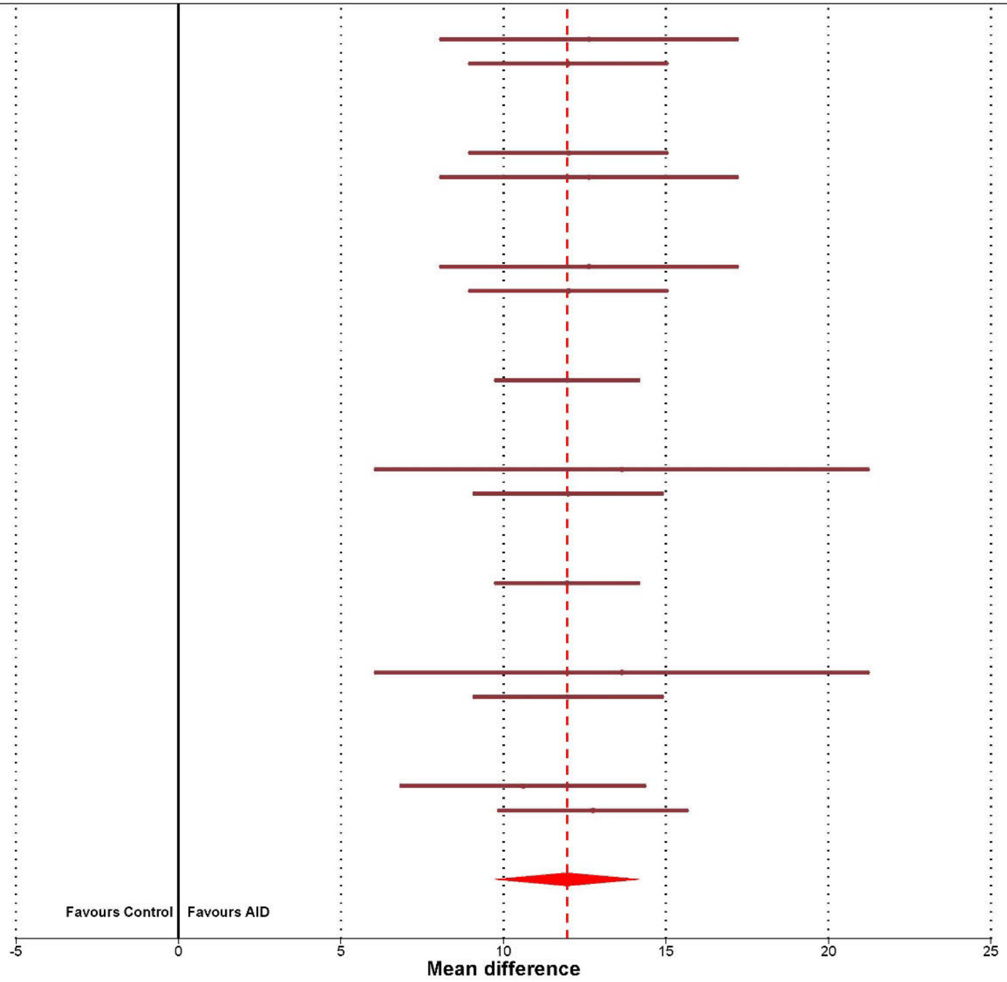

Random-effects DerSimonian–Laird model

Sensitivity Analyses - time in range 70-180 mg/dl - HCL vs. SAP

Study	K	Mean difference (95% CI)	p-value
**Intervention duration**			
<12 weeks	2	18.7 (12.6, 24.9)	0.000
≥12 weeks	10	7.2 (3.0, 11.3)	0.001
Test of group differences: $Q_b(1) = 9.33$, $p = 0.00$			
**Study design**			
parallel	8	8.7 (3.2, 14.3)	0.002
crossover	4	8.9 (3.5, 14.3)	0.001
Test of group differences: $Q_b(1) = 0.00$, $p = 0.96$			
**Sample size**			
<100 participants	10	9.6 (4.5, 14.7)	0.000
≥100 participants	2	6.0 (2.6, 9.3)	0.000
Test of group differences: $Q_b(1) = 1.39$, $p = 0.24$			
**Study population**			
only women men	2	-1.2 (-3.1, 0.7)	0.205
and women	10	10.4 (7.2, 13.6)	0.000
Test of group differences: $Q_b(1) = 38.02$, $p = 0.00$			
**Age**			
<18 years	2	16.8 (10.5, 23.2)	0.000
≥18 years	6	3.8 (-0.3, 8.0)	0.068
mixed population	4	12.7 (7.7, 17.6)	0.000
Test of group differences: $Q_b(2) = 13.87$, $p = 0.00$			
**Diabetes duration**			
longer-term T1D	12	8.8 (4.7, 13.0)	0.000
Test of group differences: $Q_b(0) = 0.00$, p = .			
**Hba1c**			
≤7.5%	7	6.1 (1.1, 11.2)	0.018
>7.5%	5	11.9 (7.7, 16.1)	0.000
Test of group differences: $Q_b(1) = 2.90$, $p = 0.09$			
**Risk of bias**			
low	7	9.4 (6.0, 12.8)	0.000
some concerns	5	8.0 (0.4, 15.5)	0.040
Test of group differences: $Q_b(1) = 0.11$, $p = 0.74$			
**Overall**		8.8 (4.7, 13.0)	0.000
Heterogeneity: $\tau^2 = 44.18$, $I^2 = 88.16\%$, $H^2 = 8.45$			
Test of $\theta_i = \theta_j$: $Q(11) = 92.94$, $p = 0.00$			

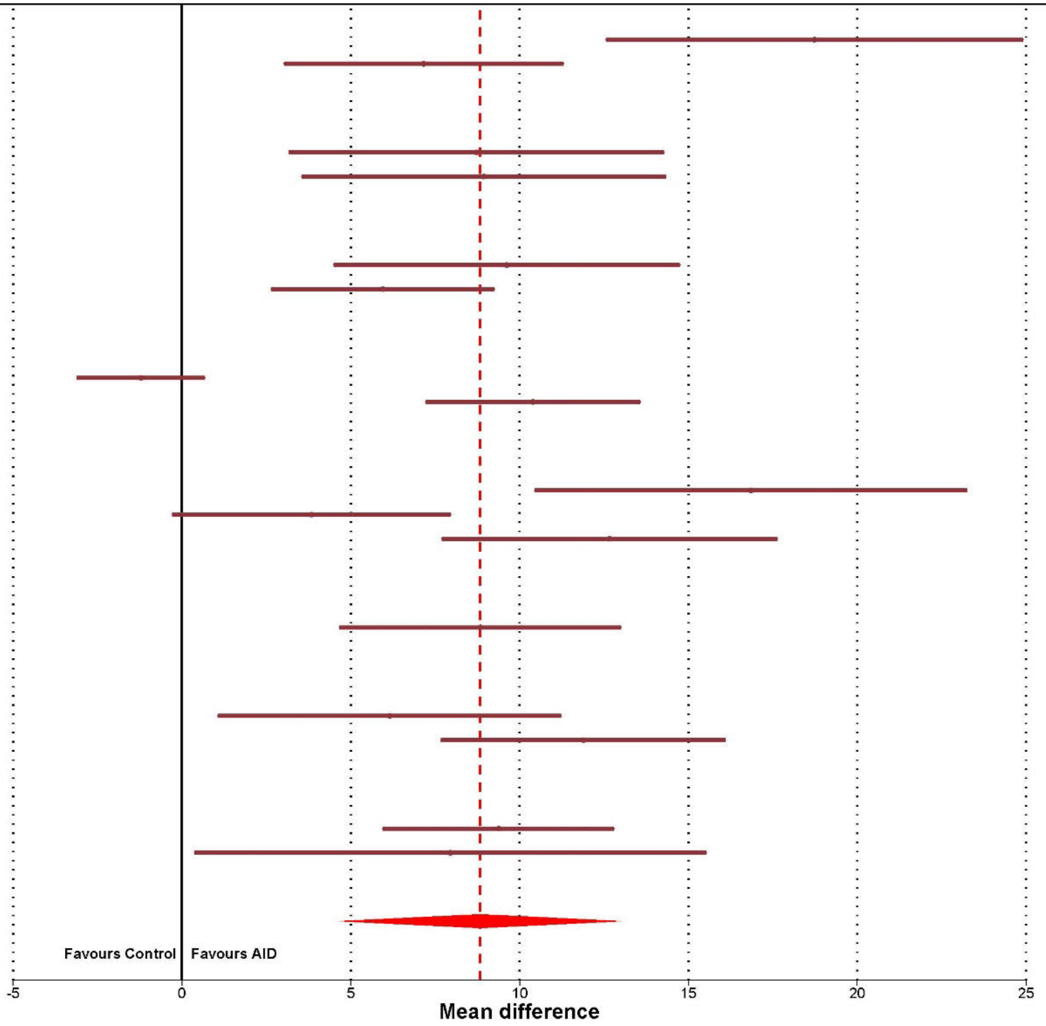

Random-effects DerSimonian–Laird model

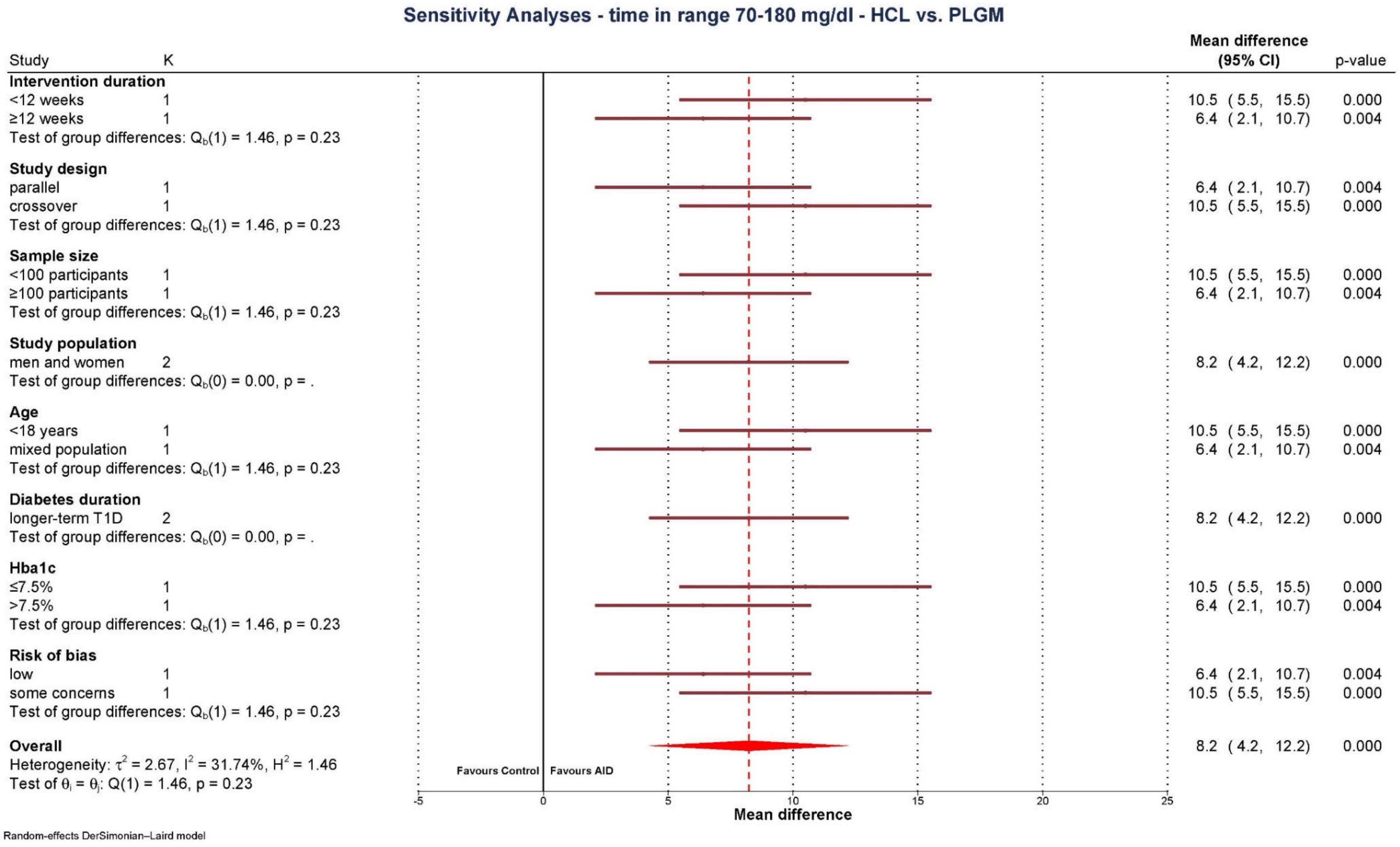
Sensitivity Analyses - time in range 70-180 mg/dl - HCL vs. PLGM
Study K
Mean difference (95% CI)
p-value
Intervention duration
<12 weeks 1 10.5 (5.5, 15.5) 0.000
≥12 weeks 1 6.4 (2.1, 10.7) 0.004
Test of group differences: $Q_b(1)$ = 1.46, p = 0.23
Study design
parallel 1 6.4 (2.1, 10.7) 0.004
crossover 1 10.5 (5.5, 15.5) 0.000
Test of group differences: $Q_b(1)$ = 1.46, p = 0.23
Sample size
<100 participants 1 10.5 (5.5, 15.5) 0.000
≥100 participants 1 6.4 (2.1, 10.7) 0.004
Test of group differences: $Q_b(1)$ = 1.46, p = 0.23
Study population
men and women 2 8.2 (4.2, 12.2) 0.000
Test of group differences: $Q_b(0)$ = 0.00, p = .
Age
<18 years 1 10.5 (5.5, 15.5) 0.000
mixed population 1 6.4 (2.1, 10.7) 0.004
Test of group differences: $Q_b(1)$ = 1.46, p = 0.23
Diabetes duration
longer-term T1D 2 8.2 (4.2, 12.2) 0.000
Test of group differences: $Q_b(0)$ = 0.00, p = .
Hba1c
≤7.5% 1 10.5 (5.5, 15.5) 0.000
>7.5% 1 6.4 (2.1, 10.7) 0.004
Test of group differences: $Q_b(1)$ = 1.46, p = 0.23
Risk of bias
low 1 6.4 (2.1, 10.7) 0.004
some concerns 1 10.5 (5.5, 15.5) 0.000
Test of group differences: $Q_b(1)$ = 1.46, p = 0.23
Overall 8.2 (4.2, 12.2) 0.000
Heterogeneity: τ^2 = 2.67, I^2 = 31.74%, H^2 = 1.46
Test of $\theta_i = \theta_j$: Q(1) = 1.46, p = 0.23
Favours Control
Favours AID
-5
0
5
10
15
20
25
Mean difference
Random-effects DerSimonian–Laird model

Sensitivity Analyses- time in range 70-180 mg/dl - AHCL vs. MDI

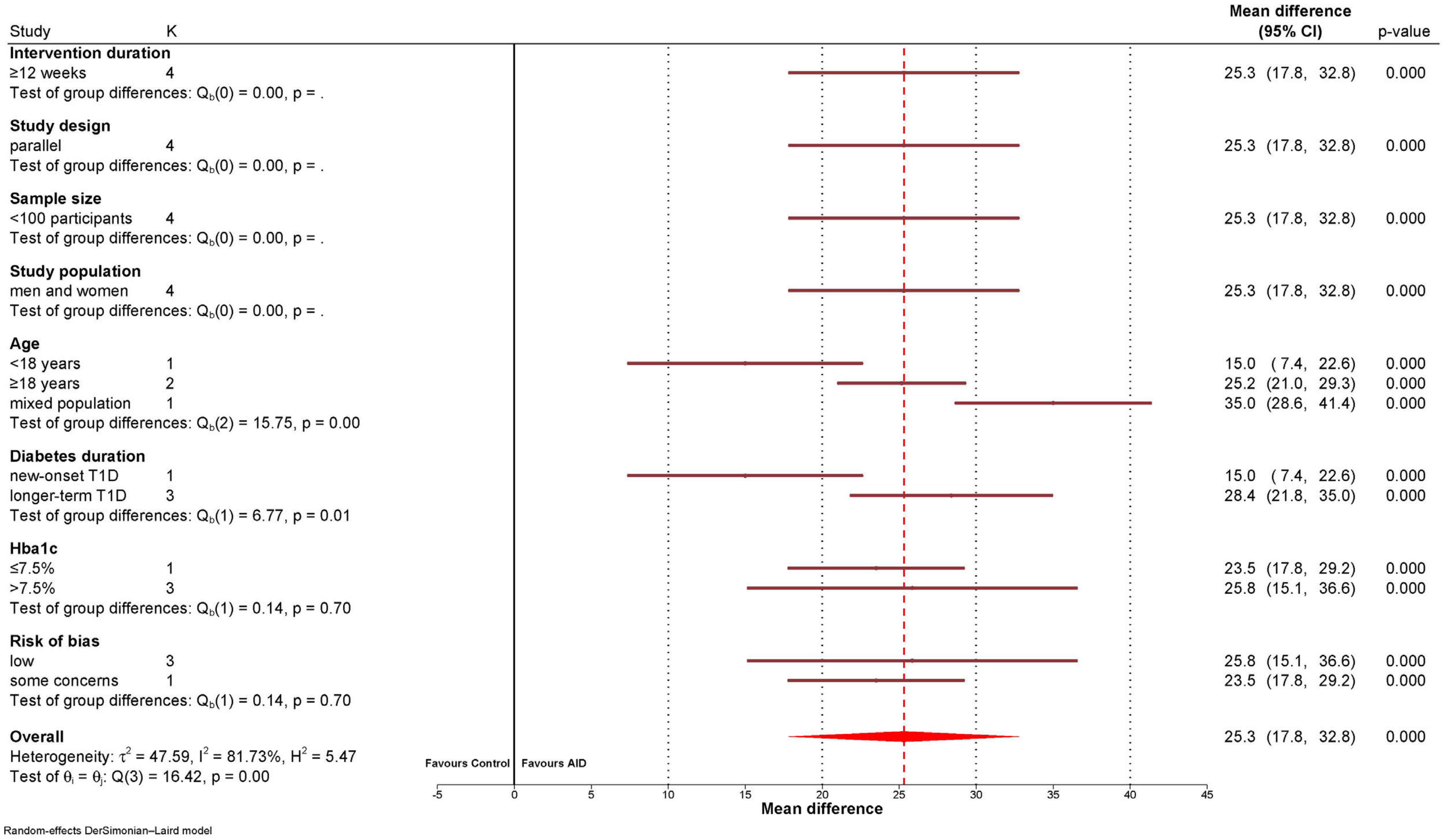

Sensitivity Analyses- time in range 70-180 mg/dl - AHCL vs. SAP

Study	K	Mean difference (95% CI)	p-value
**Intervention duration**			
≥12 weeks	4	25.3 (17.8, 32.8)	0.000
Test of group differences: $Q_b(0) = 0.00$, p = .			
**Study design**			
parallel	4	25.3 (17.8, 32.8)	0.000
Test of group differences: $Q_b(0) = 0.00$, p = .			
**Sample size**			
<100 participants	4	25.3 (17.8, 32.8)	0.000
Test of group differences: $Q_b(0) = 0.00$, p = .			
**Study population**			
men and women	4	25.3 (17.8, 32.8)	0.000
Test of group differences: $Q_b(0) = 0.00$, p = .			
**Age**			
<18 years	1	15.0 (7.4, 22.6)	0.000
≥18 years	2	25.2 (21.0, 29.3)	0.000
mixed population	1	35.0 (28.6, 41.4)	0.000
Test of group differences: $Q_b(2) = 15.75$, p = 0.00			
**Diabetes duration**			
new-onset T1D	1	15.0 (7.4, 22.6)	0.000
longer-term T1D	3	28.4 (21.8, 35.0)	0.000
Test of group differences: $Q_b(1) = 6.77$, p = 0.01			
**Hba1c**			
≤7.5%	1	23.5 (17.8, 29.2)	0.000
>7.5%	3	25.8 (15.1, 36.6)	0.000
Test of group differences: $Q_b(1) = 0.14$, p = 0.70			
**Risk of bias**			
low	3	25.8 (15.1, 36.6)	0.000
some concerns	1	23.5 (17.8, 29.2)	0.000
Test of group differences: $Q_b(1) = 0.14$, p = 0.70			
**Overall**		25.3 (17.8, 32.8)	0.000
Heterogeneity: $\tau^2 = 47.59$, $I^2 = 81.73\%$, $H^2 = 5.47$			
Test of $\theta_i = \theta_j$: Q(3) = 16.42, p = 0.00			

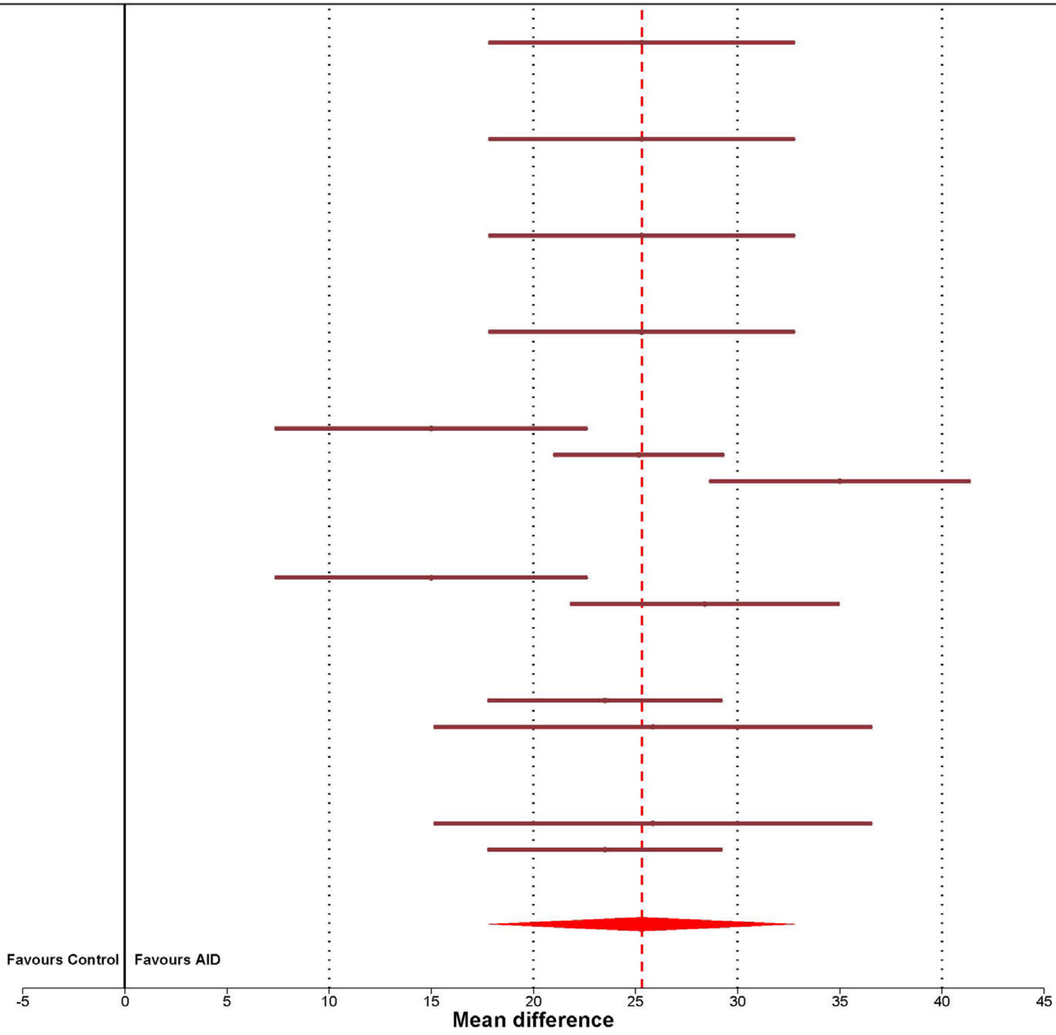

Random-effects DerSimonian–Laird model

Sensitivity Analyses- time in range 70-180 mg/dl - AHCL vs. PLGM

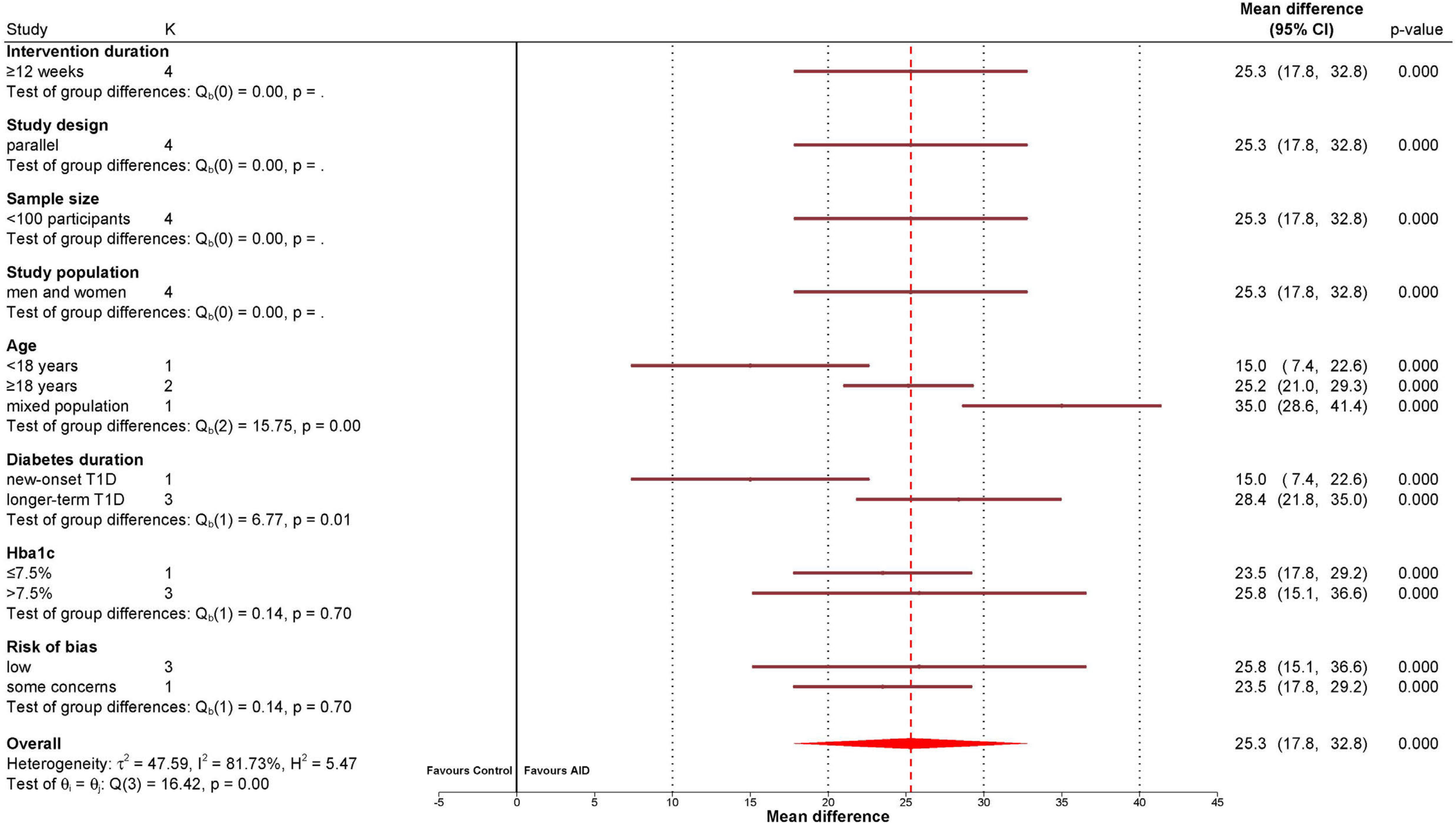

Random-effects DerSimonian–Laird model

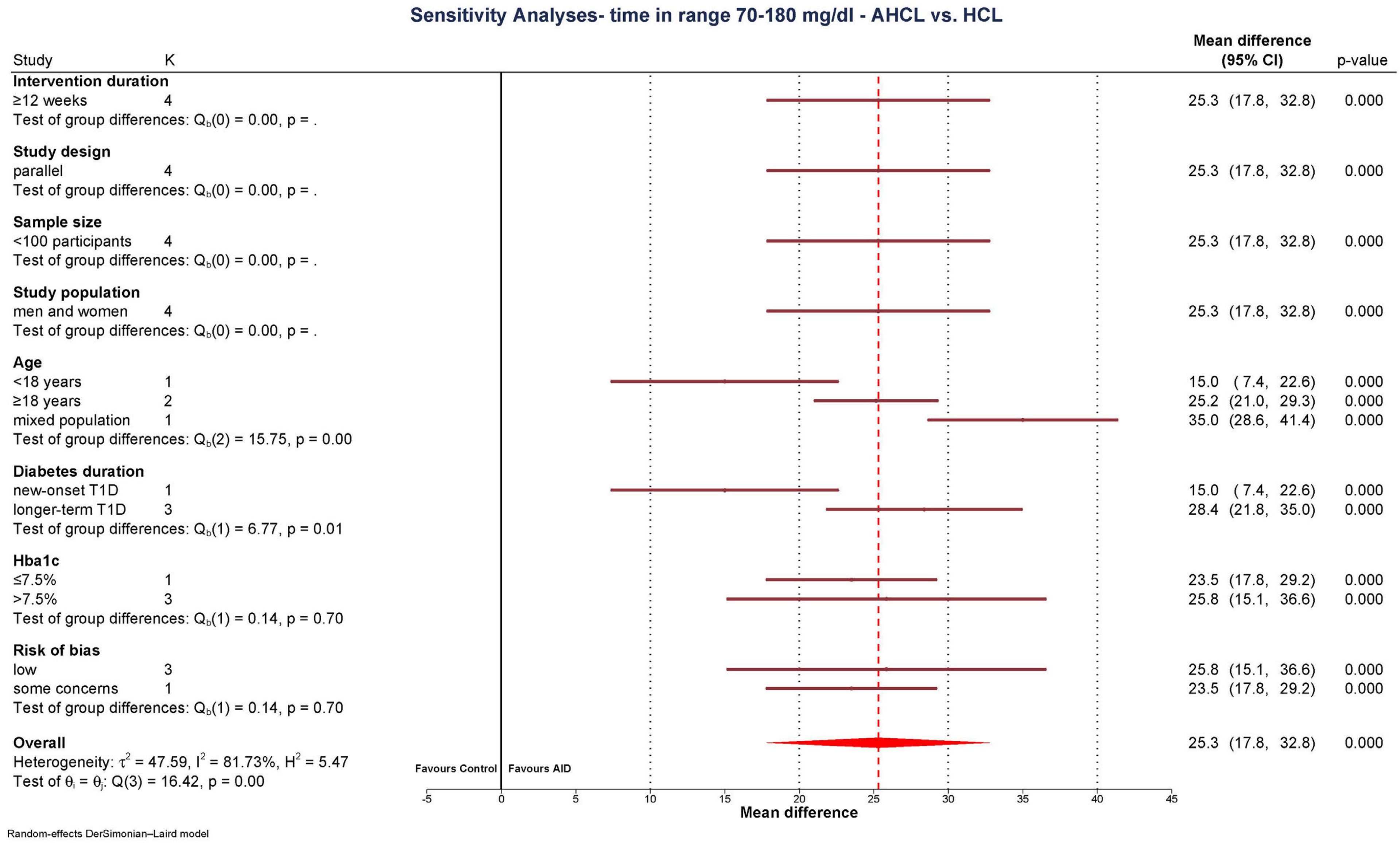
Sensitivity Analyses- time in range 70-180 mg/dl - AHCL vs. HCL
Study K
Mean difference (95% CI) p-value
Intervention duration
≥12 weeks 4 25.3 (17.8, 32.8) 0.000
Test of group differences: $Q_b(0)$ = 0.00, p = .
Study design
parallel 4 25.3 (17.8, 32.8) 0.000
Test of group differences: $Q_b(0)$ = 0.00, p = .
Sample size
<100 participants 4 25.3 (17.8, 32.8) 0.000
Test of group differences: $Q_b(0)$ = 0.00, p = .
Study population
men and women 4 25.3 (17.8, 32.8) 0.000
Test of group differences: $Q_b(0)$ = 0.00, p = .
Age
<18 years 1 15.0 (7.4, 22.6) 0.000
≥18 years 2 25.2 (21.0, 29.3) 0.000
mixed population 1 35.0 (28.6, 41.4) 0.000
Test of group differences: $Q_b(2)$ = 15.75, p = 0.00
Diabetes duration
new-onset T1D 1 15.0 (7.4, 22.6) 0.000
longer-term T1D 3 28.4 (21.8, 35.0) 0.000
Test of group differences: $Q_b(1)$ = 6.77, p = 0.01
Hba1c
≤7.5% 1 23.5 (17.8, 29.2) 0.000
>7.5% 3 25.8 (15.1, 36.6) 0.000
Test of group differences: $Q_b(1)$ = 0.14, p = 0.70
Risk of bias
low 3 25.8 (15.1, 36.6) 0.000
some concerns 1 23.5 (17.8, 29.2) 0.000
Test of group differences: $Q_b(1)$ = 0.14, p = 0.70
Overall 25.3 (17.8, 32.8) 0.000
Heterogeneity: τ^2 = 47.59, I^2 = 81.73%, H^2 = 5.47
Test of $\theta_i = \theta_j$: Q(3) = 16.42, p = 0.00
Favours Control
Favours AID
-5 0 5 10 15 20 25 30 35 40 45
Mean difference
Random-effects DerSimonian–Laird model

5.2 Sensitivity analyses TAR >180 mg/dl

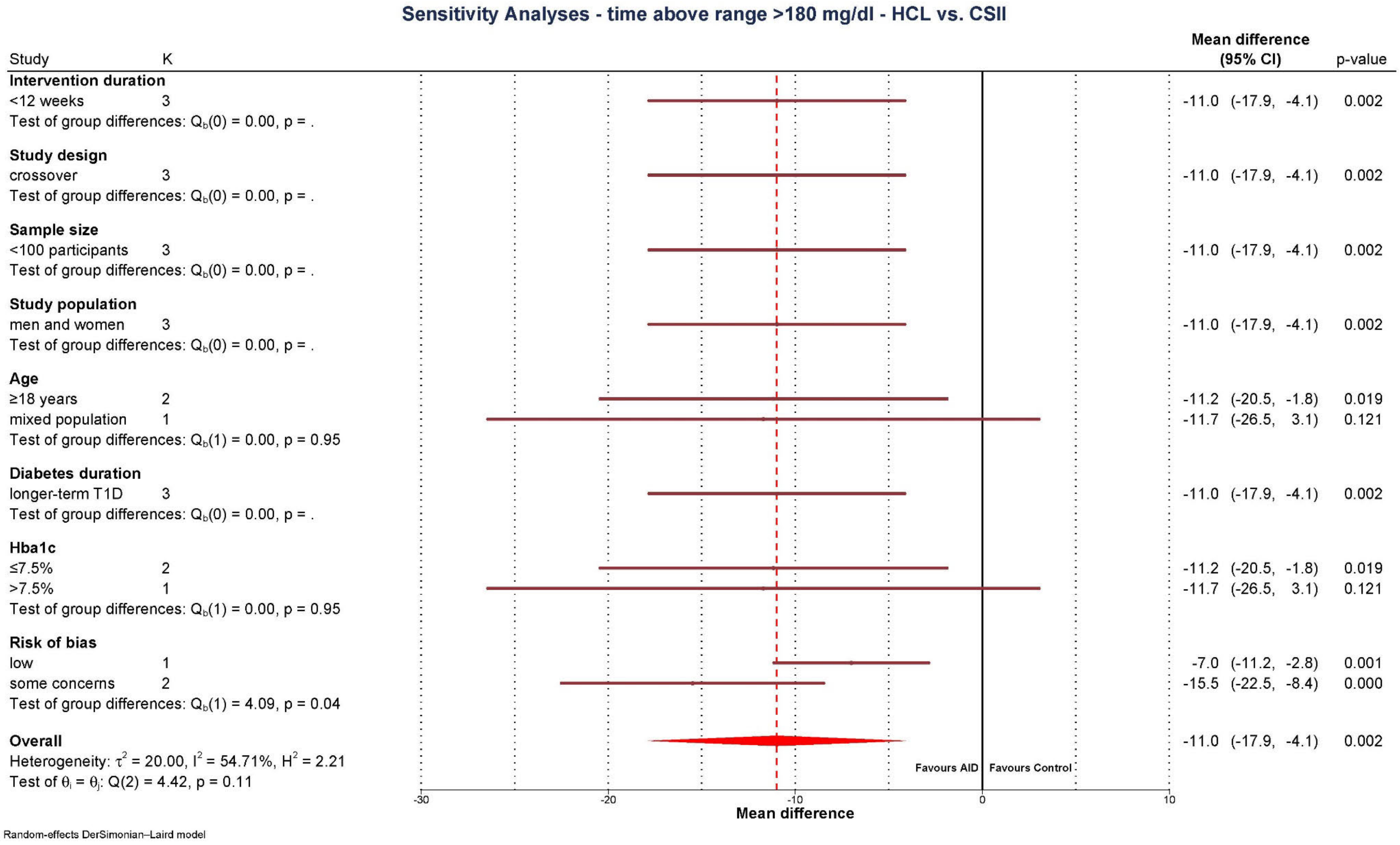

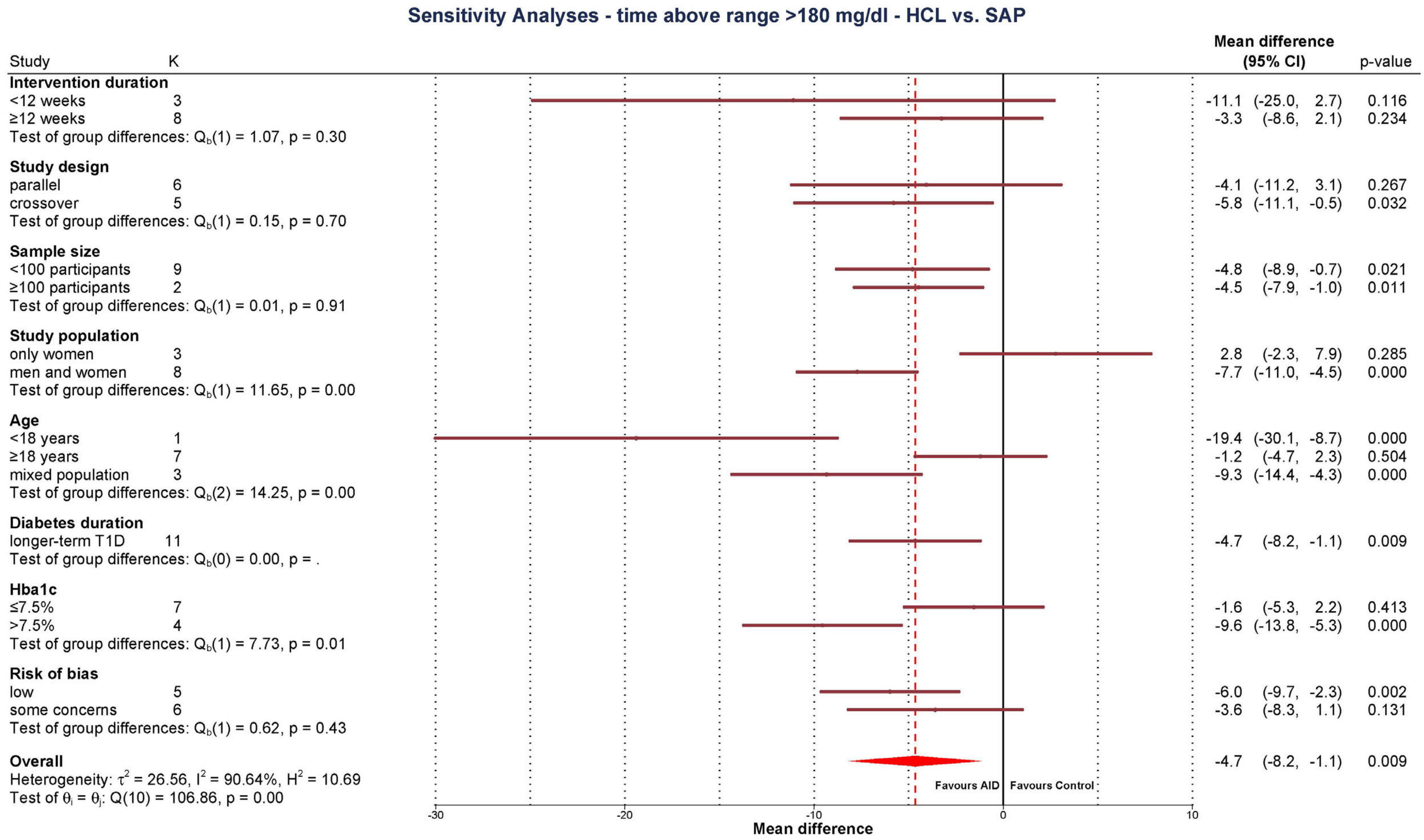

Random-effects DerSimonian–Laird model

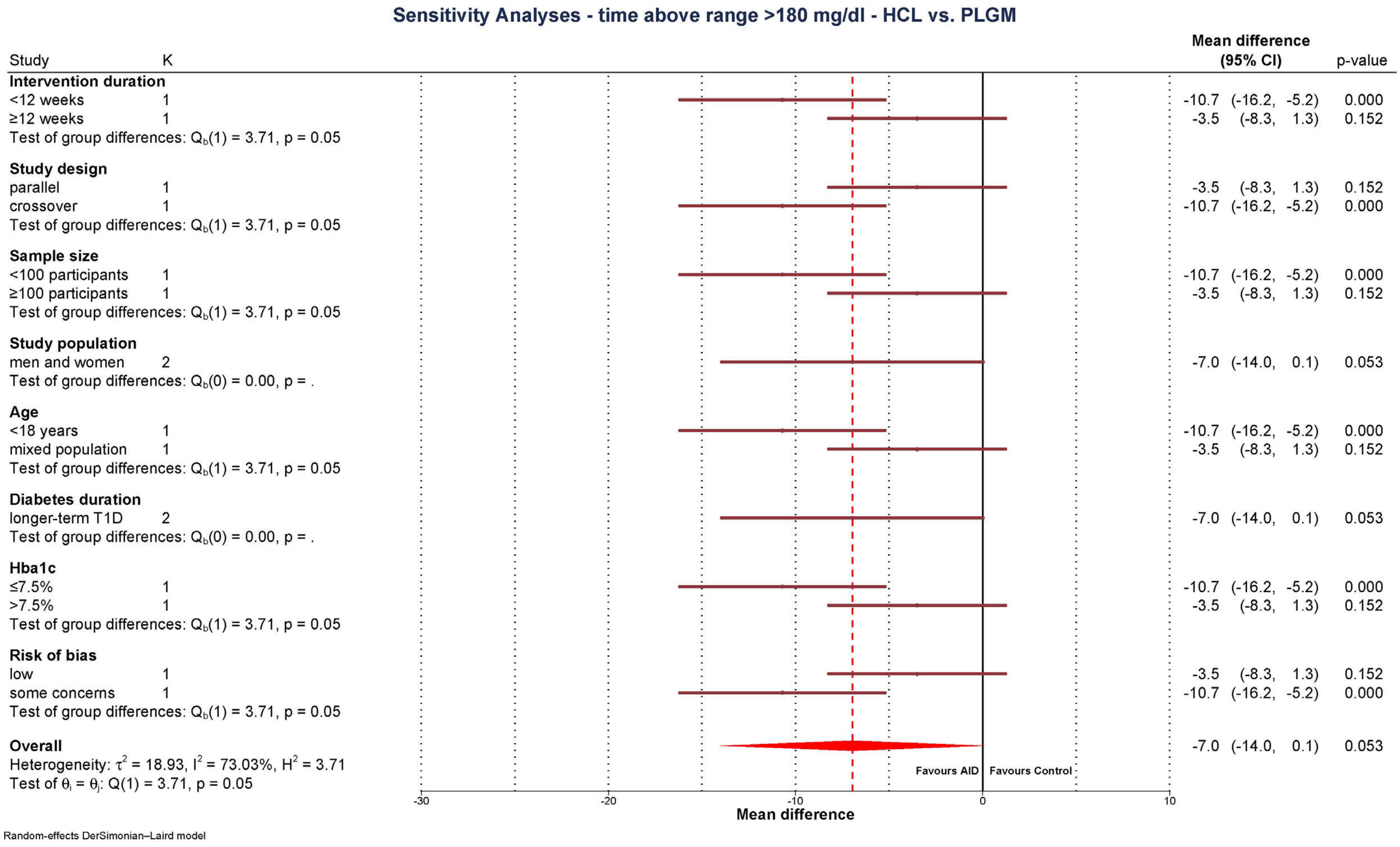
Sensitivity Analyses - time above range >180 mg/dl - HCL vs. PLGM
Study K
Mean difference (95% CI) p-value
Intervention duration
<12 weeks 1 -10.7 (-16.2, -5.2) 0.000
≥12 weeks 1 -3.5 (-8.3, 1.3) 0.152
Test of group differences: $Q_b(1) = 3.71$, p = 0.05
Study design
parallel 1 -3.5 (-8.3, 1.3) 0.152
crossover 1 -10.7 (-16.2, -5.2) 0.000
Test of group differences: $Q_b(1) = 3.71$, p = 0.05
Sample size
<100 participants 1 -10.7 (-16.2, -5.2) 0.000
≥100 participants 1 -3.5 (-8.3, 1.3) 0.152
Test of group differences: $Q_b(1) = 3.71$, p = 0.05
Study population
men and women 2 -7.0 (-14.0, 0.1) 0.053
Test of group differences: $Q_b(0) = 0.00$, p = .
Age
<18 years 1 -10.7 (-16.2, -5.2) 0.000
mixed population 1 -3.5 (-8.3, 1.3) 0.152
Test of group differences: $Q_b(1) = 3.71$, p = 0.05
Diabetes duration
longer-term T1D 2 -7.0 (-14.0, 0.1) 0.053
Test of group differences: $Q_b(0) = 0.00$, p = .
Hba1c
≤7.5% 1 -10.7 (-16.2, -5.2) 0.000
>7.5% 1 -3.5 (-8.3, 1.3) 0.152
Test of group differences: $Q_b(1) = 3.71$, p = 0.05
Risk of bias
low 1 -3.5 (-8.3, 1.3) 0.152
some concerns 1 -10.7 (-16.2, -5.2) 0.000
Test of group differences: $Q_b(1) = 3.71$, p = 0.05
Overall -7.0 (-14.0, 0.1) 0.053
Heterogeneity: $\tau^2 = 18.93$, $I^2 = 73.03\%$, $H^2 = 3.71$
Test of $\theta_i = \theta_j$: Q(1) = 3.71, p = 0.05
Favours AID
Favours Control
-30
-20
-10
0
10
Mean difference
Random-effects DerSimonian–Laird model

Sensitivity Analyses - time above range >180 mg/dl - AHCL vs. MDI

Study	K	Mean difference (95% CI)	p-value
**Intervention duration**			
≥12 weeks	5	-20.3 (-30.7, -10.0)	0.000
Test of group differences: $Q_b(0) = 0.00$, p = .			
**Study design**			
parallel	5	-20.3 (-30.7, -10.0)	0.000
Test of group differences: $Q_b(0) = 0.00$, p = .			
**Sample size**			
<100 participants	4	-24.1 (-30.5, -17.8)	0.000
≥100 participants	1	-6.0 (-9.6, -2.4)	0.001
Test of group differences: $Q_b(1) = 23.72$, p = 0.00			
**Study population**			
only women	1	-6.0 (-9.6, -2.4)	0.001
men and women	4	-24.1 (-30.5, -17.8)	0.000
Test of group differences: $Q_b(1) = 23.72$, p = 0.00			
**Age**			
<18 years	1	-20.0 (-27.0, -13.0)	0.000
≥18 years	3	-16.7 (-29.9, -3.5)	0.013
mixed population	1	-32.0 (-39.2, -24.8)	0.000
Test of group differences: $Q_b(2) = 7.09$, p = 0.03			
**Diabetes duration**			
new-onset T1D	1	-20.0 (-27.0, -13.0)	0.000
longer-term T1D	4	-20.5 (-33.3, -7.6)	0.002
Test of group differences: $Q_b(1) = 0.00$, p = 0.95			
**Hba1c**			
≤7.5%	1	-17.6 (-24.4, -10.8)	0.000
>7.5%	4	-21.1 (-34.2, -8.0)	0.002
Test of group differences: $Q_b(1) = 0.21$, p = 0.64			
**Risk of bias**			
low	4	-21.1 (-34.2, -8.0)	0.002
some concerns	1	-17.6 (-24.4, -10.8)	0.000
Test of group differences: $Q_b(1) = 0.21$, p = 0.64			
**Overall**		-20.3 (-30.7, -10.0)	0.000
Heterogeneity: $\tau^2 = 129.61$, $I^2 = 93.43\%$, $H^2 = 15.22$			
Test of $\theta_i = \theta_j$: Q(4) = 60.87, p = 0.00			

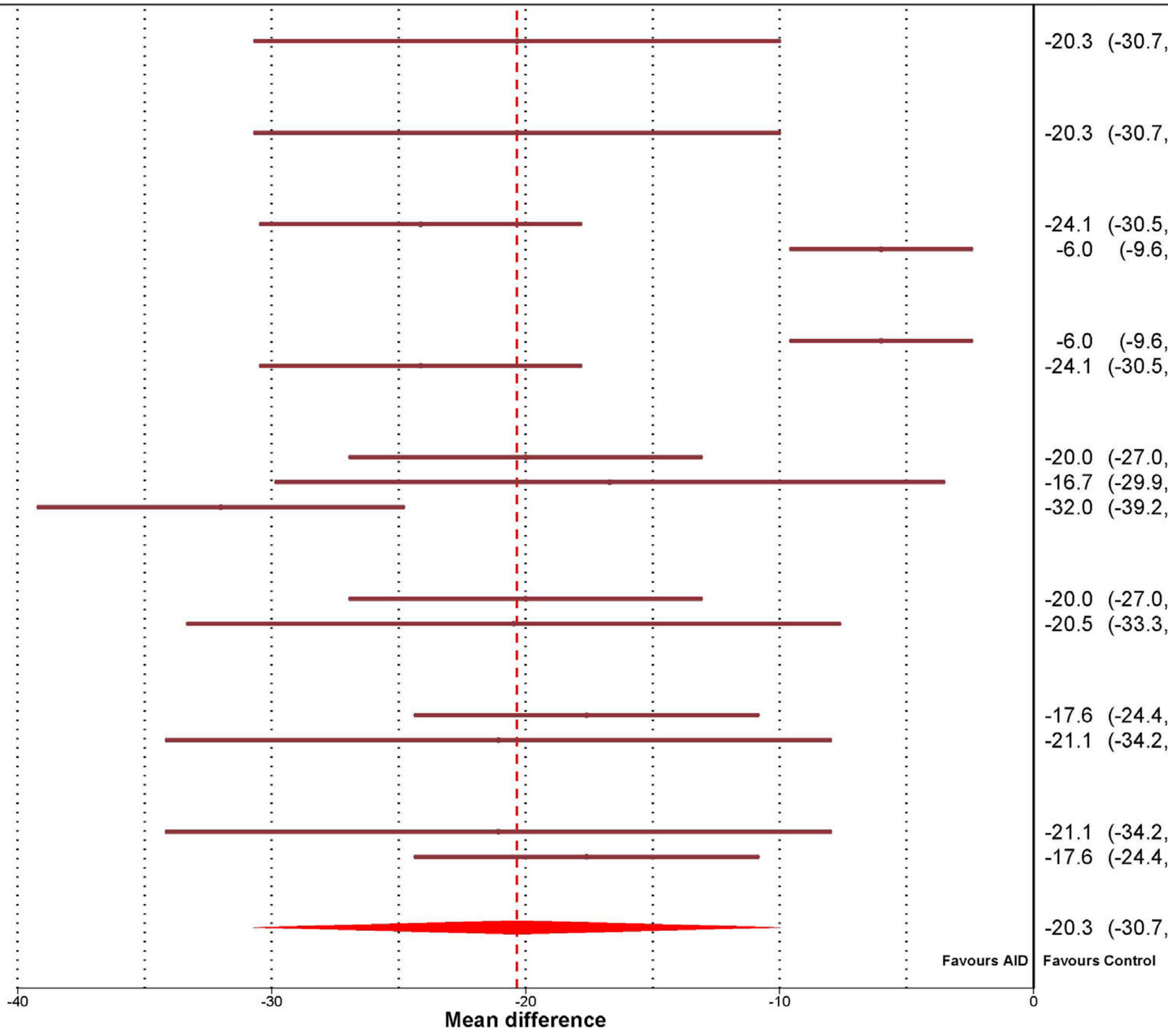

Random-effects DerSimonian–Laird model

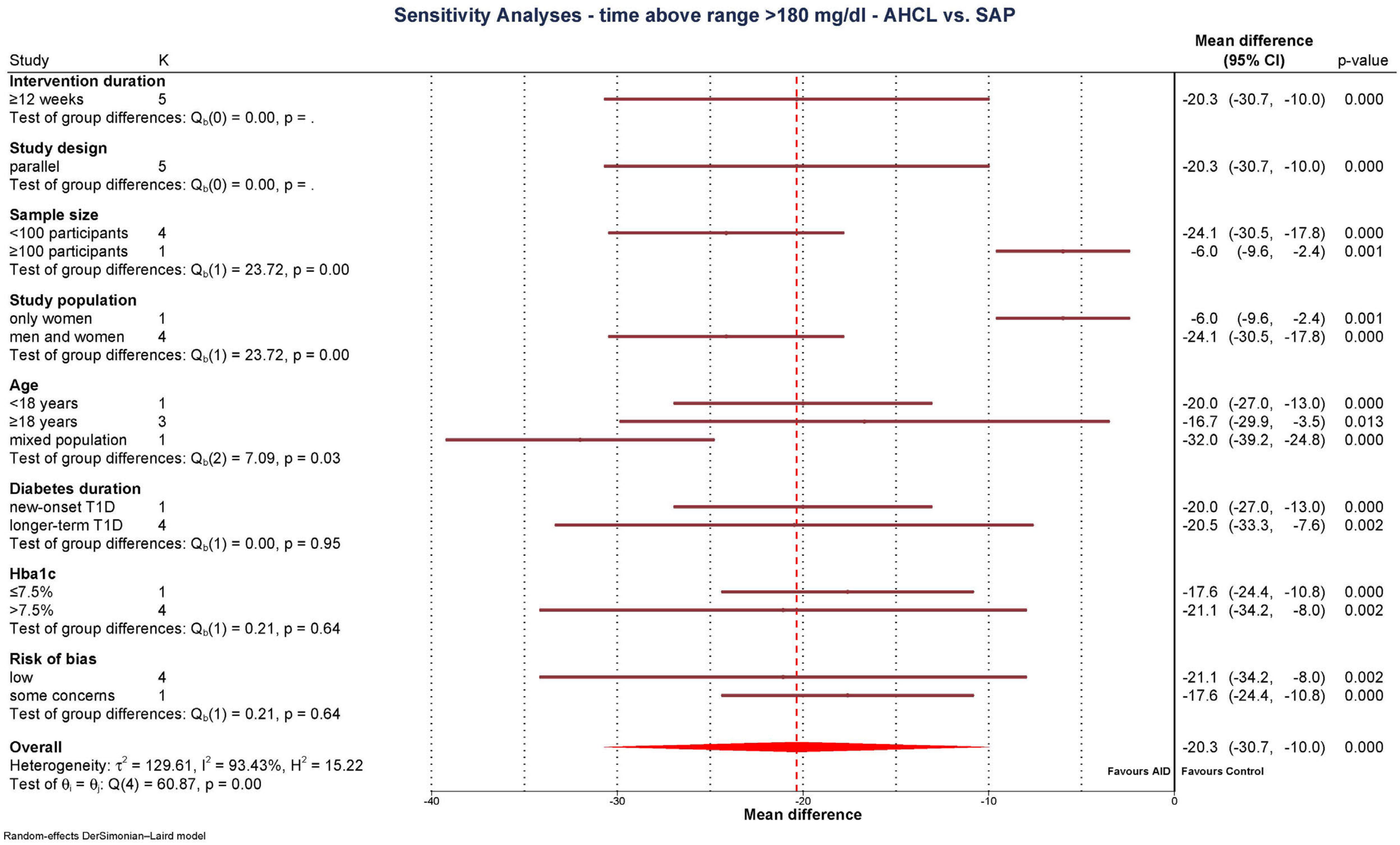
Sensitivity Analyses - time above range >180 mg/dl - AHCL vs. SAP
Study
K
Mean difference (95% CI)
p-value
Intervention duration
≥12 weeks 5 -20.3 (-30.7, -10.0) 0.000
Test of group differences: $Q_b(0) = 0.00$, p = .
Study design
parallel 5 -20.3 (-30.7, -10.0) 0.000
Test of group differences: $Q_b(0) = 0.00$, p = .
Sample size
<100 participants 4 -24.1 (-30.5, -17.8) 0.000
≥100 participants 1 -6.0 (-9.6, -2.4) 0.001
Test of group differences: $Q_b(1) = 23.72$, p = 0.00
Study population
only women 1 -6.0 (-9.6, -2.4) 0.001
men and women 4 -24.1 (-30.5, -17.8) 0.000
Test of group differences: $Q_b(1) = 23.72$, p = 0.00
Age
<18 years 1 -20.0 (-27.0, -13.0) 0.000
≥18 years 3 -16.7 (-29.9, -3.5) 0.013
mixed population 1 -32.0 (-39.2, -24.8) 0.000
Test of group differences: $Q_b(2) = 7.09$, p = 0.03
Diabetes duration
new-onset T1D 1 -20.0 (-27.0, -13.0) 0.000
longer-term T1D 4 -20.5 (-33.3, -7.6) 0.002
Test of group differences: $Q_b(1) = 0.00$, p = 0.95
Hba1c
≤7.5% 1 -17.6 (-24.4, -10.8) 0.000
>7.5% 4 -21.1 (-34.2, -8.0) 0.002
Test of group differences: $Q_b(1) = 0.21$, p = 0.64
Risk of bias
low 4 -21.1 (-34.2, -8.0) 0.002
some concerns 1 -17.6 (-24.4, -10.8) 0.000
Test of group differences: $Q_b(1) = 0.21$, p = 0.64
Overall -20.3 (-30.7, -10.0) 0.000
Heterogeneity: $\tau^2 = 129.61$, $I^2 = 93.43\%$, $H^2 = 15.22$
Test of $\theta_i = \theta_j$: Q(4) = 60.87, p = 0.00
Favours AID
Favours Control
-40
-30
-20
-10
0
Mean difference
Random-effects DerSimonian–Laird model

Sensitivity Analyses - time above range >180 mg/dl - AHCL vs. PLGM

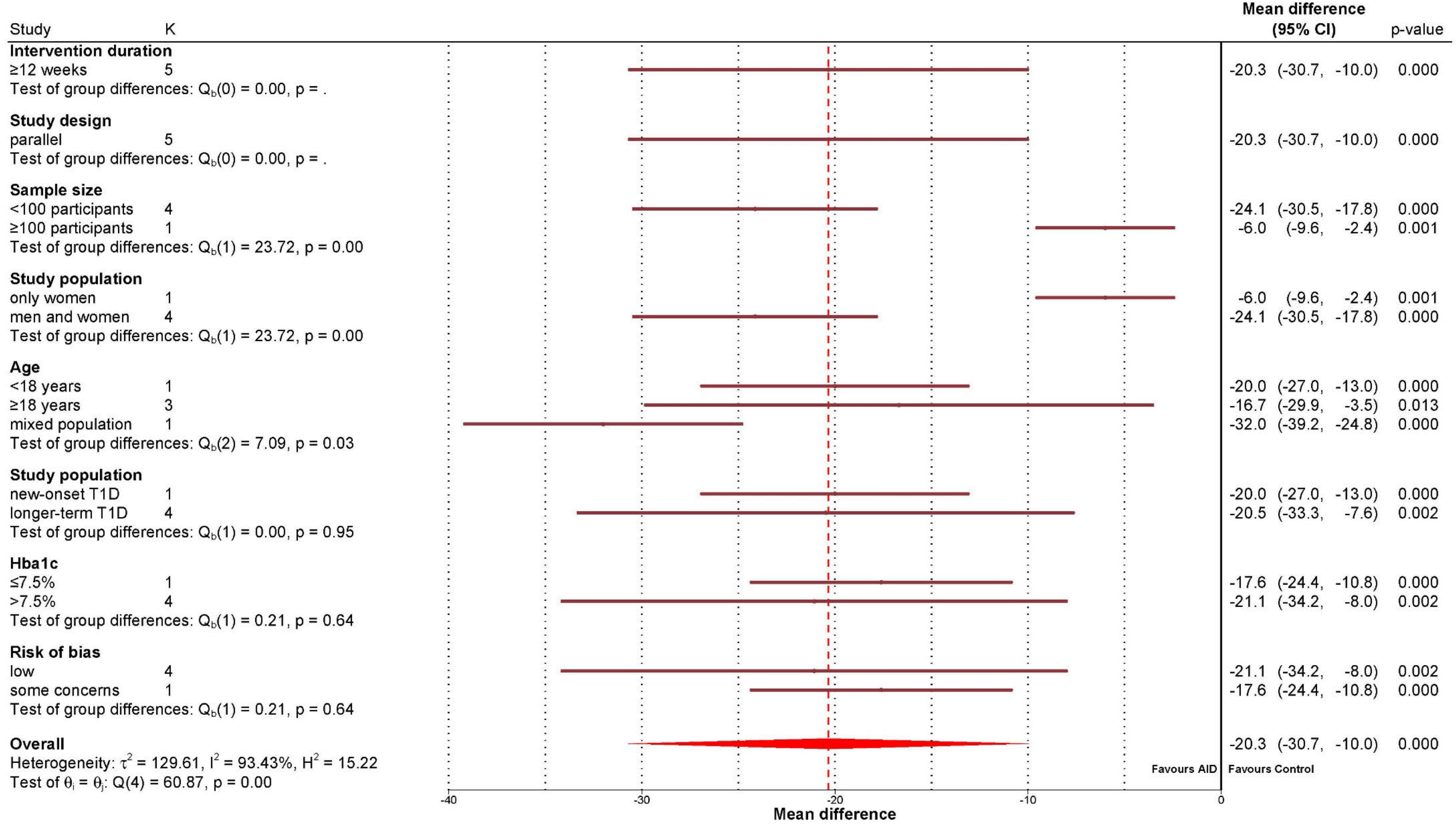

Random-effects DerSimonian–Laird model

Sensitivity Analyses - time above range >180 mg/dl - AHCL vs. HCL

Study	K	Mean difference (95% CI)	p-value
**Intervention duration**			
≥12 weeks	5	-20.3 (-30.7, -10.0)	0.000
Test of group differences: $Q_b(0) = 0.00$, p = .			
**Study design**			
parallel	5	-20.3 (-30.7, -10.0)	0.000
Test of group differences: $Q_b(0) = 0.00$, p = .			
**Sample size**			
<100 participants	4	-24.1 (-30.5, -17.8)	0.000
≥100 participants	1	-6.0 (-9.6, -2.4)	0.001
Test of group differences: $Q_b(1) = 23.72$, p = 0.00			
**Study population**			
only women	1	-6.0 (-9.6, -2.4)	0.001
men and women	4	-24.1 (-30.5, -17.8)	0.000
Test of group differences: $Q_b(1) = 23.72$, p = 0.00			
**Age**			
<18 years	1	-20.0 (-27.0, -13.0)	0.000
≥18 years	3	-16.7 (-29.9, -3.5)	0.013
mixed population	1	-32.0 (-39.2, -24.8)	0.000
Test of group differences: $Q_b(2) = 7.09$, p = 0.03			
**Diabetes duration**			
new-onset T1D	1	-20.0 (-27.0, -13.0)	0.000
longer-term T1D	4	-20.5 (-33.3, -7.6)	0.002
Test of group differences: $Q_b(1) = 0.00$, p = 0.95			
**Hba1c**			
≤7.5%	1	-17.6 (-24.4, -10.8)	0.000
>7.5%	4	-21.1 (-34.2, -8.0)	0.002
Test of group differences: $Q_b(1) = 0.21$, p = 0.64			
**Risk of bias**			
low	4	-21.1 (-34.2, -8.0)	0.002
some concerns	1	-17.6 (-24.4, -10.8)	0.000
Test of group differences: $Q_b(1) = 0.21$, p = 0.64			
**Overall**		-20.3 (-30.7, -10.0)	0.000
Heterogeneity: $\tau^2 = 129.61$, $I^2 = 93.43\%$, $H^2 = 15.22$			
Test of $\theta_i = \theta_j$: Q(4) = 60.87, p = 0.00			

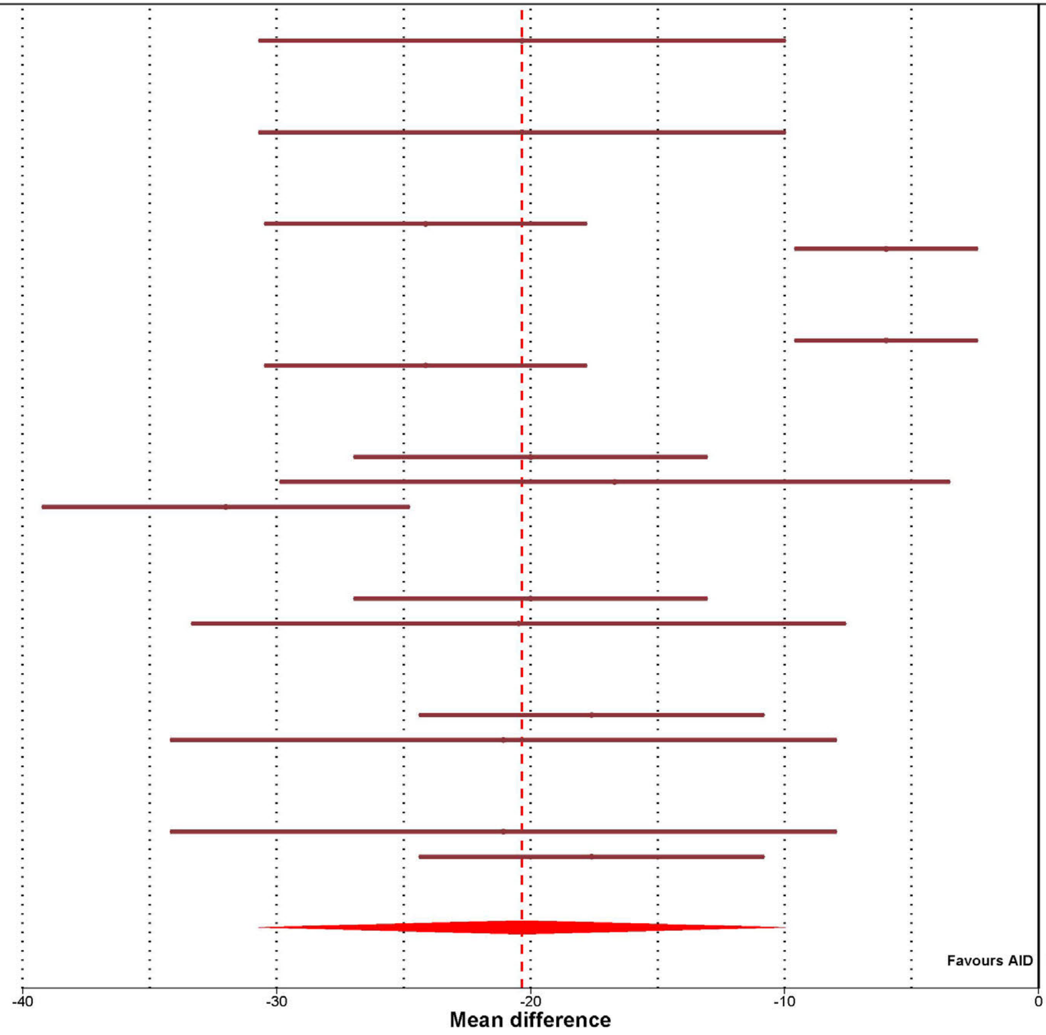

Random-effects DerSimonian–Laird model

5.3 Sensitivity analyses TAR >250 mg/dl

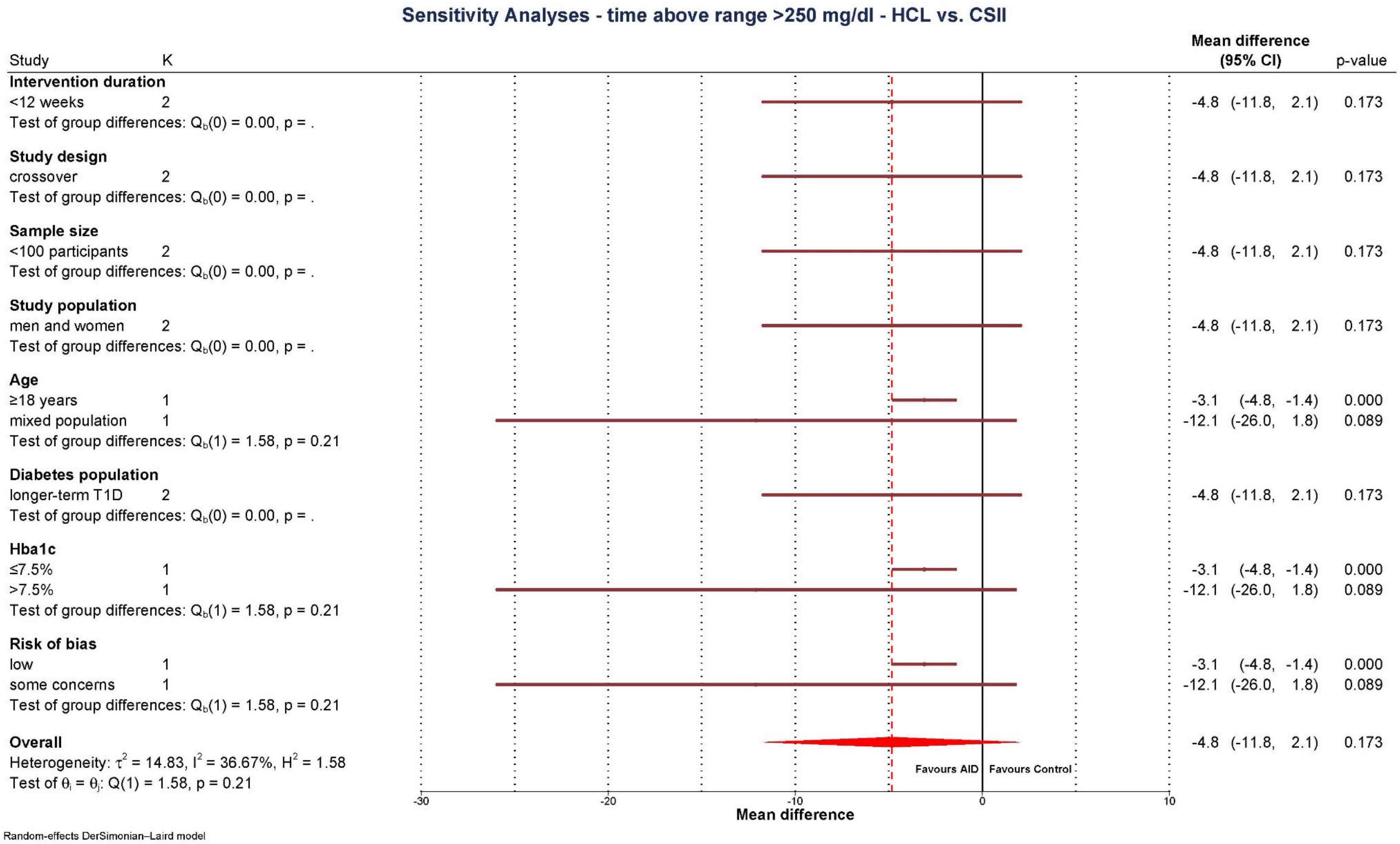

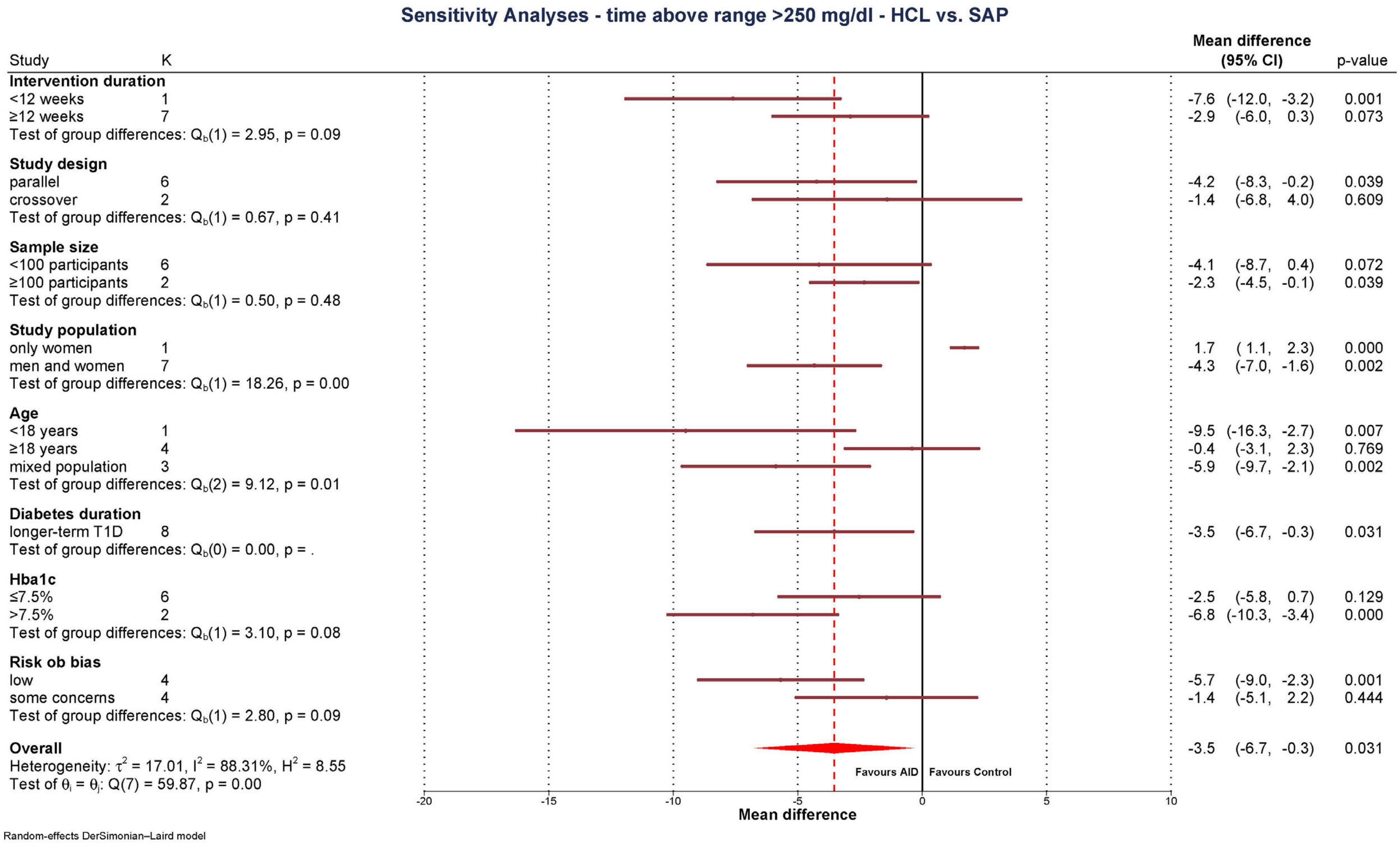
Sensitivity Analyses - time above range >250 mg/dl - HCL vs. SAP
Study
K
Mean difference (95% CI)
p-value
Intervention duration
<12 weeks 1 -7.6 (-12.0, -3.2) 0.001
≥12 weeks 7 -2.9 (-6.0, 0.3) 0.073
Test of group differences: $Q_b(1) = 2.95$, p = 0.09
Study design
parallel 6 -4.2 (-8.3, -0.2) 0.039
crossover 2 -1.4 (-6.8, 4.0) 0.609
Test of group differences: $Q_b(1) = 0.67$, p = 0.41
Sample size
<100 participants 6 -4.1 (-8.7, 0.4) 0.072
≥100 participants 2 -2.3 (-4.5, -0.1) 0.039
Test of group differences: $Q_b(1) = 0.50$, p = 0.48
Study population
only women 1 1.7 (1.1, 2.3) 0.000
men and women 7 -4.3 (-7.0, -1.6) 0.002
Test of group differences: $Q_b(1) = 18.26$, p = 0.00
Age
<18 years 1 -9.5 (-16.3, -2.7) 0.007
≥18 years 4 -0.4 (-3.1, 2.3) 0.769
mixed population 3 -5.9 (-9.7, -2.1) 0.002
Test of group differences: $Q_b(2) = 9.12$, p = 0.01
Diabetes duration
longer-term T1D 8 -3.5 (-6.7, -0.3) 0.031
Test of group differences: $Q_b(0) = 0.00$, p = .
Hba1c
≤7.5% 6 -2.5 (-5.8, 0.7) 0.129
>7.5% 2 -6.8 (-10.3, -3.4) 0.000
Test of group differences: $Q_b(1) = 3.10$, p = 0.08
Risk ob bias
low 4 -5.7 (-9.0, -2.3) 0.001
some concerns 4 -1.4 (-5.1, 2.2) 0.444
Test of group differences: $Q_b(1) = 2.80$, p = 0.09
Overall -3.5 (-6.7, -0.3) 0.031
Heterogeneity: $\tau^2 = 17.01$, $I^2 = 88.31\%$, $H^2 = 8.55$
Test of $\theta_i = \theta_j$: Q(7) = 59.87, p = 0.00
Favours AID
Favours Control
-20
-15
-10
-5
0
5
10
Mean difference
Random-effects DerSimonian–Laird model

Sensitivity Analyses - time above range >250 mg/dl - HCL vs. PLGM

Study	K	Mean difference (95% CI)	p-value
**Intervention duration**			
<12 weeks	1	-7.6 (-12.0, -3.2)	0.001
≥12 weeks	7	-2.9 (-6.0, 0.3)	0.073
Test of group differences: $Q_b(1) = 2.95$, p = 0.09			
**Study design**			
parallel	6	-4.2 (-8.3, -0.2)	0.039
crossover	2	-1.4 (-6.8, 4.0)	0.609
Test of group differences: $Q_b(1) = 0.67$, p = 0.41			
**Sample size**			
<100 participants	6	-4.1 (-8.7, 0.4)	0.072
≥100 participants	2	-2.3 (-4.5, -0.1)	0.039
Test of group differences: $Q_b(1) = 0.50$, p = 0.48			
**Study population**			
only women	1	1.7 (1.1, 2.3)	0.000
men and women	7	-4.3 (-7.0, -1.6)	0.002
Test of group differences: $Q_b(1) = 18.26$, p = 0.00			
**Age**			
<18 years	1	-9.5 (-16.3, -2.7)	0.007
≥18 years	4	-0.4 (-3.1, 2.3)	0.769
mixed population	3	-5.9 (-9.7, -2.1)	0.002
Test of group differences: $Q_b(2) = 9.12$, p = 0.01			
**Diabetes duration**			
longer-term T1D	8	-3.5 (-6.7, -0.3)	0.031
Test of group differences: $Q_b(0) = 0.00$, p = .			
**Hba1c**			
≤7.5%	6	-2.5 (-5.8, 0.7)	0.129
>7.5%	2	-6.8 (-10.3, -3.4)	0.000
Test of group differences: $Q_b(1) = 3.10$, p = 0.08			
**Risk of bias**			
low	4	-5.7 (-9.0, -2.3)	0.001
some concerns	4	-1.4 (-5.1, 2.2)	0.444
Test of group differences: $Q_b(1) = 2.80$, p = 0.09			
**Overall**		-3.5 (-6.7, -0.3)	0.031
Heterogeneity: $\tau^2 = 17.01$, $I^2 = 88.31\%$, $H^2 = 8.55$			
Test of $\theta_i = \theta_j$: Q(7) = 59.87, p = 0.00			

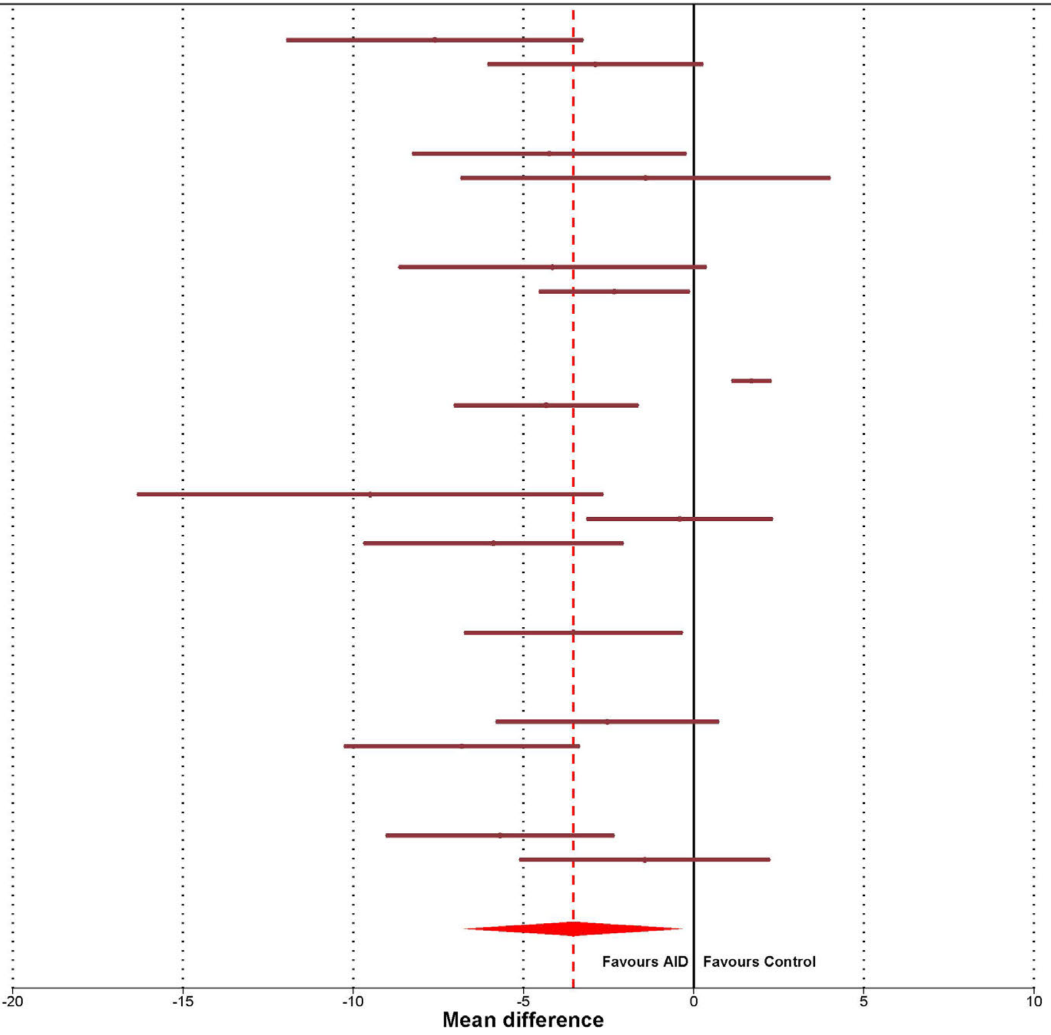

Random-effects DerSimonian–Laird model

Sensitivity Analyses - time above range >250 mg/dl - AHCL vs. MDI

Study	K	Mean difference (95% CI)	p-value
**Intervention duration**			
≥12 weeks	3	-18.6 (-30.8, -6.4)	0.003
Test of group differences: $Q_b(0) = 0.00$, p = .			
**Study design**			
parallel	3	-18.6 (-30.8, -6.4)	0.003
Test of group differences: $Q_b(0) = 0.00$, p = .			
**Sample size**			
<100 participants	3	-18.6 (-30.8, -6.4)	0.003
Test of group differences: $Q_b(0) = 0.00$, p = .			
**Study population**			
men and women	3	-18.6 (-30.8, -6.4)	0.003
Test of group differences: $Q_b(0) = 0.00$, p = .			
**Age**			
≥18 years	2	-11.7 (-19.7, -3.6)	0.004
mixed population	1	-34.0 (-42.4, -25.6)	0.000
Test of group differences: $Q_b(1) = 14.18$, p = 0.00			
**Diabetes duration**			
longer-term T1D	3	-18.6 (-30.8, -6.4)	0.003
Test of group differences: $Q_b(0) = 0.00$, p = .			
**Hba1c**			
≤7.5%	1	-7.7 (-11.4, -4.0)	0.000
>7.5%	2	-24.6 (-42.3, -6.9)	0.007
Test of group differences: $Q_b(1) = 3.35$, p = 0.07			
**Risk of bias**			
low	2	-24.6 (-42.3, -6.9)	0.007
some concerns	1	-7.7 (-11.4, -4.0)	0.000
Test of group differences: $Q_b(1) = 3.35$, p = 0.07			
**Overall**		-18.6 (-30.8, -6.4)	0.003
Heterogeneity: $\tau^2 = 107.49$, $I^2 = 94.00\%$, $H^2 = 16.66$			
Test of $\theta_i = \theta_j$: $Q(2) = 33.31$, p = 0.00			

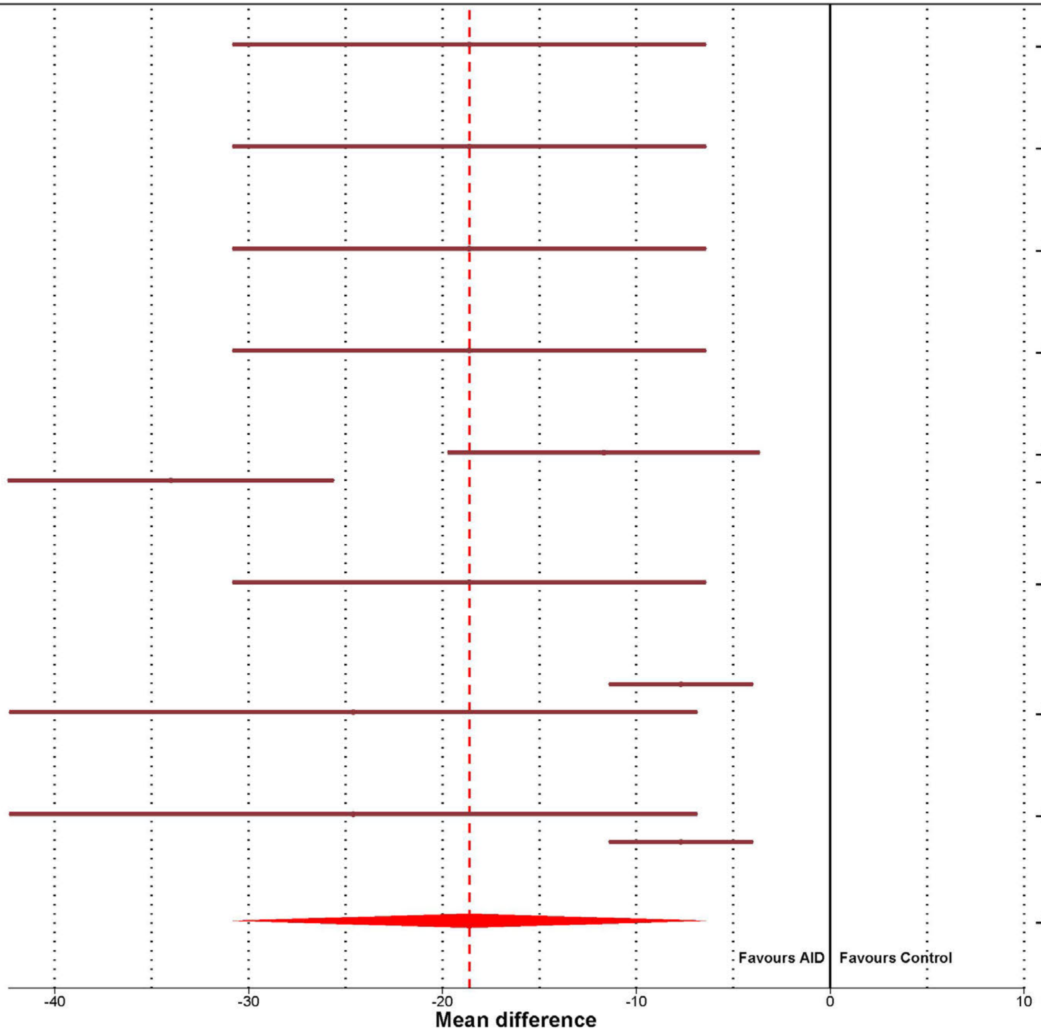

Random-effects DerSimonian–Laird model

Sensitivity Analyses - time above range >250 mg/dl - AHCL vs. SAP

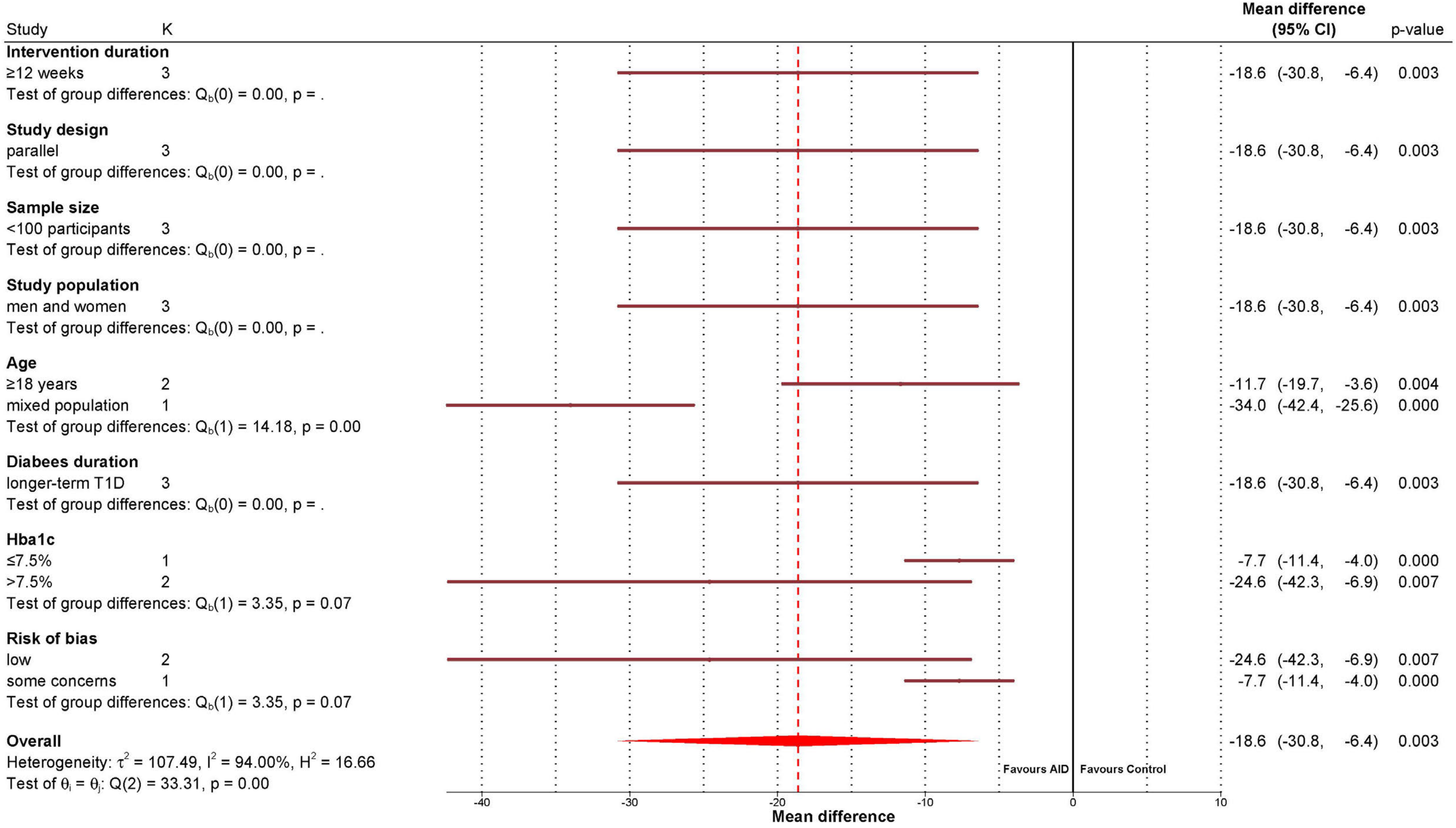

Random-effects DerSimonian–Laird model

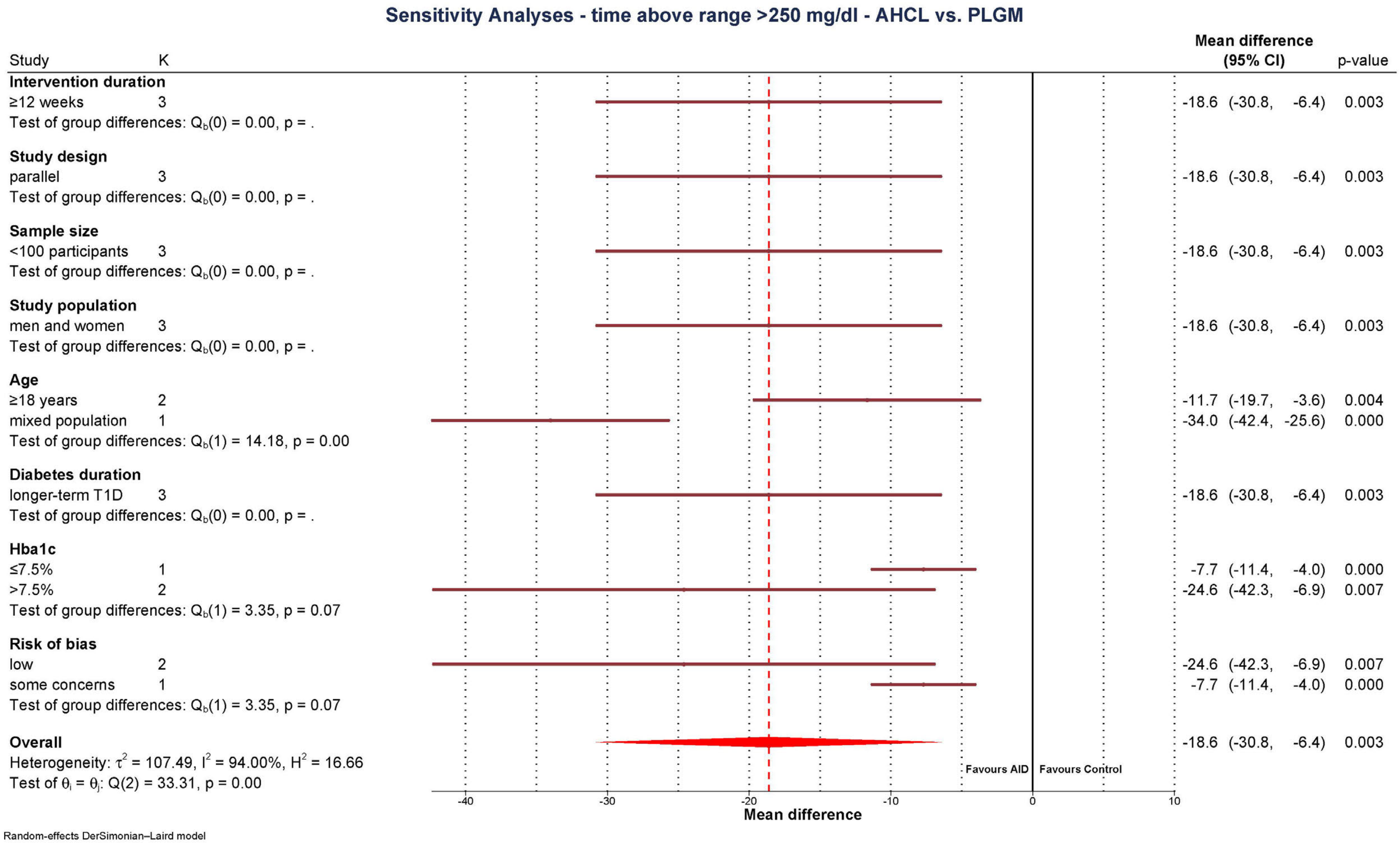
Sensitivity Analyses - time above range >250 mg/dl - AHCL vs. PLGM
Study K
Mean difference (95% CI) p-value
Intervention duration
≥12 weeks 3 -18.6 (-30.8, -6.4) 0.003
Test of group differences: Q_b(0) = 0.00, p = .
Study design
parallel 3 -18.6 (-30.8, -6.4) 0.003
Test of group differences: Q_b(0) = 0.00, p = .
Sample size
<100 participants 3 -18.6 (-30.8, -6.4) 0.003
Test of group differences: Q_b(0) = 0.00, p = .
Study population
men and women 3 -18.6 (-30.8, -6.4) 0.003
Test of group differences: Q_b(0) = 0.00, p = .
Age
≥18 years 2 -11.7 (-19.7, -3.6) 0.004
mixed population 1 -34.0 (-42.4, -25.6) 0.000
Test of group differences: Q_b(1) = 14.18, p = 0.00
Diabetes duration
longer-term T1D 3 -18.6 (-30.8, -6.4) 0.003
Test of group differences: Q_b(0) = 0.00, p = .
Hba1c
≤7.5% 1 -7.7 (-11.4, -4.0) 0.000
>7.5% 2 -24.6 (-42.3, -6.9) 0.007
Test of group differences: Q_b(1) = 3.35, p = 0.07
Risk of bias
low 2 -24.6 (-42.3, -6.9) 0.007
some concerns 1 -7.7 (-11.4, -4.0) 0.000
Test of group differences: Q_b(1) = 3.35, p = 0.07
Overall -18.6 (-30.8, -6.4) 0.003
Heterogeneity: τ² = 107.49, I² = 94.00%, H² = 16.66
Test of θ_i = θ_j: Q(2) = 33.31, p = 0.00
Favours AID
Favours Control
-40
-30
-20
-10
0
10
Mean difference
Random-effects DerSimonian–Laird model

Sensitivity Analyses - time above range >250 mg/dl - AHCL vs. HCL

Study	K	Mean difference (95% CI)	p-value
**Intervention duration**			
≥12 weeks	3	-18.6 (-30.8, -6.4)	0.003
Test of group differences: $Q_b(0) = 0.00$, p = .			
**Study design**			
parallel	3	-18.6 (-30.8, -6.4)	0.003
Test of group differences: $Q_b(0) = 0.00$, p = .			
**Sample size**			
<100 participants	3	-18.6 (-30.8, -6.4)	0.003
Test of group differences: $Q_b(0) = 0.00$, p = .			
**Study population**			
men and women	3	-18.6 (-30.8, -6.4)	0.003
Test of group differences: $Q_b(0) = 0.00$, p = .			
**Age**			
≥18 years	2	-11.7 (-19.7, -3.6)	0.004
mixed population	1	-34.0 (-42.4, -25.6)	0.000
Test of group differences: $Q_b(1) = 14.18$, p = 0.00			
**Diabetes duration**			
longer-term T1D	3	-18.6 (-30.8, -6.4)	0.003
Test of group differences: $Q_b(0) = 0.00$, p = .			
**Hba1c**			
≤7.5%	1	-7.7 (-11.4, -4.0)	0.000
>7.5%	2	-24.6 (-42.3, -6.9)	0.007
Test of group differences: $Q_b(1) = 3.35$, p = 0.07			
**Risk of bias**			
low	2	-24.6 (-42.3, -6.9)	0.007
some concerns	1	-7.7 (-11.4, -4.0)	0.000
Test of group differences: $Q_b(1) = 3.35$, p = 0.07			
**Overall**		-18.6 (-30.8, -6.4)	0.003
Heterogeneity: $\tau^2 = 107.49$, $I^2 = 94.00\%$, $H^2 = 16.66$			
Test of $\theta_i = \theta_j$: $Q(2) = 33.31$, p = 0.00			

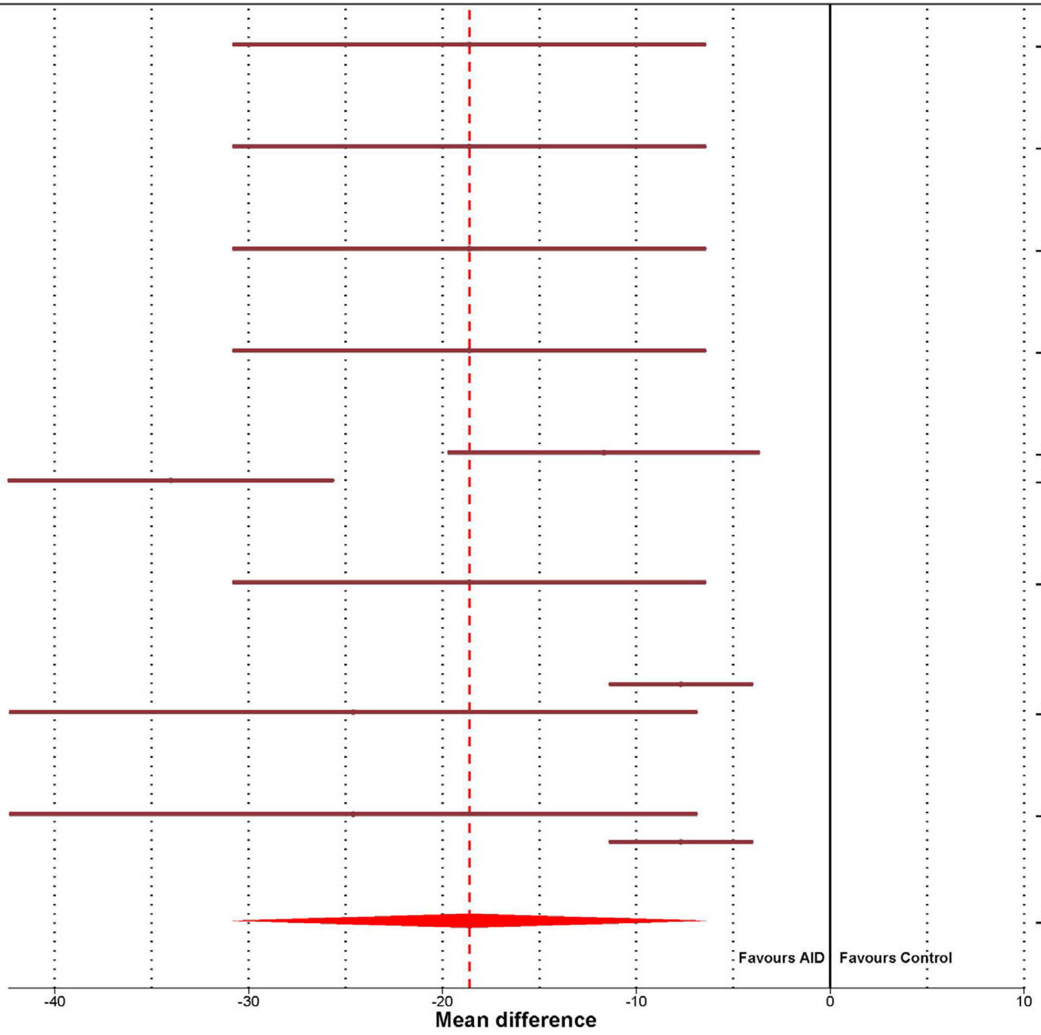

Random-effects DerSimonian–Laird model

5.4 Sensitivity analyses TBR <70 mg/dl

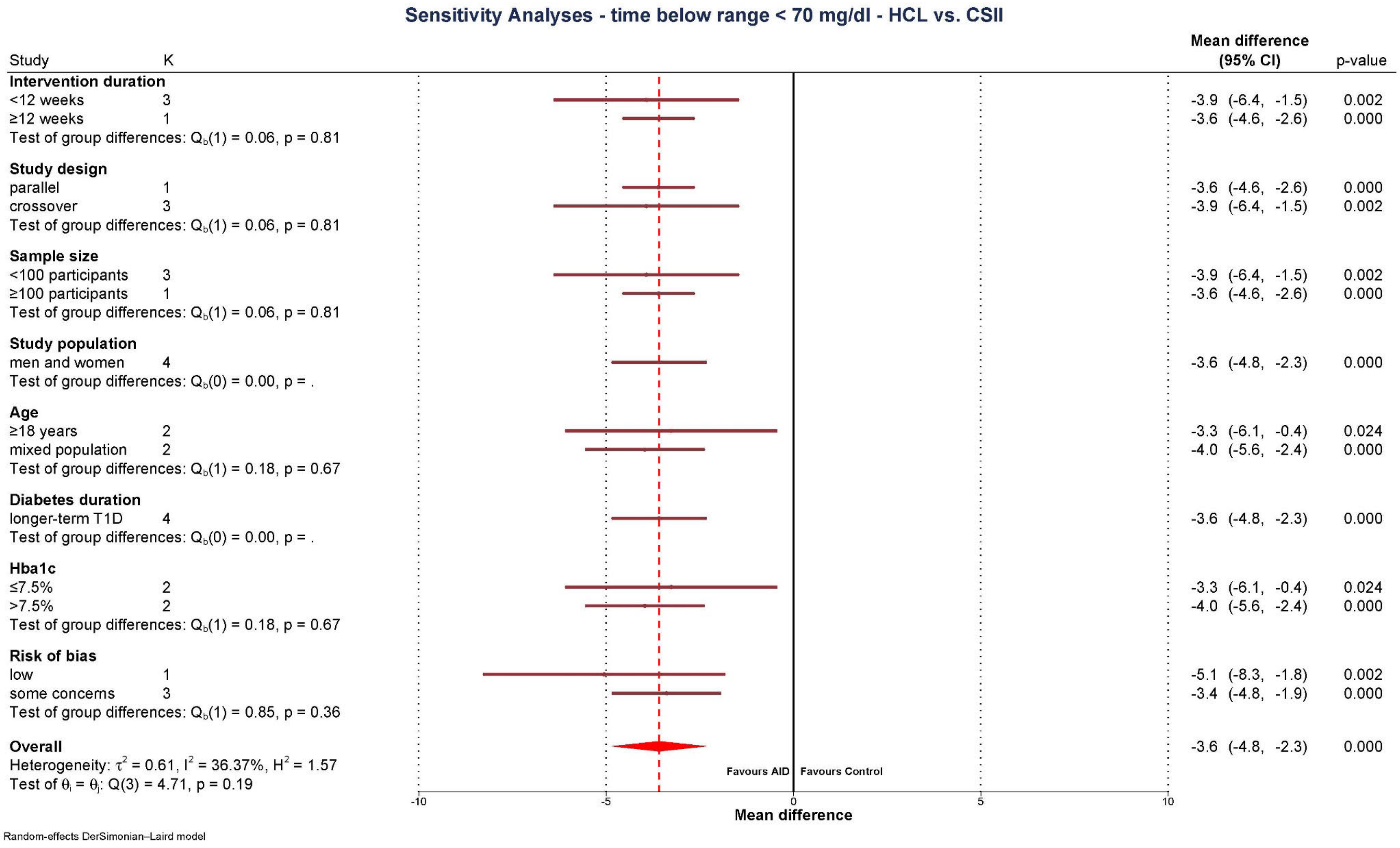

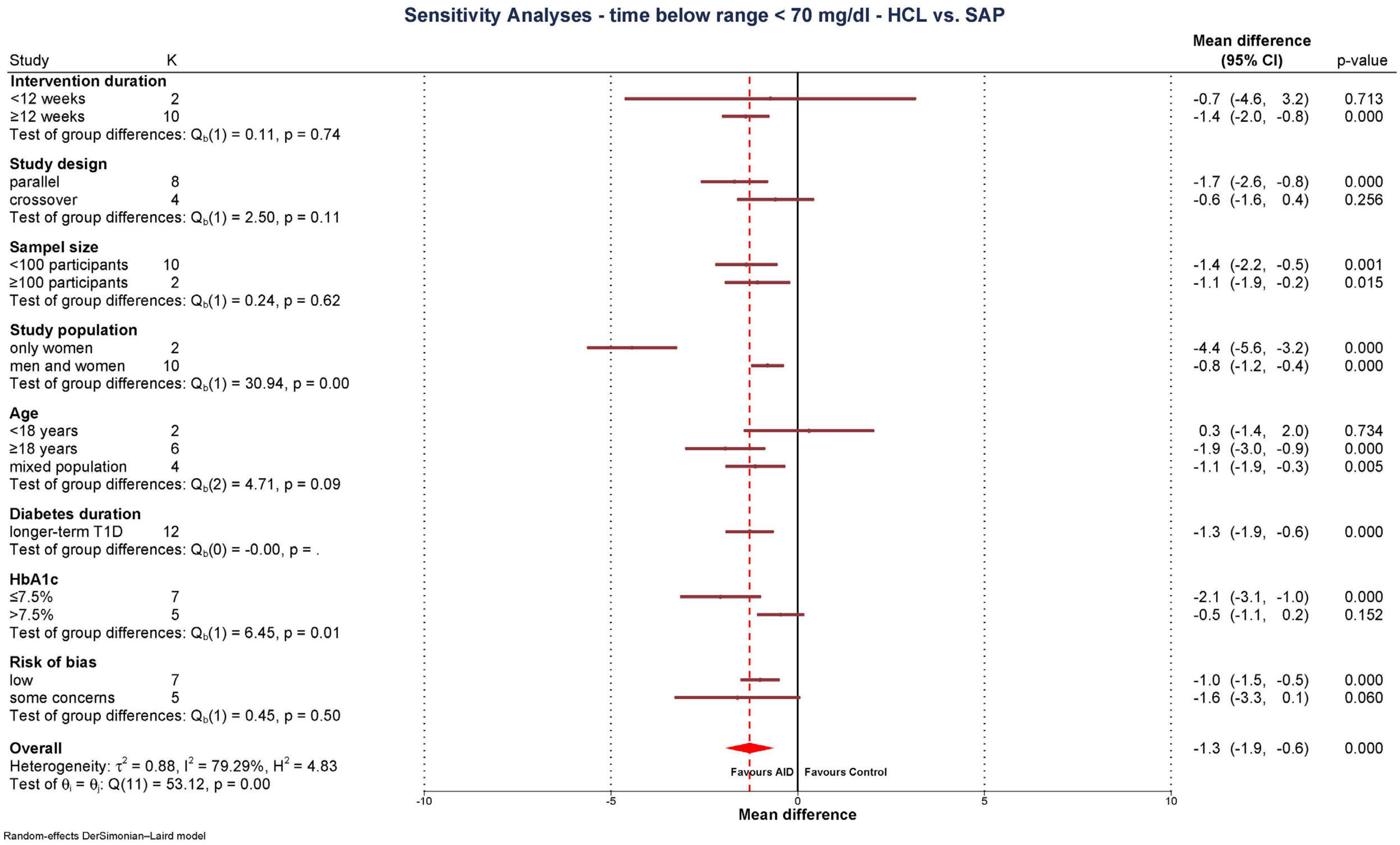

Sensitivity Analyses - time below range < 70 mg/dl - HCL vs. SAP
Study K
Mean difference (95% CI)
p-value
Intervention duration
<12 weeks 2 -0.7 (-4.6, 3.2) 0.713
≥12 weeks 10 -1.4 (-2.0, -0.8) 0.000
Test of group differences: $Q_b(1)$ = 0.11, p = 0.74
Study design
parallel 8 -1.7 (-2.6, -0.8) 0.000
crossover 4 -0.6 (-1.6, 0.4) 0.256
Test of group differences: $Q_b(1)$ = 2.50, p = 0.11
Sampel size
<100 participants 10 -1.4 (-2.2, -0.5) 0.001
≥100 participants 2 -1.1 (-1.9, -0.2) 0.015
Test of group differences: $Q_b(1)$ = 0.24, p = 0.62
Study population
only women 2 -4.4 (-5.6, -3.2) 0.000
men and women 10 -0.8 (-1.2, -0.4) 0.000
Test of group differences: $Q_b(1)$ = 30.94, p = 0.00
Age
<18 years 2 0.3 (-1.4, 2.0) 0.734
≥18 years 6 -1.9 (-3.0, -0.9) 0.000
mixed population 4 -1.1 (-1.9, -0.3) 0.005
Test of group differences: $Q_b(2)$ = 4.71, p = 0.09
Diabetes duration
longer-term T1D 12 -1.3 (-1.9, -0.6) 0.000
Test of group differences: $Q_b(0)$ = -0.00, p = .
HbA1c
≤7.5% 7 -2.1 (-3.1, -1.0) 0.000
>7.5% 5 -0.5 (-1.1, 0.2) 0.152
Test of group differences: $Q_b(1)$ = 6.45, p = 0.01
Risk of bias
low 7 -1.0 (-1.5, -0.5) 0.000
some concerns 5 -1.6 (-3.3, 0.1) 0.060
Test of group differences: $Q_b(1)$ = 0.45, p = 0.50
Overall -1.3 (-1.9, -0.6) 0.000
Heterogeneity: τ^2 = 0.88, I^2 = 79.29%, H^2 = 4.83
Test of $\theta_i = \theta_j$: Q(11) = 53.12, p = 0.00
Favours AID
Favours Control
-10
-5
0
5
10
Mean difference
Random-effects DerSimonian–Laird model

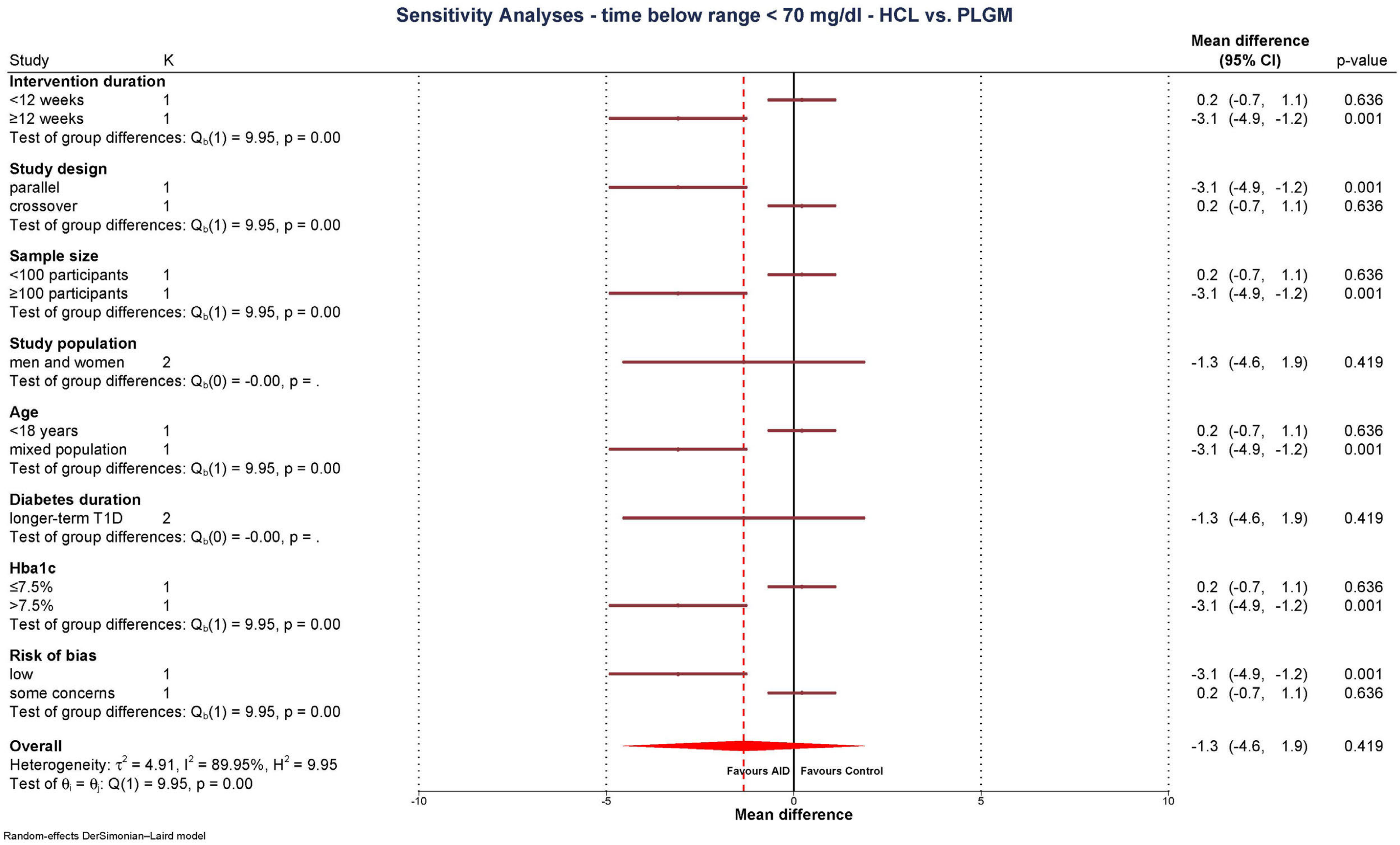
Sensitivity Analyses - time below range < 70 mg/dl - HCL vs. PLGM
Study K
Mean difference (95% CI)
p-value
Intervention duration
<12 weeks 1 0.2 (-0.7, 1.1) 0.636
≥12 weeks 1 -3.1 (-4.9, -1.2) 0.001
Test of group differences: $Q_b(1)$ = 9.95, p = 0.00
Study design
parallel 1 -3.1 (-4.9, -1.2) 0.001
crossover 1 0.2 (-0.7, 1.1) 0.636
Test of group differences: $Q_b(1)$ = 9.95, p = 0.00
Sample size
<100 participants 1 0.2 (-0.7, 1.1) 0.636
≥100 participants 1 -3.1 (-4.9, -1.2) 0.001
Test of group differences: $Q_b(1)$ = 9.95, p = 0.00
Study population
men and women 2 -1.3 (-4.6, 1.9) 0.419
Test of group differences: $Q_b(0)$ = -0.00, p = .
Age
<18 years 1 0.2 (-0.7, 1.1) 0.636
mixed population 1 -3.1 (-4.9, -1.2) 0.001
Test of group differences: $Q_b(1)$ = 9.95, p = 0.00
Diabetes duration
longer-term T1D 2 -1.3 (-4.6, 1.9) 0.419
Test of group differences: $Q_b(0)$ = -0.00, p = .
Hba1c
≤7.5% 1 0.2 (-0.7, 1.1) 0.636
>7.5% 1 -3.1 (-4.9, -1.2) 0.001
Test of group differences: $Q_b(1)$ = 9.95, p = 0.00
Risk of bias
low 1 -3.1 (-4.9, -1.2) 0.001
some concerns 1 0.2 (-0.7, 1.1) 0.636
Test of group differences: $Q_b(1)$ = 9.95, p = 0.00
Overall -1.3 (-4.6, 1.9) 0.419
Heterogeneity: τ^2 = 4.91, I^2 = 89.95%, H^2 = 9.95
Test of $\theta_i = \theta_j$: Q(1) = 9.95, p = 0.00
Favours AID
Favours Control
-10
-5
0
5
10
Mean difference
Random-effects DerSimonian–Laird model

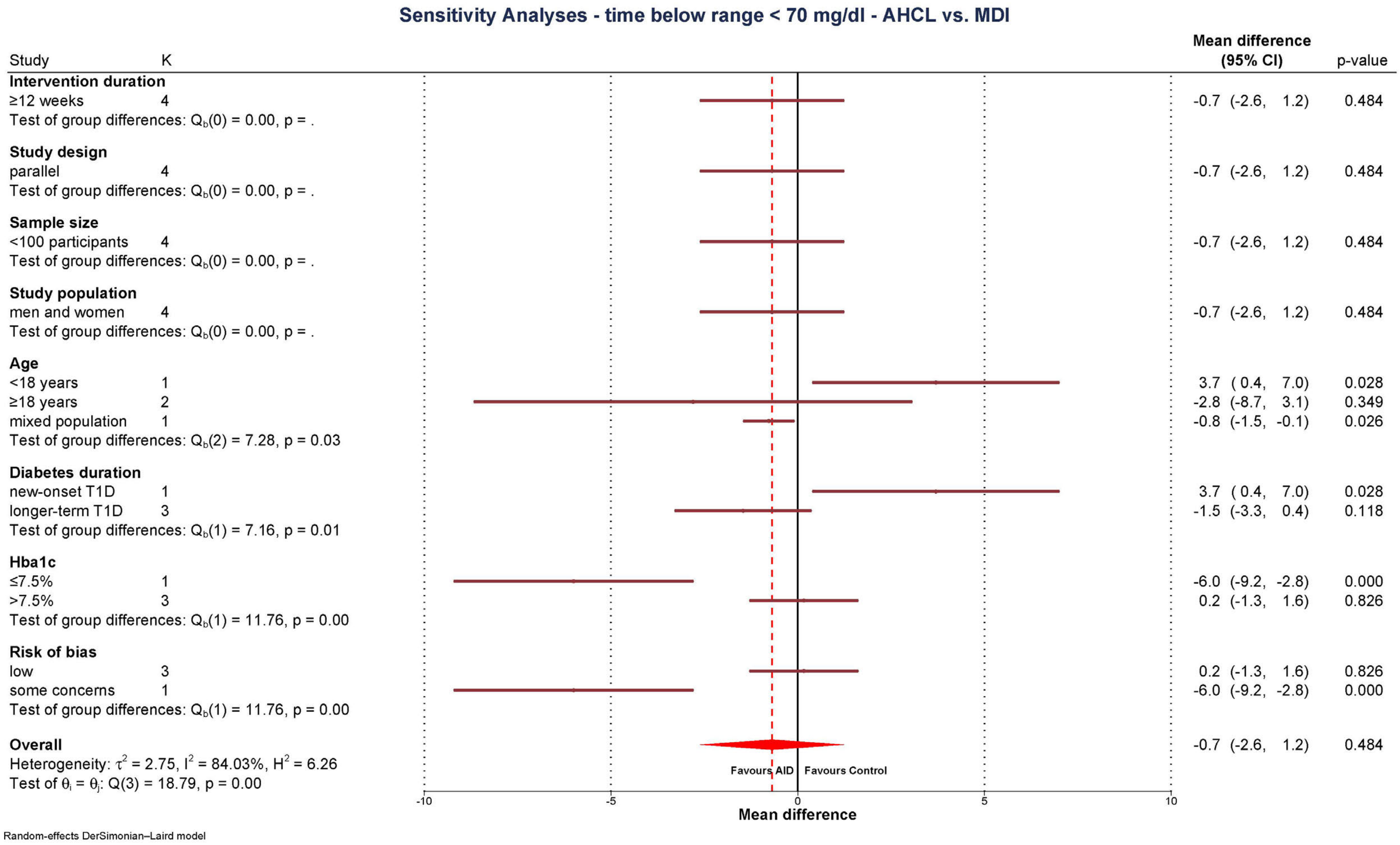
Sensitivity Analyses - time below range < 70 mg/dl - AHCL vs. MDI
Study K
Mean difference (95% CI)
p-value
Intervention duration
≥12 weeks 4 -0.7 (-2.6, 1.2) 0.484
Test of group differences: $Q_b(0)$ = 0.00, p = .
Study design
parallel 4 -0.7 (-2.6, 1.2) 0.484
Test of group differences: $Q_b(0)$ = 0.00, p = .
Sample size
<100 participants 4 -0.7 (-2.6, 1.2) 0.484
Test of group differences: $Q_b(0)$ = 0.00, p = .
Study population
men and women 4 -0.7 (-2.6, 1.2) 0.484
Test of group differences: $Q_b(0)$ = 0.00, p = .
Age
<18 years 1 3.7 (0.4, 7.0) 0.028
≥18 years 2 -2.8 (-8.7, 3.1) 0.349
mixed population 1 -0.8 (-1.5, -0.1) 0.026
Test of group differences: $Q_b(2)$ = 7.28, p = 0.03
Diabetes duration
new-onset T1D 1 3.7 (0.4, 7.0) 0.028
longer-term T1D 3 -1.5 (-3.3, 0.4) 0.118
Test of group differences: $Q_b(1)$ = 7.16, p = 0.01
Hba1c
≤7.5% 1 -6.0 (-9.2, -2.8) 0.000
>7.5% 3 0.2 (-1.3, 1.6) 0.826
Test of group differences: $Q_b(1)$ = 11.76, p = 0.00
Risk of bias
low 3 0.2 (-1.3, 1.6) 0.826
some concerns 1 -6.0 (-9.2, -2.8) 0.000
Test of group differences: $Q_b(1)$ = 11.76, p = 0.00
Overall -0.7 (-2.6, 1.2) 0.484
Heterogeneity: τ^2 = 2.75, I^2 = 84.03%, H^2 = 6.26
Test of $\theta_i = \theta_j$: Q(3) = 18.79, p = 0.00
Favours AID
Favours Control
-10
-5
0
5
10
Mean difference
Random-effects DerSimonian–Laird model

Sensitivity Analyses - time below range < 70 mg/dl - AHCL vs. SAP

Study	K	Mean difference (95% CI)	p-value
**Intervention duration**			
≥12 weeks	4	-0.7 (-2.6, 1.2)	0.484
Test of group differences: $Q_b(0) = 0.00$, p = .			
**Study design**			
parallel	4	-0.7 (-2.6, 1.2)	0.484
Test of group differences: $Q_b(0) = 0.00$, p = .			
**Sample size**			
<100 participants	4	-0.7 (-2.6, 1.2)	0.484
Test of group differences: $Q_b(0) = 0.00$, p = .			
**Study population**			
men and women	4	-0.7 (-2.6, 1.2)	0.484
Test of group differences: $Q_b(0) = 0.00$, p = .			
**Age**			
<18 years	1	3.7 (0.4, 7.0)	0.028
≥18 years	2	-2.8 (-8.7, 3.1)	0.349
mixed population	1	-0.8 (-1.5, -0.1)	0.026
Test of group differences: $Q_b(2) = 7.28$, p = 0.03			
**Diabetes duration**			
new-onset T1D	1	3.7 (0.4, 7.0)	0.028
longer-term T1D	3	-1.5 (-3.3, 0.4)	0.118
Test of group differences: $Q_b(1) = 7.16$, p = 0.01			
**Hba1c**			
≤7.5%	1	-6.0 (-9.2, -2.8)	0.000
>7.5%	3	0.2 (-1.3, 1.6)	0.826
Test of group differences: $Q_b(1) = 11.76$, p = 0.00			
**Risk of bias**			
low	3	0.2 (-1.3, 1.6)	0.826
some concerns	1	-6.0 (-9.2, -2.8)	0.000
Test of group differences: $Q_b(1) = 11.76$, p = 0.00			
**Overall**		-0.7 (-2.6, 1.2)	0.484
Heterogeneity: $\tau^2 = 2.75$, $I^2 = 84.03\%$, $H^2 = 6.26$			
Test of $\theta_i = \theta_j$: $Q(3) = 18.79$, p = 0.00			

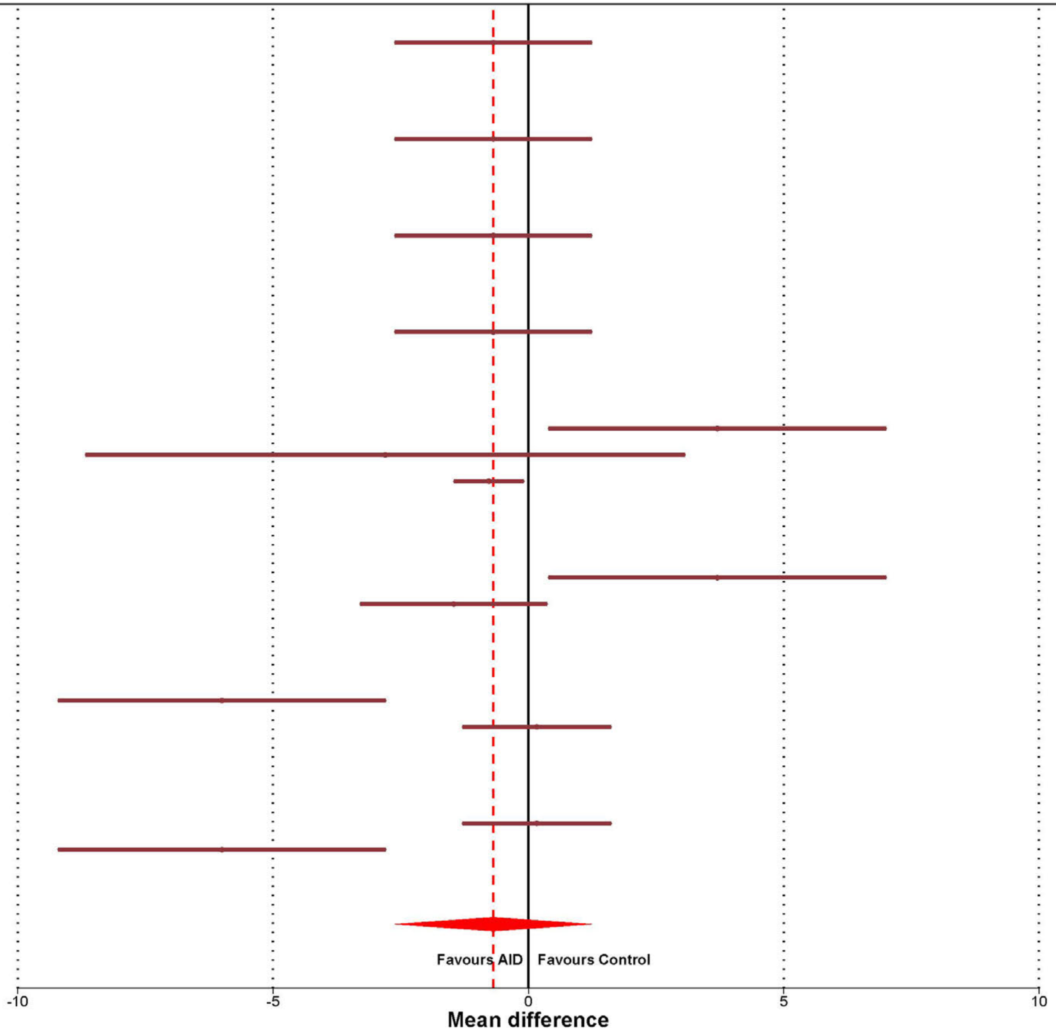

Random-effects DerSimonian–Laird model

Sensitivity Analyses - time below range < 70 mg/dl - AHCL vs. PLGM

Study	K	Mean difference (95% CI)	p-value
**Intervention duration**			
≥12 weeks	4	-0.7 (-2.6, 1.2)	0.484
Test of group differences: $Q_b(0) = 0.00$, p = .			
**Study design**			
parallel	4	-0.7 (-2.6, 1.2)	0.484
Test of group differences: $Q_b(0) = 0.00$, p = .			
**Sample size**			
<100 participants	4	-0.7 (-2.6, 1.2)	0.484
Test of group differences: $Q_b(0) = 0.00$, p = .			
**Study population**			
men and women	4	-0.7 (-2.6, 1.2)	0.484
Test of group differences: $Q_b(0) = 0.00$, p = .			
**Age**			
<18 years	1	3.7 (0.4, 7.0)	0.028
≥18 years	2	-2.8 (-8.7, 3.1)	0.349
mixed population	1	-0.8 (-1.5, -0.1)	0.026
Test of group differences: $Q_b(2) = 7.28$, p = 0.03			
**Diabetes duration**			
new-onset T1D	1	3.7 (0.4, 7.0)	0.028
longer-term T1D	3	-1.5 (-3.3, 0.4)	0.118
Test of group differences: $Q_b(1) = 7.16$, p = 0.01			
**Hba1c**			
≤7.5%	1	-6.0 (-9.2, -2.8)	0.000
>7.5%	3	0.2 (-1.3, 1.6)	0.826
Test of group differences: $Q_b(1) = 11.76$, p = 0.00			
**Risk of bias**			
low	3	0.2 (-1.3, 1.6)	0.826
some concerns	1	-6.0 (-9.2, -2.8)	0.000
Test of group differences: $Q_b(1) = 11.76$, p = 0.00			
**Overall**		-0.7 (-2.6, 1.2)	0.484
Heterogeneity: $\tau^2 = 2.75$, $I^2 = 84.03\%$, $H^2 = 6.26$			
Test of $\theta_i = \theta_j$: $Q(3) = 18.79$, p = 0.00			

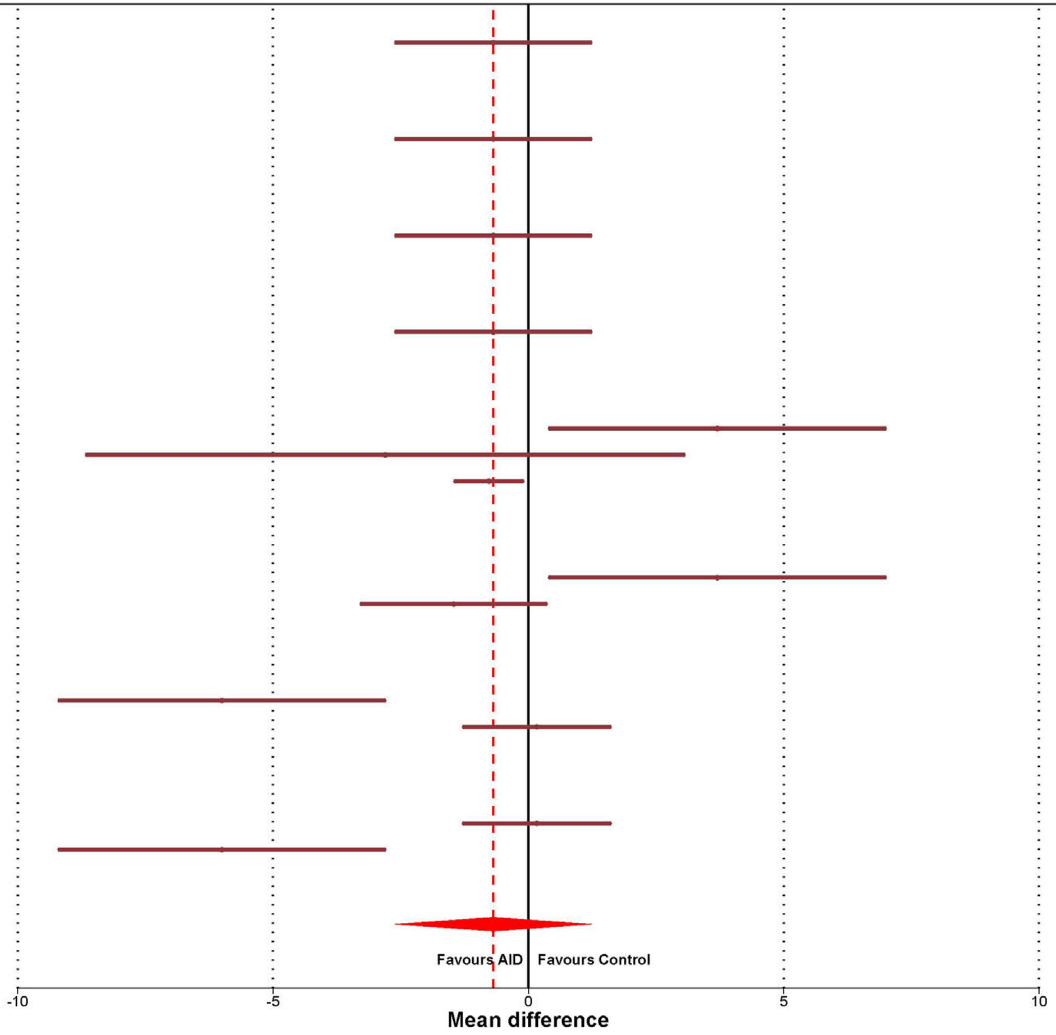

Random-effects DerSimonian–Laird model

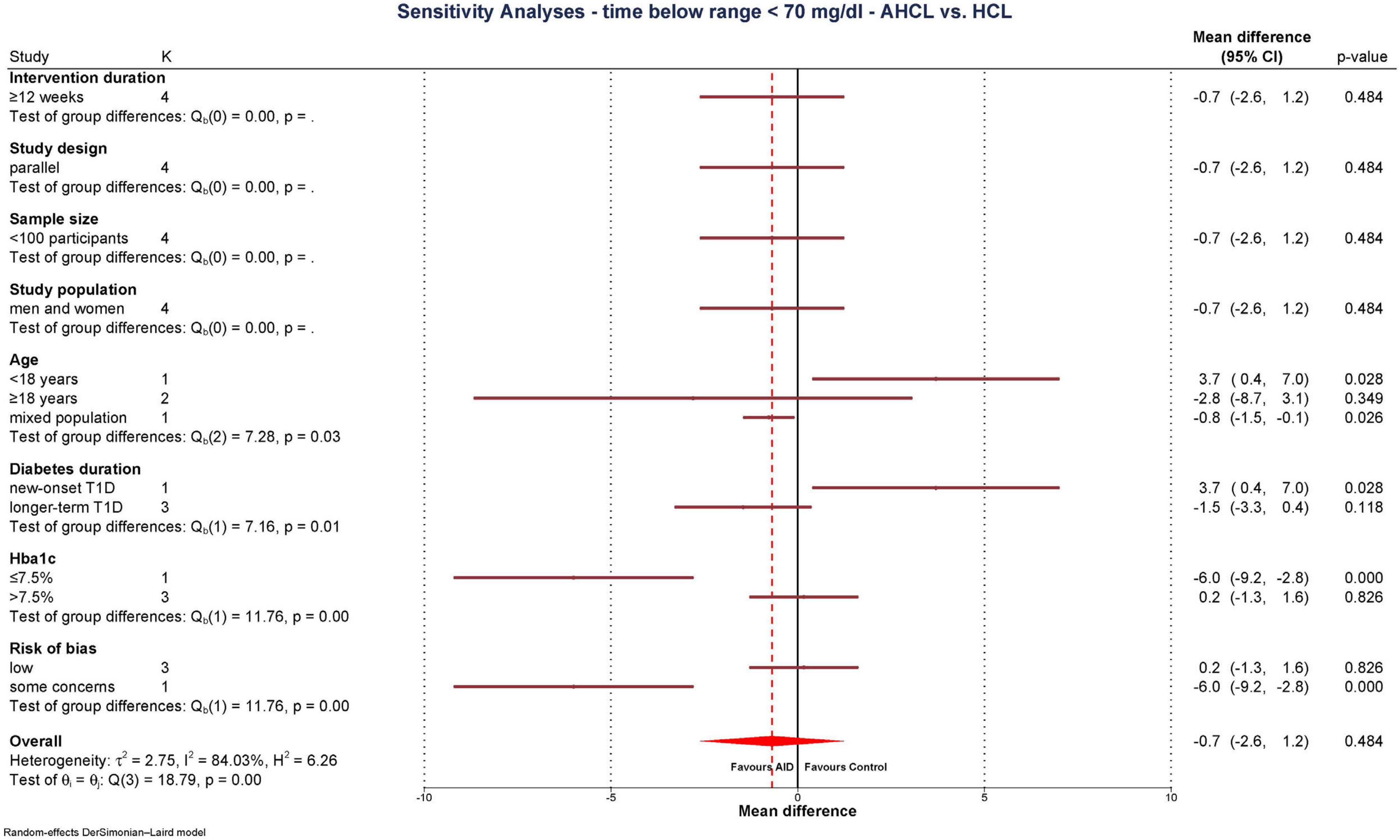
Sensitivity Analyses - time below range < 70 mg/dl - AHCL vs. HCL
Study K
Mean difference (95% CI)
p-value
Intervention duration
≥12 weeks 4 -0.7 (-2.6, 1.2) 0.484
Test of group differences: $Q_b(0) = 0.00$, p = .
Study design
parallel 4 -0.7 (-2.6, 1.2) 0.484
Test of group differences: $Q_b(0) = 0.00$, p = .
Sample size
<100 participants 4 -0.7 (-2.6, 1.2) 0.484
Test of group differences: $Q_b(0) = 0.00$, p = .
Study population
men and women 4 -0.7 (-2.6, 1.2) 0.484
Test of group differences: $Q_b(0) = 0.00$, p = .
Age
<18 years 1 3.7 (0.4, 7.0) 0.028
≥18 years 2 -2.8 (-8.7, 3.1) 0.349
mixed population 1 -0.8 (-1.5, -0.1) 0.026
Test of group differences: $Q_b(2) = 7.28$, p = 0.03
Diabetes duration
new-onset T1D 1 3.7 (0.4, 7.0) 0.028
longer-term T1D 3 -1.5 (-3.3, 0.4) 0.118
Test of group differences: $Q_b(1) = 7.16$, p = 0.01
Hba1c
≤7.5% 1 -6.0 (-9.2, -2.8) 0.000
>7.5% 3 0.2 (-1.3, 1.6) 0.826
Test of group differences: $Q_b(1) = 11.76$, p = 0.00
Risk of bias
low 3 0.2 (-1.3, 1.6) 0.826
some concerns 1 -6.0 (-9.2, -2.8) 0.000
Test of group differences: $Q_b(1) = 11.76$, p = 0.00
Overall -0.7 (-2.6, 1.2) 0.484
Heterogeneity: $\tau^2 = 2.75$, $I^2 = 84.03\%$, $H^2 = 6.26$
Test of $\theta_i = \theta_j$: Q(3) = 18.79, p = 0.00
Favours AID
Favours Control
-10
-5
0
5
10
Mean difference
Random-effects DerSimonian–Laird model

5.5 Sensitivity analyses TBR <54 mg/dl

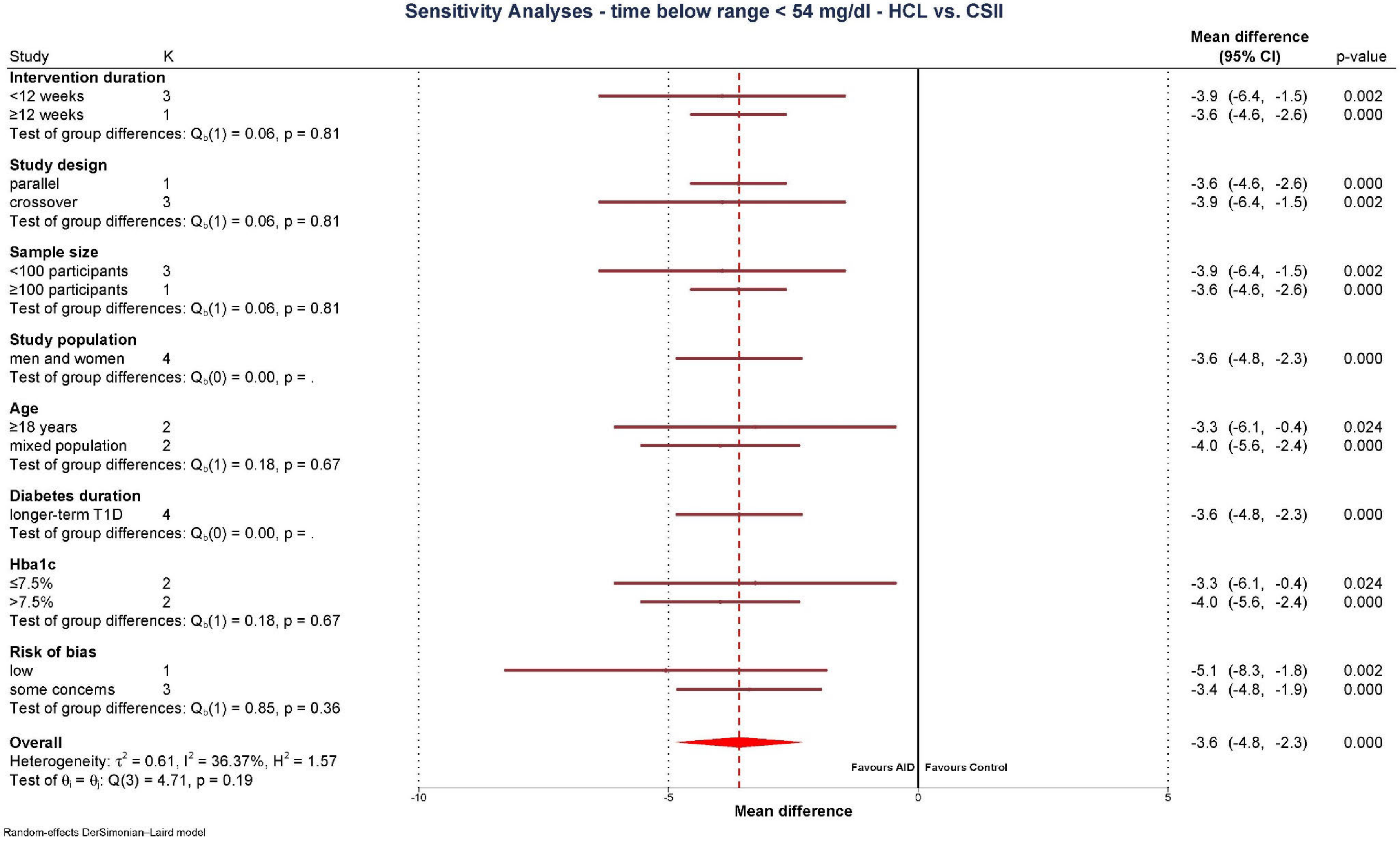

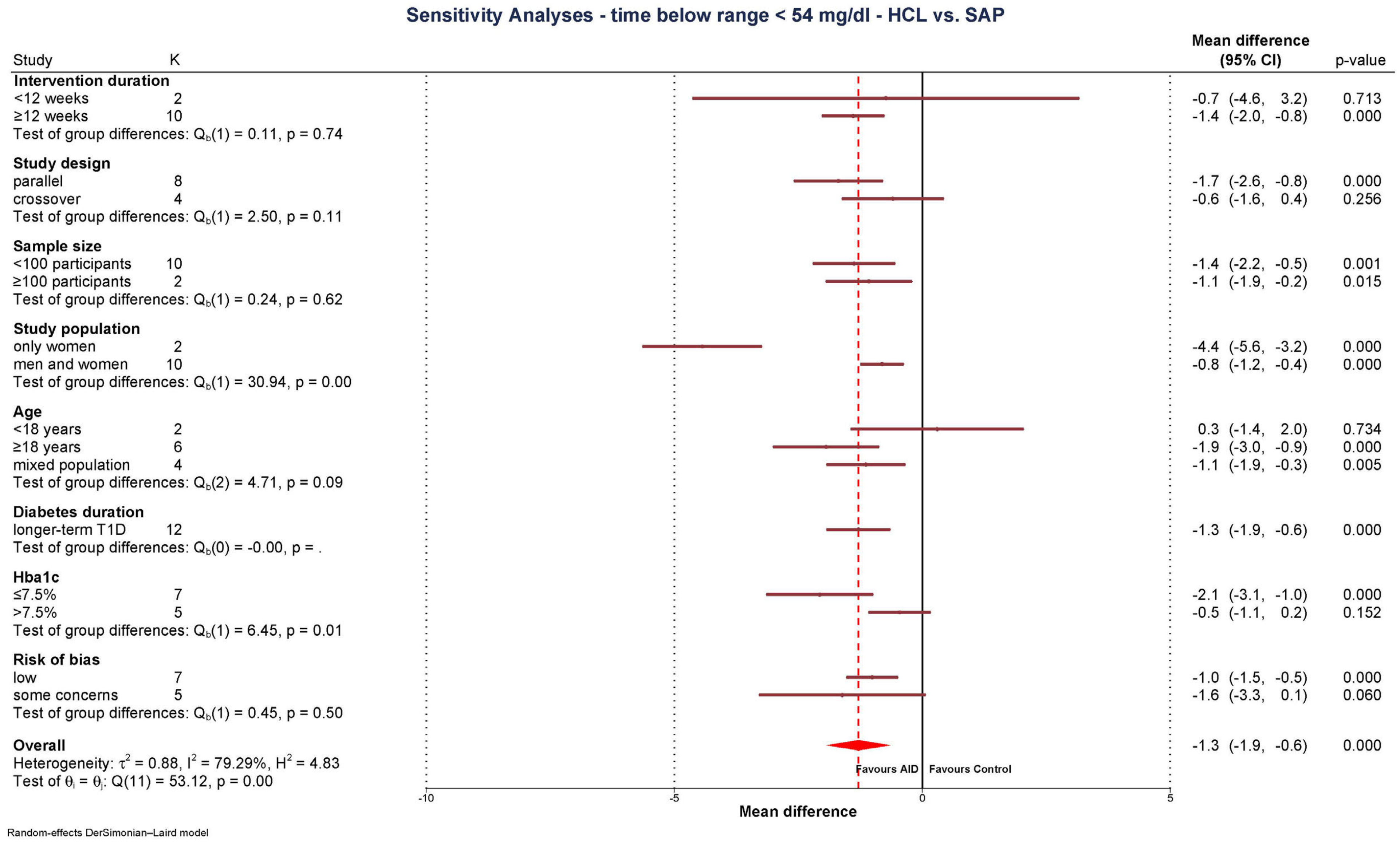
Sensitivity Analyses - time below range < 54 mg/dl - HCL vs. SAP
Study K
Mean difference (95% CI)
p-value
Intervention duration
<12 weeks 2 -0.7 (-4.6, 3.2) 0.713
≥12 weeks 10 -1.4 (-2.0, -0.8) 0.000
Test of group differences: $Q_b(1)$ = 0.11, p = 0.74
Study design
parallel 8 -1.7 (-2.6, -0.8) 0.000
crossover 4 -0.6 (-1.6, 0.4) 0.256
Test of group differences: $Q_b(1)$ = 2.50, p = 0.11
Sample size
<100 participants 10 -1.4 (-2.2, -0.5) 0.001
≥100 participants 2 -1.1 (-1.9, -0.2) 0.015
Test of group differences: $Q_b(1)$ = 0.24, p = 0.62
Study population
only women 2 -4.4 (-5.6, -3.2) 0.000
men and women 10 -0.8 (-1.2, -0.4) 0.000
Test of group differences: $Q_b(1)$ = 30.94, p = 0.00
Age
<18 years 2 0.3 (-1.4, 2.0) 0.734
≥18 years 6 -1.9 (-3.0, -0.9) 0.000
mixed population 4 -1.1 (-1.9, -0.3) 0.005
Test of group differences: $Q_b(2)$ = 4.71, p = 0.09
Diabetes duration
longer-term T1D 12 -1.3 (-1.9, -0.6) 0.000
Test of group differences: $Q_b(0)$ = -0.00, p = .
Hba1c
≤7.5% 7 -2.1 (-3.1, -1.0) 0.000
>7.5% 5 -0.5 (-1.1, 0.2) 0.152
Test of group differences: $Q_b(1)$ = 6.45, p = 0.01
Risk of bias
low 7 -1.0 (-1.5, -0.5) 0.000
some concerns 5 -1.6 (-3.3, 0.1) 0.060
Test of group differences: $Q_b(1)$ = 0.45, p = 0.50
Overall -1.3 (-1.9, -0.6) 0.000
Heterogeneity: τ^2 = 0.88, I^2 = 79.29%, H^2 = 4.83
Test of $\theta_i = \theta_j$: Q(11) = 53.12, p = 0.00
Favours AID
Favours Control
-10
-5
0
5
Mean difference
Random-effects DerSimonian–Laird model

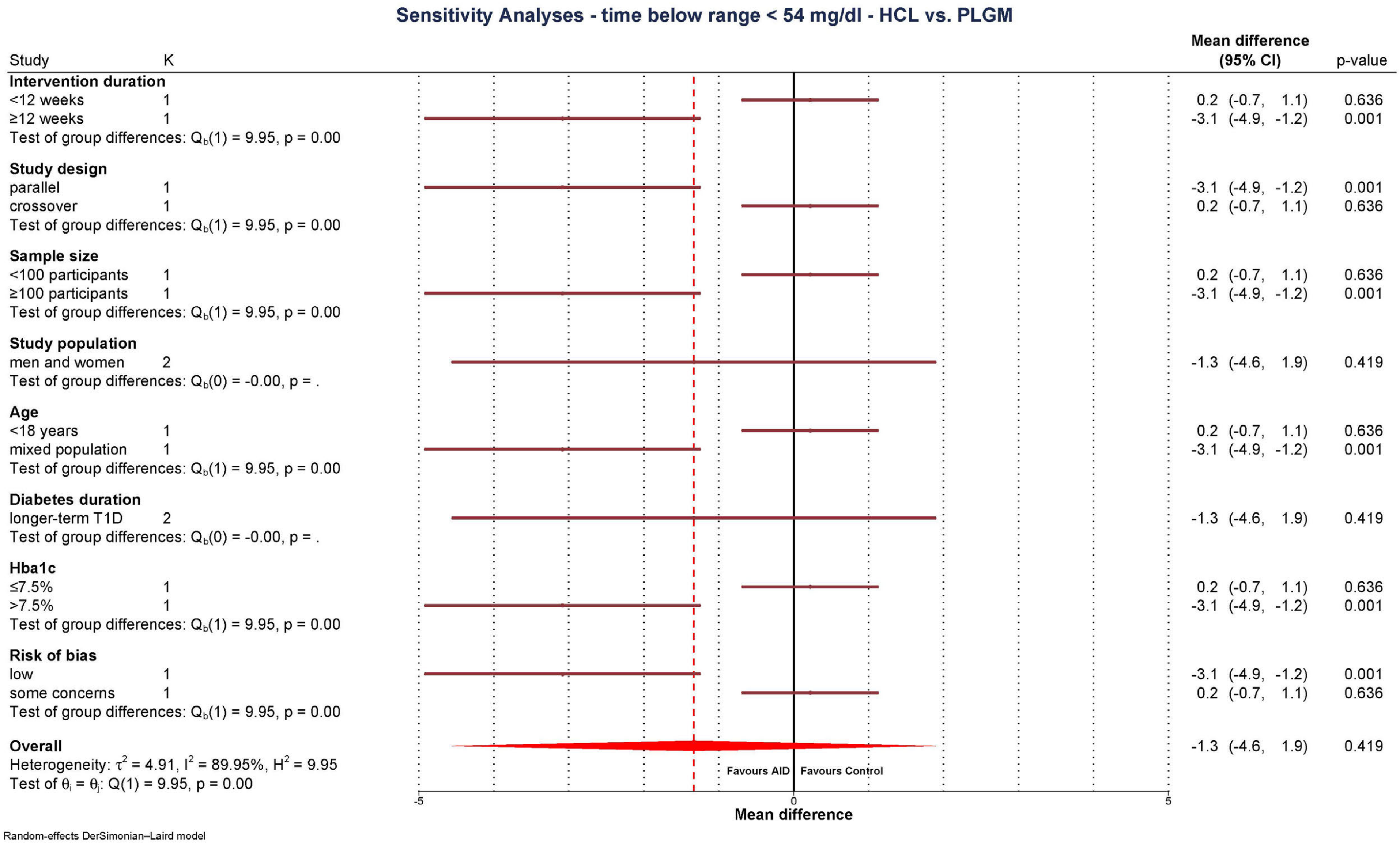
Sensitivity Analyses - time below range < 54 mg/dl - HCL vs. PLGM
Study
K
Mean difference (95% CI)
p-value
Intervention duration
<12 weeks 1 0.2 (-0.7, 1.1) 0.636
≥12 weeks 1 -3.1 (-4.9, -1.2) 0.001
Test of group differences: $Q_b(1)$ = 9.95, p = 0.00
Study design
parallel 1 -3.1 (-4.9, -1.2) 0.001
crossover 1 0.2 (-0.7, 1.1) 0.636
Test of group differences: $Q_b(1)$ = 9.95, p = 0.00
Sample size
<100 participants 1 0.2 (-0.7, 1.1) 0.636
≥100 participants 1 -3.1 (-4.9, -1.2) 0.001
Test of group differences: $Q_b(1)$ = 9.95, p = 0.00
Study population
men and women 2 -1.3 (-4.6, 1.9) 0.419
Test of group differences: $Q_b(0)$ = -0.00, p = .
Age
<18 years 1 0.2 (-0.7, 1.1) 0.636
mixed population 1 -3.1 (-4.9, -1.2) 0.001
Test of group differences: $Q_b(1)$ = 9.95, p = 0.00
Diabetes duration
longer-term T1D 2 -1.3 (-4.6, 1.9) 0.419
Test of group differences: $Q_b(0)$ = -0.00, p = .
Hba1c
≤7.5% 1 0.2 (-0.7, 1.1) 0.636
>7.5% 1 -3.1 (-4.9, -1.2) 0.001
Test of group differences: $Q_b(1)$ = 9.95, p = 0.00
Risk of bias
low 1 -3.1 (-4.9, -1.2) 0.001
some concerns 1 0.2 (-0.7, 1.1) 0.636
Test of group differences: $Q_b(1)$ = 9.95, p = 0.00
Overall -1.3 (-4.6, 1.9) 0.419
Heterogeneity: τ^2 = 4.91, I^2 = 89.95%, H^2 = 9.95
Test of $\theta_i = \theta_j$: Q(1) = 9.95, p = 0.00
Favours AID
Favours Control
-5
0
5
Mean difference
Random-effects DerSimonian–Laird model

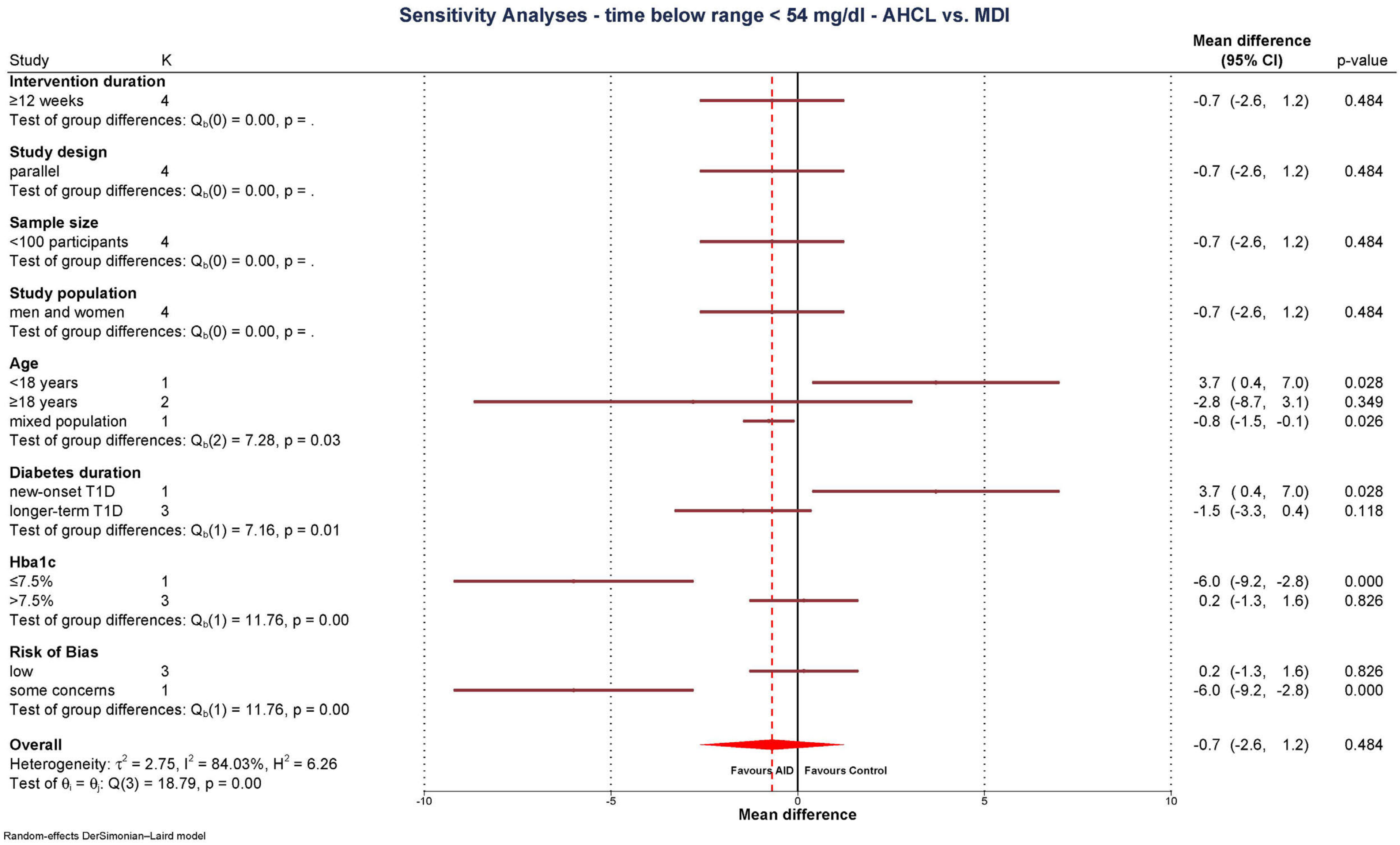
Sensitivity Analyses - time below range < 54 mg/dl - AHCL vs. MDI
Study K
Mean difference (95% CI)
p-value
Intervention duration
≥12 weeks 4 -0.7 (-2.6, 1.2) 0.484
Test of group differences: $Q_b(0) = 0.00$, p = .
Study design
parallel 4 -0.7 (-2.6, 1.2) 0.484
Test of group differences: $Q_b(0) = 0.00$, p = .
Sample size
<100 participants 4 -0.7 (-2.6, 1.2) 0.484
Test of group differences: $Q_b(0) = 0.00$, p = .
Study population
men and women 4 -0.7 (-2.6, 1.2) 0.484
Test of group differences: $Q_b(0) = 0.00$, p = .
Age
<18 years 1 3.7 (0.4, 7.0) 0.028
≥18 years 2 -2.8 (-8.7, 3.1) 0.349
mixed population 1 -0.8 (-1.5, -0.1) 0.026
Test of group differences: $Q_b(2) = 7.28$, p = 0.03
Diabetes duration
new-onset T1D 1 3.7 (0.4, 7.0) 0.028
longer-term T1D 3 -1.5 (-3.3, 0.4) 0.118
Test of group differences: $Q_b(1) = 7.16$, p = 0.01
Hba1c
≤7.5% 1 -6.0 (-9.2, -2.8) 0.000
>7.5% 3 0.2 (-1.3, 1.6) 0.826
Test of group differences: $Q_b(1) = 11.76$, p = 0.00
Risk of Bias
low 3 0.2 (-1.3, 1.6) 0.826
some concerns 1 -6.0 (-9.2, -2.8) 0.000
Test of group differences: $Q_b(1) = 11.76$, p = 0.00
Overall -0.7 (-2.6, 1.2) 0.484
Heterogeneity: $\tau^2 = 2.75$, $I^2 = 84.03\%$, $H^2 = 6.26$
Test of $\theta_i = \theta_j$: Q(3) = 18.79, p = 0.00
Favours AID
Favours Control
-10
-5
0
5
10
Mean difference
Random-effects DerSimonian–Laird model

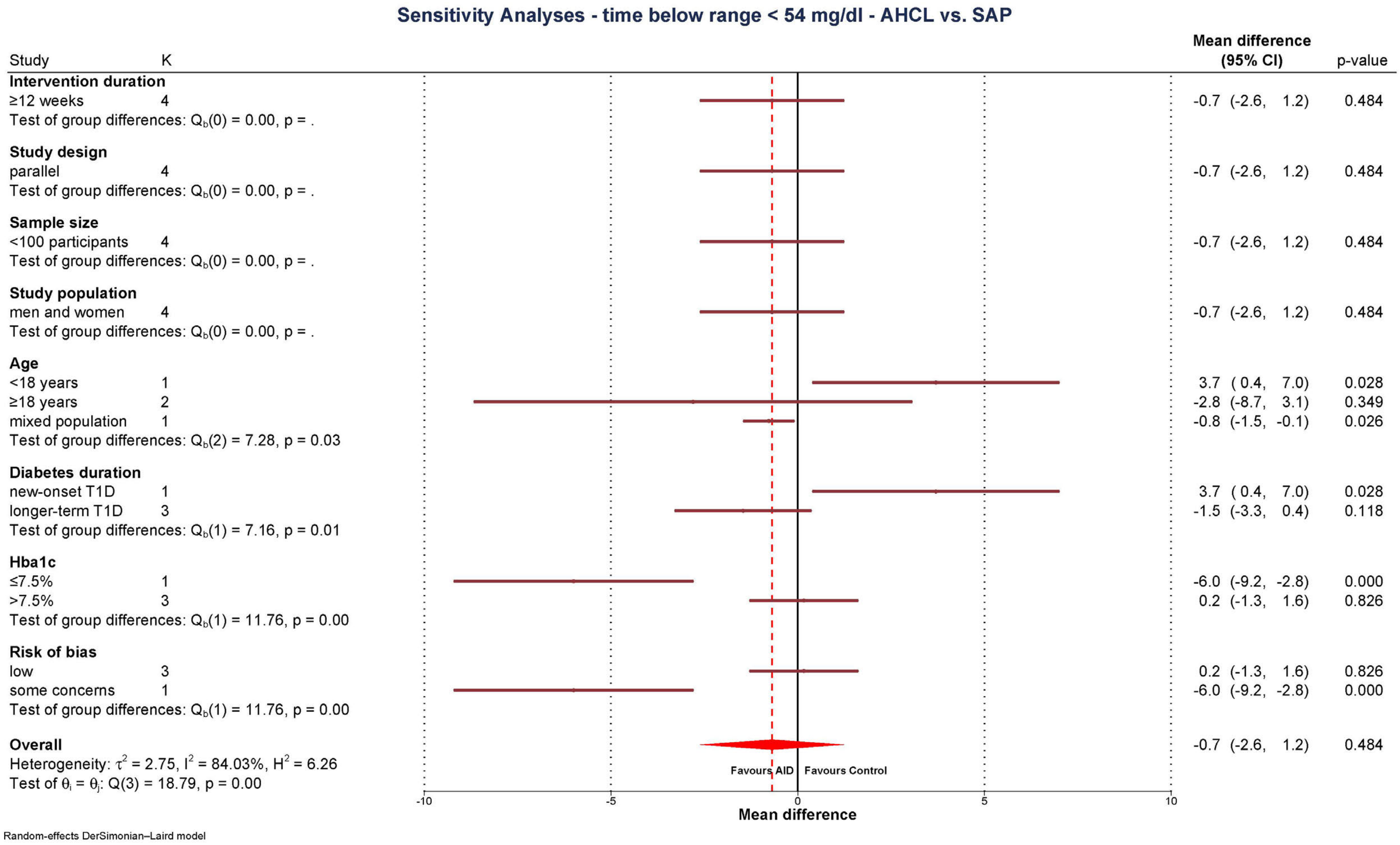

Sensitivity Analyses - time below range < 54 mg/dl - AHCL vs. SAP
Study K
Mean difference (95% CI)
p-value
Intervention duration
≥12 weeks 4 -0.7 (-2.6, 1.2) 0.484
Test of group differences: $Q_b(0)$ = 0.00, p = .
Study design
parallel 4 -0.7 (-2.6, 1.2) 0.484
Test of group differences: $Q_b(0)$ = 0.00, p = .
Sample size
<100 participants 4 -0.7 (-2.6, 1.2) 0.484
Test of group differences: $Q_b(0)$ = 0.00, p = .
Study population
men and women 4 -0.7 (-2.6, 1.2) 0.484
Test of group differences: $Q_b(0)$ = 0.00, p = .
Age
<18 years 1 3.7 (0.4, 7.0) 0.028
≥18 years 2 -2.8 (-8.7, 3.1) 0.349
mixed population 1 -0.8 (-1.5, -0.1) 0.026
Test of group differences: $Q_b(2)$ = 7.28, p = 0.03
Diabetes duration
new-onset T1D 1 3.7 (0.4, 7.0) 0.028
longer-term T1D 3 -1.5 (-3.3, 0.4) 0.118
Test of group differences: $Q_b(1)$ = 7.16, p = 0.01
Hba1c
≤7.5% 1 -6.0 (-9.2, -2.8) 0.000
>7.5% 3 0.2 (-1.3, 1.6) 0.826
Test of group differences: $Q_b(1)$ = 11.76, p = 0.00
Risk of bias
low 3 0.2 (-1.3, 1.6) 0.826
some concerns 1 -6.0 (-9.2, -2.8) 0.000
Test of group differences: $Q_b(1)$ = 11.76, p = 0.00
Overall -0.7 (-2.6, 1.2) 0.484
Heterogeneity: τ^2 = 2.75, I^2 = 84.03%, H^2 = 6.26
Test of $\theta_i = \theta_j$: Q(3) = 18.79, p = 0.00
Favours AID
Favours Control
-10
-5
0
5
10
Mean difference
Random-effects DerSimonian–Laird model

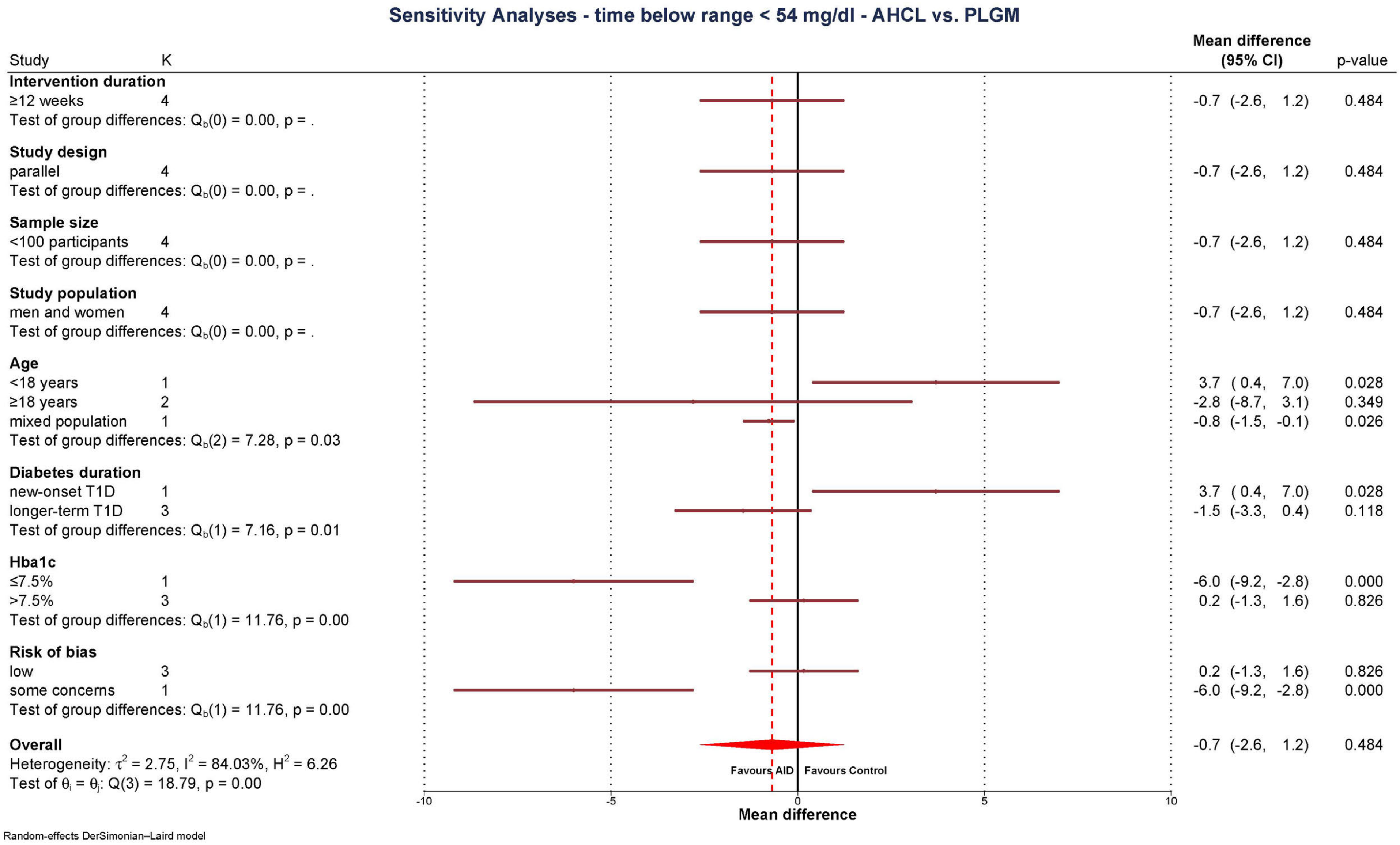
Sensitivity Analyses - time below range < 54 mg/dl - AHCL vs. PLGM
Study K
Mean difference (95% CI)
p-value
Intervention duration
≥12 weeks 4 -0.7 (-2.6, 1.2) 0.484
Test of group differences: Qb(0) = 0.00, p = .
Study design
parallel 4 -0.7 (-2.6, 1.2) 0.484
Test of group differences: Qb(0) = 0.00, p = .
Sample size
<100 participants 4 -0.7 (-2.6, 1.2) 0.484
Test of group differences: Qb(0) = 0.00, p = .
Study population
men and women 4 -0.7 (-2.6, 1.2) 0.484
Test of group differences: Qb(0) = 0.00, p = .
Age
<18 years 1 3.7 (0.4, 7.0) 0.028
≥18 years 2 -2.8 (-8.7, 3.1) 0.349
mixed population 1 -0.8 (-1.5, -0.1) 0.026
Test of group differences: Qb(2) = 7.28, p = 0.03
Diabetes duration
new-onset T1D 1 3.7 (0.4, 7.0) 0.028
longer-term T1D 3 -1.5 (-3.3, 0.4) 0.118
Test of group differences: Qb(1) = 7.16, p = 0.01
Hba1c
≤7.5% 1 -6.0 (-9.2, -2.8) 0.000
>7.5% 3 0.2 (-1.3, 1.6) 0.826
Test of group differences: Qb(1) = 11.76, p = 0.00
Risk of bias
low 3 0.2 (-1.3, 1.6) 0.826
some concerns 1 -6.0 (-9.2, -2.8) 0.000
Test of group differences: Qb(1) = 11.76, p = 0.00
Overall -0.7 (-2.6, 1.2) 0.484
Heterogeneity: τ2 = 2.75, I2 = 84.03%, H2 = 6.26
Test of θi = θj: Q(3) = 18.79, p = 0.00
Favours AID
Favours Control
-10
-5
0
5
10
Mean difference
Random-effects DerSimonian–Laird model

Sensitivity Analyses - time below range < 54 mg/dl - AHCL vs. HCL

Study	K	Mean difference (95% CI)	p-value
**Intervention duration**			
≥12 weeks	4	-0.7 (-2.6, 1.2)	0.484
Test of group differences: $Q_b(0) = 0.00$, p = .			
**Study design**			
parallel	4	-0.7 (-2.6, 1.2)	0.484
Test of group differences: $Q_b(0) = 0.00$, p = .			
**Sample size**			
<100 participants	4	-0.7 (-2.6, 1.2)	0.484
Test of group differences: $Q_b(0) = 0.00$, p = .			
**Study population**			
men and women	4	-0.7 (-2.6, 1.2)	0.484
Test of group differences: $Q_b(0) = 0.00$, p = .			
**Age**			
<18 years	1	3.7 (0.4, 7.0)	0.028
≥18 years	2	-2.8 (-8.7, 3.1)	0.349
mixed population	1	-0.8 (-1.5, -0.1)	0.026
Test of group differences: $Q_b(2) = 7.28$, p = 0.03			
**Diabetes duration**			
new-onset T1D	1	3.7 (0.4, 7.0)	0.028
longer-term T1D	3	-1.5 (-3.3, 0.4)	0.118
Test of group differences: $Q_b(1) = 7.16$, p = 0.01			
**Hba1c**			
≤7.5%	1	-6.0 (-9.2, -2.8)	0.000
>7.5%	3	0.2 (-1.3, 1.6)	0.826
Test of group differences: $Q_b(1) = 11.76$, p = 0.00			
**Risk of bias**			
low	3	0.2 (-1.3, 1.6)	0.826
some concerns	1	-6.0 (-9.2, -2.8)	0.000
Test of group differences: $Q_b(1) = 11.76$, p = 0.00			
**Overall**		-0.7 (-2.6, 1.2)	0.484
Heterogeneity: $\tau^2 = 2.75$, $I^2 = 84.03\%$, $H^2 = 6.26$			
Test of $\theta_i = \theta_j$: $Q(3) = 18.79$, p = 0.00			

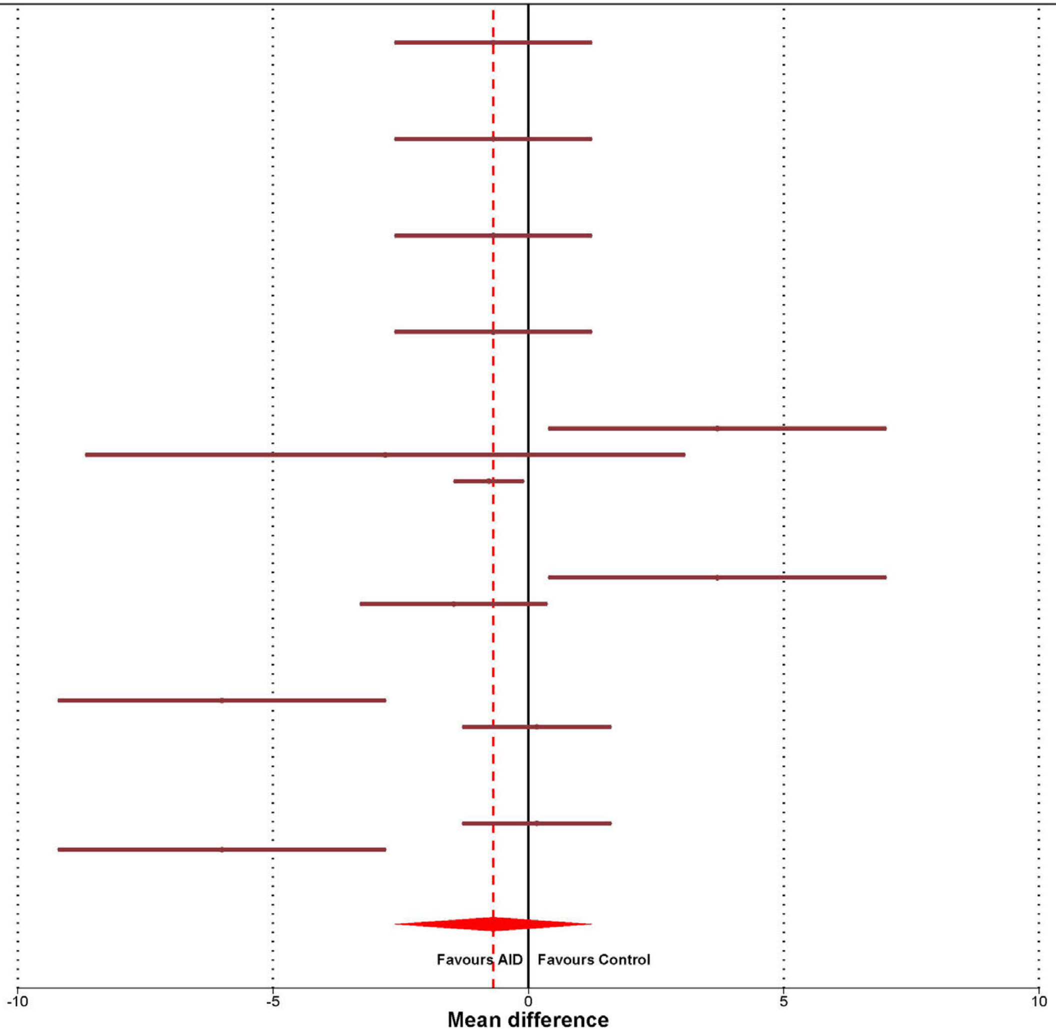

Random-effects DerSimonian–Laird model

5.6 Sensitivity analyses HbA1c

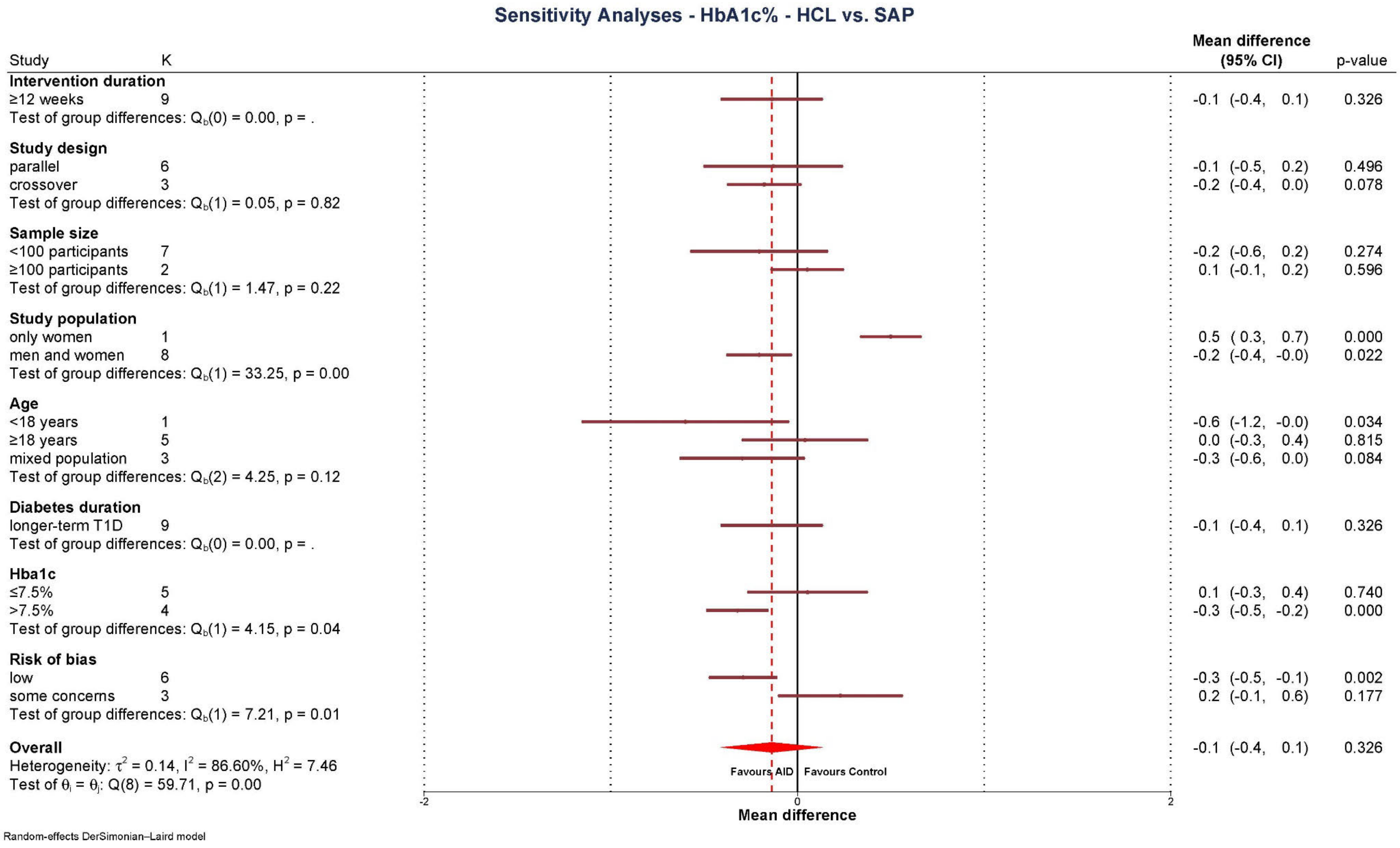

Sensitivity Analyses - HbA1c% - AHCL vs. MDI

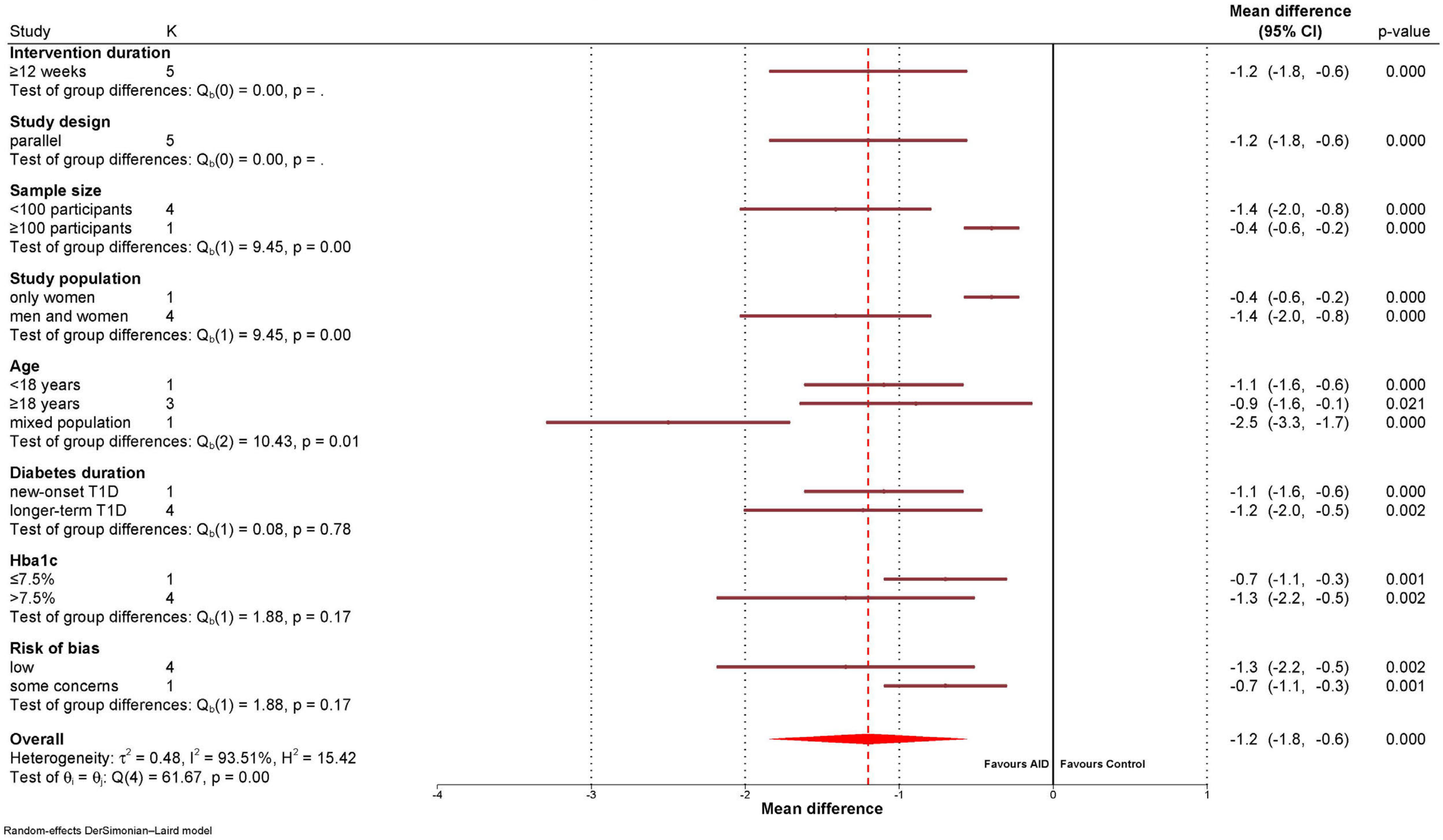

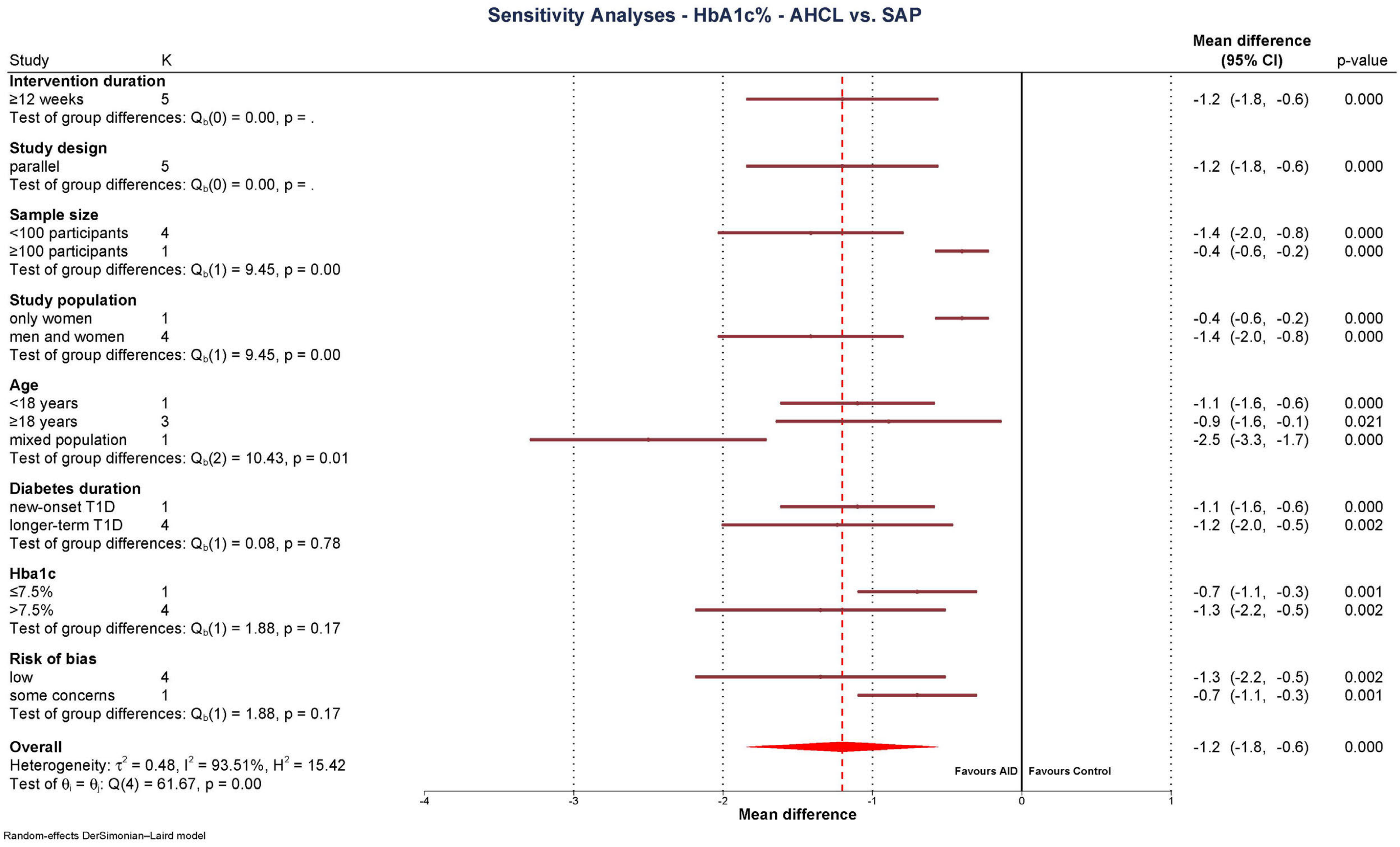
Sensitivity Analyses - HbA1c% - AHCL vs. SAP
Study K
Mean difference (95% CI)
p-value
Intervention duration
≥12 weeks 5 -1.2 (-1.8, -0.6) 0.000
Test of group differences: $Q_b(0)$ = 0.00, p = .
Study design
parallel 5 -1.2 (-1.8, -0.6) 0.000
Test of group differences: $Q_b(0)$ = 0.00, p = .
Sample size
<100 participants 4 -1.4 (-2.0, -0.8) 0.000
≥100 participants 1 -0.4 (-0.6, -0.2) 0.000
Test of group differences: $Q_b(1)$ = 9.45, p = 0.00
Study population
only women 1 -0.4 (-0.6, -0.2) 0.000
men and women 4 -1.4 (-2.0, -0.8) 0.000
Test of group differences: $Q_b(1)$ = 9.45, p = 0.00
Age
<18 years 1 -1.1 (-1.6, -0.6) 0.000
≥18 years 3 -0.9 (-1.6, -0.1) 0.021
mixed population 1 -2.5 (-3.3, -1.7) 0.000
Test of group differences: $Q_b(2)$ = 10.43, p = 0.01
Diabetes duration
new-onset T1D 1 -1.1 (-1.6, -0.6) 0.000
longer-term T1D 4 -1.2 (-2.0, -0.5) 0.002
Test of group differences: $Q_b(1)$ = 0.08, p = 0.78
Hba1c
≤7.5% 1 -0.7 (-1.1, -0.3) 0.001
>7.5% 4 -1.3 (-2.2, -0.5) 0.002
Test of group differences: $Q_b(1)$ = 1.88, p = 0.17
Risk of bias
low 4 -1.3 (-2.2, -0.5) 0.002
some concerns 1 -0.7 (-1.1, -0.3) 0.001
Test of group differences: $Q_b(1)$ = 1.88, p = 0.17
Overall -1.2 (-1.8, -0.6) 0.000
Heterogeneity: τ^2 = 0.48, I^2 = 93.51%, H^2 = 15.42
Test of $\theta_i = \theta_j$: Q(4) = 61.67, p = 0.00
Favours AID
Favours Control
-4
-3
-2
-1
0
1
Mean difference
Random-effects DerSimonian–Laird model

Sensitivity Analyses - HbA1c% - AHCL vs. PLGM

Study	K	Mean difference (95% CI)	p-value
**Intervention duration**			
≥12 weeks	5	-1.2 (-1.8, -0.6)	0.000
Test of group differences: $Q_b(0) = 0.00$, p = .			
**Study design**			
parallel	5	-1.2 (-1.8, -0.6)	0.000
Test of group differences: $Q_b(0) = 0.00$, p = .			
**Sample size**			
<100 participants	4	-1.4 (-2.0, -0.8)	0.000
≥100 participants	1	-0.4 (-0.6, -0.2)	0.000
Test of group differences: $Q_b(1) = 9.45$, p = 0.00			
**Study population**			
only women	1	-0.4 (-0.6, -0.2)	0.000
men and women	4	-1.4 (-2.0, -0.8)	0.000
Test of group differences: $Q_b(1) = 9.45$, p = 0.00			
**Age**			
<18 years	1	-1.1 (-1.6, -0.6)	0.000
≥18 years	3	-0.9 (-1.6, -0.1)	0.021
mixed population	1	-2.5 (-3.3, -1.7)	0.000
Test of group differences: $Q_b(2) = 10.43$, p = 0.01			
**Diabetes duration**			
new-onset T1D	1	-1.1 (-1.6, -0.6)	0.000
longer-term T1D	4	-1.2 (-2.0, -0.5)	0.002
Test of group differences: $Q_b(1) = 0.08$, p = 0.78			
**Hba1c**			
≤7.5%	1	-0.7 (-1.1, -0.3)	0.001
>7.5%	4	-1.3 (-2.2, -0.5)	0.002
Test of group differences: $Q_b(1) = 1.88$, p = 0.17			
**Risk of bias**			
low	4	-1.3 (-2.2, -0.5)	0.002
some concerns	1	-0.7 (-1.1, -0.3)	0.001
Test of group differences: $Q_b(1) = 1.88$, p = 0.17			
**Overall**		-1.2 (-1.8, -0.6)	0.000
Heterogeneity: $\tau^2 = 0.48$, $I^2 = 93.51\%$, $H^2 = 15.42$			
Test of $\theta_i = \theta_j$: $Q(4) = 61.67$, p = 0.00			

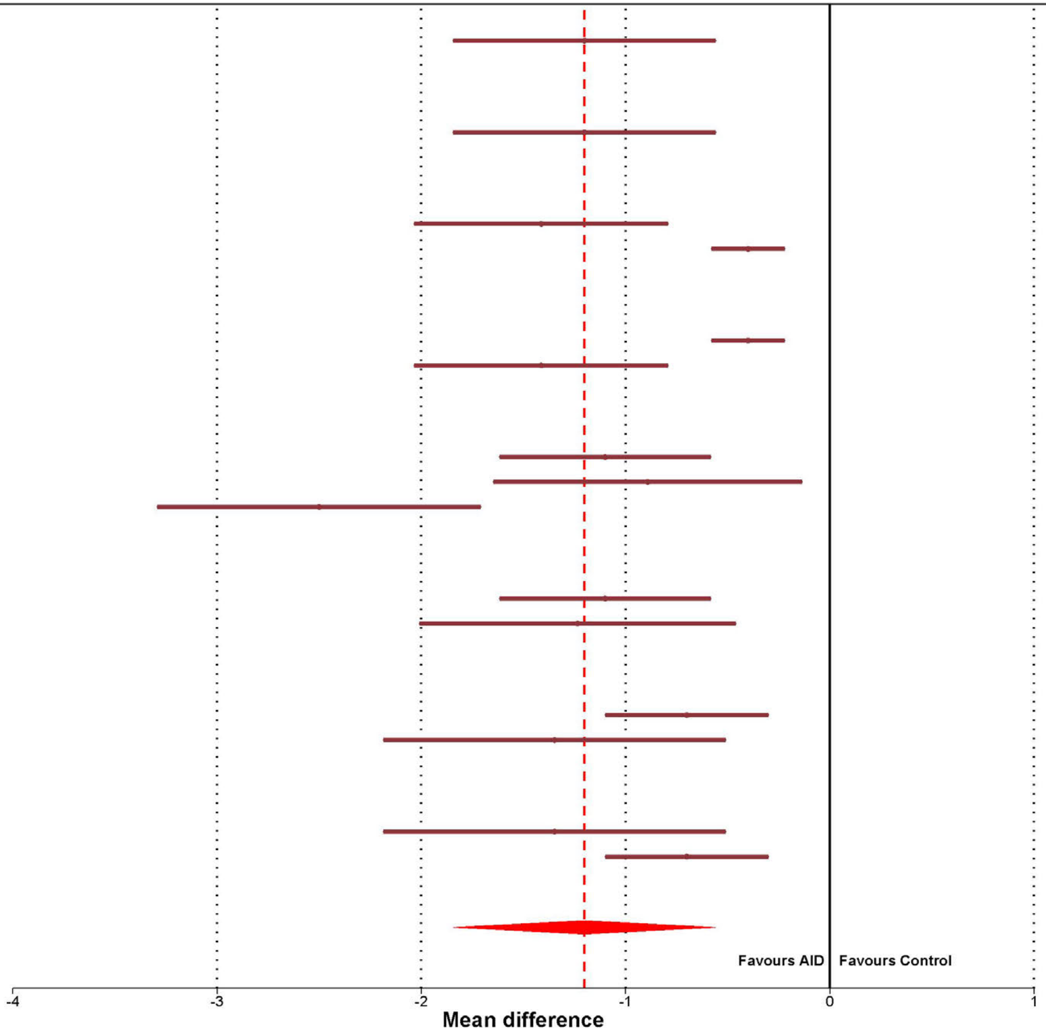

Random-effects DerSimonian–Laird model

Sensitivity Analyses - HbA1c% - AHCL vs. HCL

Study	K	Mean difference (95% CI)	p-value
**Intervention duration**			
≥12 weeks	5	-1.2 (-1.8, -0.6)	0.000
Test of group differences: $Q_b(0) = 0.00$, p = .			
**Study design**			
parallel	5	-1.2 (-1.8, -0.6)	0.000
Test of group differences: $Q_b(0) = 0.00$, p = .			
**Sample size**			
<100 participants	4	-1.4 (-2.0, -0.8)	0.000
≥100 participants	1	-0.4 (-0.6, -0.2)	0.000
Test of group differences: $Q_b(1) = 9.45$, p = 0.00			
**Study population**			
only women	1	-0.4 (-0.6, -0.2)	0.000
men and women	4	-1.4 (-2.0, -0.8)	0.000
Test of group differences: $Q_b(1) = 9.45$, p = 0.00			
**Age**			
<18 years	1	-1.1 (-1.6, -0.6)	0.000
≥18 years	3	-0.9 (-1.6, -0.1)	0.021
mixed population	1	-2.5 (-3.3, -1.7)	0.000
Test of group differences: $Q_b(2) = 10.43$, p = 0.01			
**Diabetes duration**			
new-onset T1D	1	-1.1 (-1.6, -0.6)	0.000
longer-term T1D	4	-1.2 (-2.0, -0.5)	0.002
Test of group differences: $Q_b(1) = 0.08$, p = 0.78			
**Hba1c**			
≤7.5%	1	-0.7 (-1.1, -0.3)	0.001
>7.5%	4	-1.3 (-2.2, -0.5)	0.002
Test of group differences: $Q_b(1) = 1.88$, p = 0.17			
**Risk of bias**			
low	4	-1.3 (-2.2, -0.5)	0.002
some concerns	1	-0.7 (-1.1, -0.3)	0.001
Test of group differences: $Q_b(1) = 1.88$, p = 0.17			
**Overall**		-1.2 (-1.8, -0.6)	0.000
Heterogeneity: $\tau^2 = 0.48$, $I^2 = 93.51\%$, $H^2 = 15.42$			
Test of $\theta_i = \theta_j$: $Q(4) = 61.67$, p = 0.00			

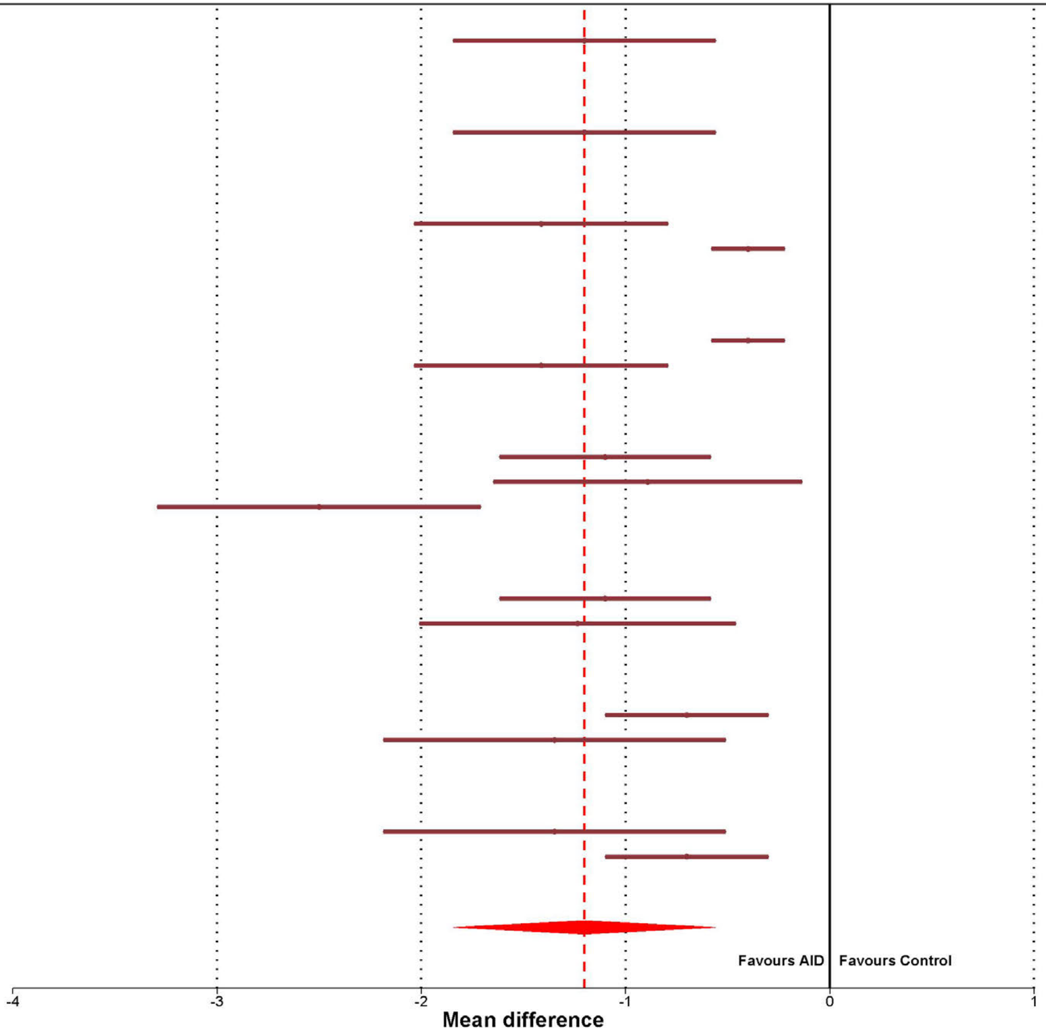

Random-effects DerSimonian–Laird model

6 Supplement to leave-one-out analysis

6.1 Leave-one-out TIR

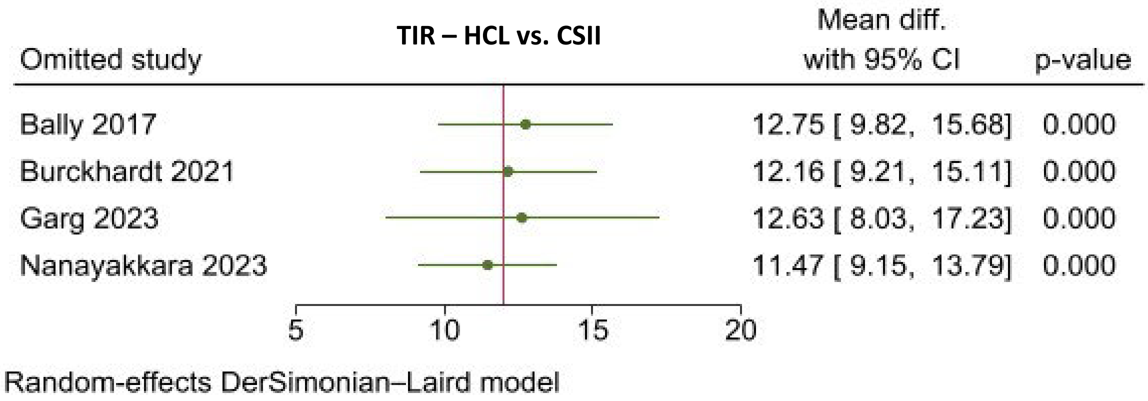

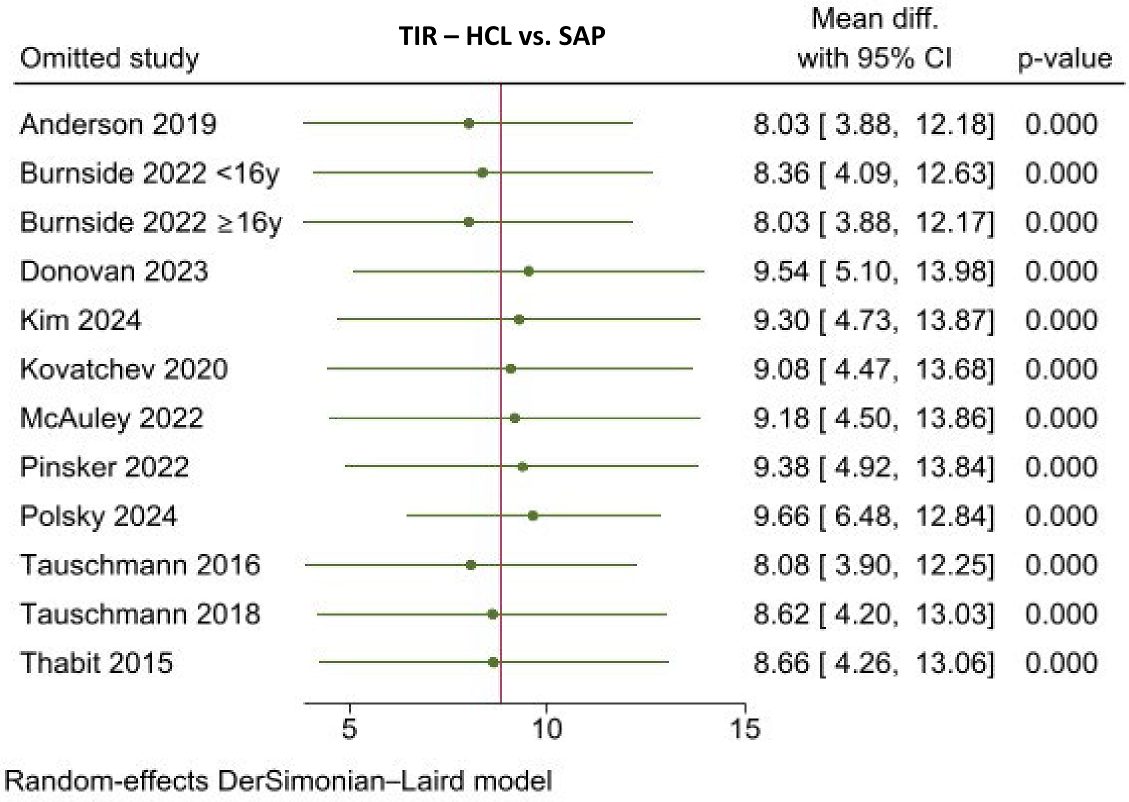

TIR – HCL vs. PLGM

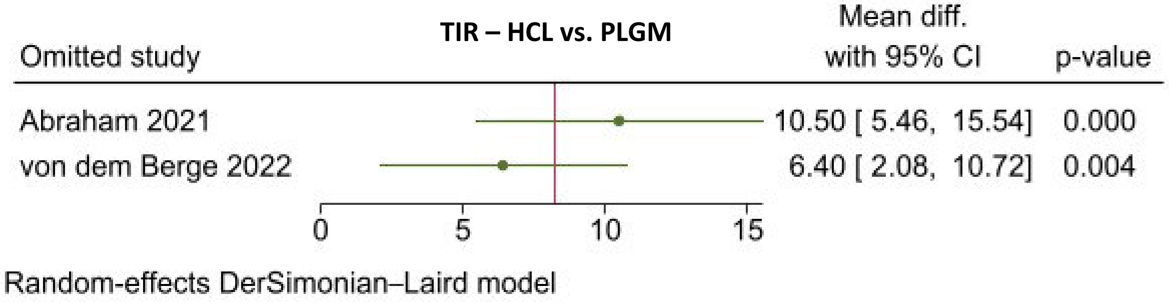

TIR – AHCL vs. MDI

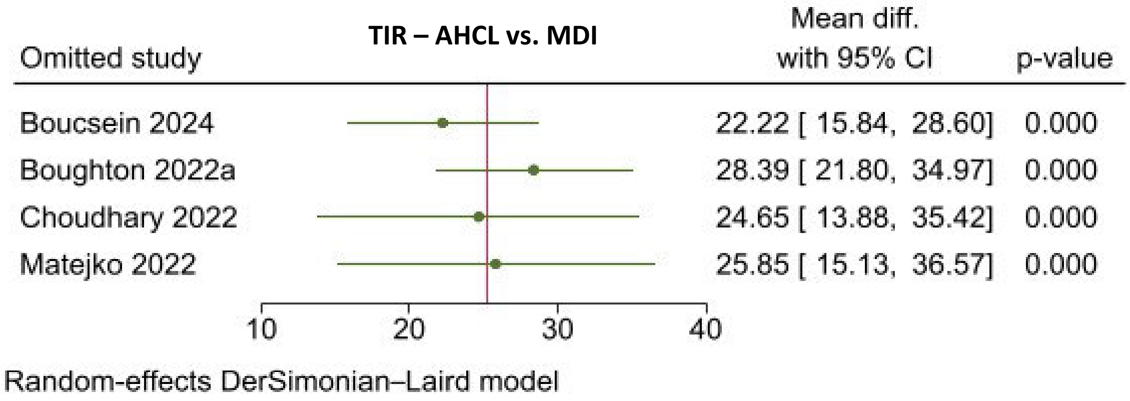

TIR – AHCL vs. SAP

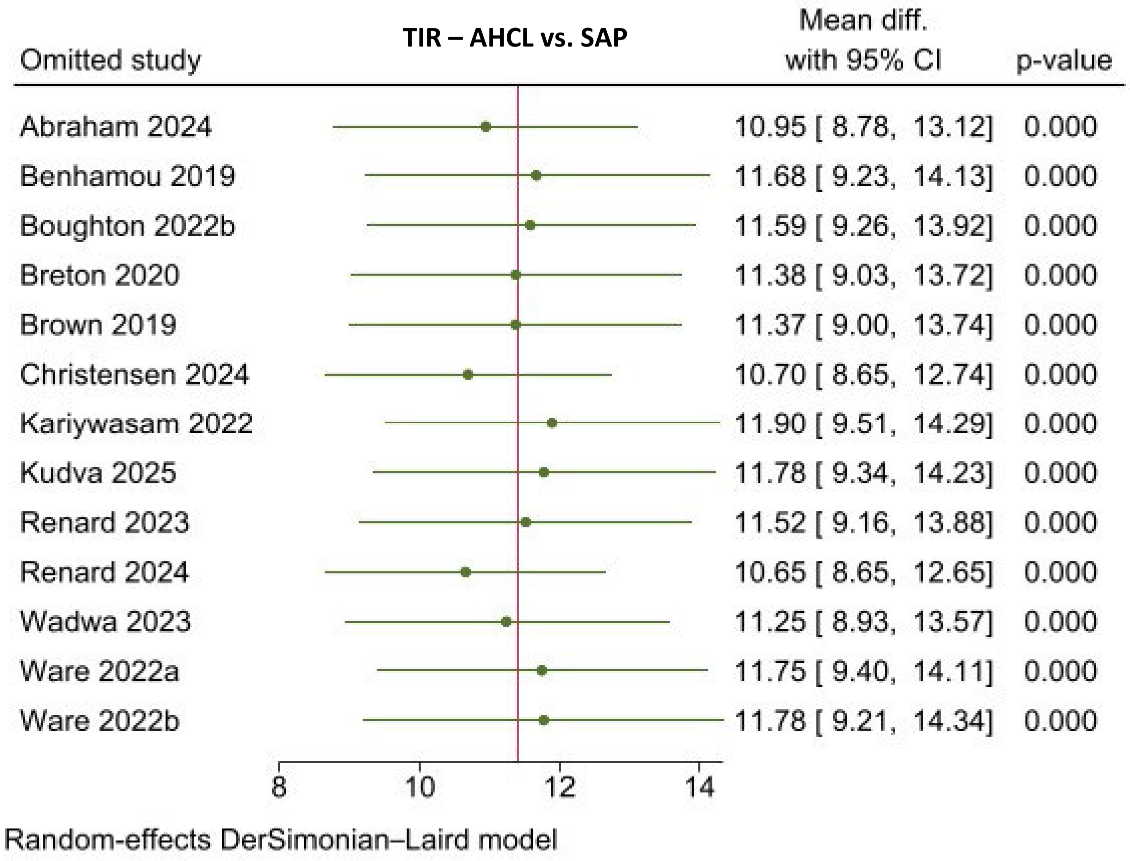

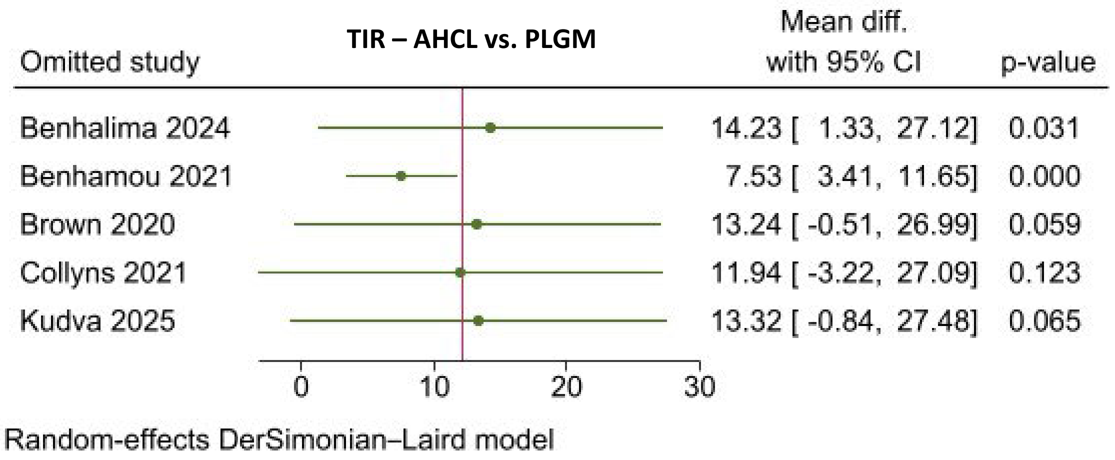

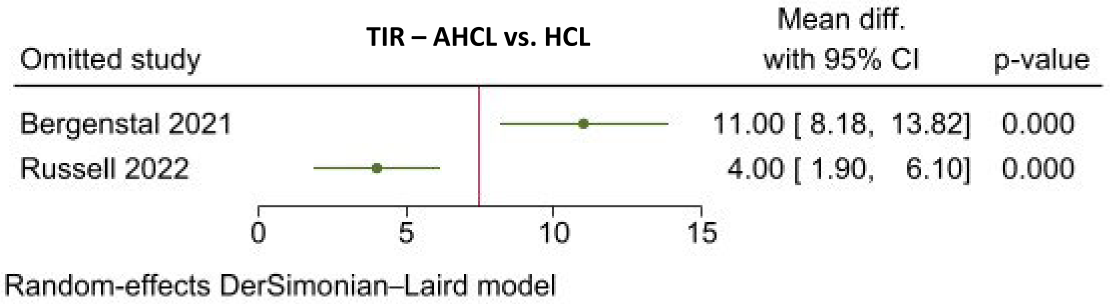

6.2 Leave-one-out TAR >180 mg/dl

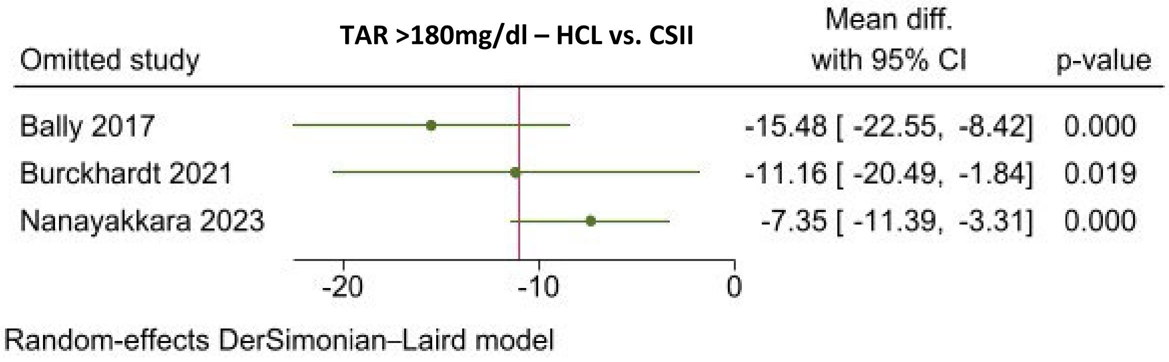

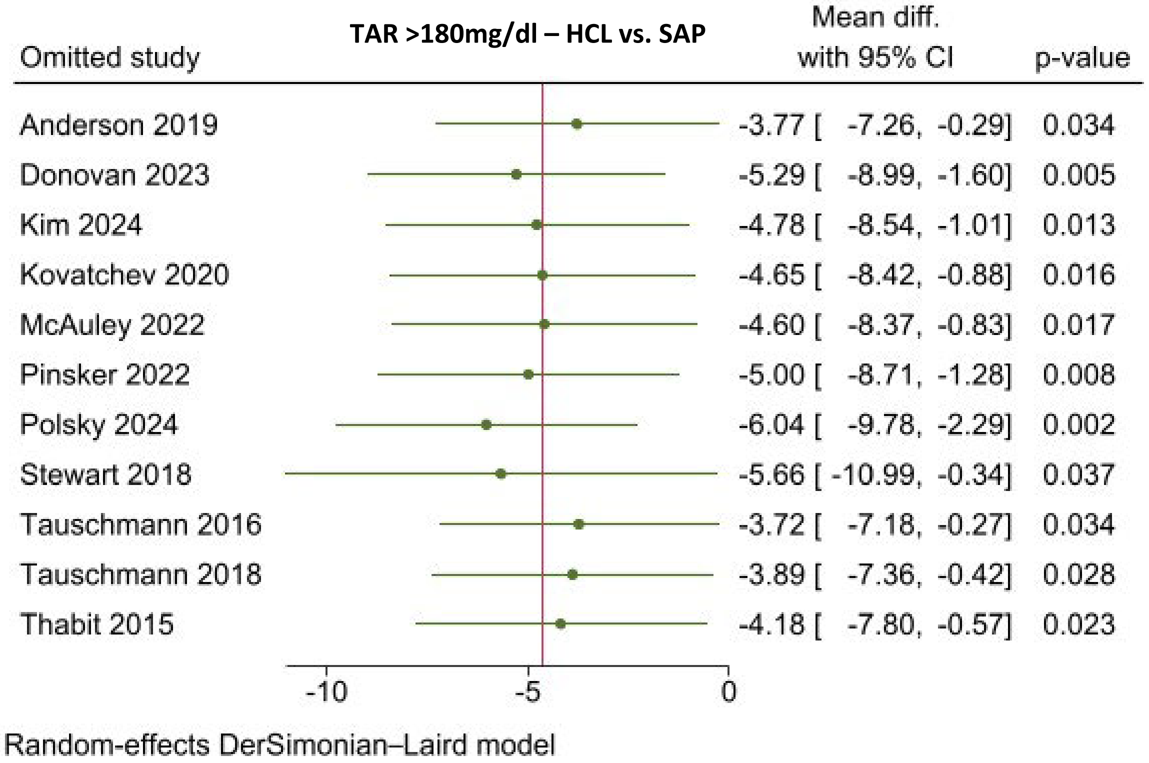
TAR >180mg/dl – HCL vs. SAP
Omitted study
Mean diff. with 95% CI
p-value
Anderson 2019 -3.77 [-7.26, -0.29] 0.034
Donovan 2023 -5.29 [-8.99, -1.60] 0.005
Kim 2024 -4.78 [-8.54, -1.01] 0.013
Kovatchev 2020 -4.65 [-8.42, -0.88] 0.016
McAuley 2022 -4.60 [-8.37, -0.83] 0.017
Pinsker 2022 -5.00 [-8.71, -1.28] 0.008
Polsky 2024 -6.04 [-9.78, -2.29] 0.002
Stewart 2018 -5.66 [-10.99, -0.34] 0.037
Tauschmann 2016 -3.72 [-7.18, -0.27] 0.034
Tauschmann 2018 -3.89 [-7.36, -0.42] 0.028
Thabit 2015 -4.18 [-7.80, -0.57] 0.023
-10
-5
0
Random-effects DerSimonian–Laird model

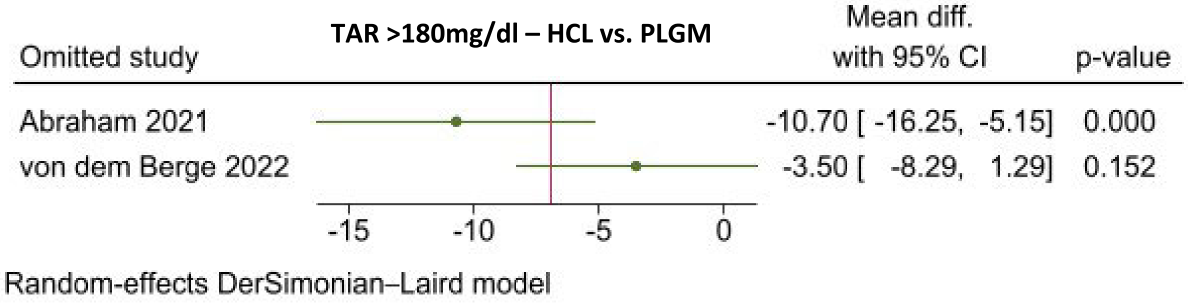
TAR >180mg/dl – HCL vs. PLGM
Omitted study
Mean diff. with 95% CI
p-value
Abraham 2021 -10.70 [-16.25, -5.15] 0.000
von dem Berge 2022 -3.50 [-8.29, 1.29] 0.152
-15
-10
-5
0
Random-effects DerSimonian–Laird model

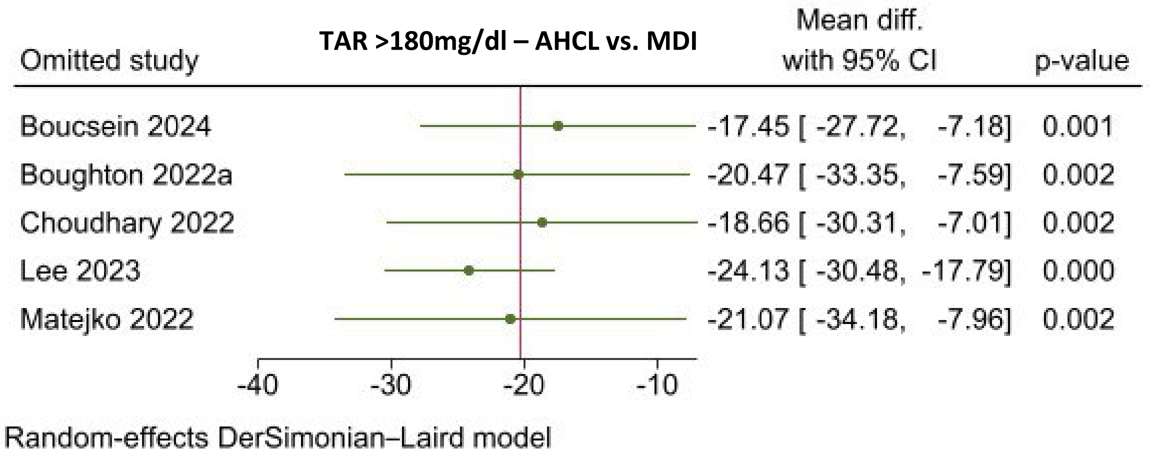
TAR >180mg/dl – AHCL vs. MDI
Omitted study
Mean diff. with 95% CI
p-value
Boucsein 2024 -17.45 [-27.72, -7.18] 0.001
Boughton 2022a -20.47 [-33.35, -7.59] 0.002
Choudhary 2022 -18.66 [-30.31, -7.01] 0.002
Lee 2023 -24.13 [-30.48, -17.79] 0.000
Matejko 2022 -21.07 [-34.18, -7.96] 0.002
-40
-30
-20
-10
Random-effects DerSimonian–Laird model

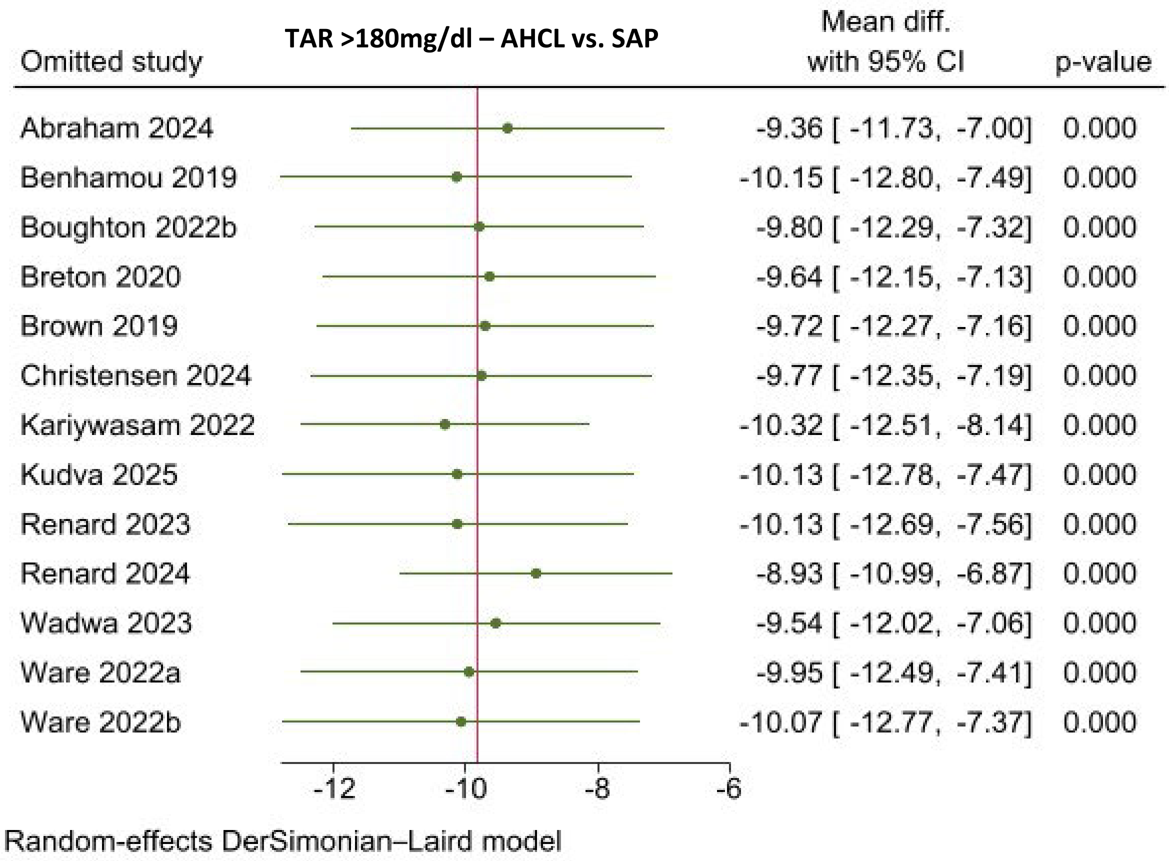
TAR >180mg/dl – AHCL vs. SAP
Omitted study
Mean diff. with 95% CI
p-value
Abraham 2024 -9.36 [-11.73, -7.00] 0.000
Benhamou 2019 -10.15 [-12.80, -7.49] 0.000
Boughton 2022b -9.80 [-12.29, -7.32] 0.000
Breton 2020 -9.64 [-12.15, -7.13] 0.000
Brown 2019 -9.72 [-12.27, -7.16] 0.000
Christensen 2024 -9.77 [-12.35, -7.19] 0.000
Kariywasam 2022 -10.32 [-12.51, -8.14] 0.000
Kudva 2025 -10.13 [-12.78, -7.47] 0.000
Renard 2023 -10.13 [-12.69, -7.56] 0.000
Renard 2024 -8.93 [-10.99, -6.87] 0.000
Wadwa 2023 -9.54 [-12.02, -7.06] 0.000
Ware 2022a -9.95 [-12.49, -7.41] 0.000
Ware 2022b -10.07 [-12.77, -7.37] 0.000
-12
-10
-8
-6
Random-effects DerSimonian–Laird model

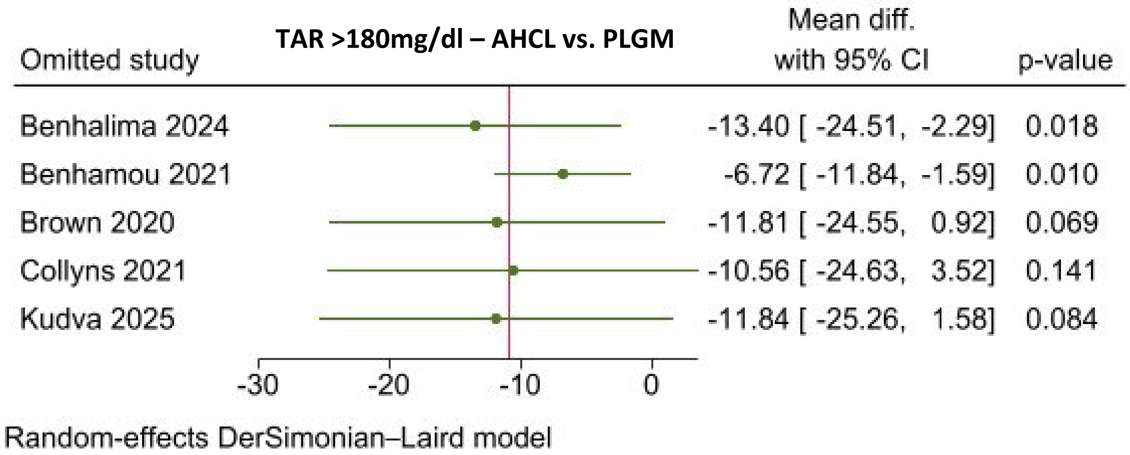
TAR >180mg/dl – AHCL vs. PLGM
Omitted study
Mean diff. with 95% CI
p-value
Benhalima 2024 -13.40 [-24.51, -2.29] 0.018
Benhamou 2021 -6.72 [-11.84, -1.59] 0.010
Brown 2020 -11.81 [-24.55, 0.92] 0.069
Collyns 2021 -10.56 [-24.63, 3.52] 0.141
Kudva 2025 -11.84 [-25.26, 1.58] 0.084
-30
-20
-10
0
Random-effects DerSimonian–Laird model

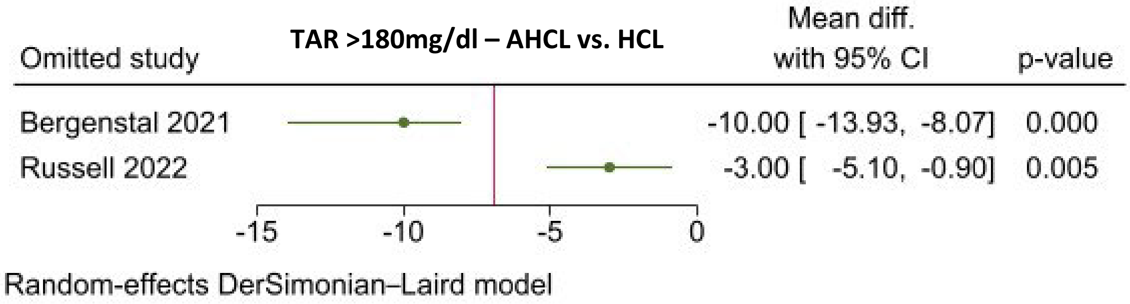
TAR >180mg/dl – AHCL vs. HCL
Omitted study
Mean diff. with 95% CI
p-value
Bergenstal 2021 -10.00 [-13.93, -8.07] 0.000
Russell 2022 -3.00 [-5.10, -0.90] 0.005
-15
-10
-5
0
Random-effects DerSimonian–Laird model

6.3 Leave-one-out TAR >250 mg/dl

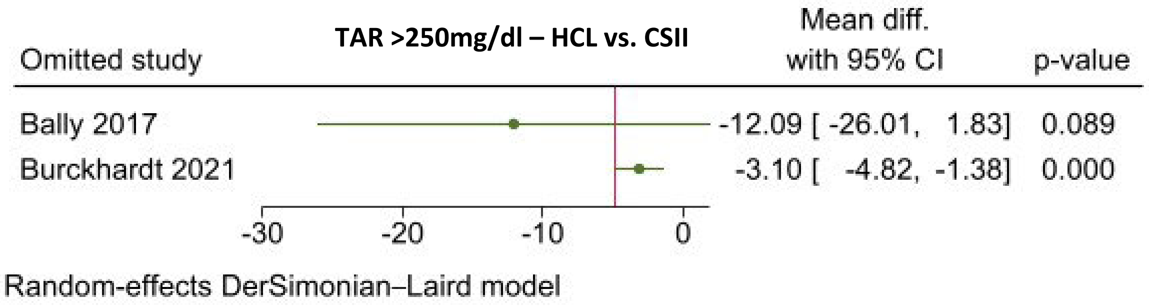

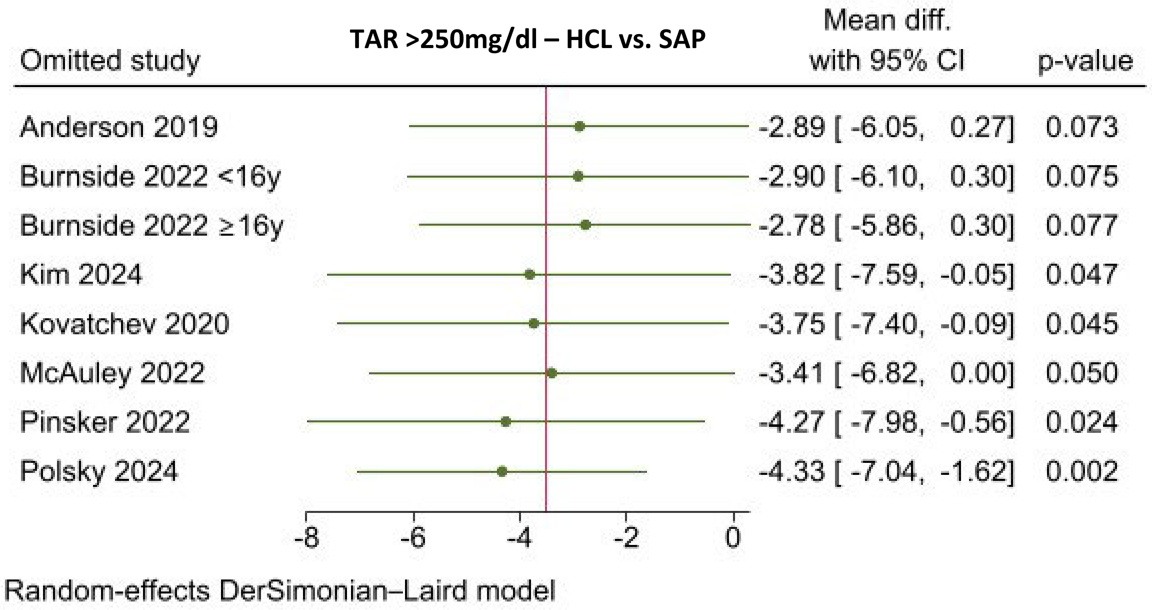

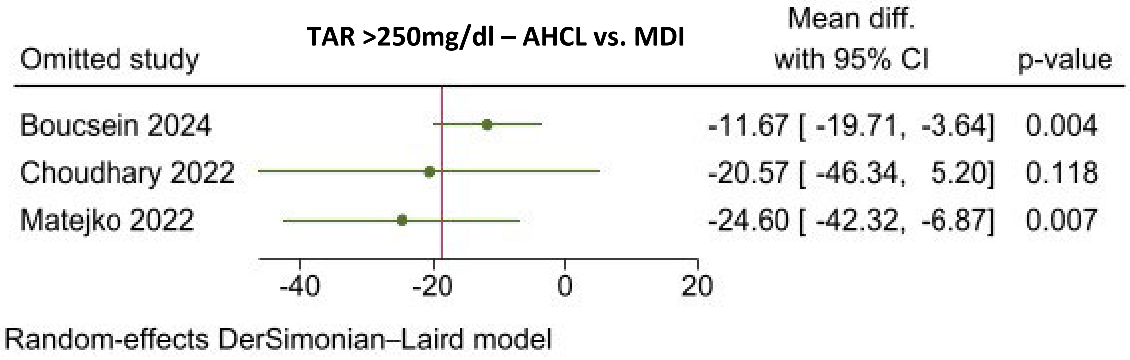

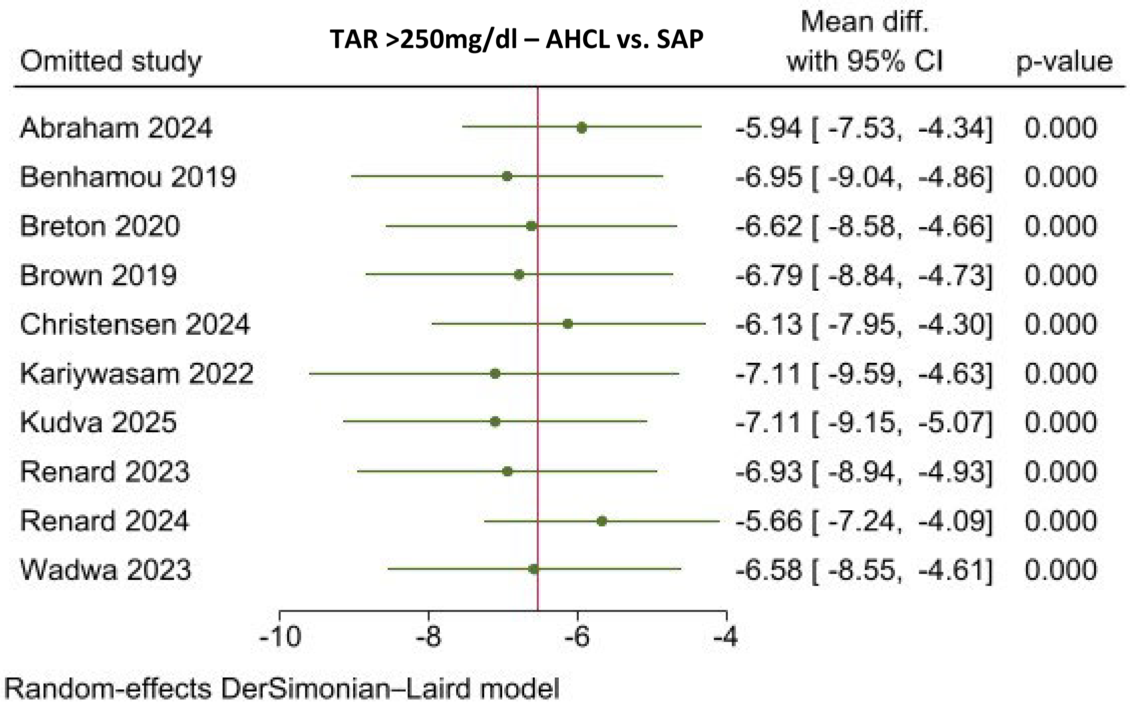
TAR >250mg/dl – AHCL vs. SAP
Omitted study
Mean diff. with 95% CI
p-value
Abraham 2024 -5.94 [-7.53, -4.34] 0.000
Benhamou 2019 -6.95 [-9.04, -4.86] 0.000
Breton 2020 -6.62 [-8.58, -4.66] 0.000
Brown 2019 -6.79 [-8.84, -4.73] 0.000
Christensen 2024 -6.13 [-7.95, -4.30] 0.000
Kariywasam 2022 -7.11 [-9.59, -4.63] 0.000
Kudva 2025 -7.11 [-9.15, -5.07] 0.000
Renard 2023 -6.93 [-8.94, -4.93] 0.000
Renard 2024 -5.66 [-7.24, -4.09] 0.000
Wadwa 2023 -6.58 [-8.55, -4.61] 0.000
-10
-8
-6
-4
Random-effects DerSimonian–Laird model

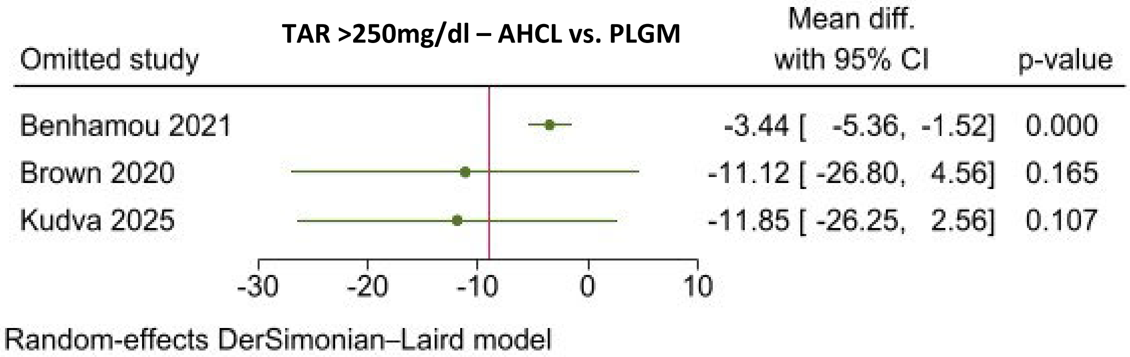
TAR >250mg/dl – AHCL vs. PLGM
Omitted study
Mean diff. with 95% CI
p-value
Benhamou 2021 -3.44 [-5.36, -1.52] 0.000
Brown 2020 -11.12 [-26.80, 4.56] 0.165
Kudva 2025 -11.85 [-26.25, 2.56] 0.107
-30
-20
-10
0
10
Random-effects DerSimonian–Laird model

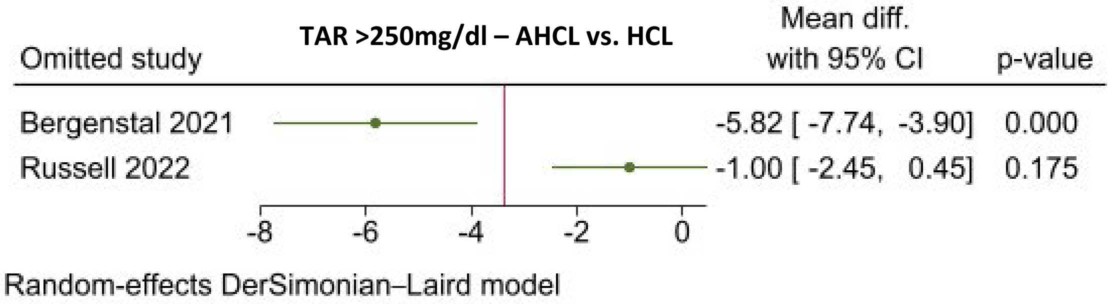
TAR >250mg/dl – AHCL vs. HCL
Omitted study
Mean diff. with 95% CI
p-value
Bergenstal 2021 -5.82 [-7.74, -3.90] 0.000
Russell 2022 -1.00 [-2.45, 0.45] 0.175
-8
-6
-4
-2
0
Random-effects DerSimonian–Laird model

6.4 Leave-one-out TBR <70 mg/dl

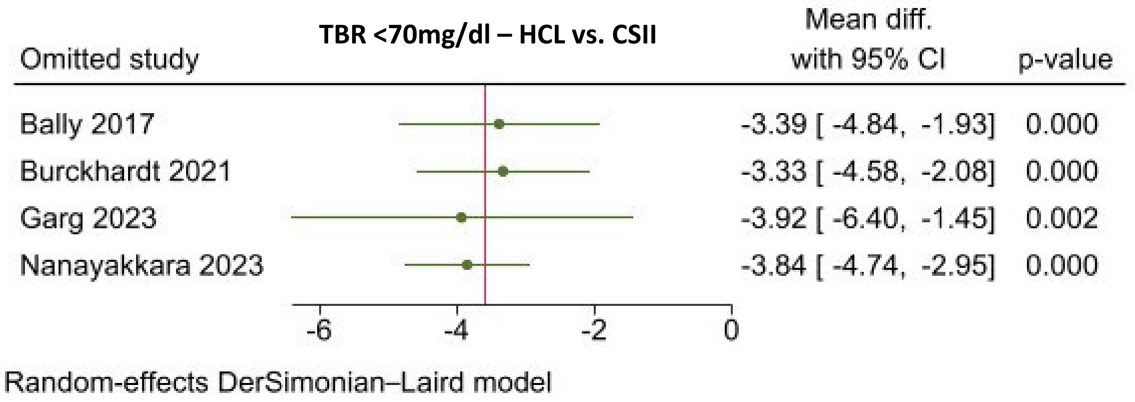

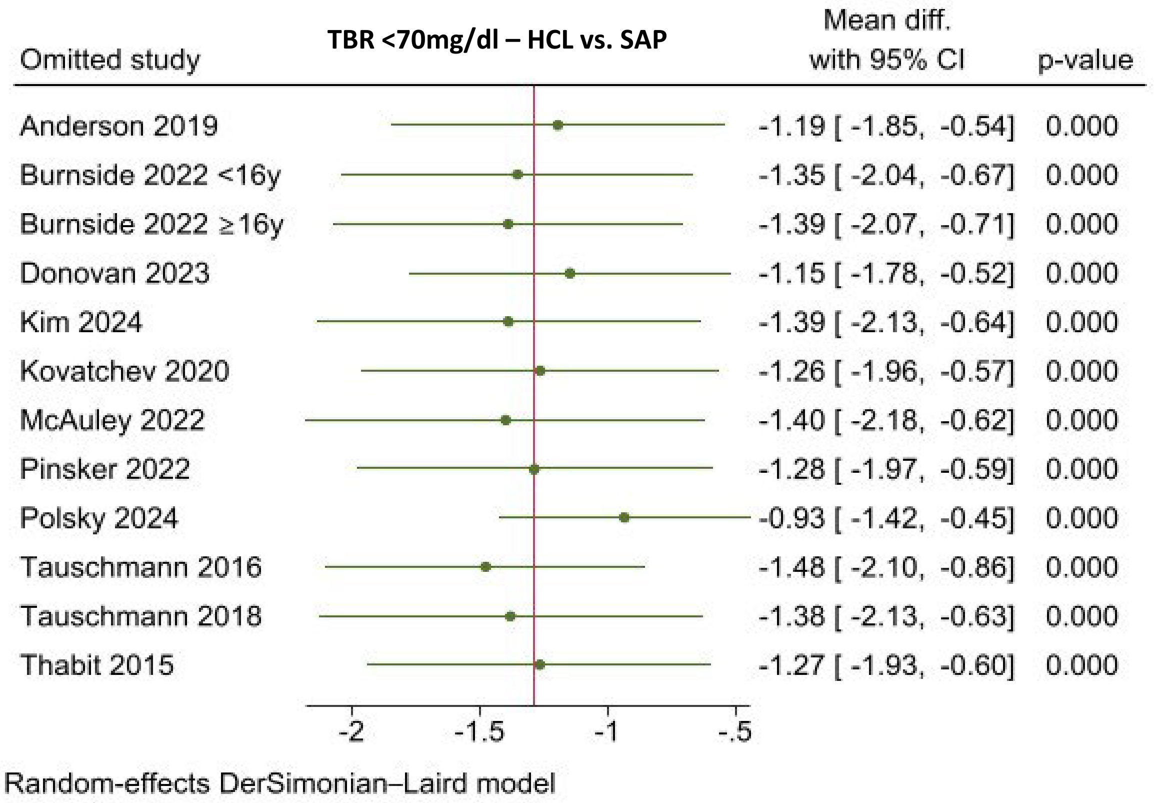

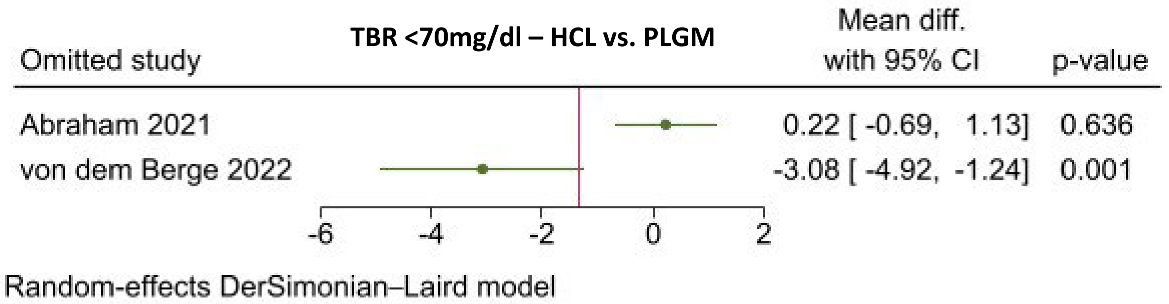

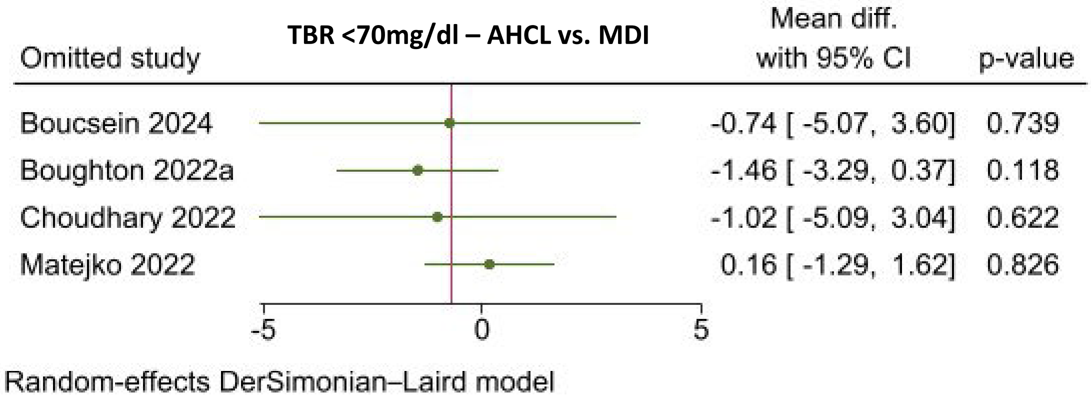
TBR <70mg/dl – AHCL vs. MDI
Omitted study
Mean diff. with 95% CI
p-value
Boucsein 2024 -0.74 [-5.07, 3.60] 0.739
Boughton 2022a -1.46 [-3.29, 0.37] 0.118
Choudhary 2022 -1.02 [-5.09, 3.04] 0.622
Matejko 2022 0.16 [-1.29, 1.62] 0.826
-5
0
5
Random-effects DerSimonian–Laird model

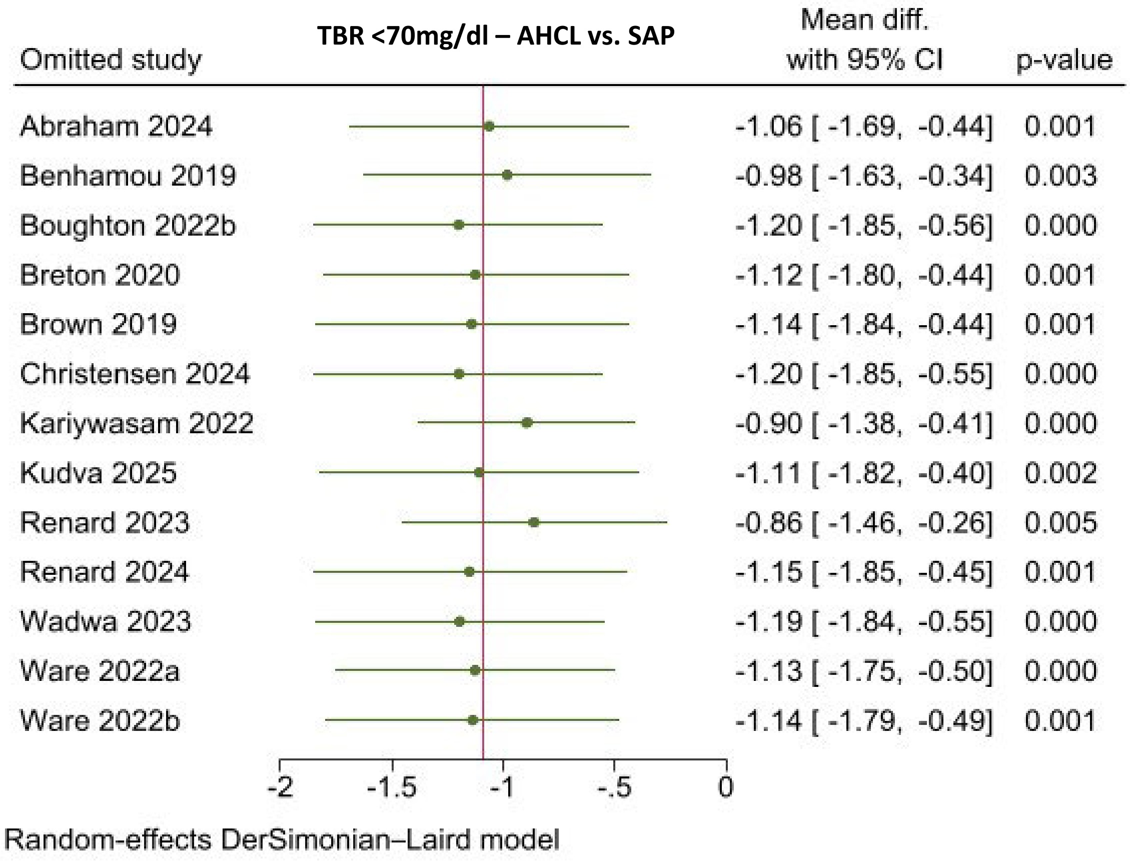
TBR <70mg/dl – AHCL vs. SAP
Omitted study
Mean diff. with 95% CI
p-value
Abraham 2024 -1.06 [-1.69, -0.44] 0.001
Benhamou 2019 -0.98 [-1.63, -0.34] 0.003
Boughton 2022b -1.20 [-1.85, -0.56] 0.000
Breton 2020 -1.12 [-1.80, -0.44] 0.001
Brown 2019 -1.14 [-1.84, -0.44] 0.001
Christensen 2024 -1.20 [-1.85, -0.55] 0.000
Kariywasam 2022 -0.90 [-1.38, -0.41] 0.000
Kudva 2025 -1.11 [-1.82, -0.40] 0.002
Renard 2023 -0.86 [-1.46, -0.26] 0.005
Renard 2024 -1.15 [-1.85, -0.45] 0.001
Wadwa 2023 -1.19 [-1.84, -0.55] 0.000
Ware 2022a -1.13 [-1.75, -0.50] 0.000
Ware 2022b -1.14 [-1.79, -0.49] 0.001
-2
-1.5
-1
-.5
0
Random-effects DerSimonian–Laird model

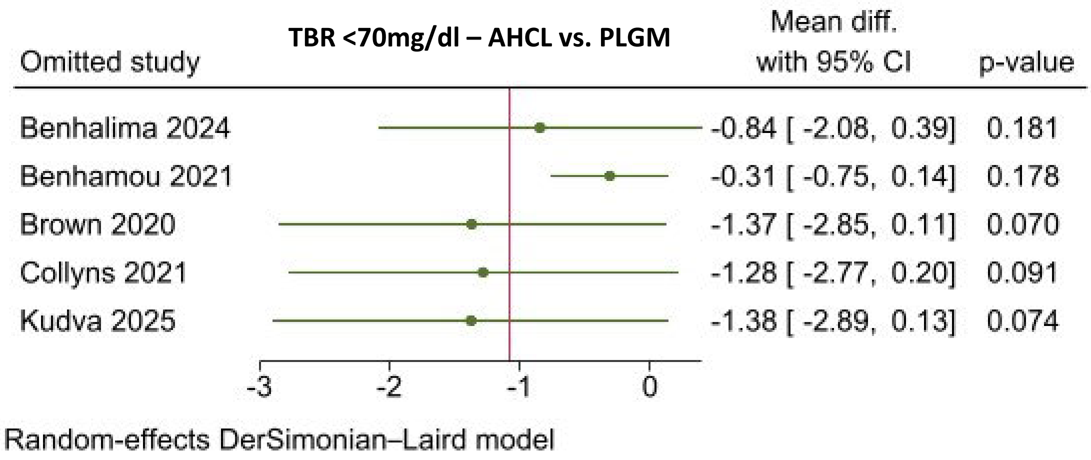

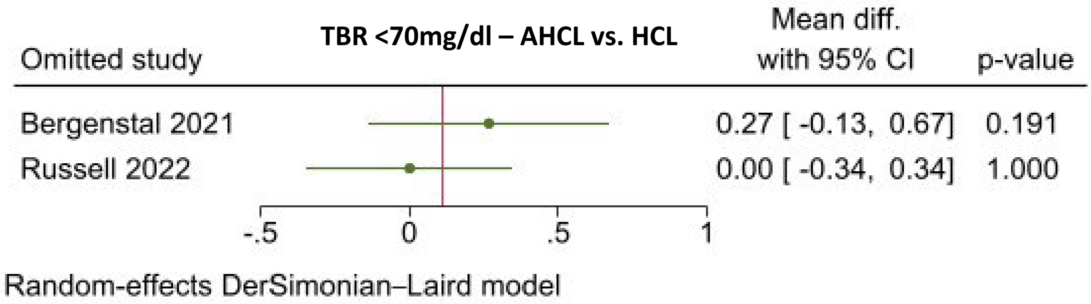

6.5 Leave-one-out TBR <54 mg/dl

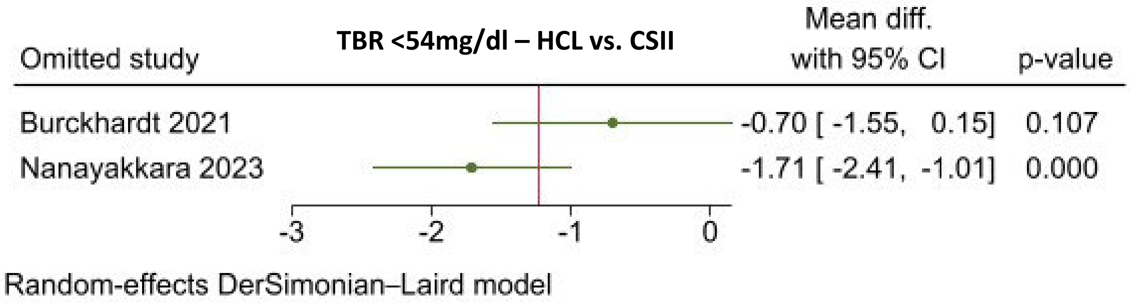

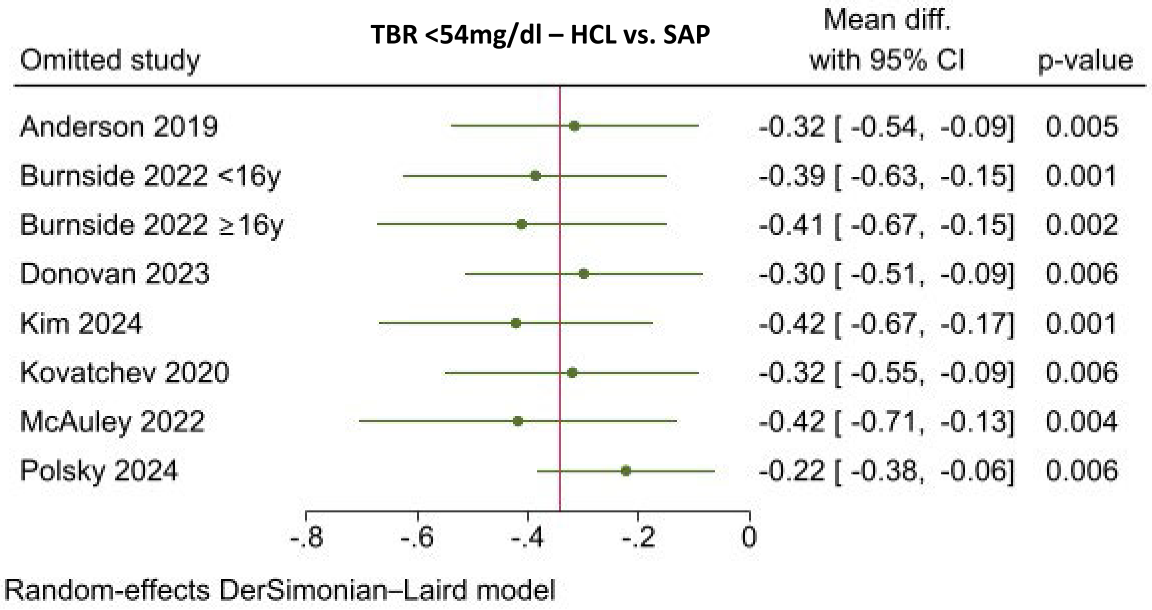
TBR <54mg/dl – HCL vs. SAP
Omitted study
Mean diff. with 95% CI
p-value
Anderson 2019 -0.32 [-0.54, -0.09] 0.005
Burnside 2022 <16y -0.39 [-0.63, -0.15] 0.001
Burnside 2022 ≥16y -0.41 [-0.67, -0.15] 0.002
Donovan 2023 -0.30 [-0.51, -0.09] 0.006
Kim 2024 -0.42 [-0.67, -0.17] 0.001
Kovatchev 2020 -0.32 [-0.55, -0.09] 0.006
McAuley 2022 -0.42 [-0.71, -0.13] 0.004
Polsky 2024 -0.22 [-0.38, -0.06] 0.006
-.8 -.6 -.4 -.2 0
Random-effects DerSimonian–Laird model

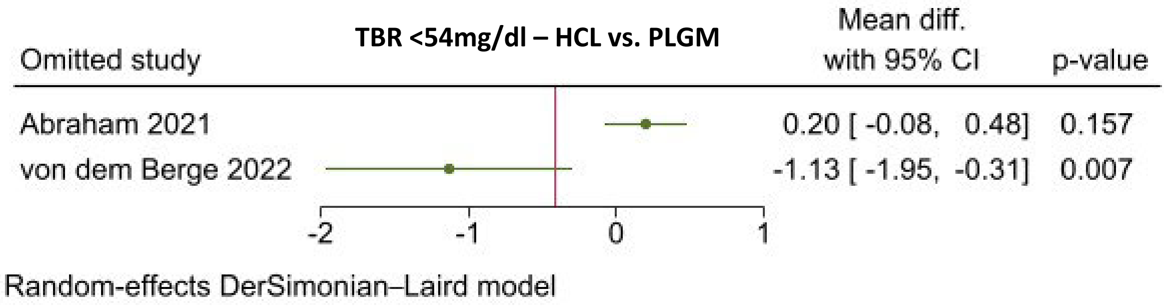
TBR <54mg/dl – HCL vs. PLGM
Omitted study
Mean diff. with 95% CI
p-value
Abraham 2021 0.20 [-0.08, 0.48] 0.157
von dem Berge 2022 -1.13 [-1.95, -0.31] 0.007
-2 -1 0 1
Random-effects DerSimonian–Laird model

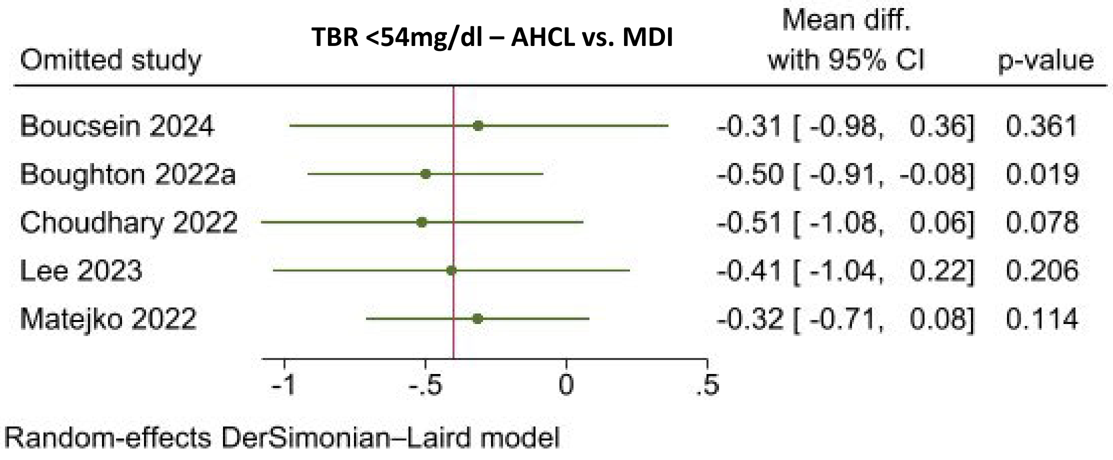
TBR <54mg/dl – AHCL vs. MDI
Omitted study
Mean diff. with 95% CI
p-value
Boucsein 2024 -0.31 [-0.98, 0.36] 0.361
Boughton 2022a -0.50 [-0.91, -0.08] 0.019
Choudhary 2022 -0.51 [-1.08, 0.06] 0.078
Lee 2023 -0.41 [-1.04, 0.22] 0.206
Matejko 2022 -0.32 [-0.71, 0.08] 0.114
-1 -.5 0 .5
Random-effects DerSimonian–Laird model

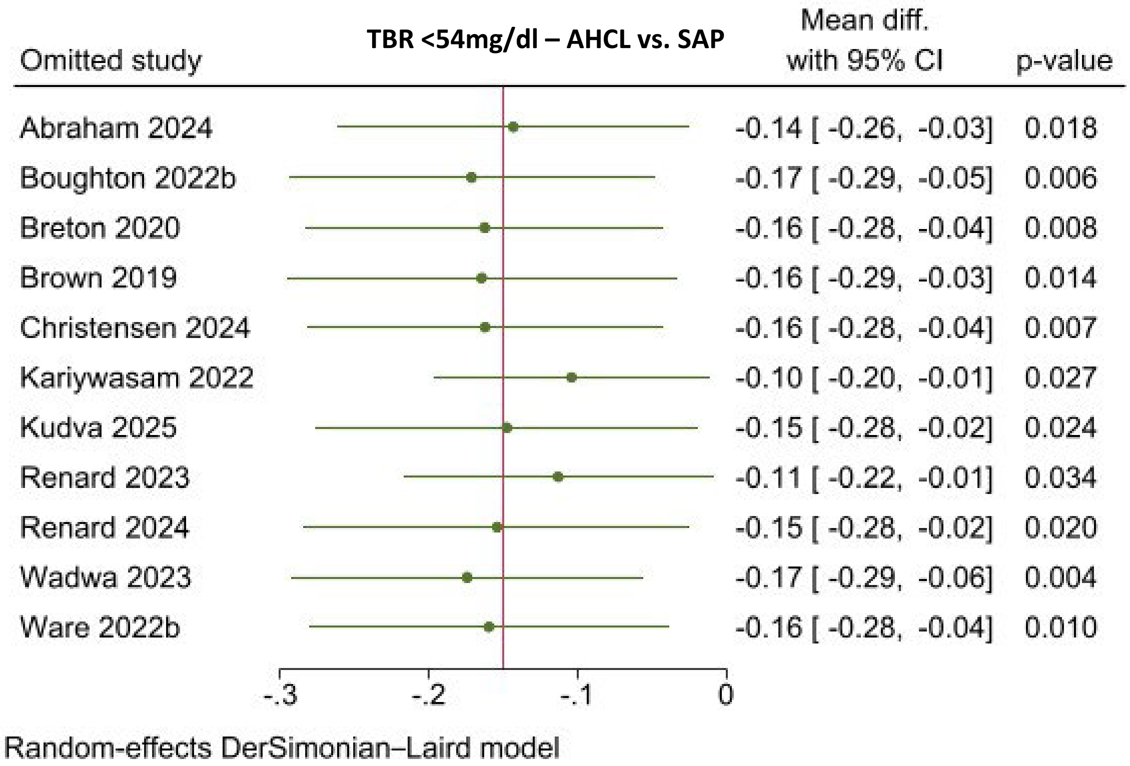
TBR <54mg/dl – AHCL vs. SAP
Omitted study
Mean diff. with 95% CI
p-value
Abraham 2024 -0.14 [-0.26, -0.03] 0.018
Boughton 2022b -0.17 [-0.29, -0.05] 0.006
Breton 2020 -0.16 [-0.28, -0.04] 0.008
Brown 2019 -0.16 [-0.29, -0.03] 0.014
Christensen 2024 -0.16 [-0.28, -0.04] 0.007
Kariywasam 2022 -0.10 [-0.20, -0.01] 0.027
Kudva 2025 -0.15 [-0.28, -0.02] 0.024
Renard 2023 -0.11 [-0.22, -0.01] 0.034
Renard 2024 -0.15 [-0.28, -0.02] 0.020
Wadwa 2023 -0.17 [-0.29, -0.06] 0.004
Ware 2022b -0.16 [-0.28, -0.04] 0.010
-.3
-.2
-.1
0
Random-effects DerSimonian–Laird model

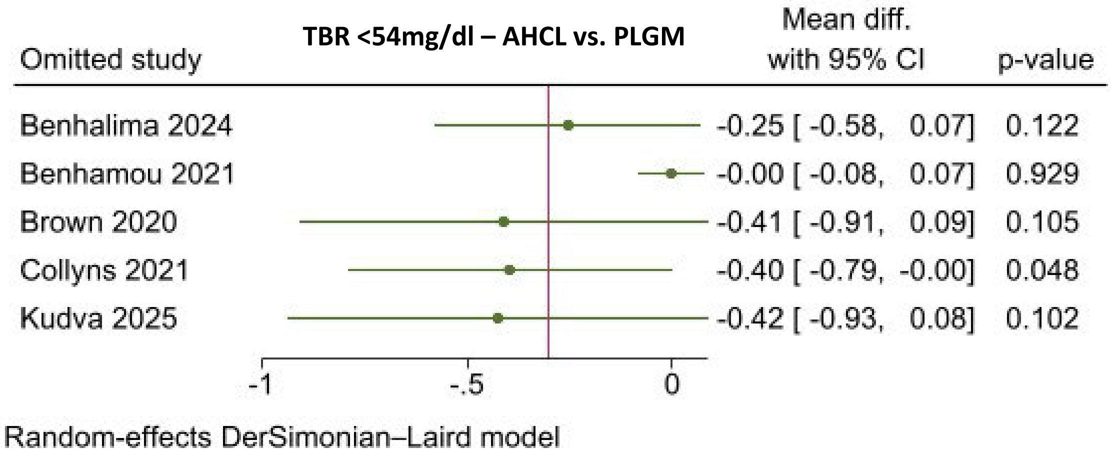
TBR <54mg/dl – AHCL vs. PLGM
Omitted study
Mean diff. with 95% CI
p-value
Benhalima 2024 -0.25 [-0.58, 0.07] 0.122
Benhamou 2021 -0.00 [-0.08, 0.07] 0.929
Brown 2020 -0.41 [-0.91, 0.09] 0.105
Collyns 2021 -0.40 [-0.79, -0.00] 0.048
Kudva 2025 -0.42 [-0.93, 0.08] 0.102
-1
-.5
0
Random-effects DerSimonian–Laird model

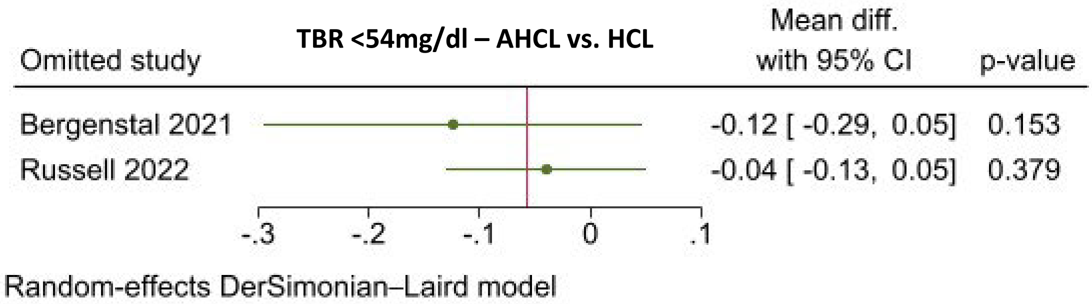
TBR <54mg/dl – AHCL vs. HCL
Omitted study
Mean diff. with 95% CI
p-value
Bergenstal 2021 -0.12 [-0.29, 0.05] 0.153
Russell 2022 -0.04 [-0.13, 0.05] 0.379
-.3
-.2
-.1
0
.1
Random-effects DerSimonian–Laird model

6.6 Leave-one-out HbA1c

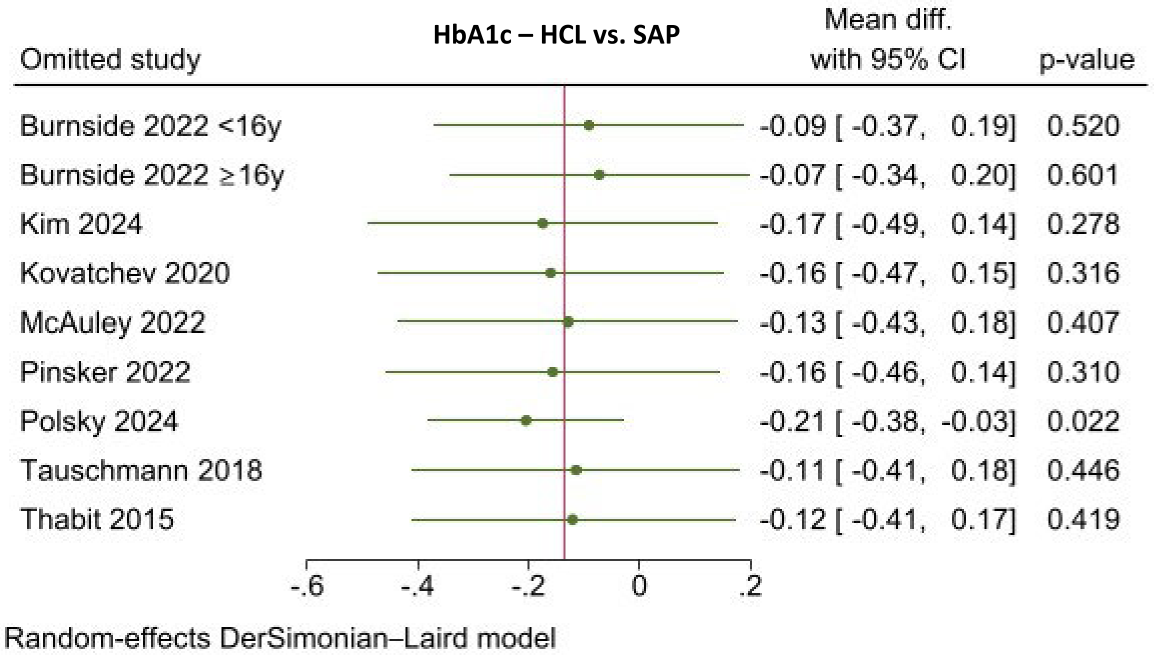

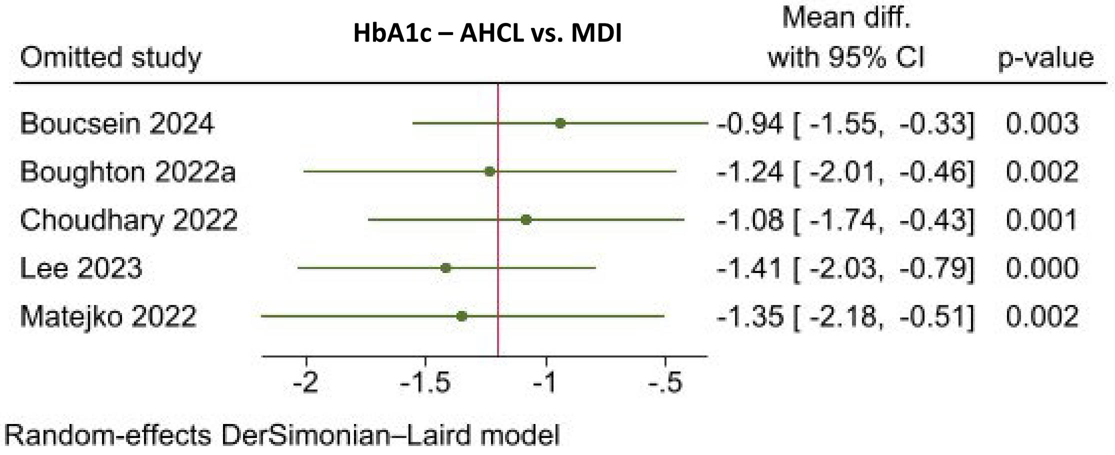

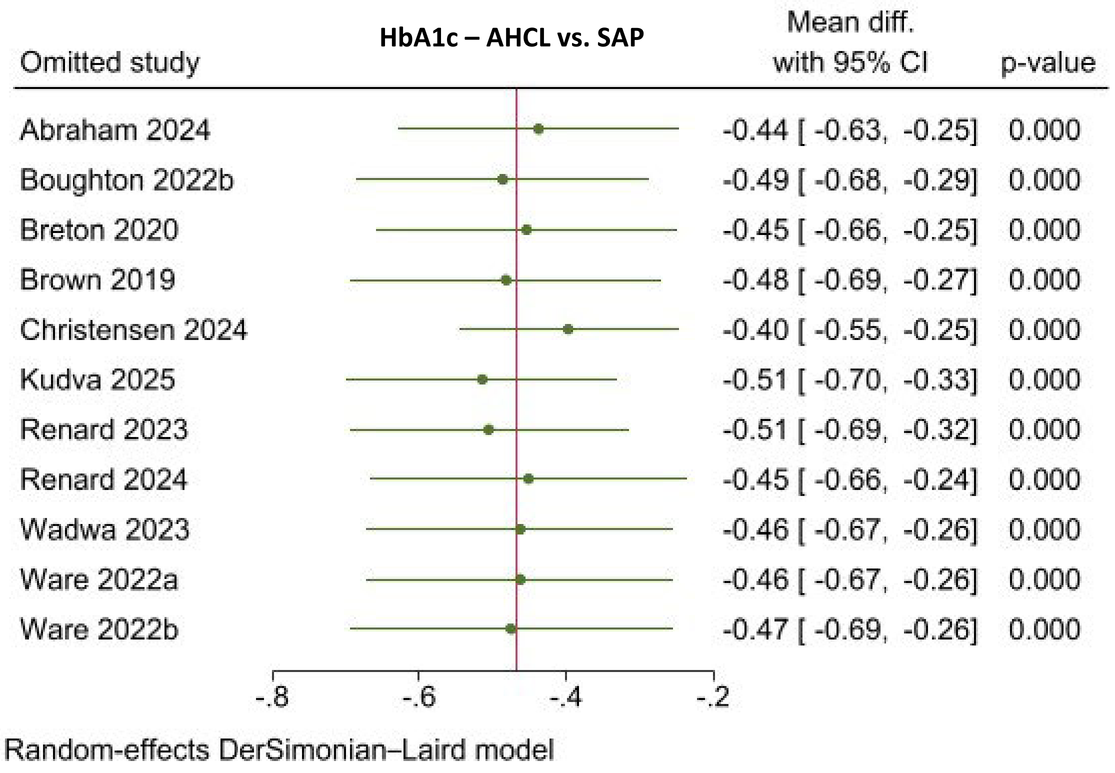
HbA1c – AHCL vs. SAP
Omitted study
Mean diff. with 95% CI
p-value
Abraham 2024 -0.44 [-0.63, -0.25] 0.000
Boughton 2022b -0.49 [-0.68, -0.29] 0.000
Breton 2020 -0.45 [-0.66, -0.25] 0.000
Brown 2019 -0.48 [-0.69, -0.27] 0.000
Christensen 2024 -0.40 [-0.55, -0.25] 0.000
Kudva 2025 -0.51 [-0.70, -0.33] 0.000
Renard 2023 -0.51 [-0.69, -0.32] 0.000
Renard 2024 -0.45 [-0.66, -0.24] 0.000
Wadwa 2023 -0.46 [-0.67, -0.26] 0.000
Ware 2022a -0.46 [-0.67, -0.26] 0.000
Ware 2022b -0.47 [-0.69, -0.26] 0.000
-.8
-.6
-.4
-.2
Random-effects DerSimonian–Laird model

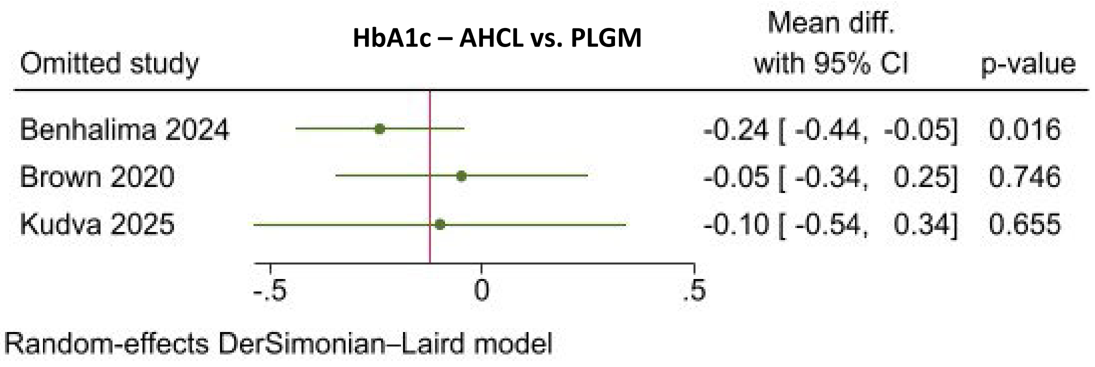
HbA1c – AHCL vs. PLGM
Omitted study
Mean diff. with 95% CI
p-value
Benhalima 2024 -0.24 [-0.44, -0.05] 0.016
Brown 2020 -0.05 [-0.34, 0.25] 0.746
Kudva 2025 -0.10 [-0.54, 0.34] 0.655
-.5
0
.5
Random-effects DerSimonian–Laird model

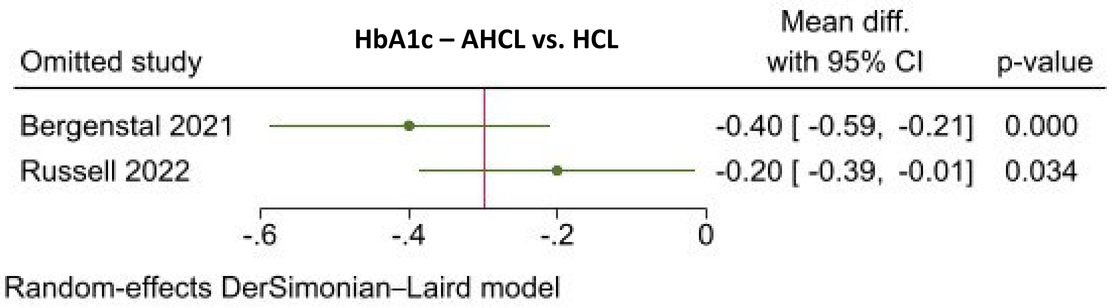
HbA1c – AHCL vs. HCL
Omitted study
Mean diff. with 95% CI
p-value
Bergenstal 2021 -0.40 [-0.59, -0.21] 0.000
Russell 2022 -0.20 [-0.39, -0.01] 0.034
-.6
-.4
-.2
0
Random-effects DerSimonian–Laird model

7 Supplement to small-study bias assessment

7.1 Small-study bias TIR

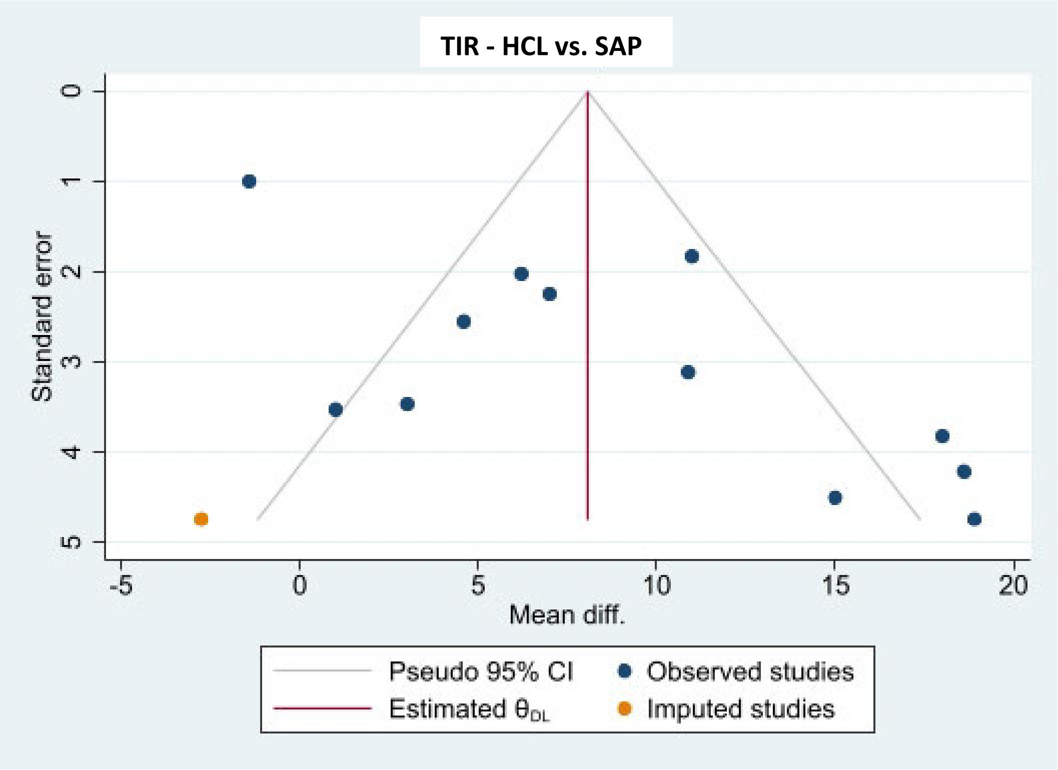

Egger’s test p value: 0.0031

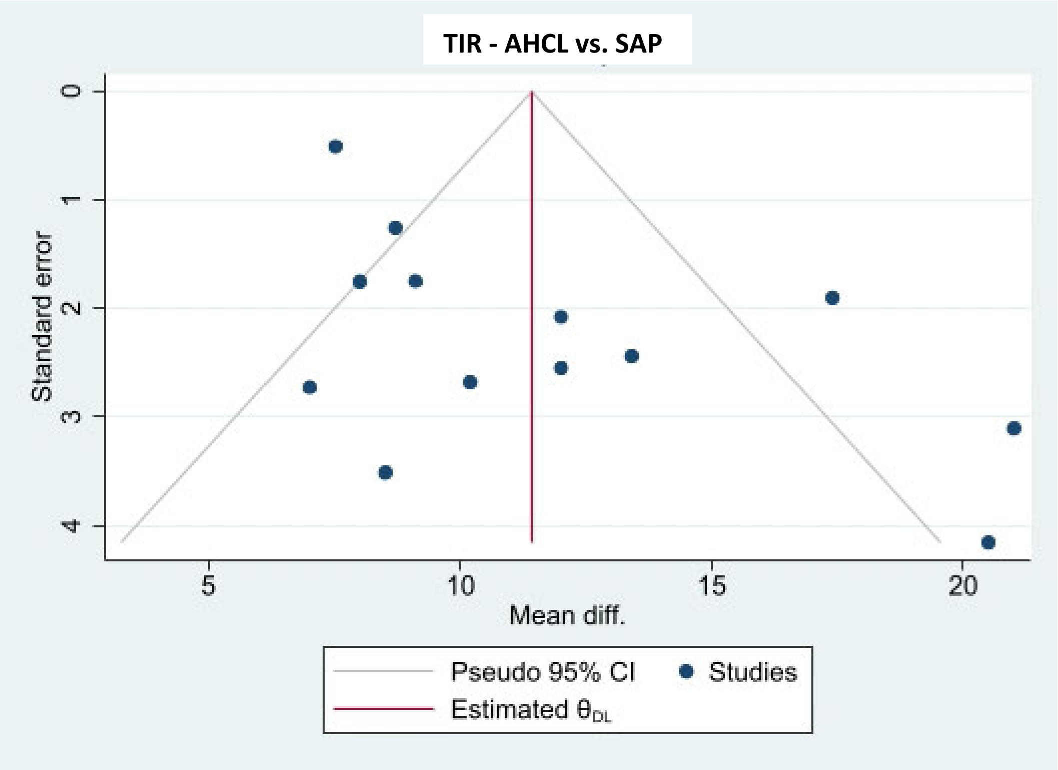

Egger’s test p value: 0.0301

7.2 Small-study bias TAR >180 mg/dl

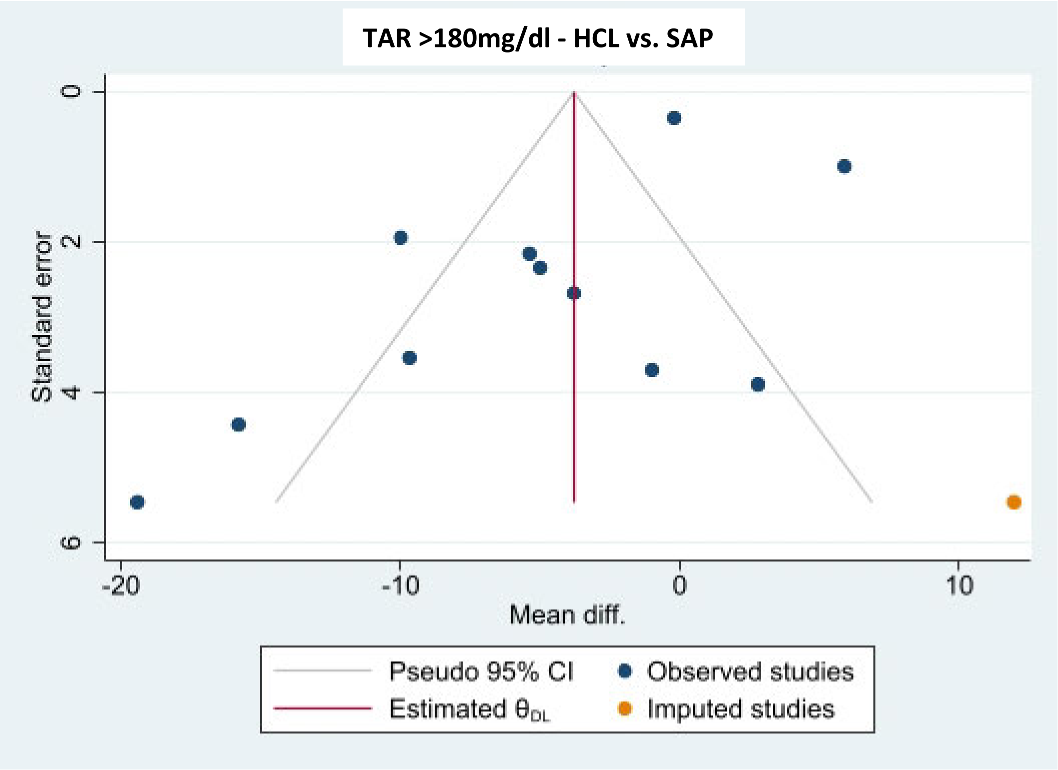

Egger's test p value: 0.0182

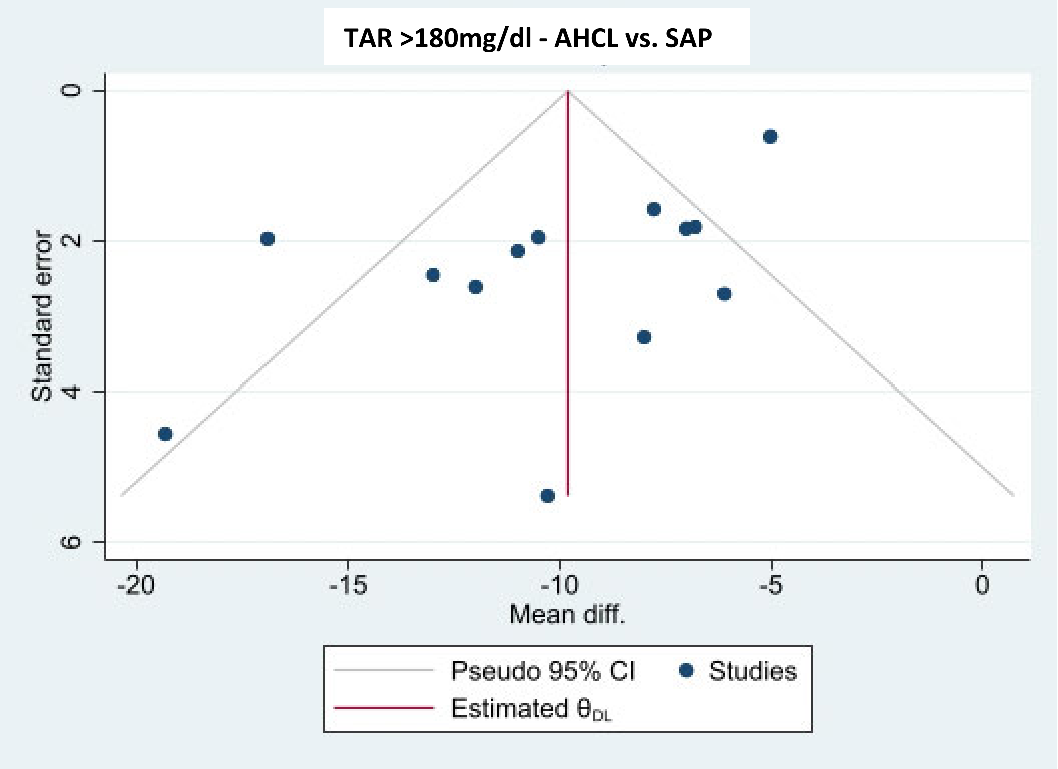

Egger's test p value: 0.0602

7.3 Small-study bias TAR >250 mg/dl

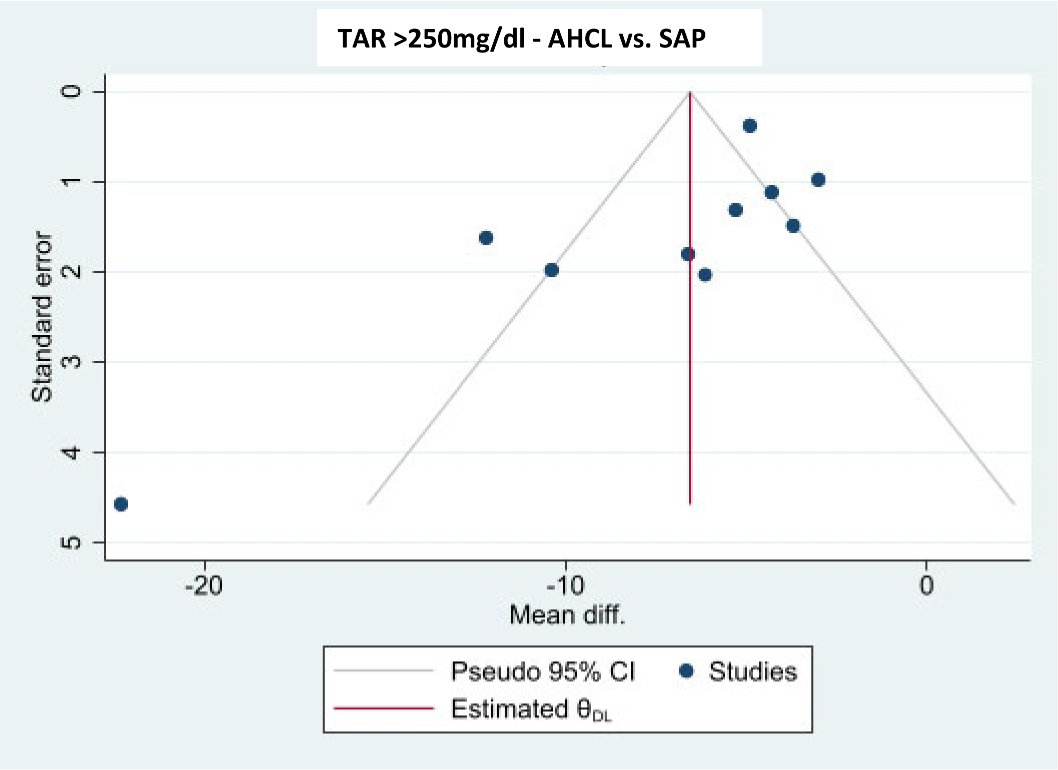

Egger's test p value: 0.0006

7.4 Small-study bias TBR <70 mg/dl

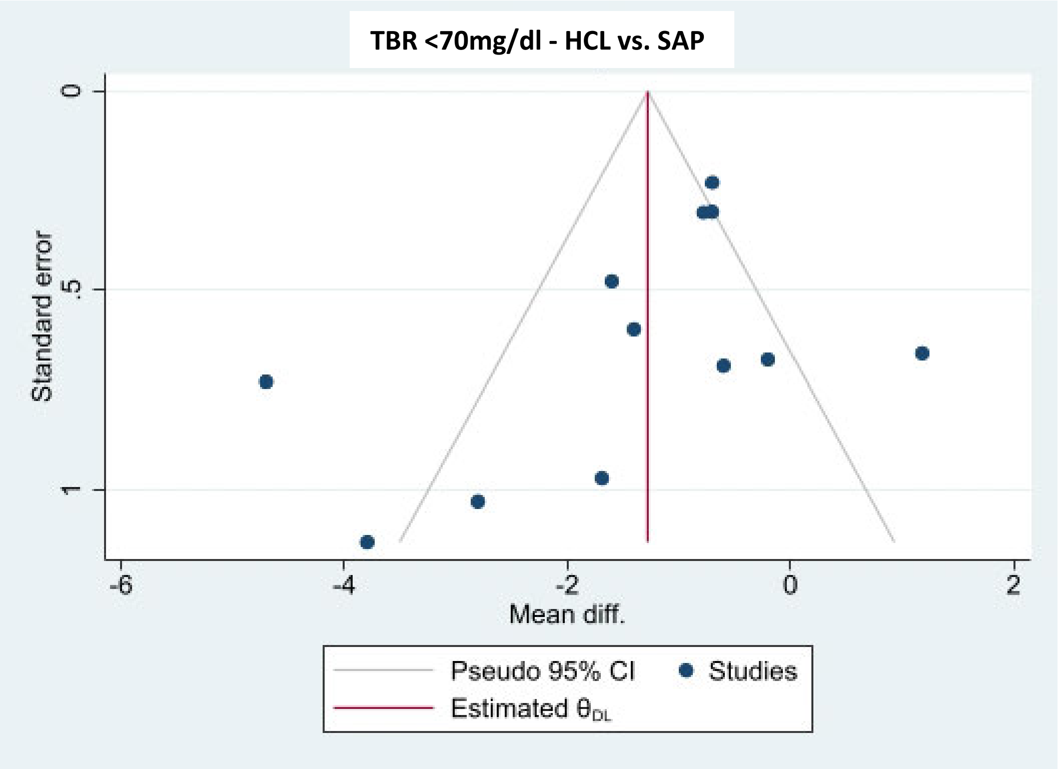

Egger's test p value: 0.0796

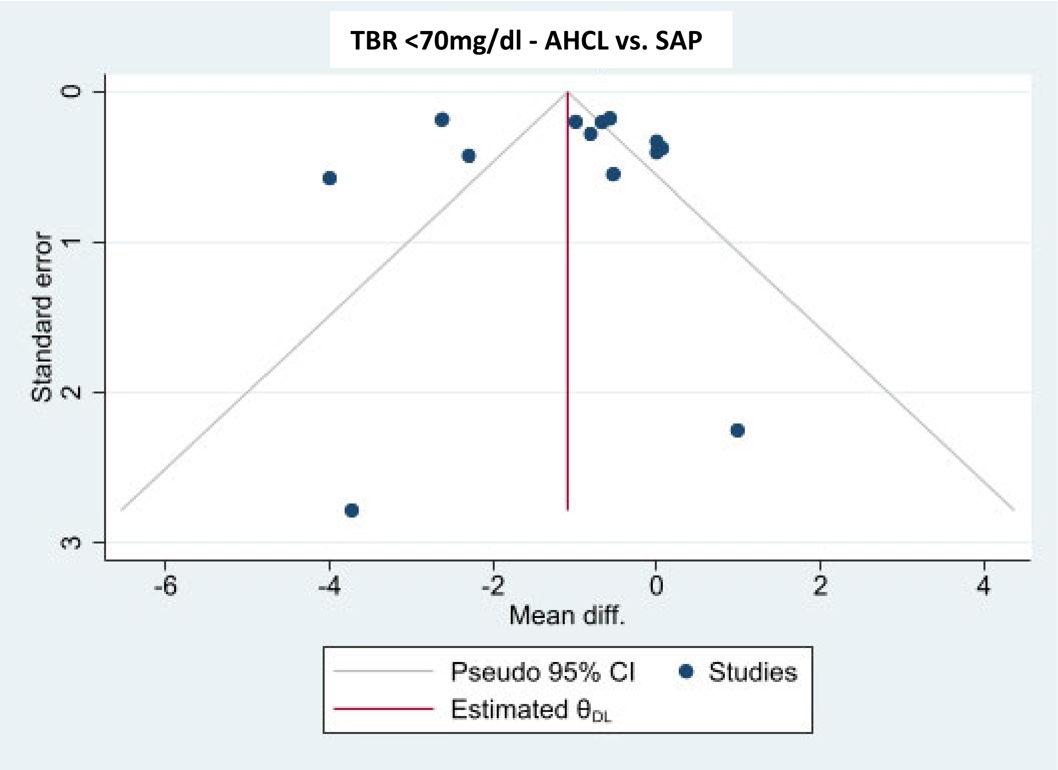

Egger's test p value: 0.7505

7.5 Small-study bias TBR <54 mg/dl

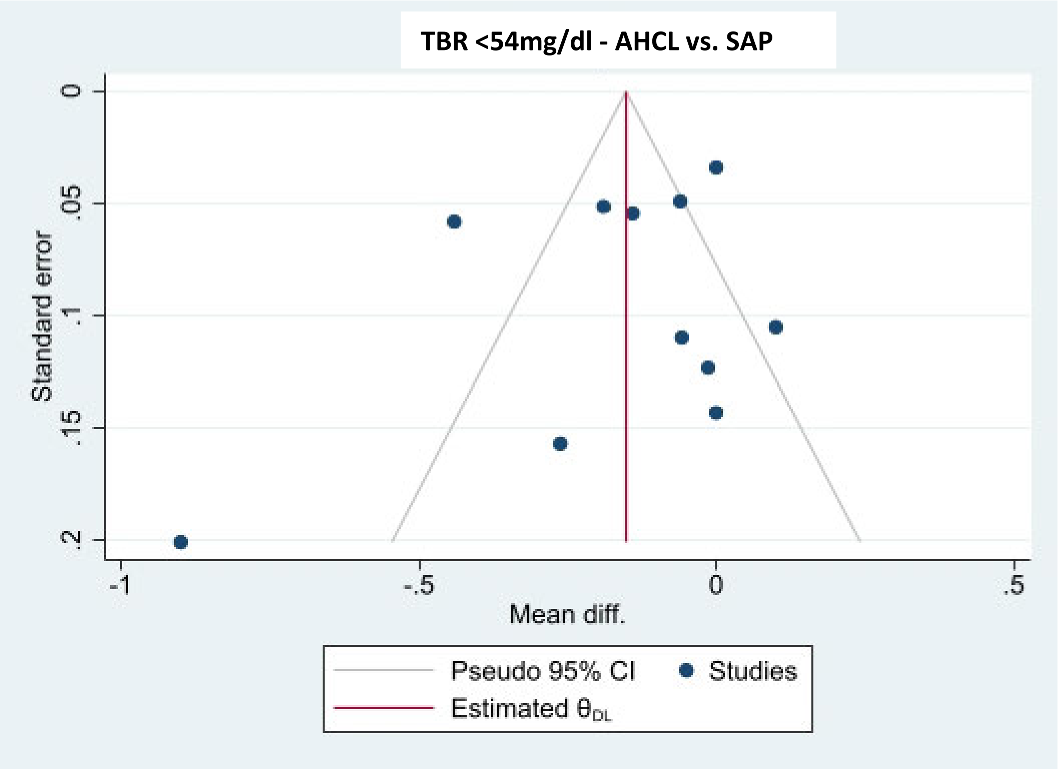

Egger's test p value: 0.2168

7.6 Small-study bias HbA1c

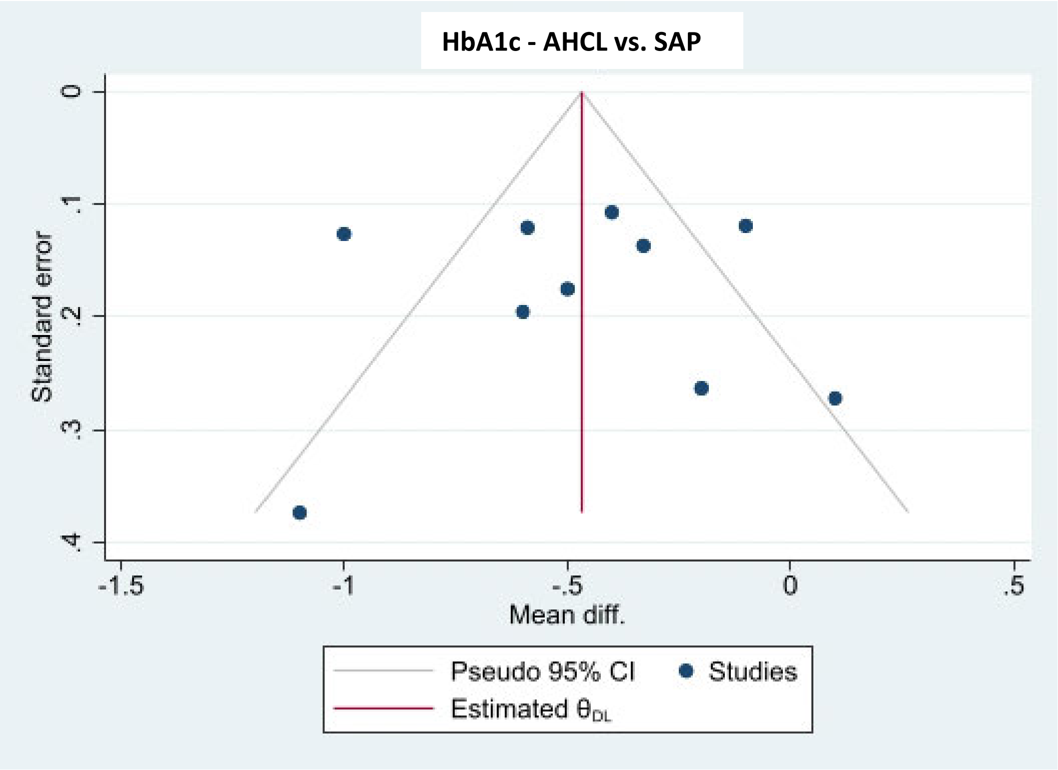

Egger’s test p value: 0.9331

8 Supplement to GRADE CoE assessment from pairwise meta-analysis

Certainty assessment							№ of patients		Certainty
№ of studies	Study design	Risk of bias	Inconsistency	Indirectness	Imprecision	Other considerations	Intervention	Control	
**TIR 70-180 mg/dl (HCL versus CSII)**									
4	randomised trials	serious[a]	not serious	not serious	serious[b]	none	205	202	⊕⊕◯◯ Low[a,b]
**TIR 70-180 mg/dl (HCL versus SAP)**									
12	randomised trials	not serious	very serious[c]	not serious	very serious[d]	publication bias strongly suspected[e]	355	357	⊕◯◯◯ Very low[c,d,e]
**TIR 70-180 mg/dl (HCL versus PLGM)**									
2	randomised trials	serious[a]	not serious	not serious	very serious[d]	none	95	100	⊕◯◯◯ Very low[a,d]
**TIR 70-180 mg/dl (AHCL versus MDI)**									
4	randomised trials	not serious	serious[f]	not serious	serious[b]	none	135	121	⊕⊕◯◯ Low[b,f]
**TIR 70-180 mg/dl (AHCL versus SAP)**									
13	randomised trials	not serious	serious[f]	not serious	not serious	publication bias strongly suspected[e]	784	547	⊕⊕◯◯ Low[e,f]
**TIR 70-180 mg/dl (AHCL versus PLGM)**									
5	randomised trials	not serious	serious[f]	not serious	very serious[d]	none	247	243	⊕◯◯◯ Very low[d,f]
**TIR 70-180 mg/dl (AHCL versus HCL)**									
2	randomised trials	not serious	serious[f]	not serious	very serious[d]	none	331	219	⊕◯◯◯ Very low[d,f]

Certainty assessment							№ of patients		Certainty
**№ of studies**	**Study design**	**Risk of bias**	**Inconsistency**	**Indirectness**	**Imprecision**	**Other considerations**	**Intervention**	**Control**	
**TAR >180 mg/dl (HCL versus CSII)**									
3	randomised trials	serious[a]	not serious	not serious	very serious[d]	none	65	64	⊕◯◯◯ Very low[a,d]
**TAR >180 mg/dl (HCL versus SAP)**									
11	randomised trials	serious[a]	very serious[c]	not serious	very serious[d]	publication bias strongly suspected[e]	329	320	⊕◯◯◯ Very low[a,c,d,e]
**TAR >180 mg/dl (HCL versus PLGM)**									
2	randomised trials	serious[a]	not serious	not serious	very serious[g]	none	95	100	⊕◯◯◯ Very low[a,g]
**TAR >180 mg/dl (AHCL versus MDI)**									
5	randomised trials	not serious	serious[f]	not serious	serious[b]	none	194	182	⊕⊕◯◯ Low[b,f]
**TAR >180 mg/dl (AHCL versus SAP)**									
13	randomised trials	not serious	serious[f]	not serious	not serious	publication bias strongly suspected[e]	784	548	⊕⊕◯◯ Low[e,f]
**TAR >180 mg/dl (AHCL versus PLGM)**									
5	randomised trials	not serious	serious[f]	not serious	very serious[g]	none	247	243	⊕◯◯◯ Very low[f,g]
**TAR >180 mg/dl (AHCL versus HCL)**									
2	randomised trials	not serious	serious[f]	not serious	very serious[g]	none	331	219	⊕◯◯◯ Very low[f,g]

Certainty assessment							№ of patients		Certainty
№ of studies	Study design	Risk of bias	Inconsistency	Indirectness	Imprecision	Other considerations	Intervention	Control	
**TAR >250 mg/dl (HCL versus CSII)**									
2	randomised trials	serious[a]	not serious	not serious	very serious[g]	none	45	44	⨁◯◯◯ Very low[a,g]
**TAR >250 mg/dl (HCL versus SAP)**									
8	randomised trials	serious[a]	very serious[c]	not serious	very serious[d]	none	256	263	⨁◯◯◯ Very low[a,c,d]
**TAR >250 mg/dl (AHCL versus MDI)**									
3	randomised trials	not serious	serious[f]	not serious	serious[b]	none	93	91	⨁⨁◯◯ Low[b,f]
**TAR >250 mg/dl (AHCL versus SAP)**									
10	randomised trials	not serious	serious[f]	not serious	not serious*	publication bias strongly suspected[e]	639	394	⨁⨁◯◯ Low[e,f]
**TAR >250 mg/dl (AHCL versus PLGM)**									
3	randomised trials	not serious	serious[f]	not serious	very serious[g]	none	140	139	⨁◯◯◯ Very low[f,g]
**TAR >250 mg/dl (AHCL versus HCL)**									
2	randomised trials	not serious	serious[f]	not serious	very serious[g]	none	331	219	⨁◯◯◯ Very low[f,g]

Certainty assessment							№ of patients		Certainty
№ of studies	Study design	Risk of bias	Inconsistency	Indirectness	Imprecision	Other considerations	Intervention	Control	
**TBR <70 mg/dl (HCL versus CSII)**									
4	randomised trials	serious[a]	not serious	not serious	serious[b]	none	205	202	⊕⊕◯◯ Low[a,b]
**TBR <70 mg/dl (HCL versus SAP)**									
12	randomised trials	not serious	very serious[c]	not serious	very serious[d]	publication bias strongly suspected[e]	355	357	⊕◯◯◯ Very low[c,d,e]
**TBR <70 mg/dl (HCL versus PLGM)**									
2	randomised trials	serious[a]	very serious[c]	not serious	very serious[g]	none	95	100	⊕◯◯◯ Very low[a,c,g]
**TBR <70 mg/dl (AHCL versus MDI)**									
4	randomised trials	not serious	very serious[c]	not serious	very serious[g]	none	135	121	⊕◯◯◯ Very low[c,g]
**TBR <70 mg/dl (AHCL versus SAP)**									
13	randomised trials	not serious	serious[h]	not serious	serious[i]	none	784	547	⊕⊕◯◯ Low[h,i]
**TBR <70 mg/dl (AHCL versus PLGM)**									
5	randomised trials	not serious	serious[f]	not serious	very serious[g]	none	247	243	⊕◯◯◯ Very low[f,g]
**TBR <70 mg/dl (AHCL versus HCL)**									
2	randomised trials	not serious	not serious	not serious	very serious[g]	none	331	219	⊕⊕◯◯ Low[g]

Certainty assessment							№ of patients		Certainty
№ of studies	Study design	Risk of bias	Inconsistency	Indirectness	Imprecision	Other considerations	Intervention	Control	
**TBR <54 mg/dl (HCL versus CSII)**									
2	randomised trials	serious[a]	not serious	not serious	very serious[d]	none	36	36	⊕◯◯◯ Very low[a,d]
**TBR <54 mg/dl (HCL versus SAP)**									
8	randomised trials	not serious	serious[f]	not serious	very serious[d]	none	230	237	⊕◯◯◯ Very low[d,f]
**TBR <54 mg/dl (HCL versus PLGM)**									
2	randomised trials	serious[a]	very serious[c]	not serious	very serious[g]	none	95	100	⊕◯◯◯ Very low[a,c,g]
**TBR <54 mg/dl (AHCL versus MDI)**									
5	randomised trials	not serious	very serious[c]	not serious	very serious[g]	none	194	182	⊕◯◯◯ Very low[c,g]
**TBR <54 mg/dl (AHCL versus SAP)**									
11	randomised trials	not serious	very serious[c]	not serious	serious[j]	none	669	422	⊕◯◯◯ Very low[c,j]
**TBR <54 mg/dl (AHCL versus PLGM)**									
5	randomised trials	not serious	serious[f]	not serious	very serious[g]	none	247	243	⊕◯◯◯ Very low[f,g]
**TBR <54 mg/dl (AHCL versus HCL)**									
2	randomised trials	not serious	not serious	not serious	very serious[g]	none	331	219	⊕⊕◯◯ Low[g]

Certainty assessment							№ of patients		Certainty
№ of studies	Study design	Risk of bias	Inconsistency	Indirectness	Imprecision	Other considerations	Intervention	Control	
**HbA1c [%] (HCL versus SAP)**									
9	randomised trials	not serious	very serious[c]	not serious	very serious[g]	none	312	313	⊕◯◯◯ Very low[c,g]
**HbA1c [%] (AHCL versus MDI)**									
5	randomised trials	not serious	serious[f]	not serious	serious[b]	none	199	195	⊕⊕◯◯ Low[b,f]
**HbA1c [%] (AHCL versus SAP)**									
11	randomised trials	not serious	serious[h]	not serious	serious[i]	none	709	467	⊕⊕◯◯ Low[h,i]
**HbA1c [%] (AHCL versus PLGM)**									
3	randomised trials	not serious	serious[h]	not serious	very serious[g]	none	183	179	⊕◯◯◯ Very low[g,h]
**HbA1c [%] (AHCL versus HCL)**									
2	randomised trials	not serious	not serious	not serious	very serious[d]	none	331	219	⊕⊕◯◯ Low[d]

Explanations:

a. Downgraded by one level for risk of bias: RoB unclear (≥50% some concerns)
b. Downgraded by one level for imprecision: Null value not in CI, threshold value not in CI and <400 participants per group
c. Downgraded by two levels for inconsistency: Estimates pointed to different directions and 95% CI did not overlap
d. Downgraded by two levels for imprecision: Null value not in CI, threshold value in CI and <400 participants per group
e. Downgraded by one level for publication bias: ≥10 studies and publication bias was detected
f. Downgraded by one level for inconsistency: Estimates pointed to same directions but were appreciably different and 95% CIs did not overlap
g. Downgraded by two levels for imprecision: Null value in 95%-CI and <400 participants per group
h. Downgraded by one level for inconsistency: Estimates pointed to different directions but 95% CIs overlapped

i. Downgraded by one level for imprecision: Null value not in CI, threshold value in CI and ≥400 participants per group
j. Downgraded by one level for imprecision: Null value in CI, threshold value not in CI and ≥400 participants per group

* Although the control group comprised only 396 participants, we did not downgrade for imprecision because the total sample size was much larger than 800 due to the 639 participants in the intervention group.

9 Supplement to network meta-analysis

9.1 Network meta-analysis TIR

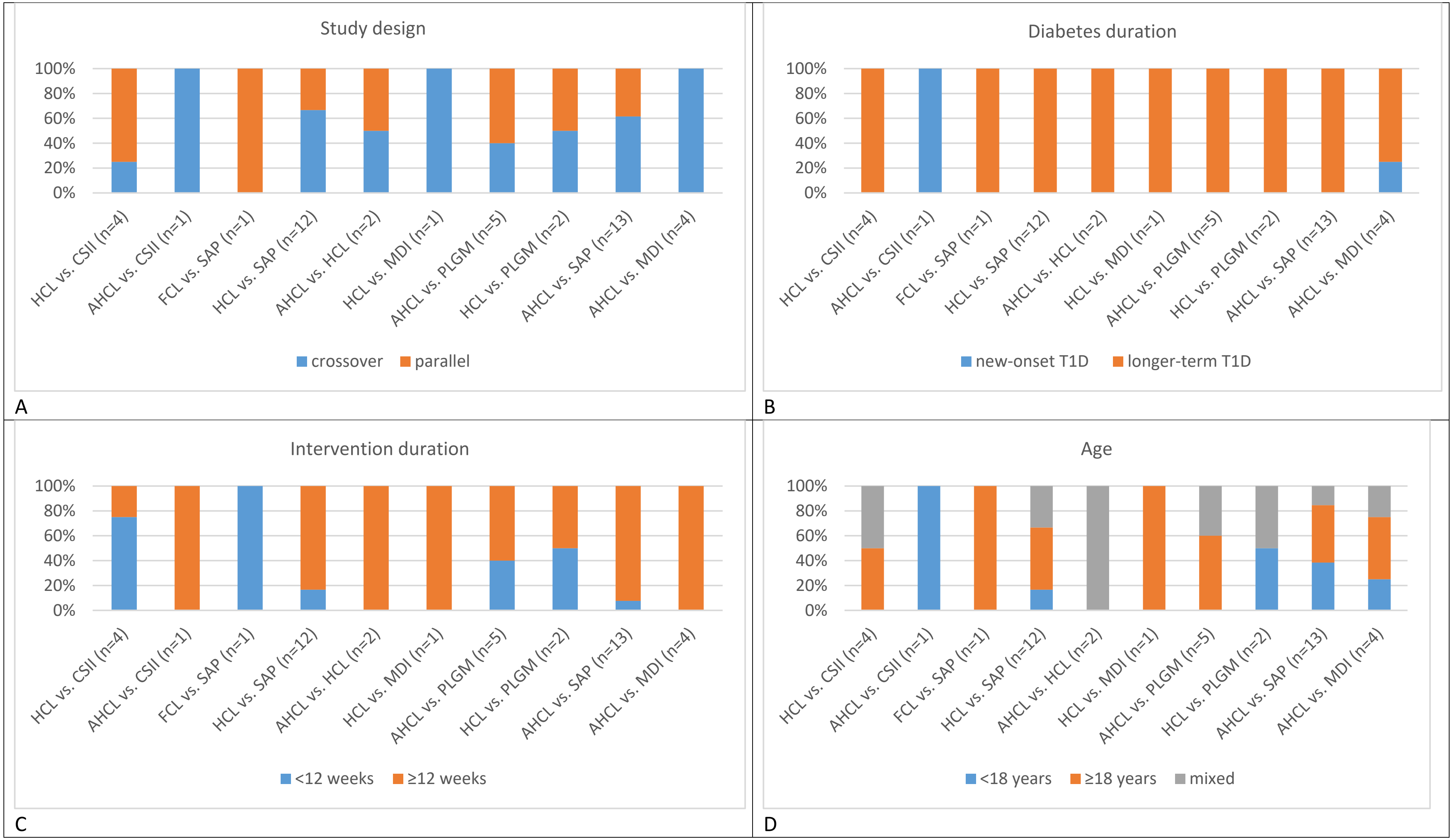

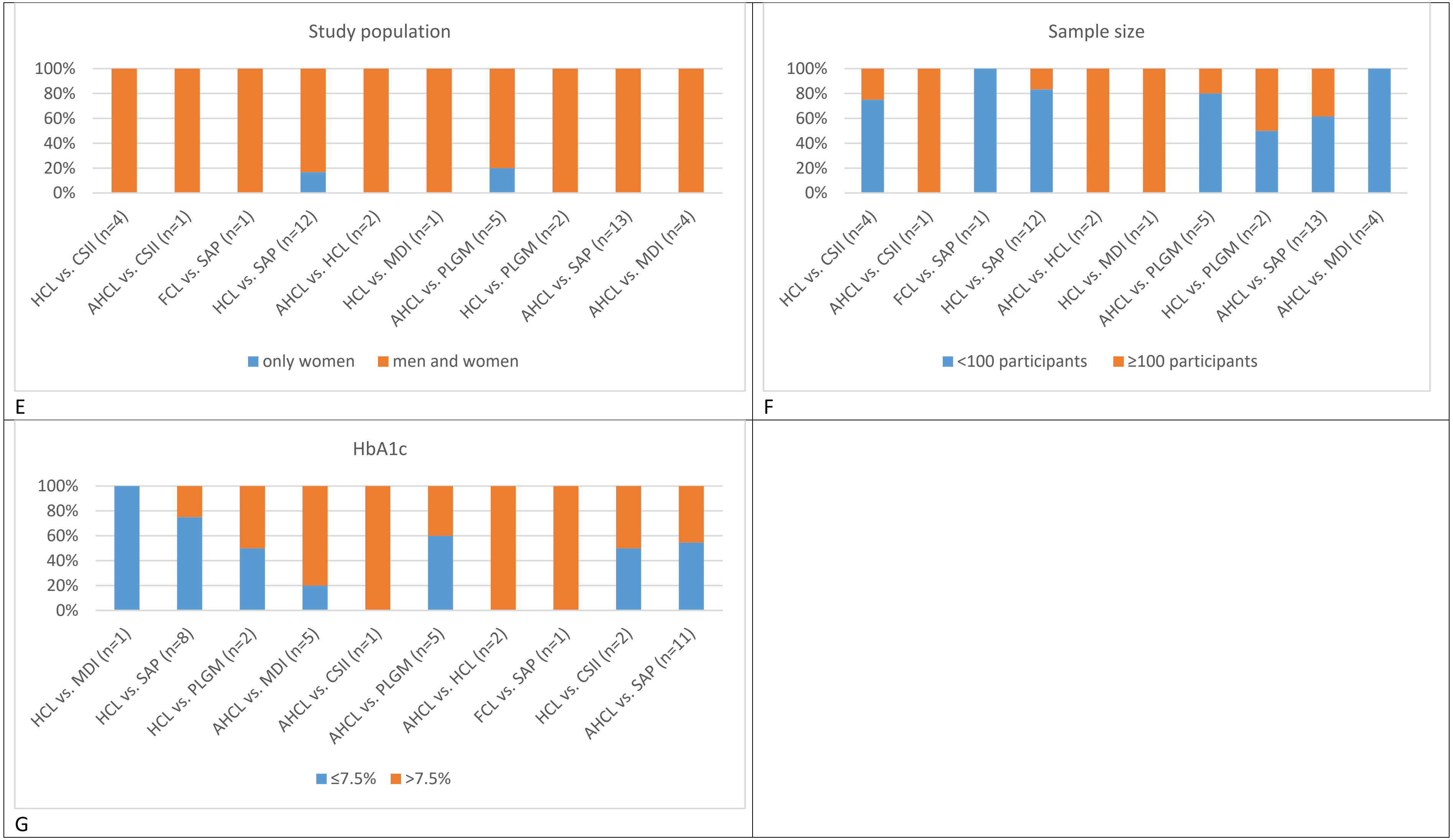

Figure A: Results of the transitivity assessment for time in range 70-180 mg/dl for (A) study design, (B) diabetes duration, (C) intervention duration, (D) age, (E) study population, (F) sample size, and (G) baseline HbA1c.

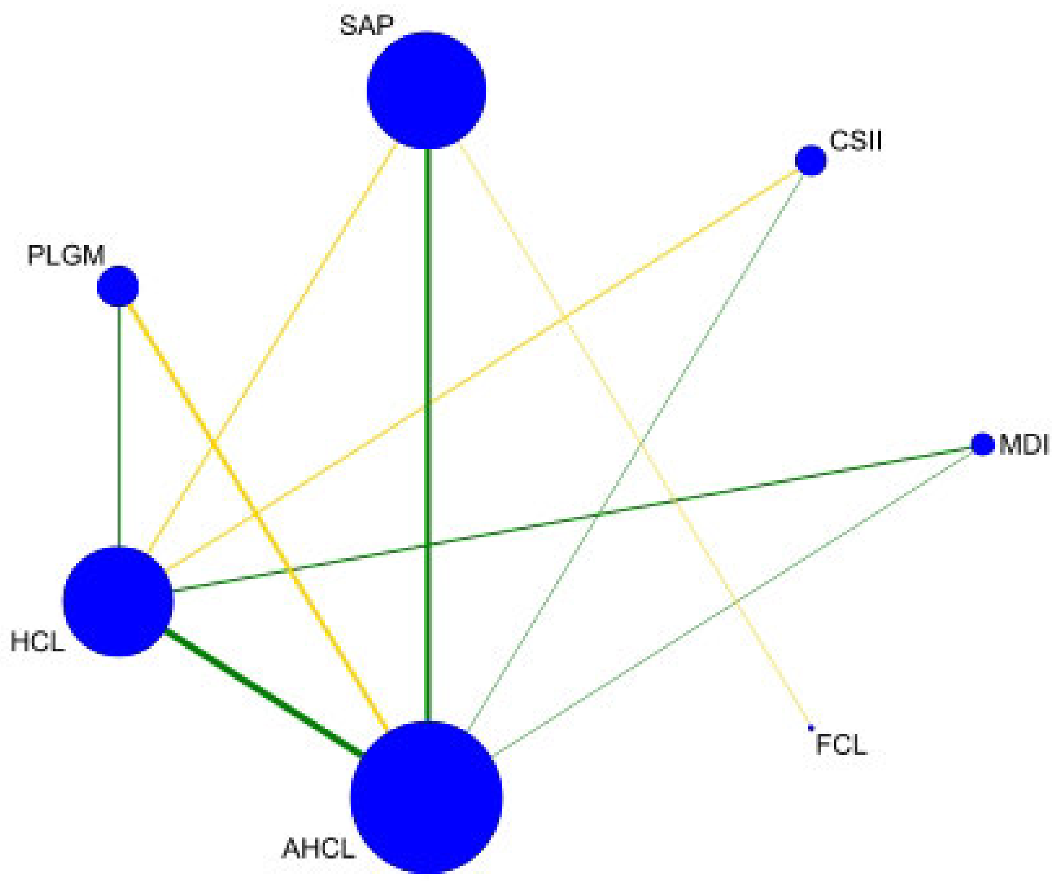

Number of studies	45
Number of treatments	7
Number of comparisons with direct evidence	10
Number of comparisons with indirect evidence	11
Density	0.5
Percentage of common comparators	85.7
Percentages of strong edges	70.0
Median thickness (IQR25; IQR75)	3 (1; 5)

Figure B: Plot and metrics of the TIR network

The node size in the network graph represents the number of participants with the respective intervention. The line width of the edge represents the mean of inverse variances (precision) of the treatment effect of the studies on which the direct comparison is based. The colour of the edge corresponds to the average level of the RoB estimated as the precision-weighted mean of the trials of the direct comparison.

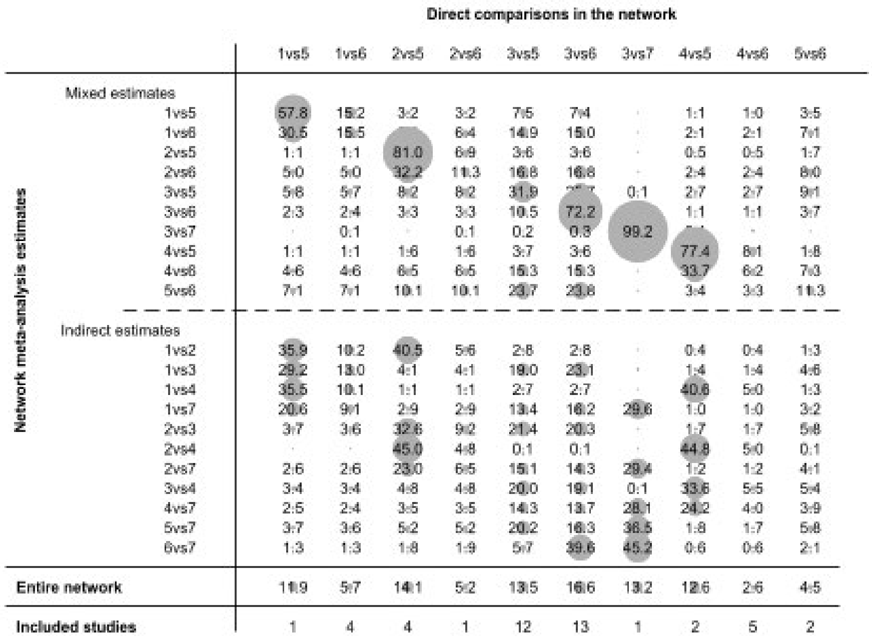

Coding	Treatment
1	MDI
2	CSII
3	SAP
4	PLGM
5	HCL
6	AHCL
7	FCL

Figure C: Contribution matrix for the TIR network

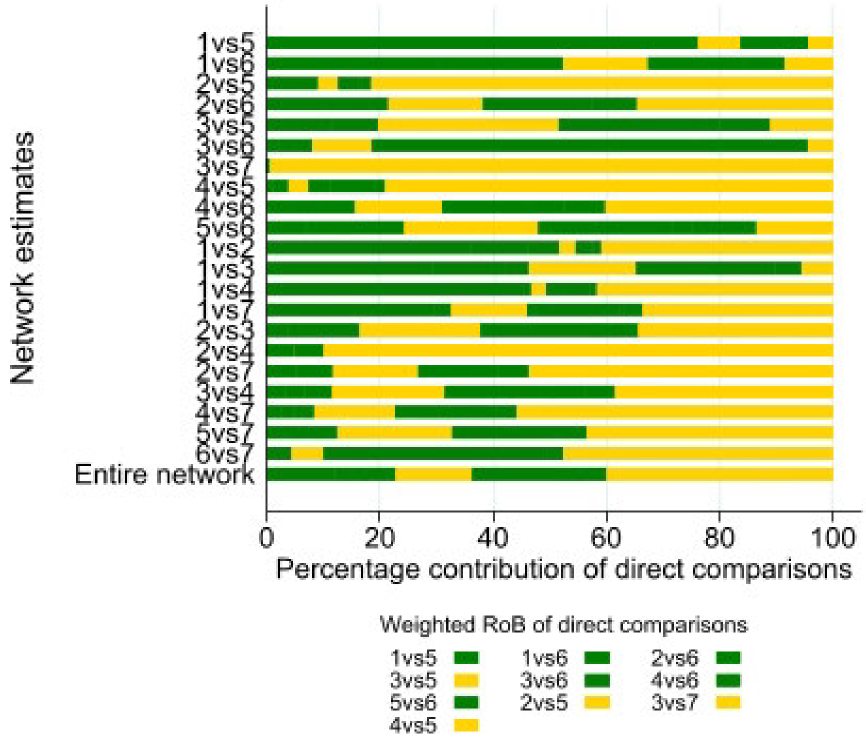

Coding	Treatment
1	MDI
2	CSII
3	SAP
4	PLGM
5	HCL
6	AHCL
7	FCL

Figure D: Study limitations for each network estimate for pairwise comparisons in the TIR network

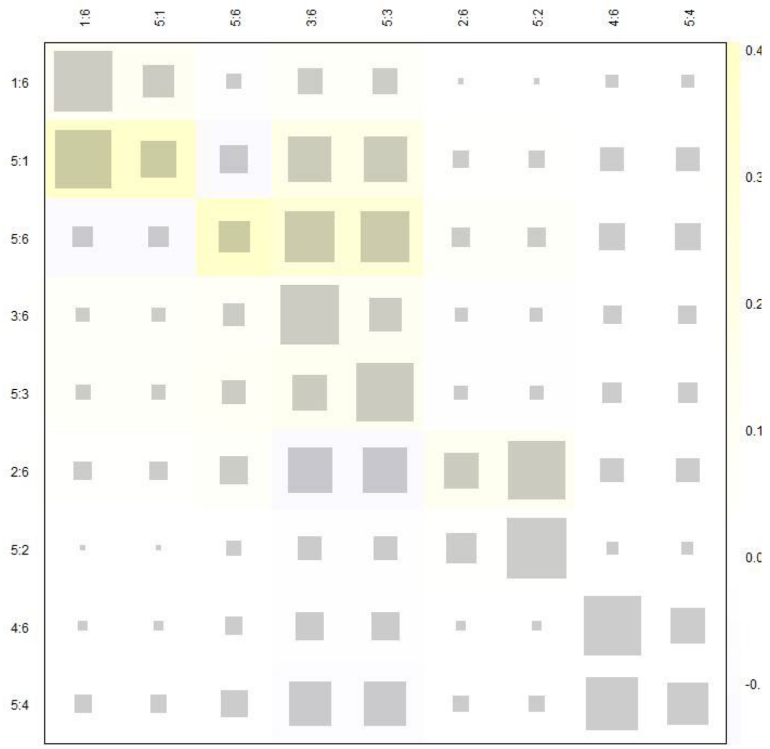

Coding	Treatment
1	MDI
2	CSII
3	SAP
4	PLGM
5	HCL
6	AHCL
7	FCL

Figure E: Net heat plot

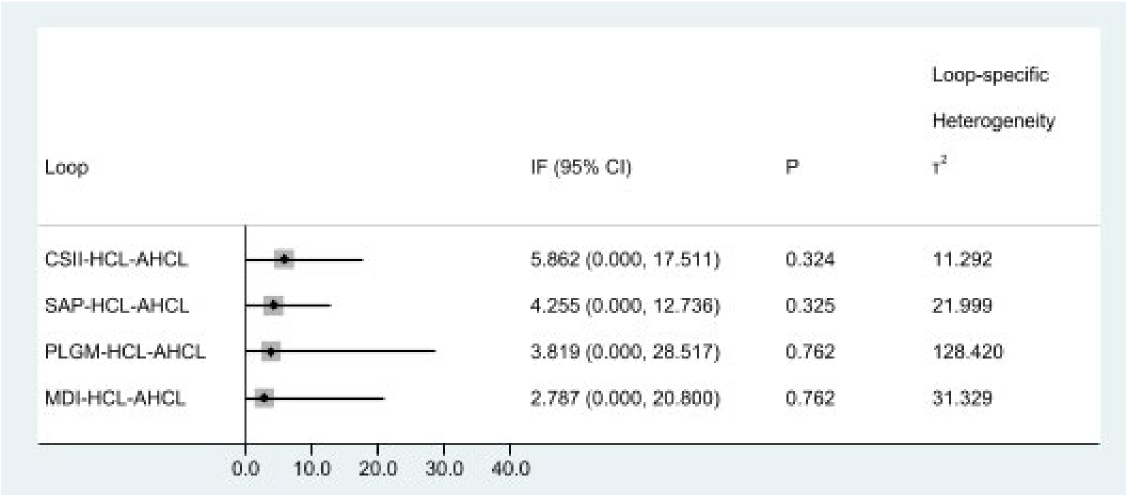

Figure F: Inconsistency plot for the TIR network assuming loop-specific heterogeneity estimates

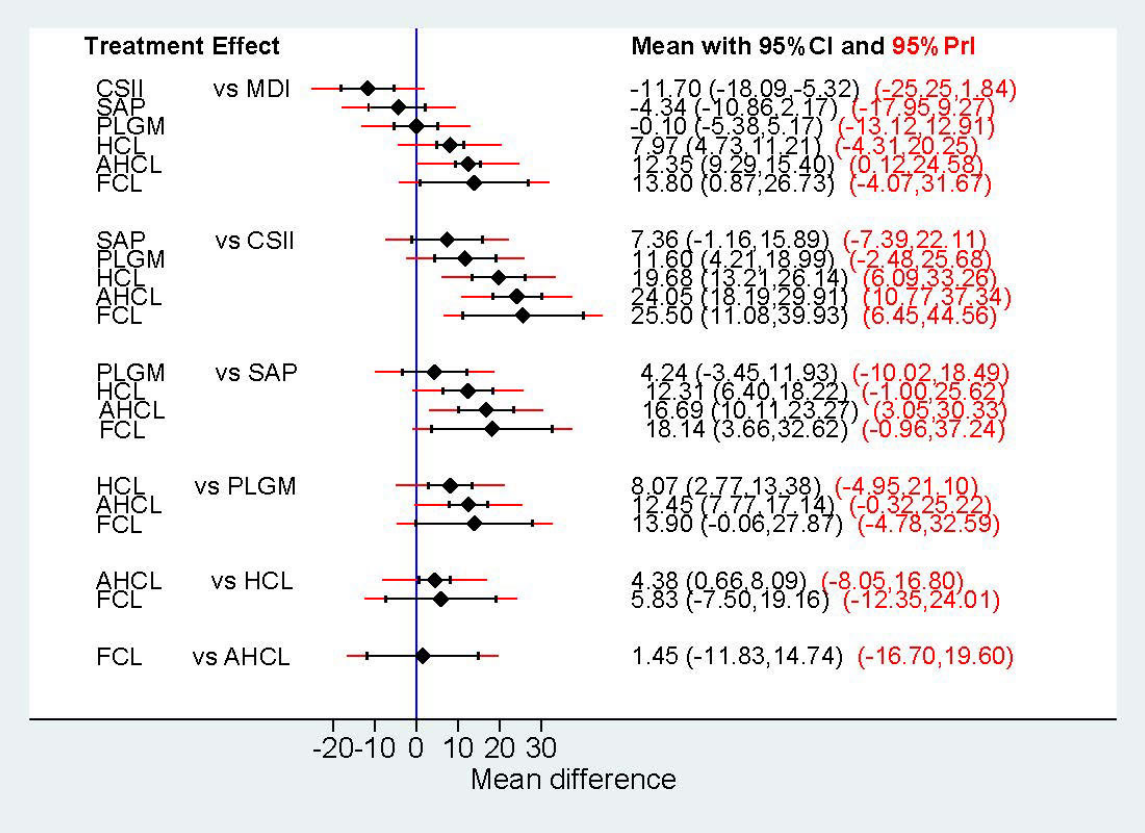

Figure G: Predictive interval plot for the TIR network

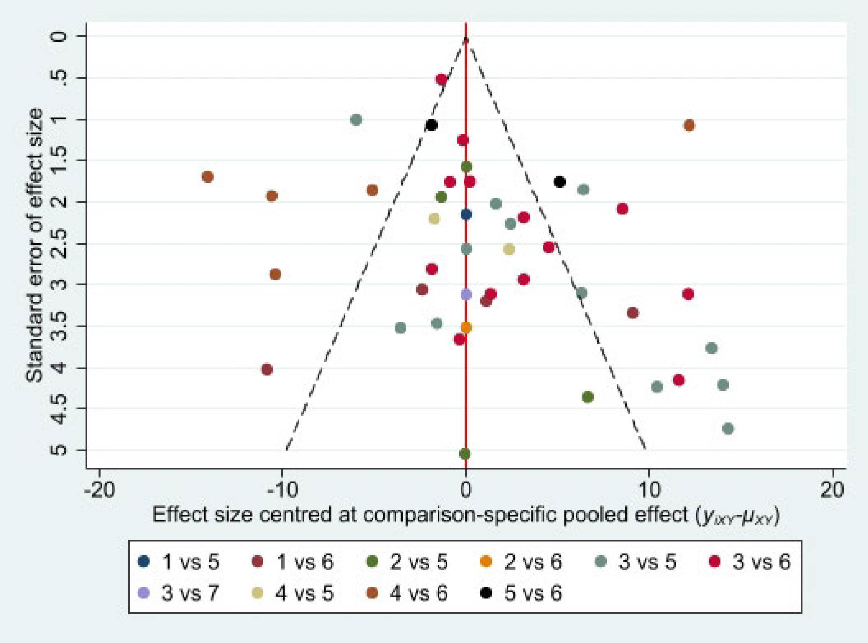

Coding	Treatment
1	MDI
2	CSII
3	SAP
4	PLGM
5	HCL
6	AHCL
7	FCL

Figure H: Comparison-adjusted funnel plot for the TIR network

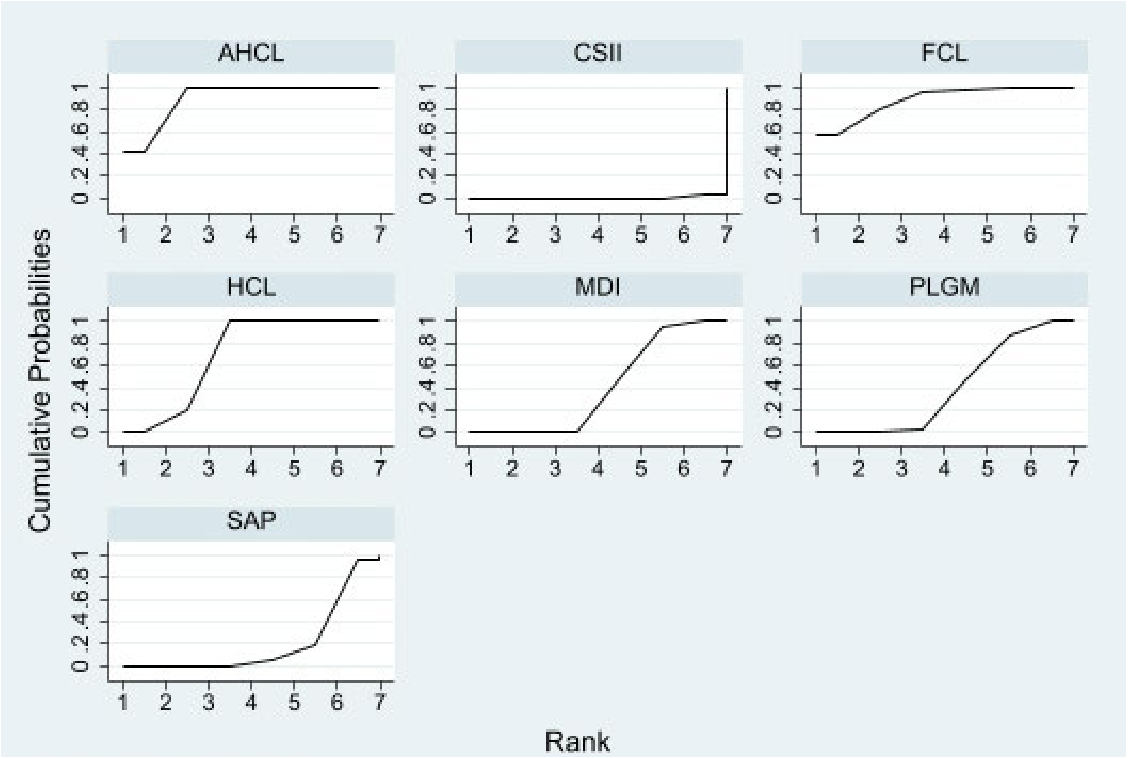

Figure I: Plots of the surface under the cumulative ranking curves for all treatments in the TIR network

Table A: League table of TIR network estimates

MDI	-11.70 (-18.09; -5.32)	-4.34 (-10.86; 2.17)	-0.10 (-5.38; 5.17)	7.97 (4.73; 11.21)	12.35 (9.29; 15.40)	13.80 (0.87; 26.73)
11.70 (5.32; 18.09)	CSII	7.36 (-1.16; 15.89)	11.60 (4.21; 18.99)	19.68 (13.21; 26.14)	24.05 (18.19; 29.91)	25.50 (11.08; 39.93)
4.34 (-2.17; 10.86)	-7.36 (-15.89; 1.16)	SAP	4.24 (-3.45; 11.93)	12.31 (6.40; 18.22)	16.69 (10.11; 23.27)	18.14 (3.66; 32.62)
0.10 (-5.17; 5.38)	-11.60 (-18.99; -4.21)	-4.24 (-11.93; 3.45)	PLGM	8.07 (2.77; 13.38)	12.45 (7.77; 17.14)	13.90 (-0.06; 27.87)
-7.97 (-11.21; -4.73)	-19.68 (-26.14; -13.21)	-12.31 (-18.22; -6.40)	-8.07 (-13.38; -2.77)	HCL	4.38 (0.66; 8.09)	5.83 (-7.50; 19.16)
-12.35 (-15.40; -9.29)	-24.05 (-29.91; -18.19)	-16.69 (-23.27; -10.11)	-12.45 (-17.14; -7.77)	-4.38 (-8.09; -0.66)	AHCL	1.45 (-11.83; 14.74)
-13.80 (-26.73; -0.87)	-25.50 (-39.93; -11.08)	-18.14 (-32.62; -3.66)	-13.90 (-27.87; 0.06)	-5.83 (-19.16; 7.50)	-1.45 (-14.74; 11.83)	FCL

Table B: Direct estimates, indirect estimates, and differences between direct and indirect estimates of the outcome TIR

**Side**	**Direct**		**Indirect**		**Difference**			
	**Estimate**	**SE**	**Estimate**	**SE**	**Estimate**	**SE**	**p**	**tau**
HCL vs. MDI	15.20	6.22	21.45	3.91	-6.25	7.35	0.396	5.84
HCL vs. CSII	12.96	3.36	9.41	7.13	3.56	7.88	0.652	5.88
HCL vs. SAP	8.74	1.92	5.71	3.30	3.03	3.82	0.427	5.84
HCL vs. PLGM	8.41	4.51	7.88	3.46	0.52	5.68	0.926	5.91
AHCL vs. MDI	25.37	3.38	19.12	6.53	6.25	7.35	0.396	5.84
AHCL vs. CSII	14.00	6.85	17.56	3.90	-3.56	7.88	0.652	5.88
AHCL vs. SAP	11.70	1.77	14.73	3.38	-3.03	3.82	0.427	5.84
AHCL vs. PLGM	12.32	2.78	12.84	4.95	-0.53	5.68	0.926	5.91
AHCL vs. HCL	7.41	4.27	3.61	2.13	3.79	4.77	0.427	5.86

Table C: Rankogram for the outcome TIR

Treatment	P-score	SUCRA	Mean Rank
MDI	0.0	40.7	4.6
CSII	0.0	0.8	7.0
SAP	0.0	20.5	5.8
PLGM	0.0	39.3	4.6
HCL	0.4	70.1	2.8
AHCL	41.3	90.1	1.6
FCL	58.3	88.8	1.7

9.2 Network meta-analysis TAR >180 mg/dl

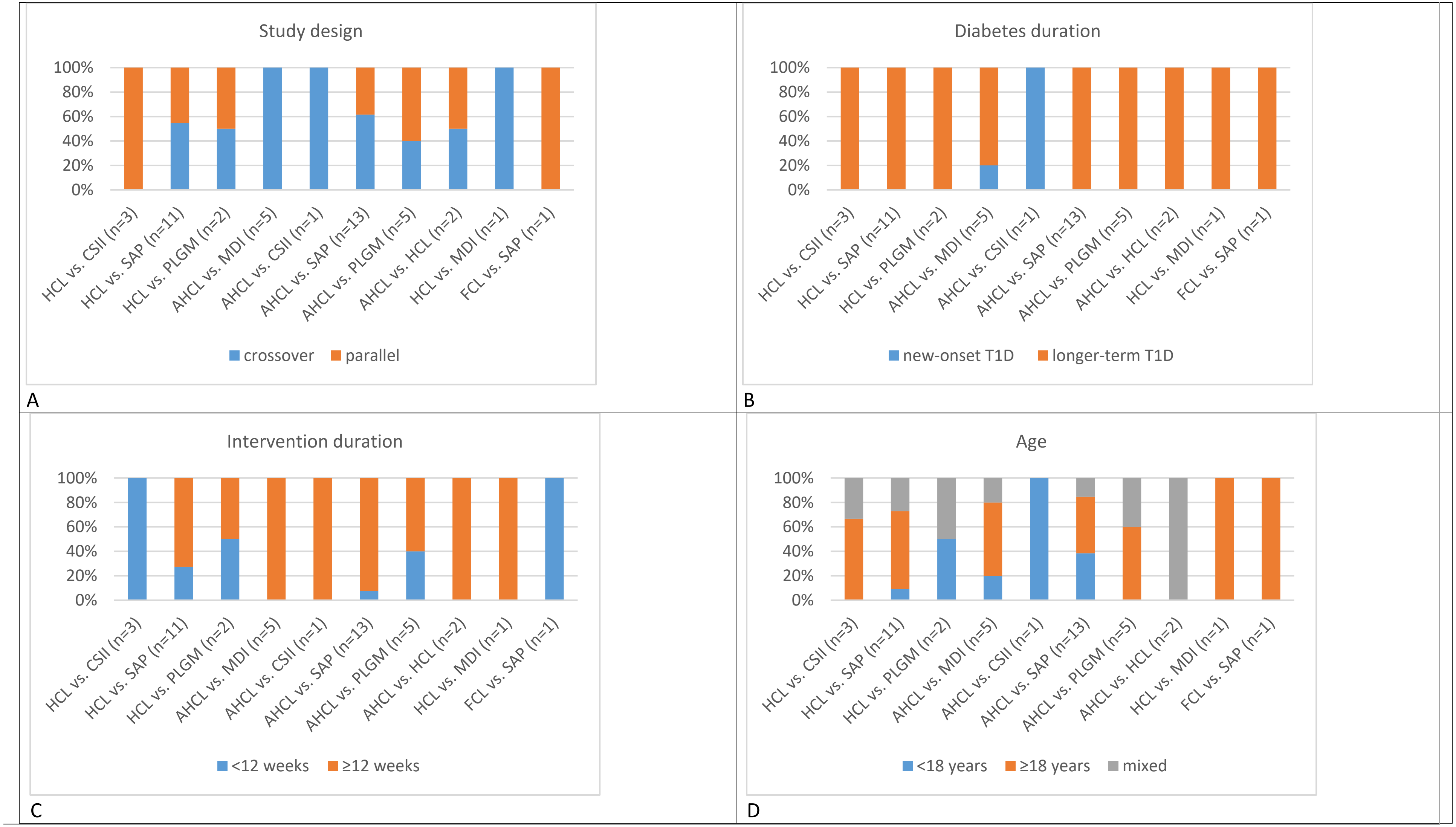

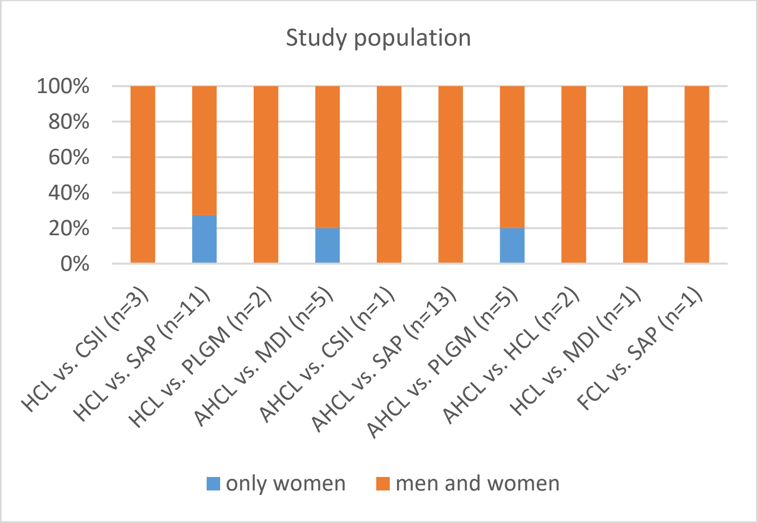

E

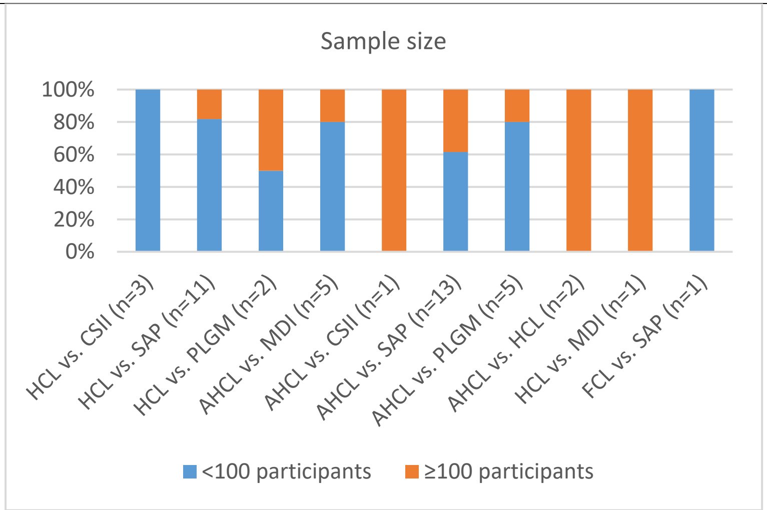

F

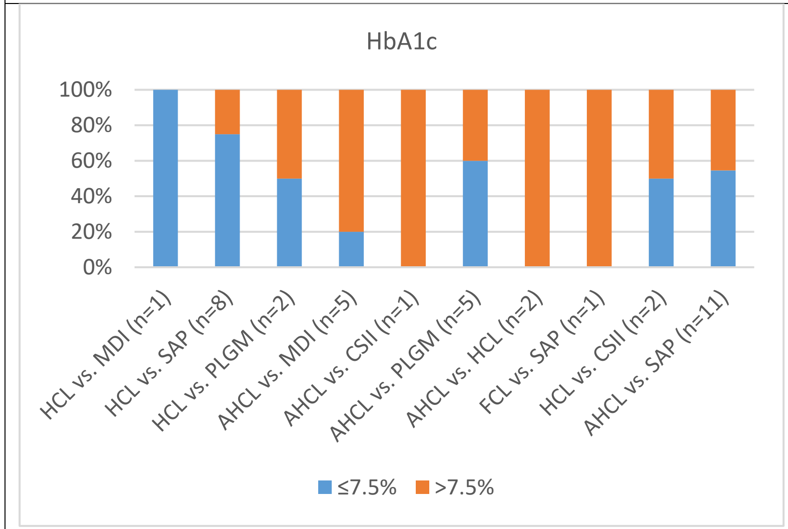

G

Figure A: Results of the transitivity assessment for time above range >180 mg/dl for (A) study design, (B) diabetes duration, (C) intervention duration, (D) age, (E) study population, (F) sample size, and (G) baseline HbA1c.

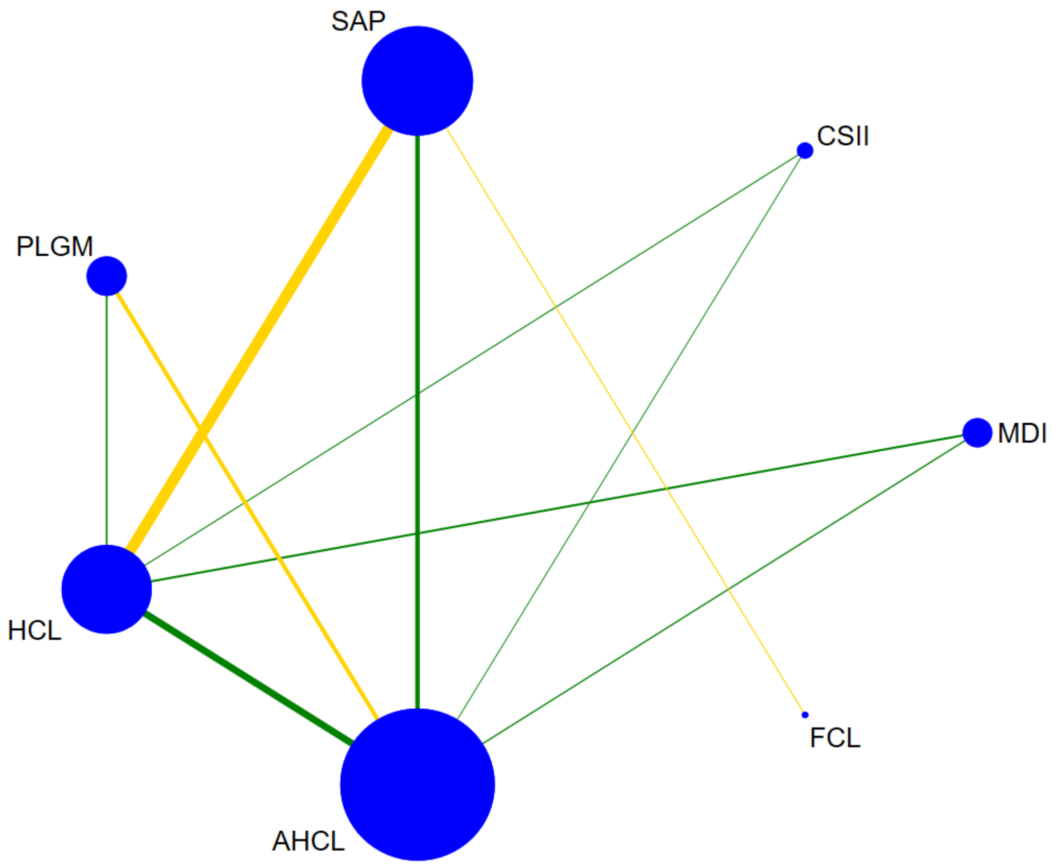

Number of studies	44
Number of treatments	7
Number of comparisons with direct evidence	10
Number of comparisons with indirect evidence	11
Density	0.5
Percentage of common comparators	85.7
Percentages of strong edges	70.0
Median thickness (IQR25; IQR75)	2.5 (1;5)

Figure B Plot and metrics of the TAR >180 mg/dl network

The node size in the network graph represents the number of participants with the respective intervention. The line width of the edge represents the mean of inverse variances (precision) of the treatment effect of the studies on which the direct comparison is based. The colour of the edge corresponds to the average level of the RoB estimated as the precision-weighted mean of the trials of the direct comparison.

Direct comparisons in the network

Network meta-analysis estimates	1vs5	1vs6	2vs5	2vs6	3vs5	3vs6	3vs7	4vs5	4vs6	5vs6
**Mixed estimates**										
1vs5	67.5	11.5	1.4	1.4	7.3	7.2		0.8	0.8	2.1
1vs6	32.2	9.2	3.8	3.8	20.3	20.4		2.1	2.1	5.9
2vs5	2.2	2.1	30.7	24.6	16.1	16.1		1.7	1.7	4.7
2vs6	2.1	2.2	24.6	30.7	16.1	16.2		1.7	1.7	4.7
3vs5	3.7	3.6	5.2	5.1	49.0	[illegible]	0.1	2.9	2.9	8.0
3vs6	2.0	2.0	2.9	2.9	10.9	71.6	[illegible]	1.6	1.6	4.4
3vs7	0.1			0.1	0.3	0.3	99.2	0.1		
4vs5	1.6	1.5	2.2	2.1	11.4	11.3	0.1	48.3	18.2	3.3
4vs6	2.5	2.5	3.5	3.5	18.6	18.7	0.1	30.0	15.1	5.4
5vs6	4.3	4.2	6.0	6.0	31.8	31.8		3.3	3.3	9.3
**Indirect estimates**										
1vs2	32.7	7.4	21.5	18.5	8.0	8.0		0.8	0.8	2.3
1vs3	35.3	8.7	2.5	2.5	27.4	16.6	0.1	1.4	1.4	3.9
1vs4	37.7	7.6	0.7	0.7	3.6	3.5		32.4	12.8	1.0
1vs7	24.6	6.1	1.8	1.8	19.0	11.5	30.6	1.0	1.0	2.7
2vs3	0.7	0.7	23.0	25.0	20.3	27.7	0.1	0.5	0.5	1.5
2vs4	0.7	0.7	22.6	20.7	5.2	5.3		30.1	13.2	1.5
2vs7	0.5	0.5	15.6	16.9	13.7	18.7	32.4	0.4	0.4	1.0
3vs4	1.4	1.4	1.9	2.0	24.6	20.6	0.1	30.9	14.3	3.0
4vs7	1.0	0.9	1.3	1.3	16.9	14.2	31.1	21.3	9.8	2.1
5vs7	2.2	2.2	3.1	3.0	29.1	11.6	40.7	1.7	1.7	4.7
6vs7	1.1	1.1	1.6	1.6	5.9	39.3	45.2	0.8	0.9	2.5
**Entire network**	12.1	3.6	8.5	8.5	17.1	17.7	13.5	10.3	5.1	3.4
**Included studies**	1	5	3	1	11	13	1	2	5	2

Coding	Treatment
1	MDI
2	CSII
3	SAP
4	PLGM
5	HCL
6	AHCL
7	FCL

Figure C Contribution matrix for the TAR >180 mg/dl network

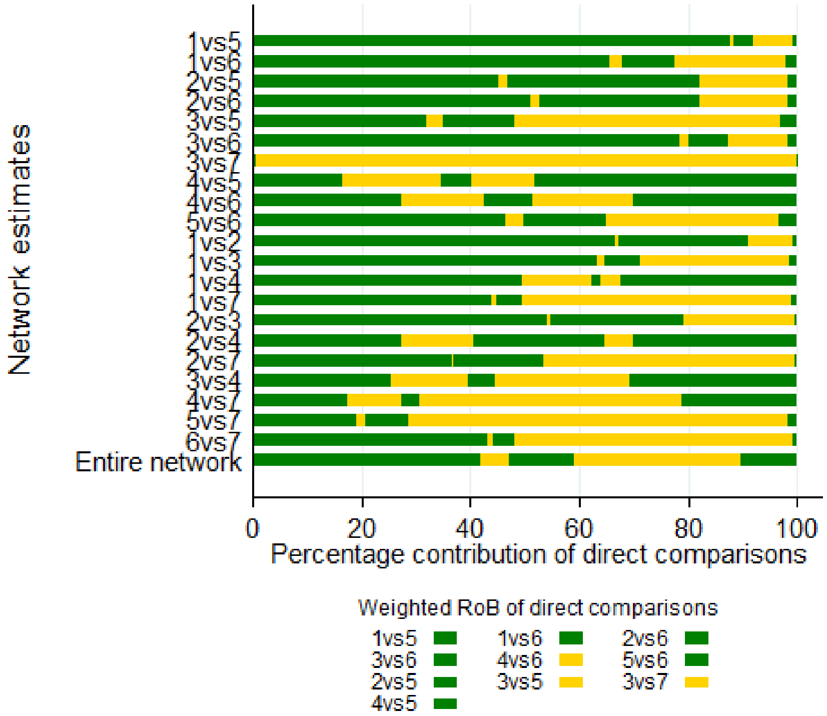

Coding	Treatment
1	MDI
2	CSII
3	SAP
4	PLGM
5	HCL
6	AHCL
7	FCL

Figure D Study limitations for each network estimate for pairwise comparisons in the TAR >180 mg/dl network

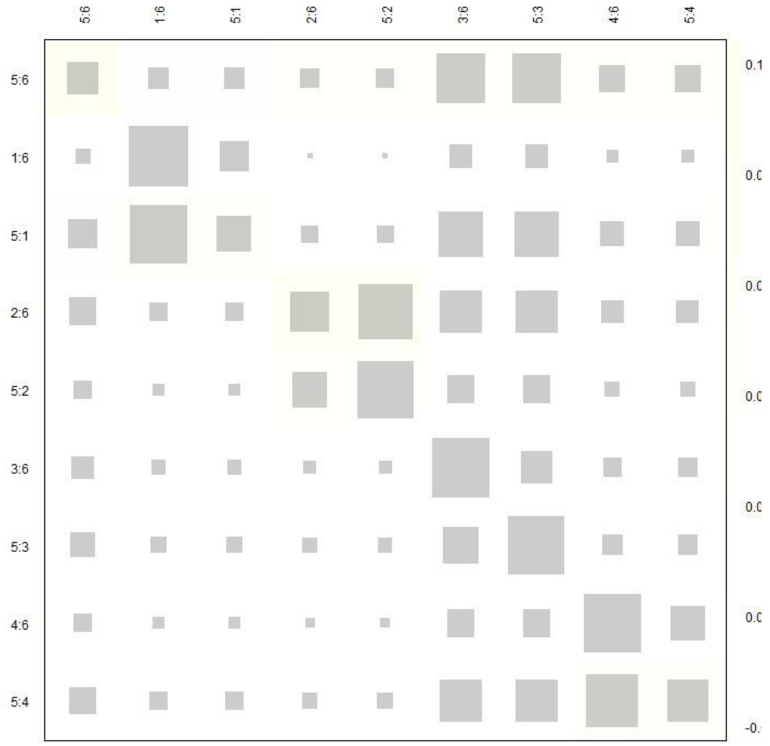

Coding	Treatment
1	MDI
2	CSII
3	SAP
4	PLGM
5	HCL
6	AHCL
7	FCL

Figure E Net heat plot

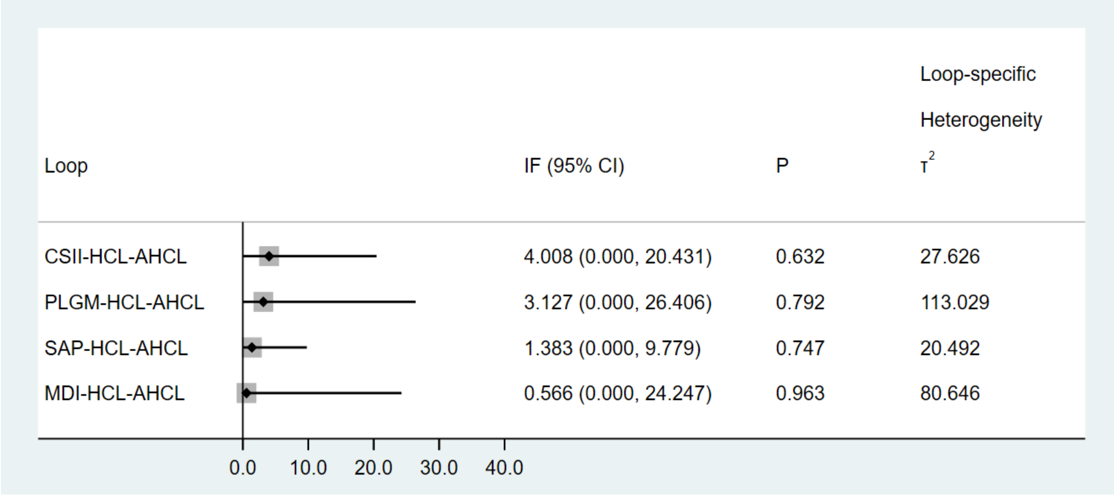

Figure F Inconsistency plot for the TAR >180 mg/dl network assuming loop-specific heterogeneity estimates

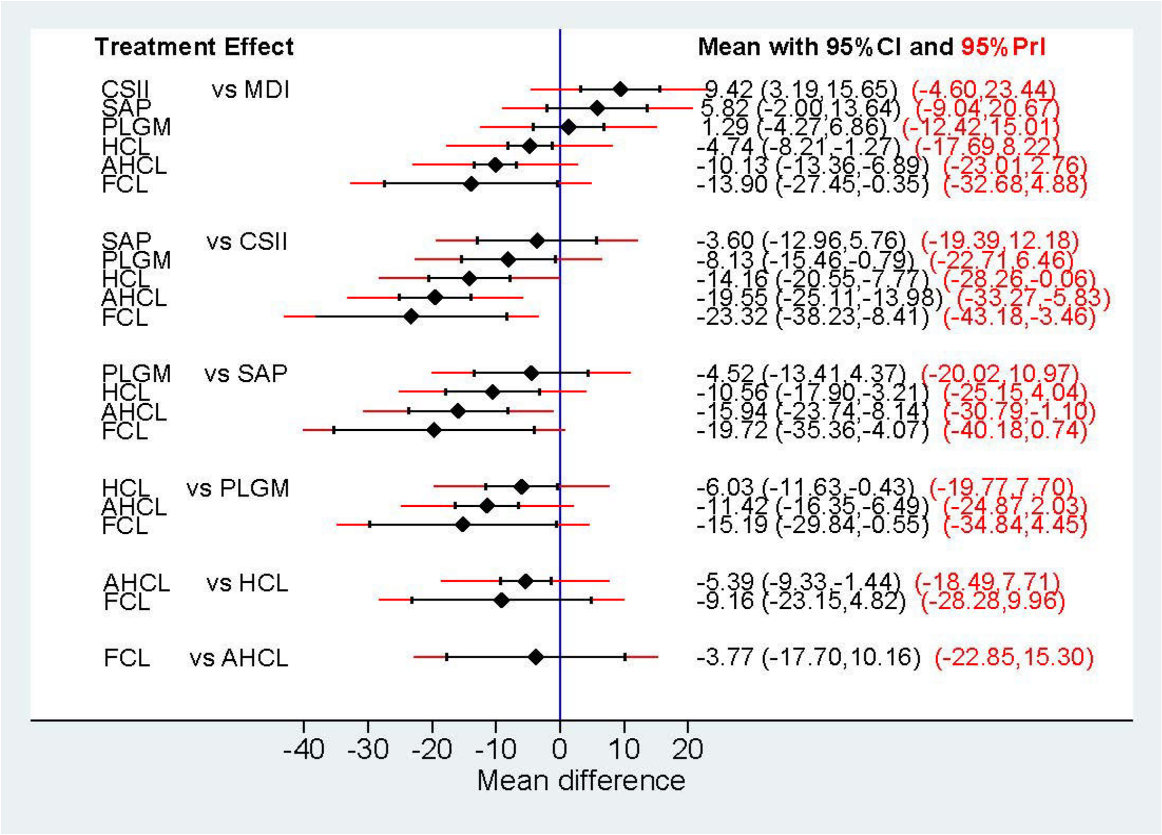

Figure G Predictive interval plot for the TAR >180 mg/dl network

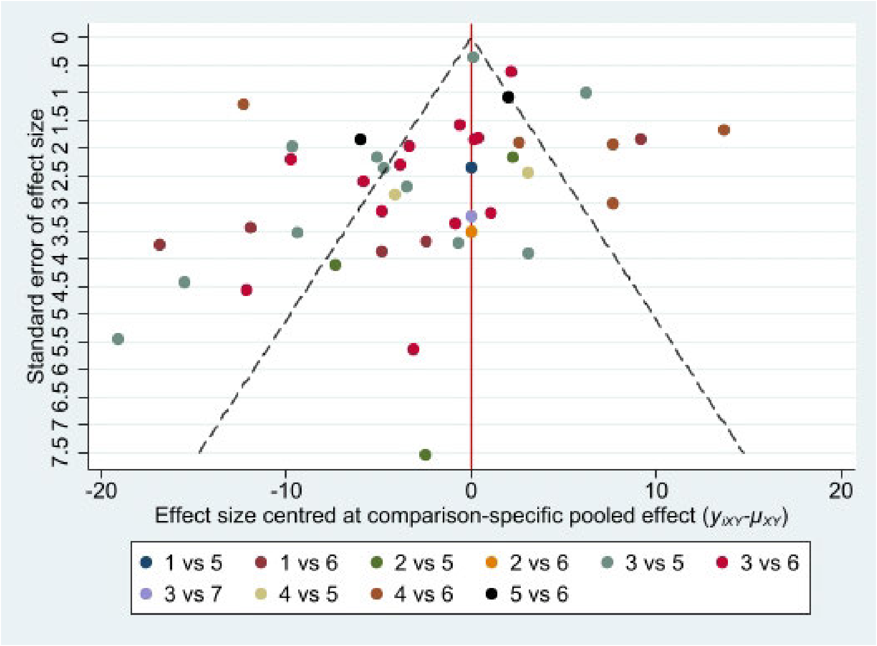

Coding	Treatment
1	MDI
2	CSII
3	SAP
4	PLGM
5	HCL
6	AHCL
7	FCL

Figure H Comparison-adjusted funnel plot for the TAR >180 mg/dl network

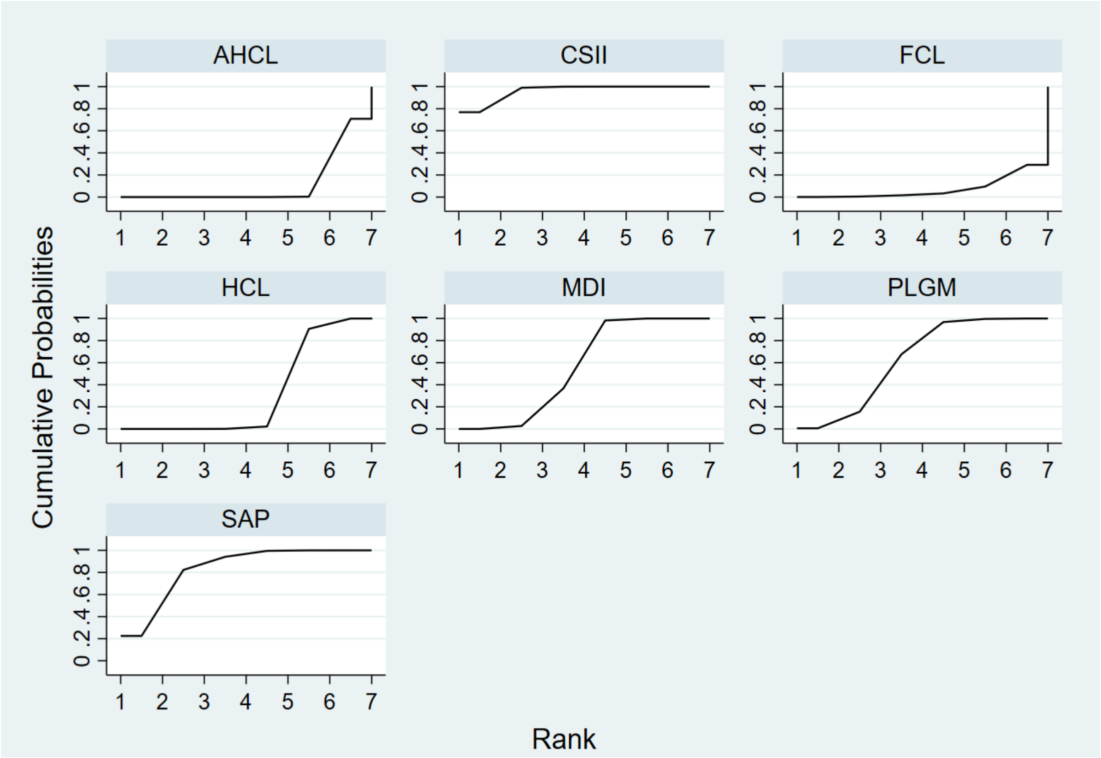

Figure I Plots of the surface under the cumulative ranking curves for all treatments in the TAR >180 mg/dl network

Table A League table of TAR >180 mg/dl network estimates

MDI	9.42 (3.19; 15.65)	5.82 (-2.00; 13.64)	1.29 (-4.27; 6.86)	-4.74 (-8.21; -1.27)	-10.13 (-13.36; -6.89)	-13.90 (-27.45; -0.35)
-9.42 (-15.65; -3.19)	CSII	-3.60 (-12.96; 5.76)	-8.13 (-15.46; -0.79)	-14.16 (-20.55; -7.77)	-19.55 (-25.11; -13.98)	-23.32 (-38.23; -8.41)
-5.82 (-13.64; 2.00)	3.60 (-5.76; 12.96)	SAP	-4.52 (-13.41; 4.37)	-10.56 (-17.90; -3.21)	-15.94 (-23.74; -8.14)	-19.72 (-35.36; -4.07)
-1.29 (-6.86; 4.27)	8.13 (0.79; 15.46)	4.52 (-4.37; 13.41)	PLGM	-6.03 (-11.63; -0.43)	-11.42 (-16.35; -6.49)	-15.19 (-29.84; -0.55)
4.74 (1.27; 8.21)	14.16 (7.77; 20.55)	10.56 (3.21; 17.90)	6.03 (0.43; 11.63)	HCL	-5.39 (-9.33; -1.44)	-9.16 (-23.15; 4.82)
10.13 (6.89; 13.36)	19.55 (13.98; 25.11)	15.94 (8.14; 23.74)	11.42 (6.49; 16.35)	5.39 (1.44; 9.33)	AHCL	-3.77 (-17.70; 10.16)
13.90 (0.35; 27.45)	23.32 (8.41; 38.23)	19.72 (4.07; 35.36)	15.19 (0.55; 29.84)	9.16 (-4.82; 23.15)	3.77 (-10.16; 17.70)	FCL

Table B Direct estimates, indirect estimates, and differences between direct and indirect estimates of the outcome TAR >180 mg/dl

Side	Direct		Indirect		Difference			
	**Estimate**	**SE**	**Estimate**	**SE**	**Estimate**	**SE**	**p**	**tau**
HCL vs. MDI	-12.70	6.64	-14.67	3.81	1.96	7.66	0.797	6.21
HCL vs. CSII	-11.31	4.39	-8.45	7.421	-2.86	8.63	0.740	6.19
HCL vs. SAP	-4.86	2.09	-4.43	3.52	-0.43	4.09	0.916	6.22
HCL vs. PLGM	-7.02	4.77	-5.46	3.65	-1.56	6.00	0.795	6.21
AHCL vs. MDI	-19.89	3.16	-17.93	7.00	-1.96	7.66	0.797	6.21
AHCL vs. CSII	-14.00	7.12	-16.86	4.87	2.86	8.63	0.740	6.19
AHCL vs. SAP	-10.04	1.89	-10.47	3.63	0.43	4.09	0.916	6.22
AHCL vs. PLGM	-11.05	2.92	-12.61	5.25	1.56	6.00	0.795	6.21
AHCL vs. HCL	-6.89	4.52	-5.00	2.29	-1.89	5.06	0.708	6.21

Table C Rankogram for the outcome TAR >180 mg/dl

Treatment	P-score	SUCRA	Mean Rank
MDI	0.0	56.2	3.6
CSII	76.8	96.0	1.2
SAP	22.5	83.1	2.0
PLGM	0.6	63.4	3.2
HCL	0.0	32.1	5.1
AHCL	0.0	11.9	6.3
FCL	0.1	7.4	6.6

9.3 Network meta-analysis TAR >250 mg/dl

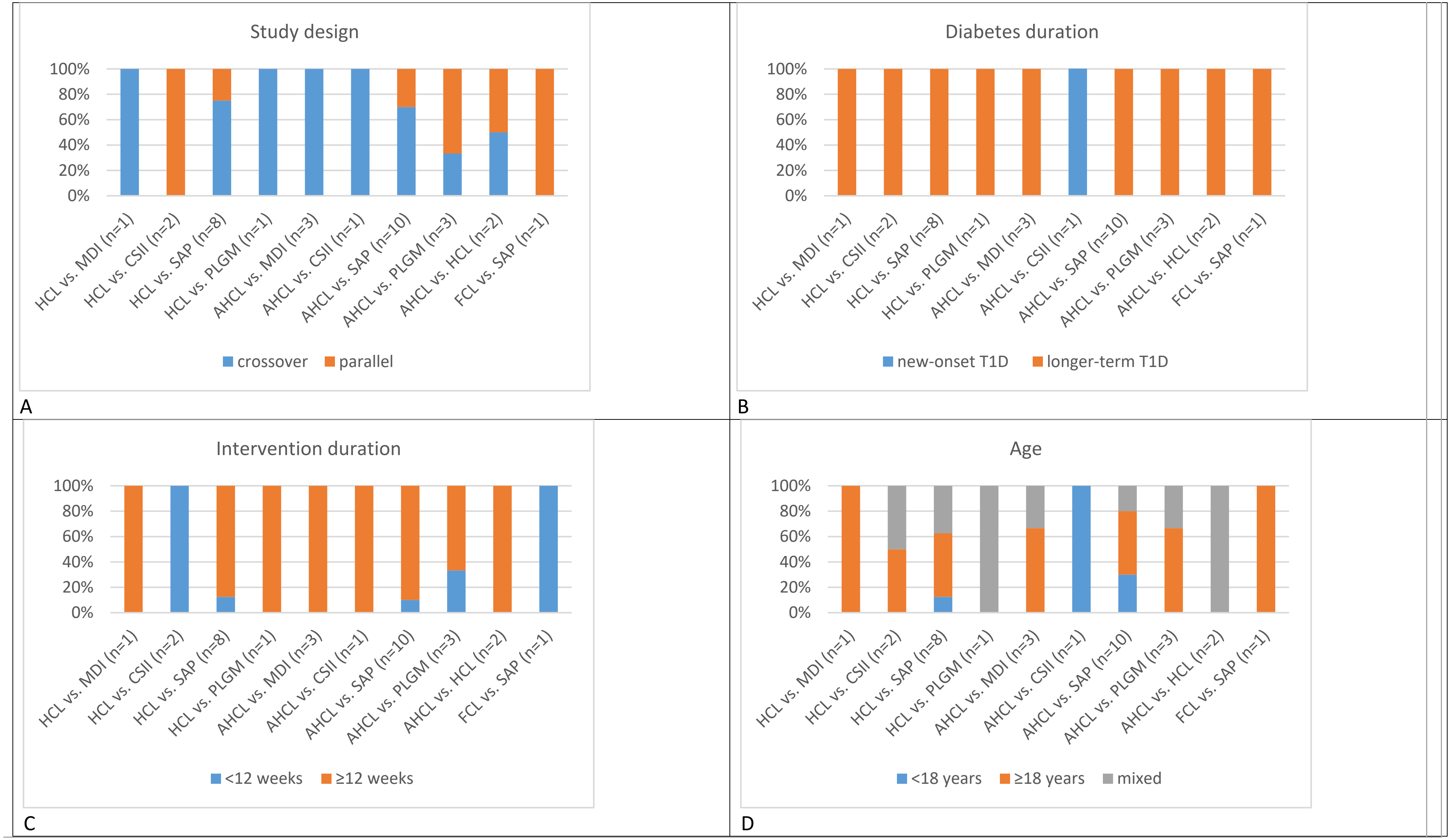

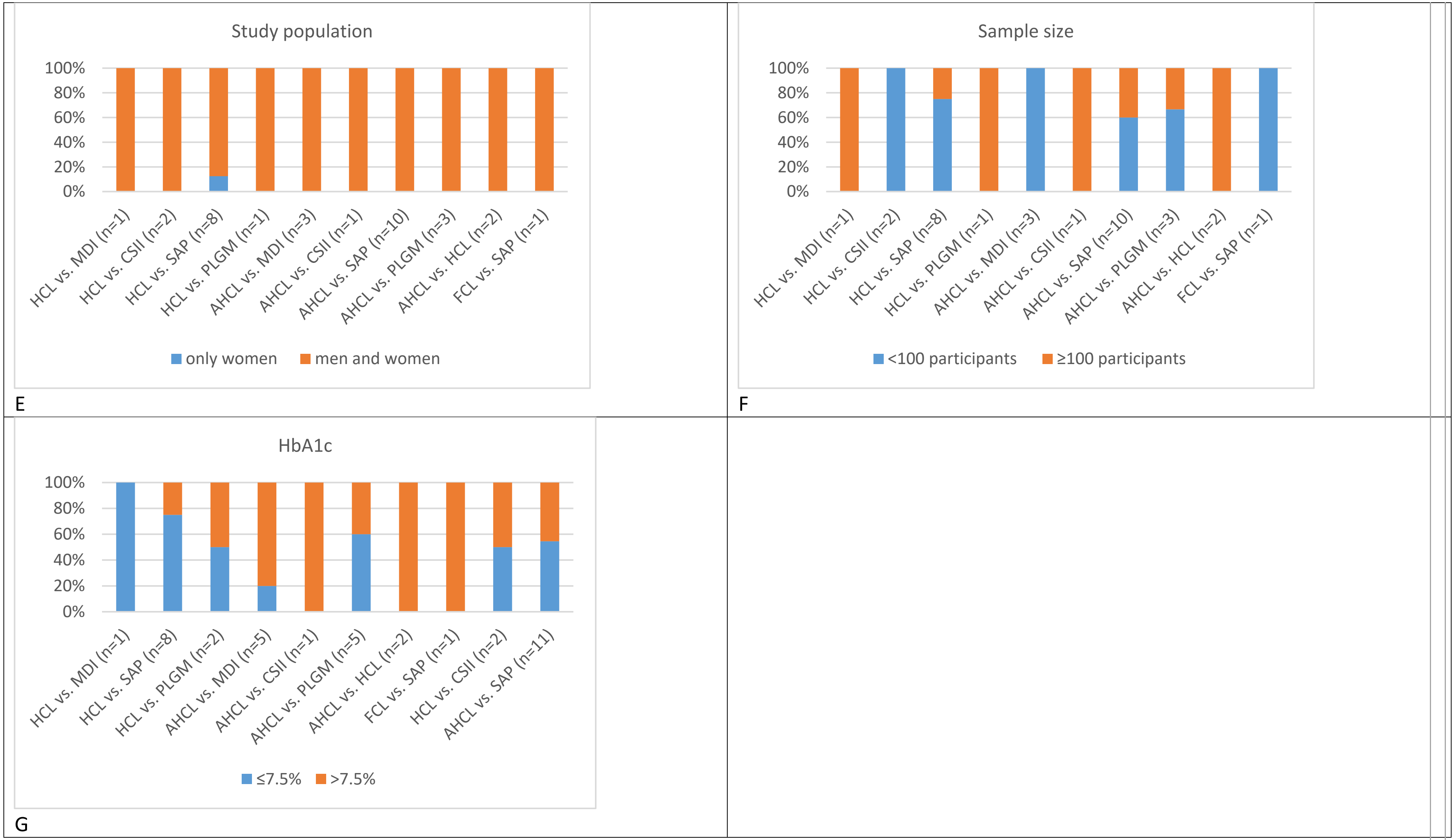

Figure A: Results of the transitivity assessment for time above range >250 mg/dl for (A) study design, (B) diabetes duration, (C) intervention duration, (D) age, (E) study population, (F) sample size, and (G) baseline HbA1c.

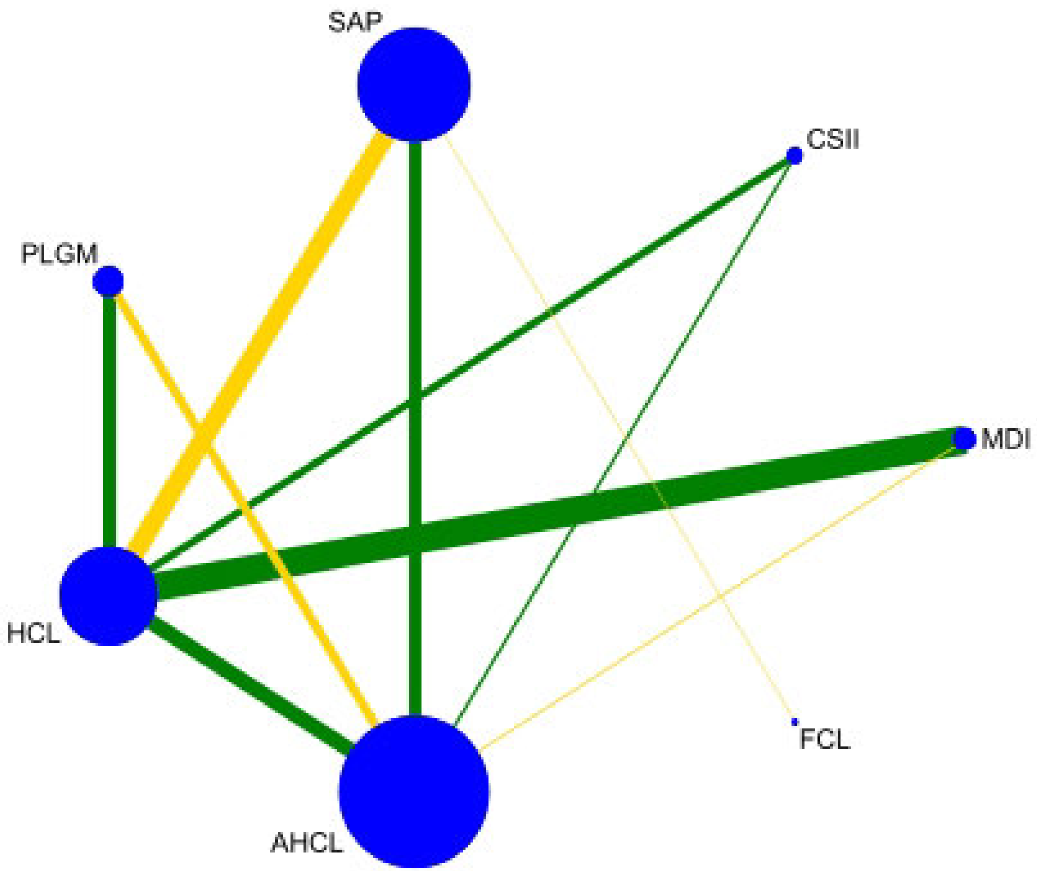

Number of studies	33
Number of treatments	7
Number of comparisons with direct evidence	10
Number of comparisons with indirect evidence	11
Density	0.5
Percentage of common comparators	85.7
Percentages of strong edges	60
Median thickness (IQR25; IQR75)	2 (1;3)

Figure B Plot and metrics of the TAR >250 mg/dl network

The node size in the network graph represents the number of participants with the respective intervention. The line width of the edge represents the mean of inverse variances (precision) of the treatment effect of the studies on which the direct comparison is based. The colour of the edge corresponds to the average level of the RoB estimated as the precision-weighted mean of the trials of the direct comparison.

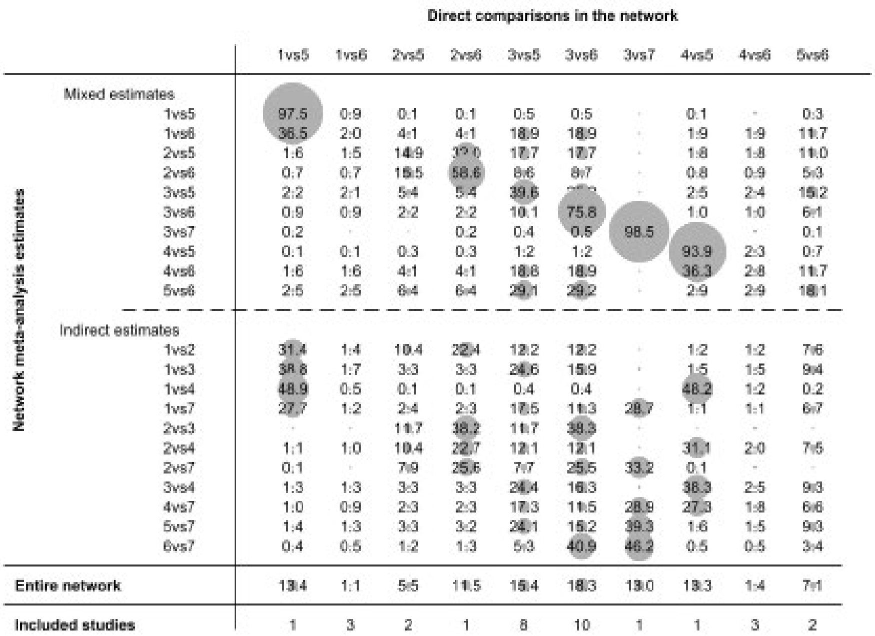

Coding	Treatment
1	MDI
2	CSII
3	SAP
4	PLGM
5	HCL
6	AHCL
7	FCL

Figure C Contribution matrix for the TAR >250 mg/dl network

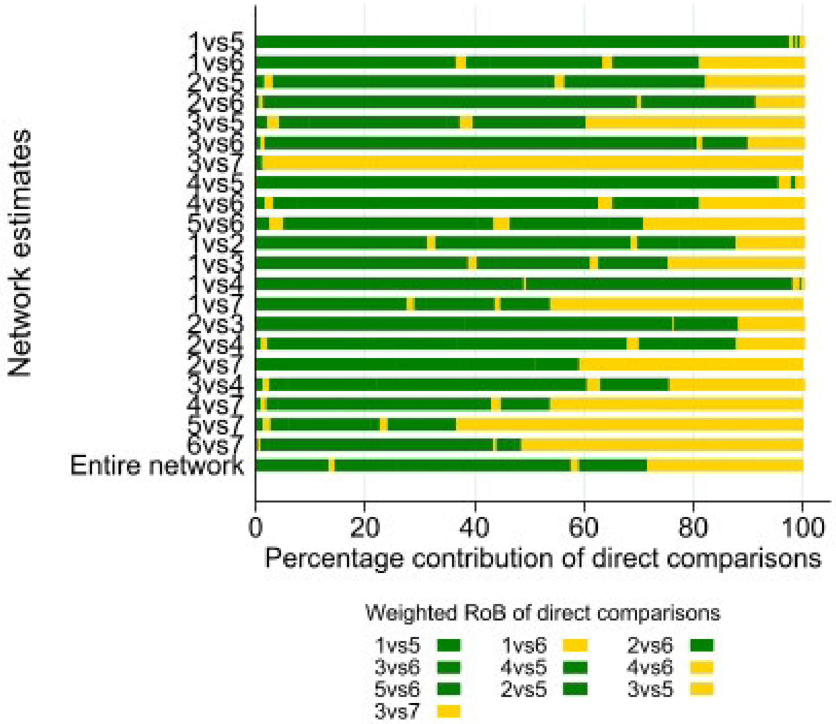

Coding	Treatment
1	MDI
2	CSII
3	SAP
4	PLGM
5	HCL
6	AHCL
7	FCL

Figure D Study limitations for each network estimate for pairwise comparisons in the TAR >250 mg/dl network

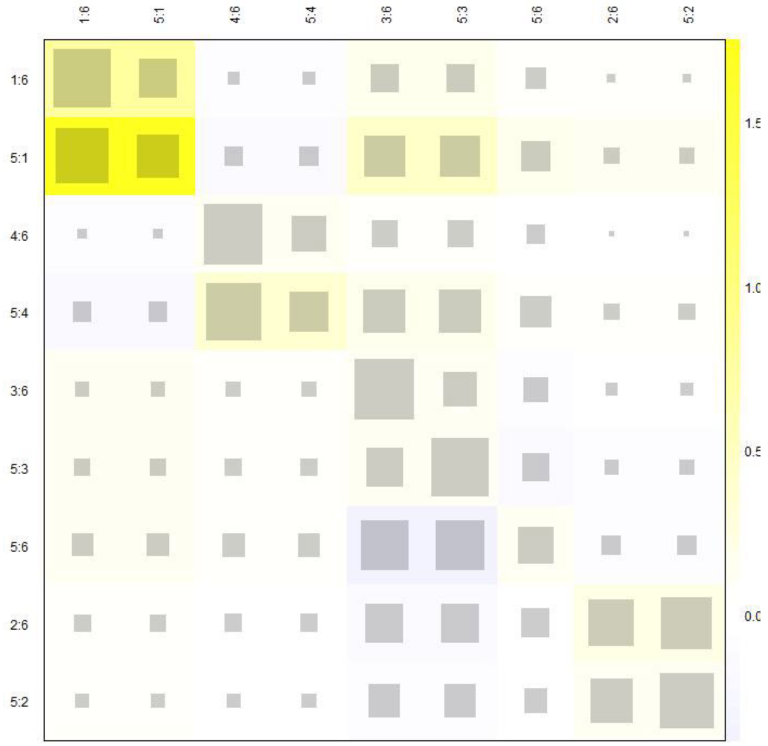

Coding	Treatment
1	MDI
2	CSII
3	SAP
4	PLGM
5	HCL
6	AHCL
7	FCL

Figure E Net heat plot

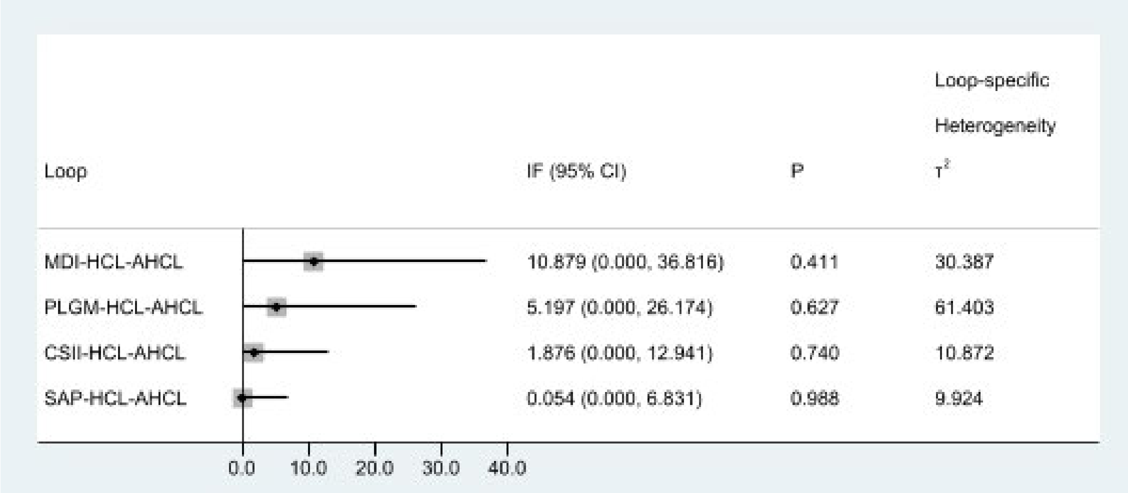

Figure F Inconsistency plot for the TAR >250 mg/dl network assuming loop-specific heterogeneity estimates

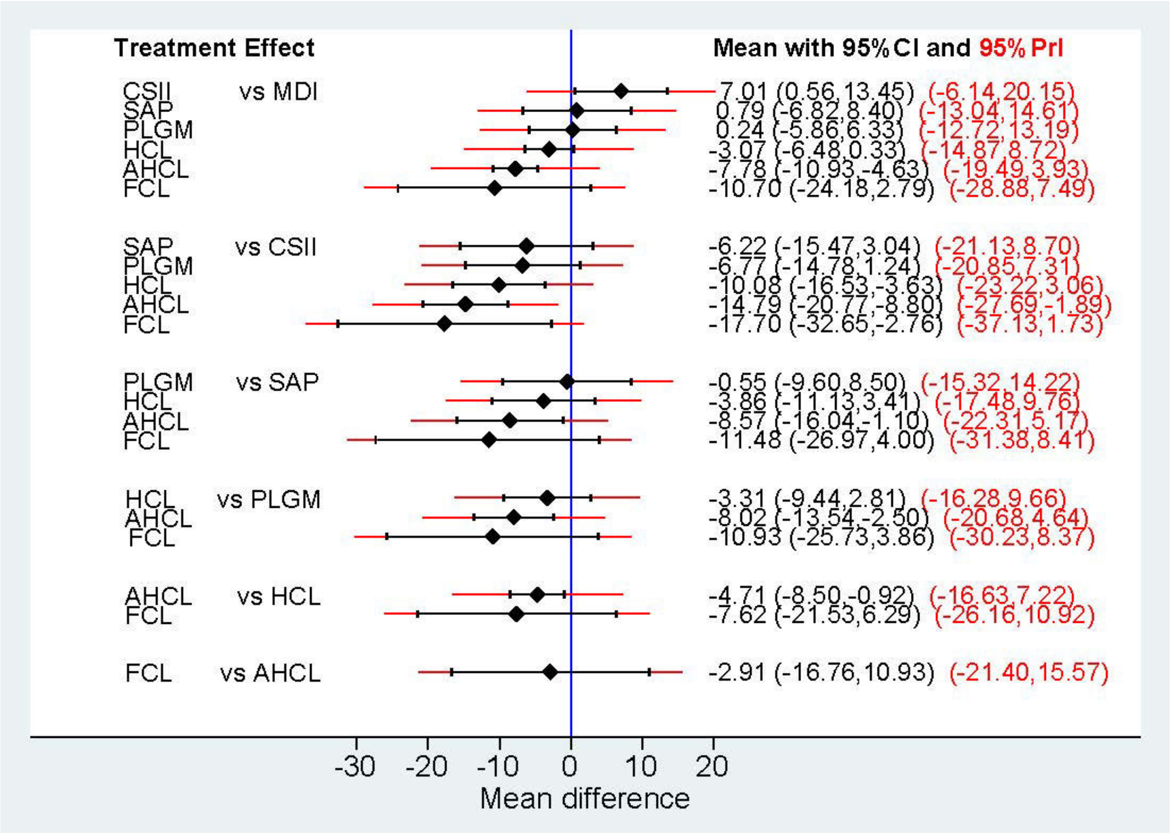

Figure G Predictive interval plot for the TAR >250 mg/dl network

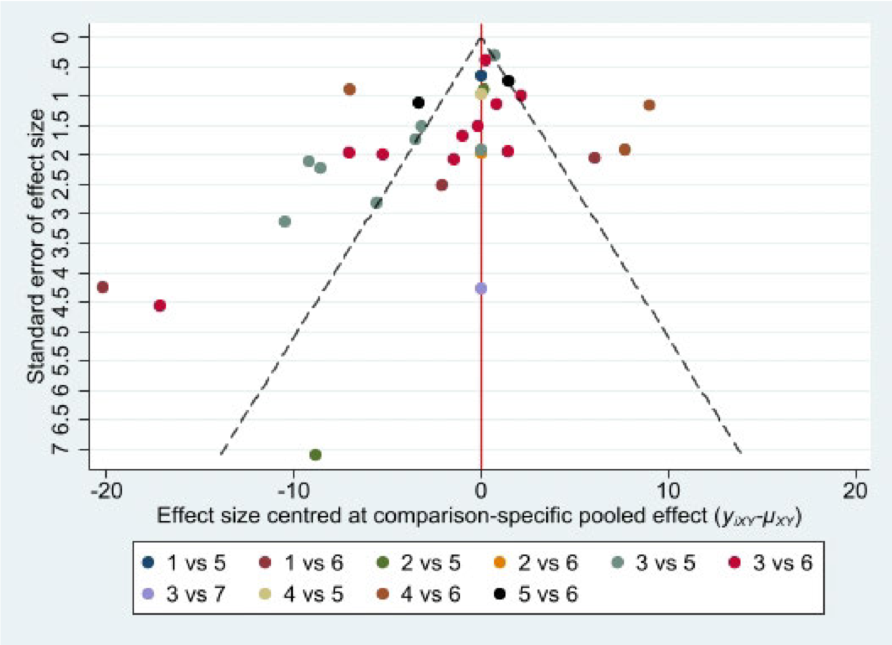

Coding	Treatment
1	MDI
2	CSII
3	SAP
4	PLGM
5	HCL
6	AHCL
7	FCL

Figure H Comparison-adjusted funnel plot for the TAR >250 mg/dl network

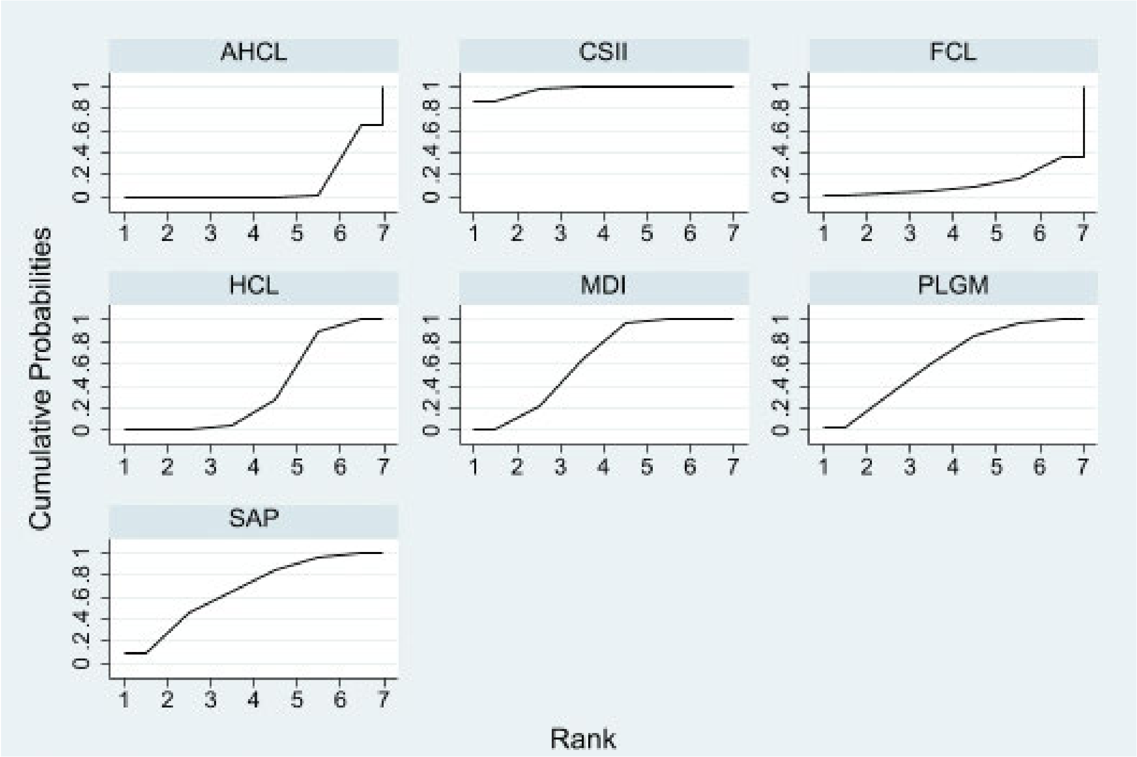

Figure I Plots of the surface under the cumulative ranking curves for all treatments in the TAR >250 mg/dl network

Table A League table of TAR >250 mg/dl network estimates

MDI	7.01 (0.56; 13.45)	0.79 (-6.82; 8.40)	0.24 (-5.86; 6.33)	-3.07 (-6.48; 0.33)	-7.78 (-10.93; -4.63)	-10.70 (-24.18; 2.79)
-7.01 (-13.45; -0.56)	CSII	-6.22 (-15.47; 3.04)	-6.77 (-14.78; 1.24)	-10.08 (-16.53; -3.63)	-14.79 (-20.77; -8.80)	-17.70 (-32.65; -2.76)
-0.79 (-8.40; 6.82)	6.22 (-3.04; 15.47)	SAP	-0.55 (-9.60; 8.50)	-3.86 (-11.13; 3.41)	-8.57 (-16.04; -1.10)	-11.48 (-26.97; 4.00)
-0.24 (-6.33; 5.86)	6.77 (-1.24; 14.78)	0.55 (-8.50; 9.60)	PLGM	-3.31 (-9.44; 2.81)	-8.02 (-13.54; -2.50)	-10.93 (-25.73; 3.86)
3.07 (-0.33; 6.48)	10.08 (3.63; 16.53)	3.86 (-3.41; 11.13)	3.31 (-2.81; 9.44)	HCL	-4.71 (-8.50; -0.92)	-7.62 (-21.53; 6.29)
7.78 (4.63; 10.93)	14.79 (8.80; 20.77)	8.57 (1.10; 16.04)	8.02 (2.50; 13.54)	4.71 (0.92; 8.50)	AHCL	-2.91 (-16.76; 10.93)
10.70 (-2.79; 24.18)	17.70 (2.76; 32.65)	11.48 (-4.00; 26.97)	10.93 (-3.86; 25.73)	7.62 (-6.29; 21.53)	2.91 (-10.93; 16.76)	FCL

Table B Direct estimates, indirect estimates, network estimates and differences between direct and indirect estimates of the outcome TAR >250 mg/dl

Side	Direct		Indirect		Difference			
	Estimate	SE	Estimate	SE	Estimate	SE	p	tau
HCL vs. MDI	-3.41	5.31	-13.80	4.03	10.38	6.66	0.119	5.27
HCL vs. CSII	-5.61	4.76	-0.97	6.21	-4.63	7.83	0.554	5.52
HCL vs. SAP	-3.74	2.07	-1.38	3.34	-2.36	3.93	0.548	5.49
HCL vs. PLGM	-0.36	5.56	-4.73	3.86	4.37	6.77	0.519	5.48
AHCL vs. MDI	-17.58	3.50	-7.19	5.67	-10.38	6.66	0.119	5.27
AHCL vs. CSII	-6.00	5.86	-10.63	5.19	4.63	7.83	0.554	5.52
AHCL vs. SAP	-7.28	1.84	-9.63	3.47	2.36	3.93	0.548	5.49
AHCL vs. PLGM	-9.03	3.26	-4.66	5.93	-4.37	6.77	0.519	5.48
AHCL vs. HCL	-3.38	3.97	-5.15	2.28	1.76	4.58	0.700	5.53

Table C Rankogram for the outcome TAR >250 mg/dl

Treatment	P-score	SUCRA	Mean Rank
MDI	0.4	63.6	3.2
CSII	86.5	97.2	1.2
SAP	8.6	66.3	3.0
PLGM	3.5	63.0	3.2
HCL	0.0	36.6	4.8
AHCL	0.0	11.2	6.3
FCL	1.0	12.1	6.3

9.4 Network meta-analysis TBR <70 mg/dl

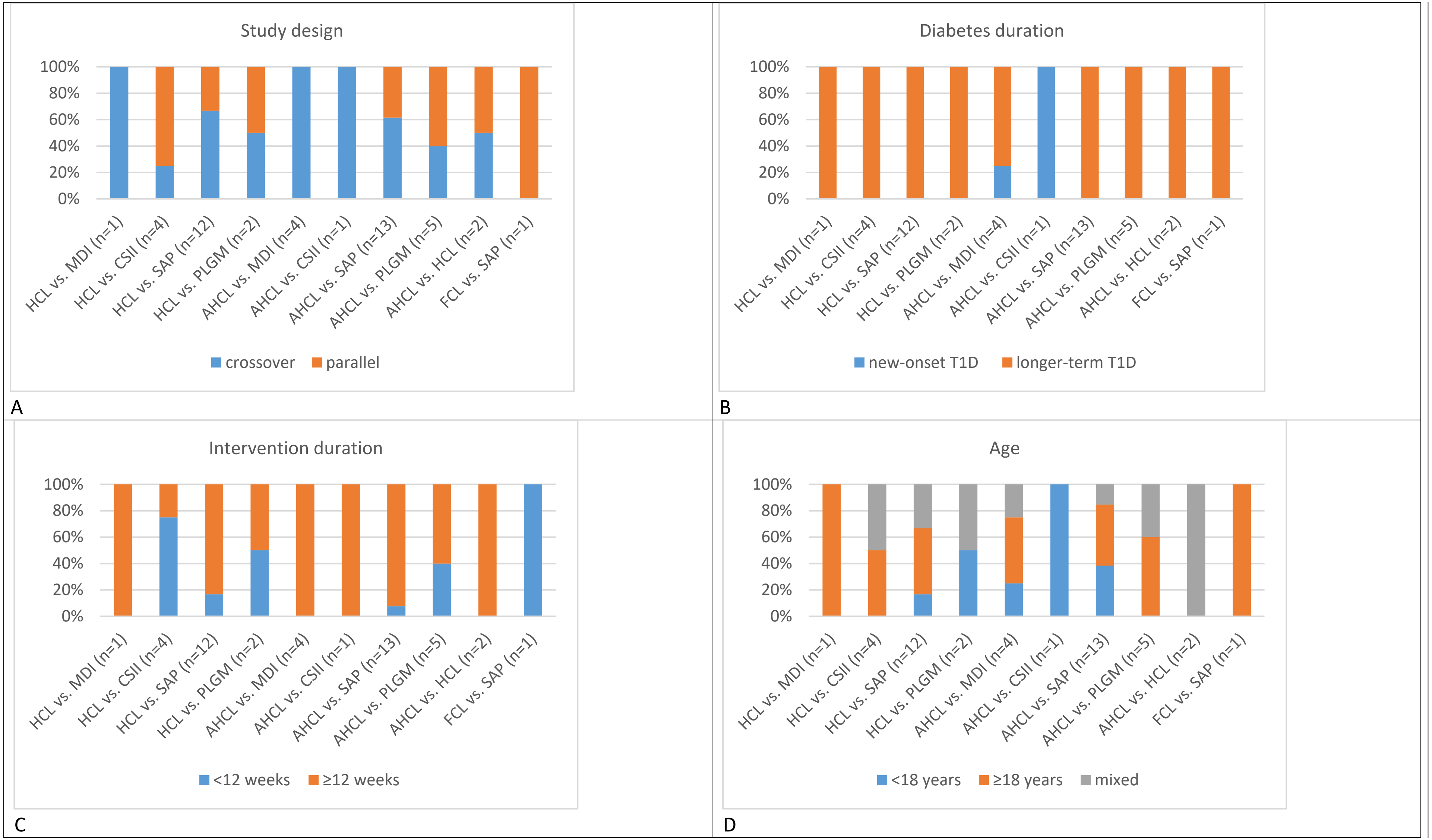

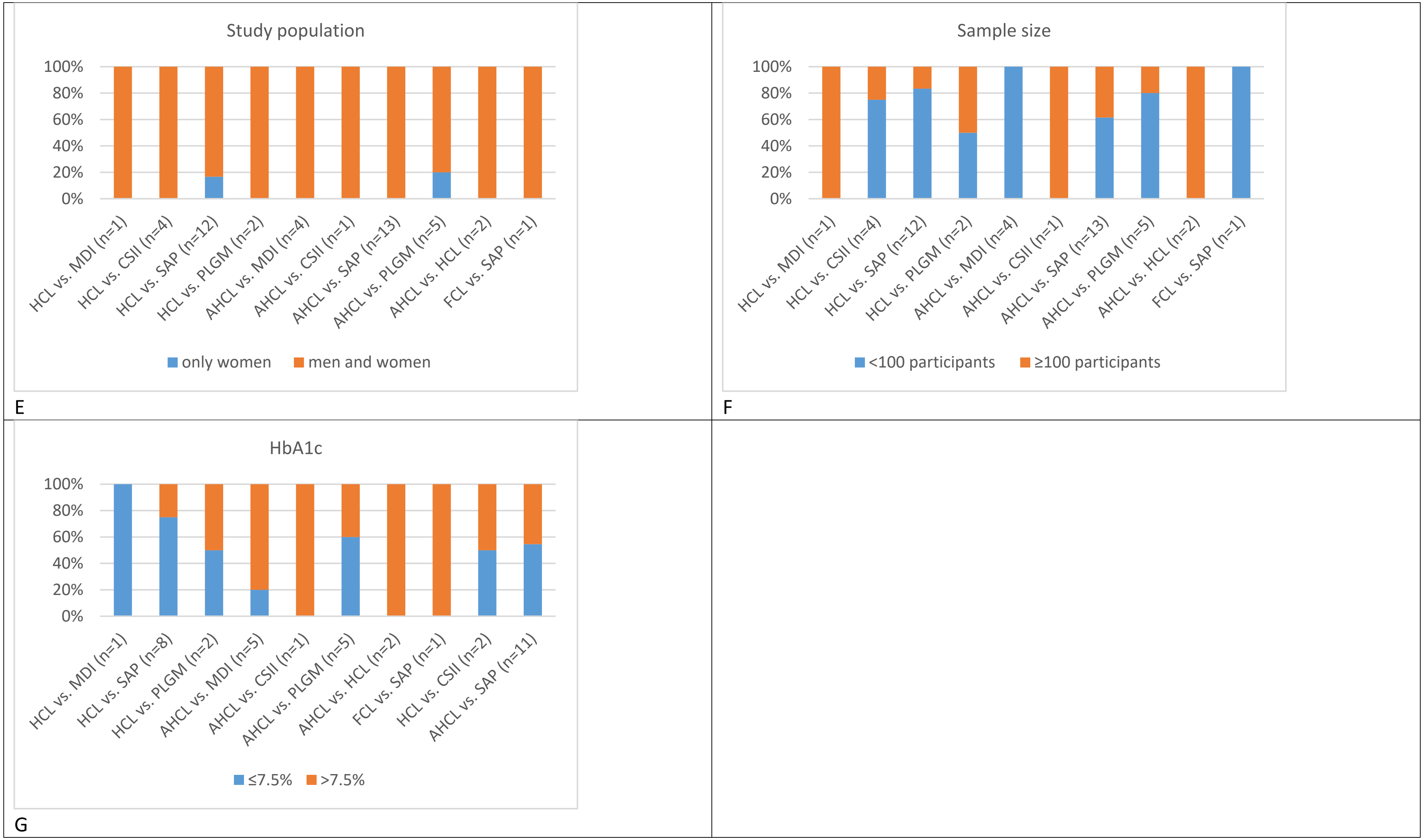

Figure A: Results of the transitivity assessment for time below range <70 mg/dl for (A) study design, (B) diabetes duration, (C) intervention duration, (D) age, (E) study population, (F) sample size, and (G) baseline HbA1c.

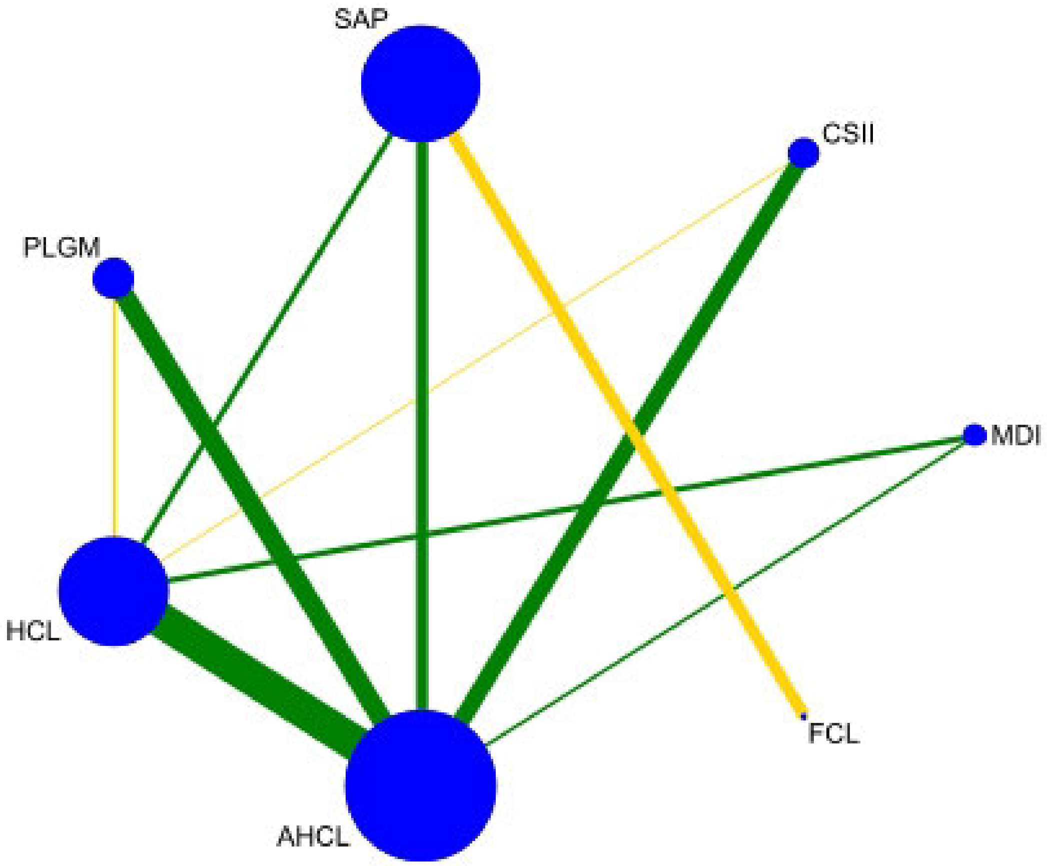

Number of studies	45
Number of treatments	7
Number of comparisons with direct evidence	10
Number of comparisons with indirect evidence	11
Density	0.5
Percentage of common comparators	85,7
Percentages of strong edges	63,6
Median thickness (IQR25; IQR75)	3 (1; 5)

Figure B Plot and metrics of the TBR <70 mg/dl network

The node size in the network graph represents the number of participants with the respective intervention. The line width of the edge represents the mean of inverse variances (precision) of the treatment effect of the studies on which the direct comparison is based. The colour of the edge corresponds to the average level of the RoB estimated as the precision-weighted mean of the trials of the direct comparison.

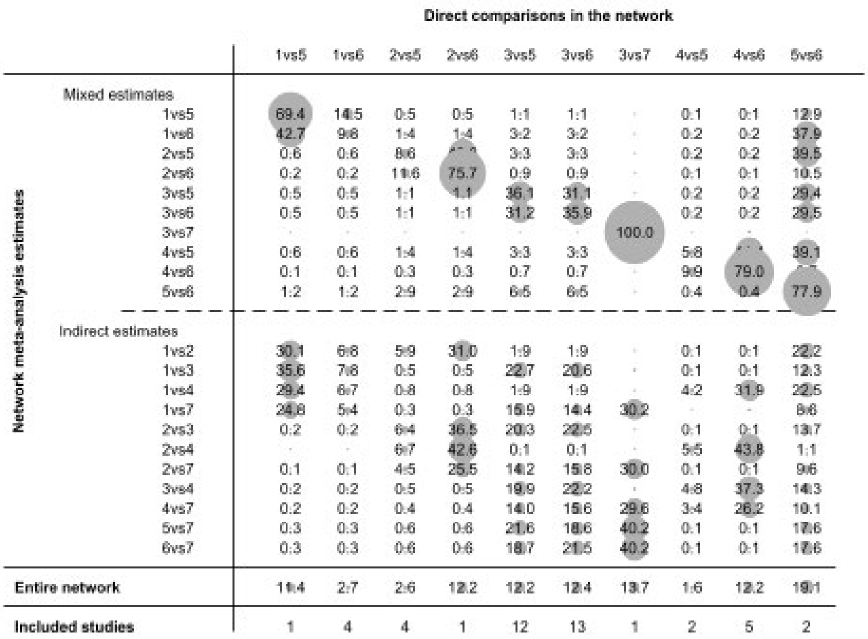

Coding	Treatment
1	MDI
2	CSII
3	SAP
4	PLGM
5	HCL
6	AHCL
7	FCL

Figure C Contribution matrix for the TBR <70 mg/dl network

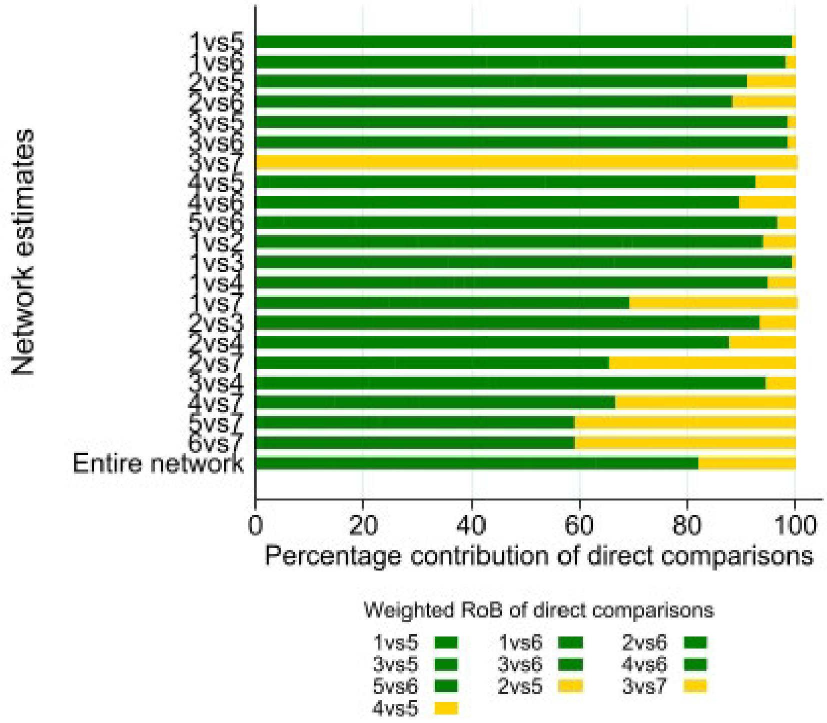

Coding	Treatment
1	MDI
2	CSII
3	SAP
4	PLGM
5	HCL
6	AHCL
7	FCL

Figure D Study limitations for each network estimate for pairwise comparisons in the TBR <70 mg/dl network

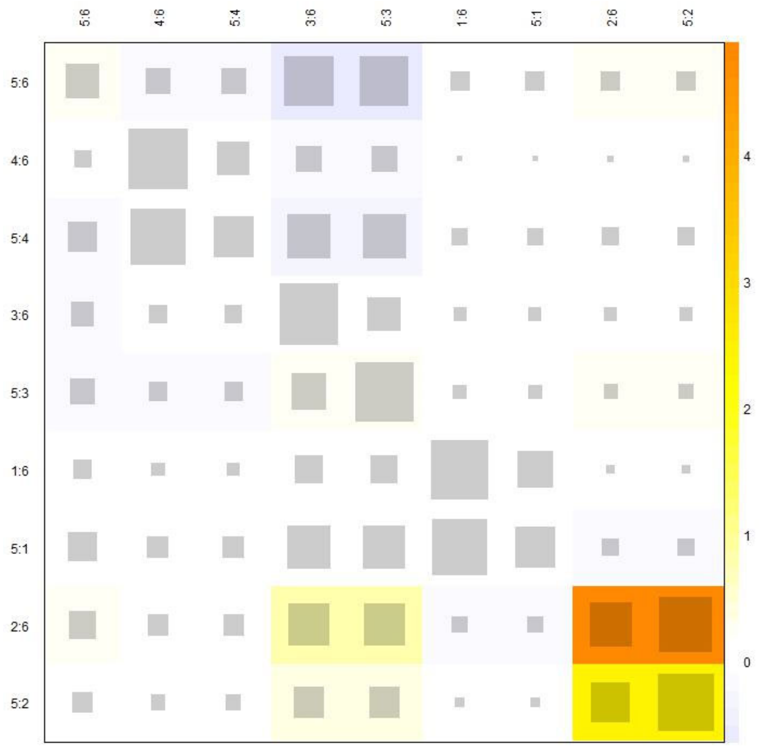

Coding	Treatment
1	MDI
2	CSII
3	SAP
4	PLGM
5	HCL
6	AHCL
7	FCL

Figure E Net heat plot

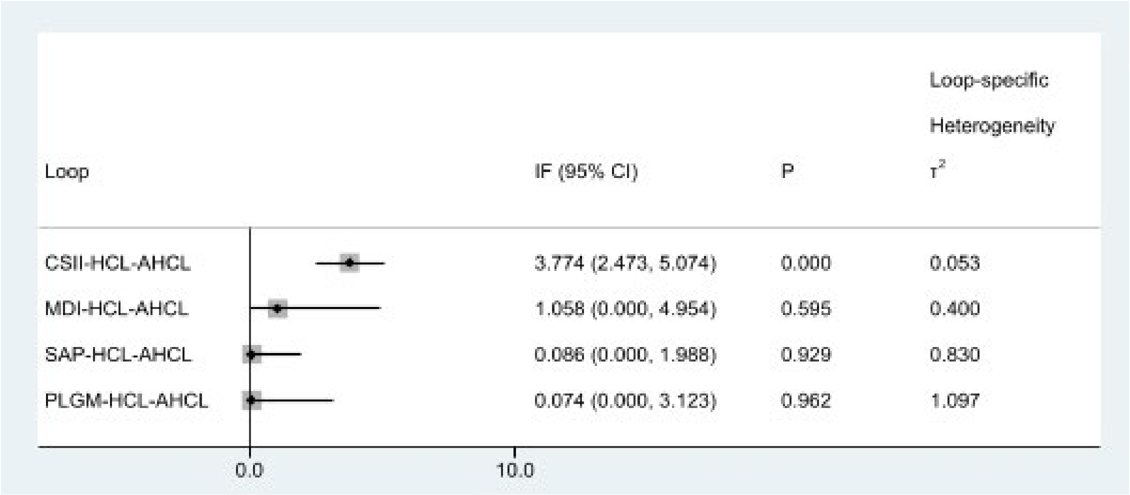

Figure F Inconsistency plot for the TBR <70 mg/dl network assuming loop-specific heterogeneity estimates

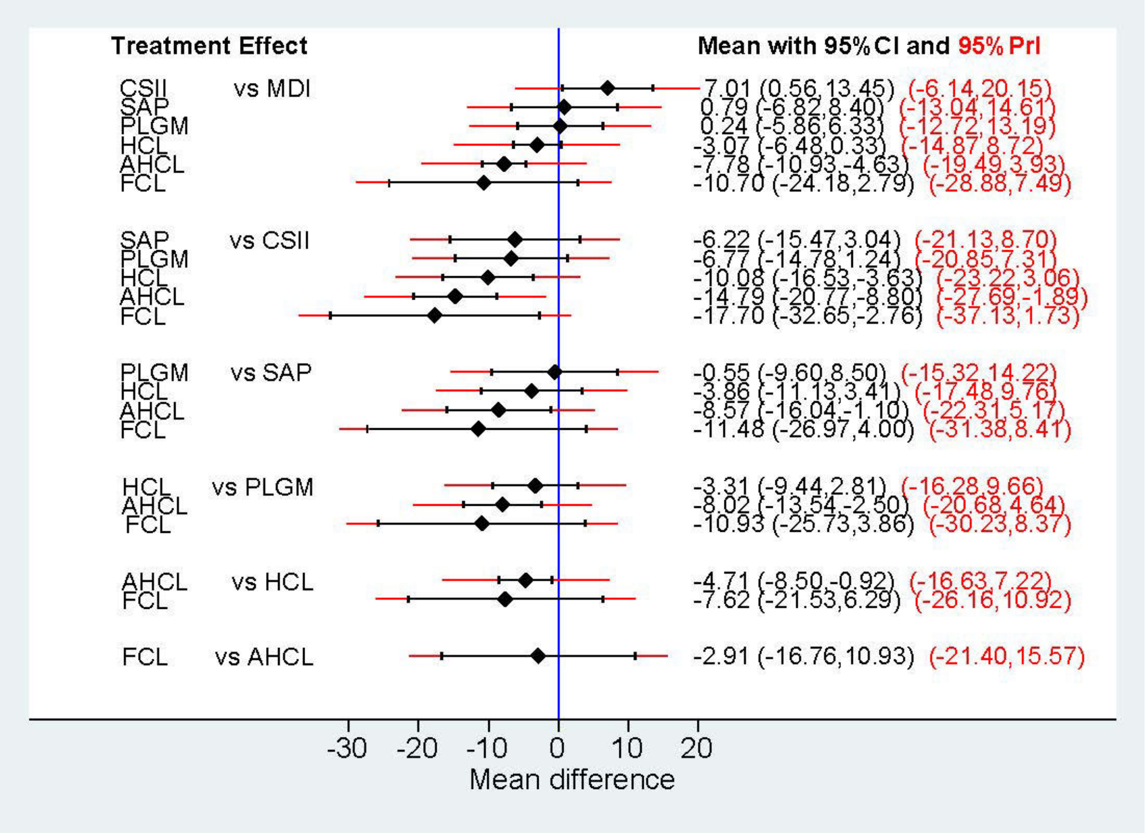

Figure G Predictive interval plot for the TBR <70 mg/dl network

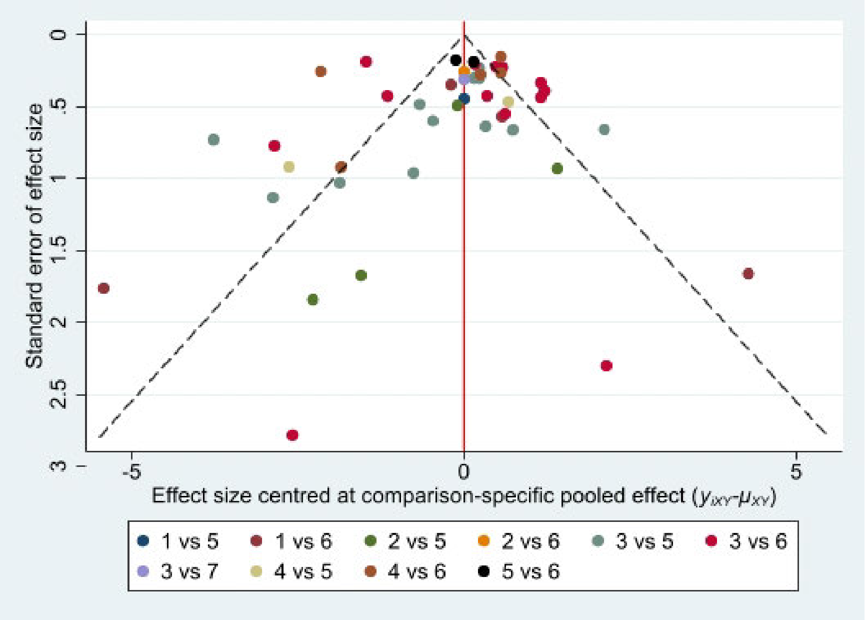

Coding	Treatment
1	MDI
2	CSII
3	SAP
4	PLGM
5	HCL
6	AHCL
7	FCL

Figure H Comparison-adjusted funnel plot for the TBR <70 mg/dl network

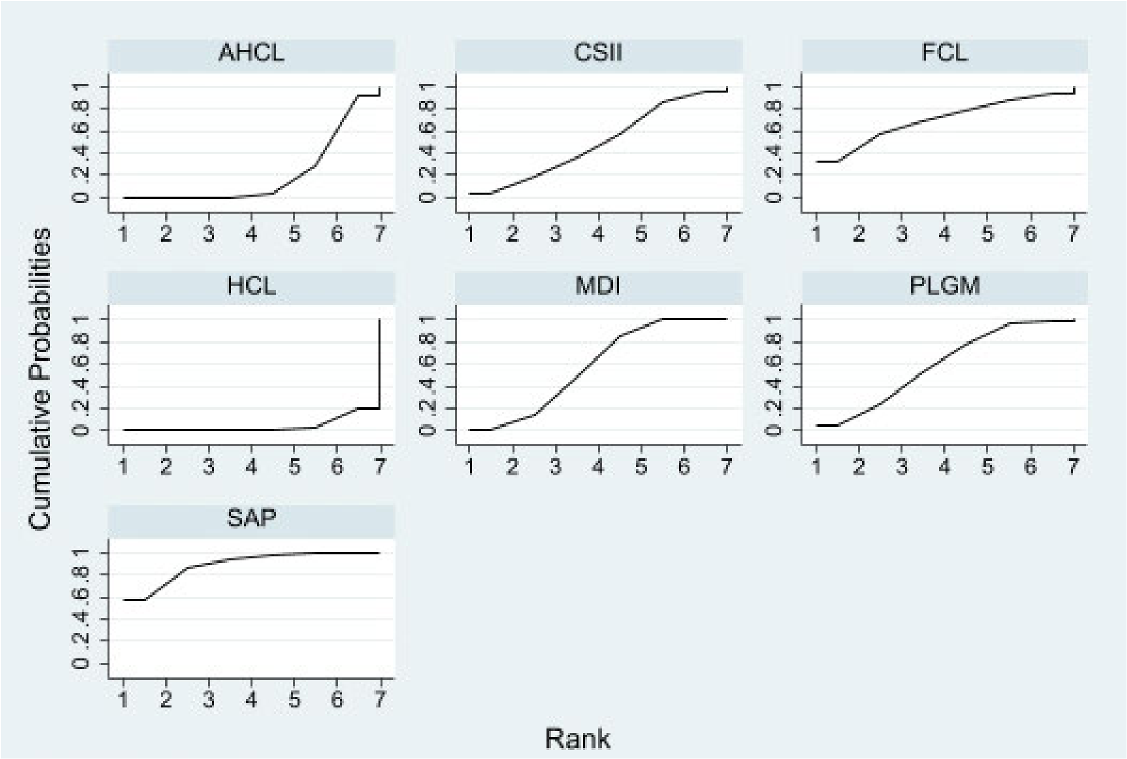

Figure I Plots of the surface under the cumulative ranking curves for all treatments in the TBR <70 mg/dl network

Table A League table of TBR <70 mg/dl network estimates

MDI	-0.22 (-1.76; 1.32)	1.17 (-0.42; 2.76)	0.03 (-1.19; 1.25)	-1.50 (-2.24; -0.77)	-0.95 (-1.66; -0.23)	0.61 (-2.08; 3.30)
0.22 (-1.32; 1.76)	CSII	1.39 (-0.68; 3.46)	0.25 (-1.52; 2.02)	-1.28 (-2.83; 0.26)	-0.73 (-2.16; 0.70)	0.83 (-2.27; 3.93)
-1.17 (-2.76; 0.42)	-1.39 (-3.46; 0.68)	SAP	-1.14 (-2.98; 0.71)	-2.67 (-4.17; -1.18)	-2.11 (-3.70; -0.53)	-0.56 (-3.68; 2.56)
-0.03 (-1.25; 1.19)	-0.25 (-2.02; 1.52)	1.14 (-0.71; 2.98)	PLGM	-1.54 (-2.77; -0.30)	-0.98 (-2.06; 0.11)	0.58 (-2.38; 3.53)
1.50 (0.77; 2.24)	1.28 (-0.26; 2.83)	2.67 (1.18; 4.17)	1.54 (0.30; 2.77)	HCL	0.56 (-0.29; 1.41)	2.11 (-0.67; 4.90)
0.95 (0.23; 1.66)	0.73 (-0.70; 2.16)	2.11 (0.53; 3.70)	0.98 (-0.11; 2.06)	-0.56 (-1.41; 0.29)	AHCL	1.55 (-1.23; 4.34)
-0.61 (-3.30; 2.08)	-0.83 (-3.93; 2.27)	0.56 (-2.56; 3.68)	-0.58 (-3.53; 2.38)	-2.11 (-4.90; 0.67)	-1.55 (-4.34; 1.23)	FCL

Table B Direct estimates, indirect estimates, and differences between direct and indirect estimates of the outcome TBR <70 mg/dl

Side	Direct		Indirect		Difference			
	Estimate	SE	Estimate	SE	Estimate	SE	p	tau
HCL vs. MDI	-1.71	1.43	-1.10	0.97	-0.61	1.73	0.723	1.36
HCL vs. CSII	-3.70	0.83	0.12	1.31	-3.82	1.55	0.014	1.22
HCL vs. SAP	-1.36	0.44	-1.97	0.76	0.61	0.88	0.484	1.35
HCL vs. PLGM	-1.21	1.08	-1.71	0.79	0.50	1.34	0.709	1.36
AHCL vs. MDI	-0.58	0.85	-1.19	1.50	0.61	1.73	0.723	1.36
AHCL vs. CSII	0.40	1.24	-3.42	0.93	3.82	1.55	0.014	1.22
AHCL vs. SAP	-1.08	0.42	-0.47	0.77	-0.61	0.88	0.484	1.35
AHCL vs. PLGM	-1.09	0.64	-0.59	1.18	-0.50	1.34	0.709	1.36
AHCL vs. HCL	0.13	0.97	0.67	0.49	-0.54	1.09	0.621	1.36

Table C Rankogram for the outcome TBR <70 mg/dl

Treatment	P-score	SUCRA	Mean Rank
MDI	0.9	58.1	3.5
CSII	4.0	49.7	4.0
SAP	57.9	89.4	1.6
PLGM	4.3	58.9	3.5
HCL	0.0	3.8	6.8
AHCL	0.0	20.6	5.8
FCL	32.9	69.5	2.8

9.5 Network meta-analysis TBR <54 mg/dl

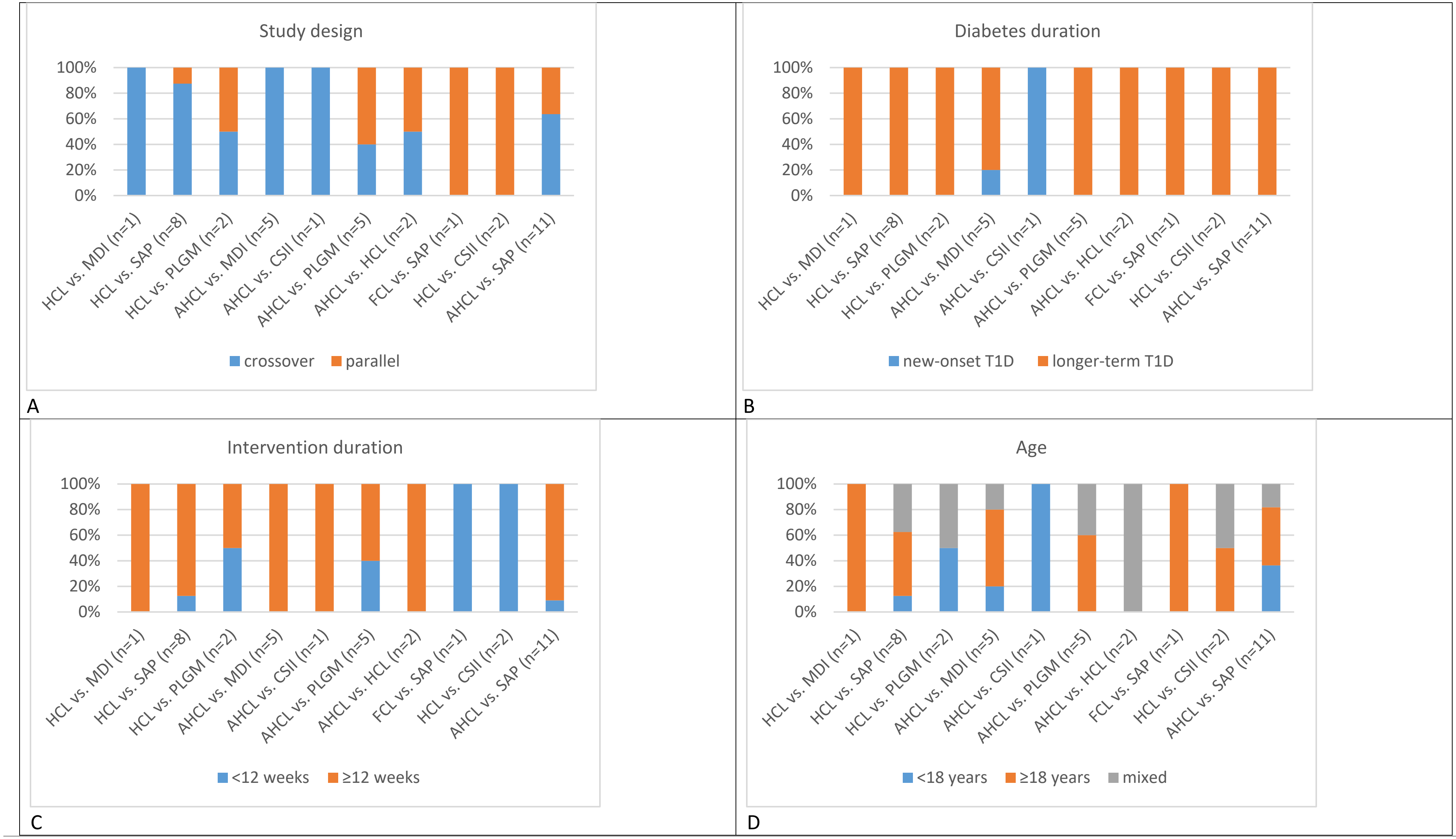

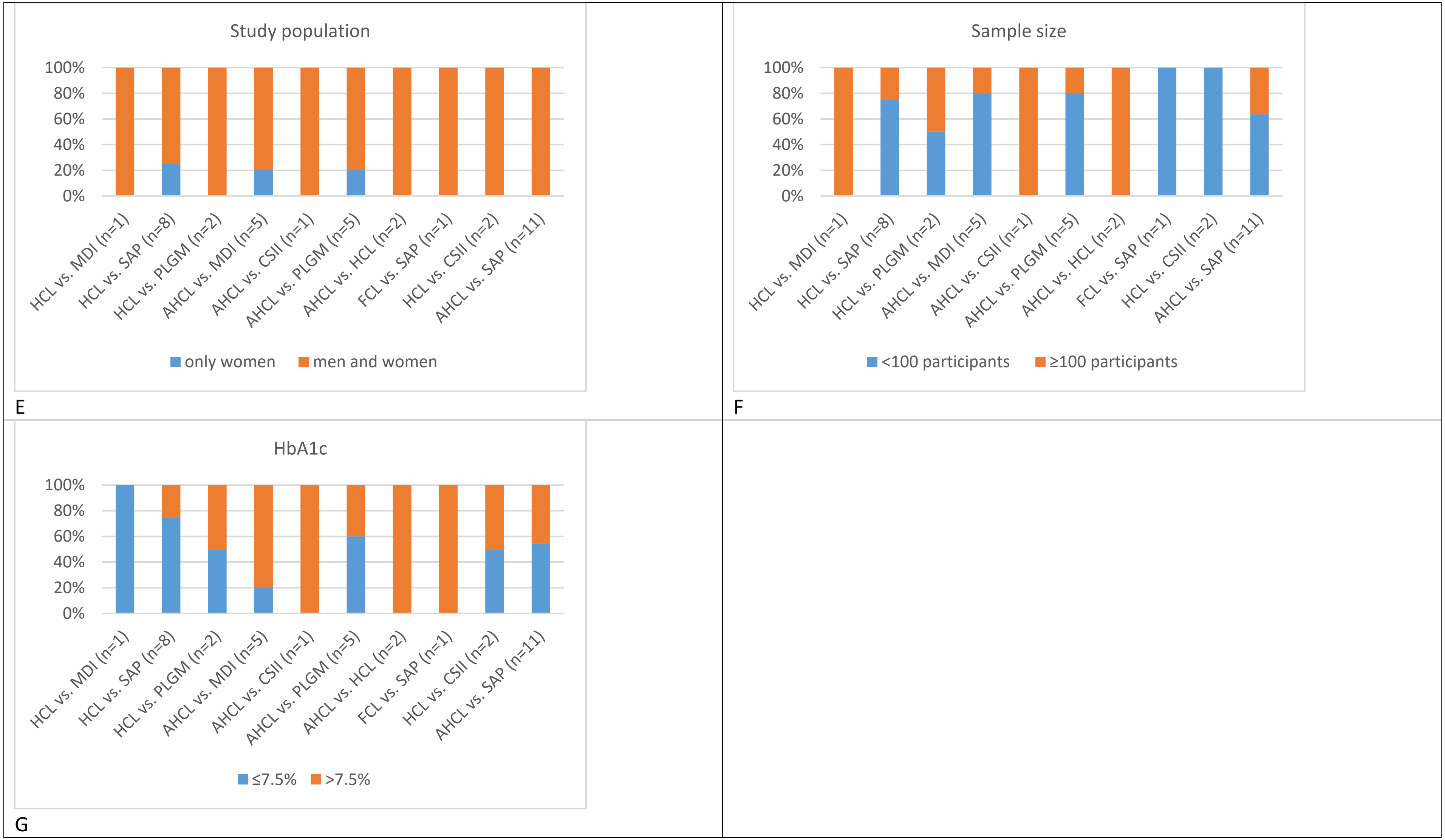

Figure A: Results of the transitivity assessment for time below range <54 mg/dl for (A) study design, (B) diabetes duration, (C) intervention duration, (D) age, (E) study population, (F) sample size, and (G) baseline HbA1c.

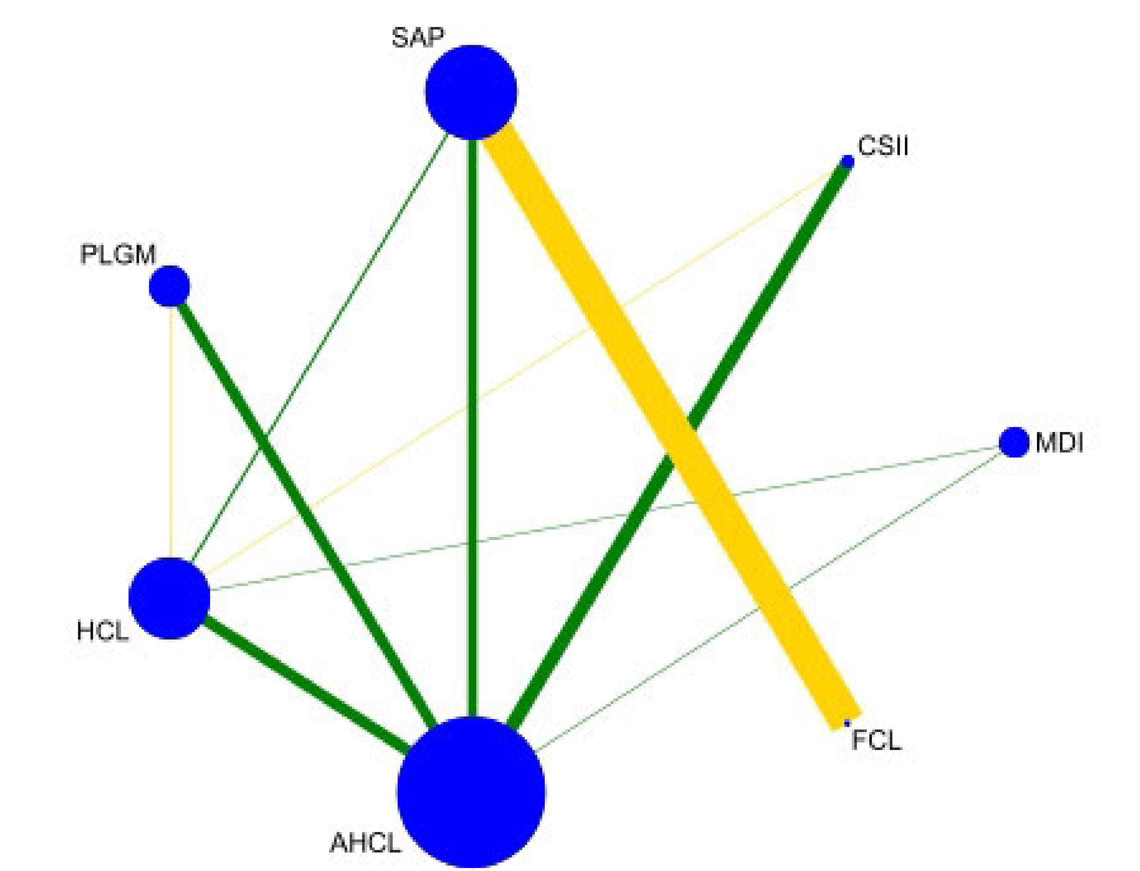

Number of studies	38
Number of treatments	7
Number of comparisons with direct evidence	10
Number of comparisons with indirect evidence	11
Density	0.5
Percentage of common comparators	85.7
Percentages of strong edges	70
Median thickness (IQR25; IQR75)	2 (1; 5)

Figure B Plot and metrics of the TBR <54 mg/dl network

The node size in the network graph represents the number of participants with the respective intervention. The line width of the edge represents the mean of inverse variances (precision) of the treatment effect of the studies on which the direct comparison is based. The colour of the edge corresponds to the average level of the RoB estimated as the precision-weighted mean of the trials of the direct comparison.

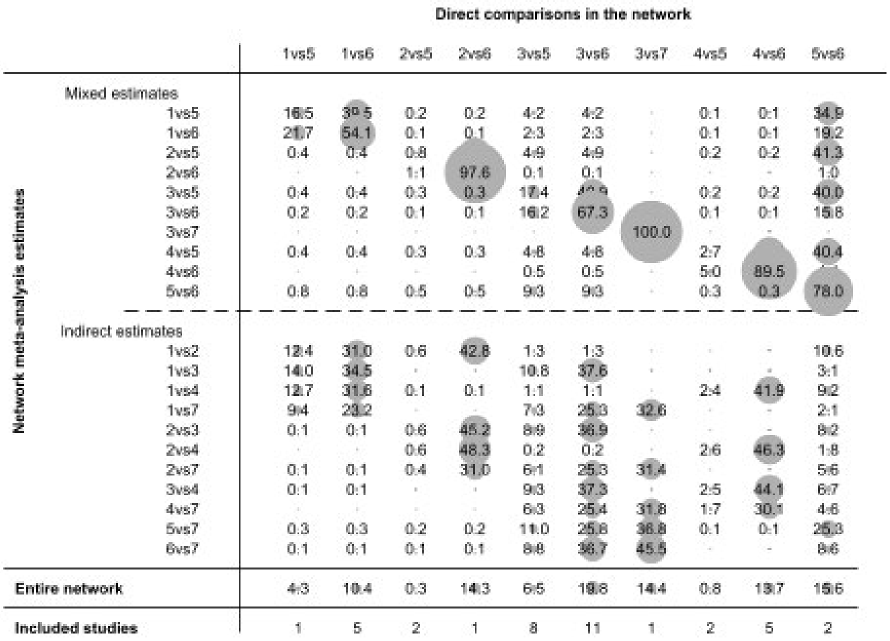

Coding	Treatment
1	MDI
2	CSII
3	SAP
4	PLGM
5	HCL
6	AHCL
7	FCL

Figure C Contribution matrix for the TBR <54 mg/dl network

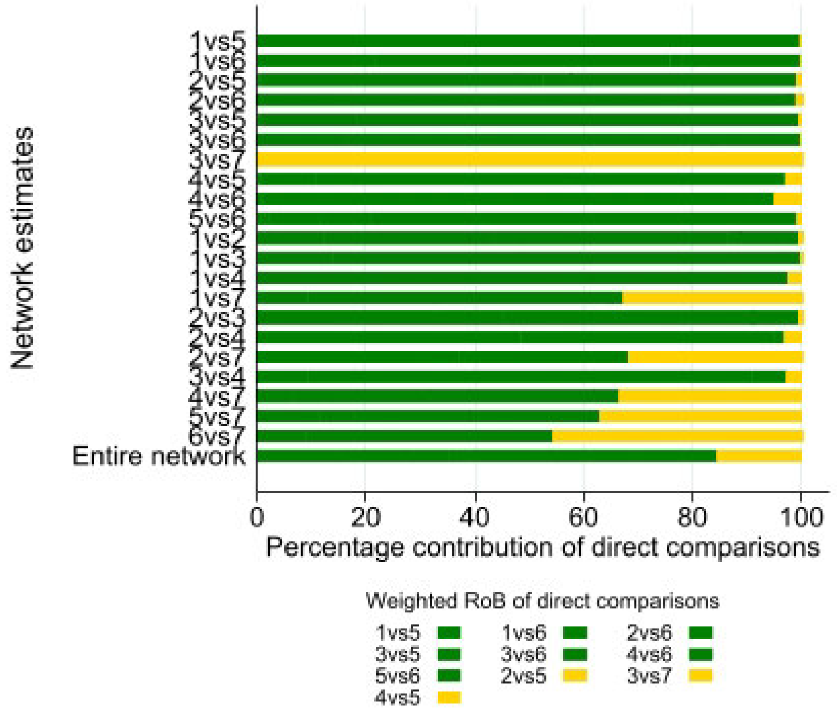

Coding	Treatment
1	MDI
2	CSII
3	SAP
4	PLGM
5	HCL
6	AHCL
7	FCL

Figure D Study limitations for each network estimate for pairwise comparisons in the TBR <54 mg/dl network

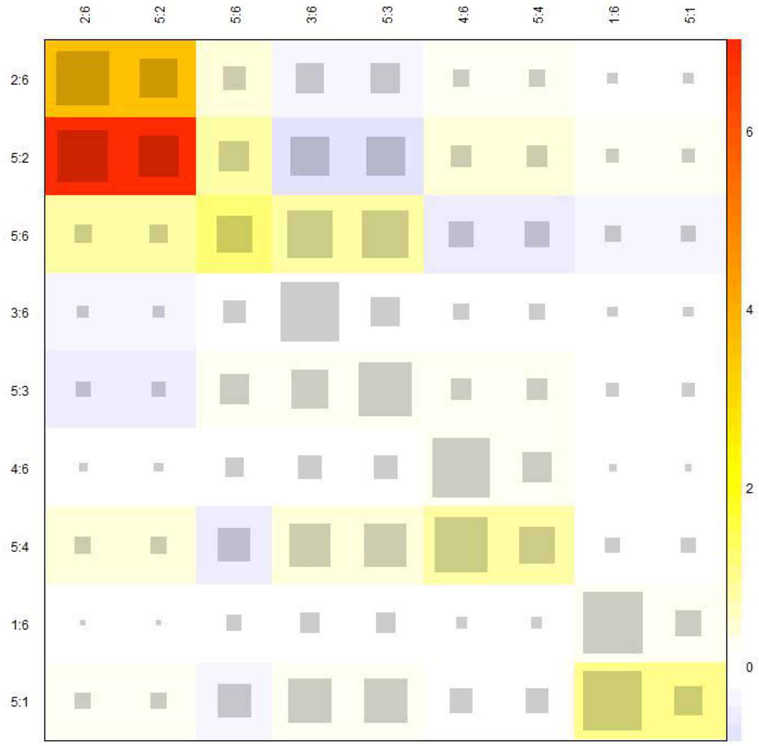

Coding	Treatment
1	MDI
2	CSII
3	SAP
4	PLGM
5	HCL
6	AHCL
7	FCL

Figure E Net heat plot

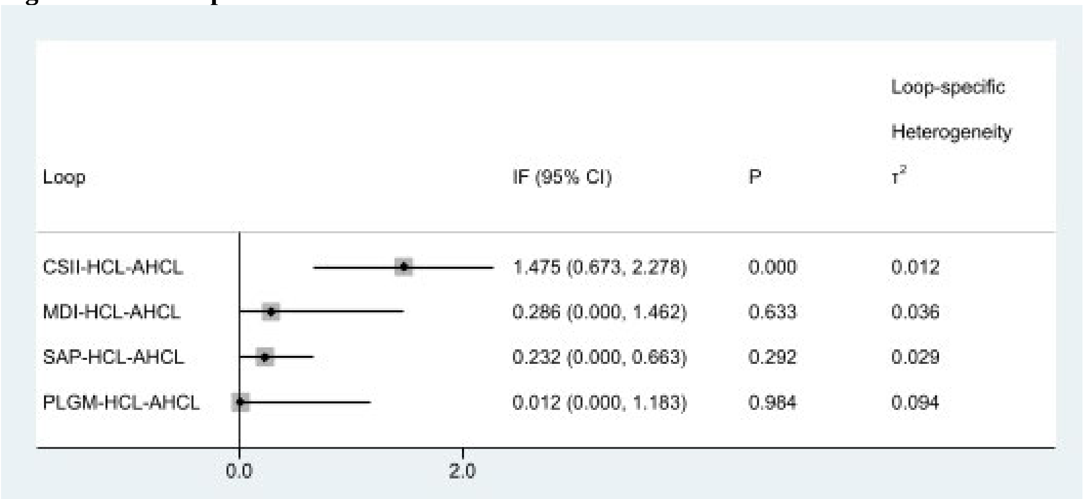

Figure F Inconsistency plot for the TBR <54 mg/dl network assuming loop-specific heterogeneity estimates

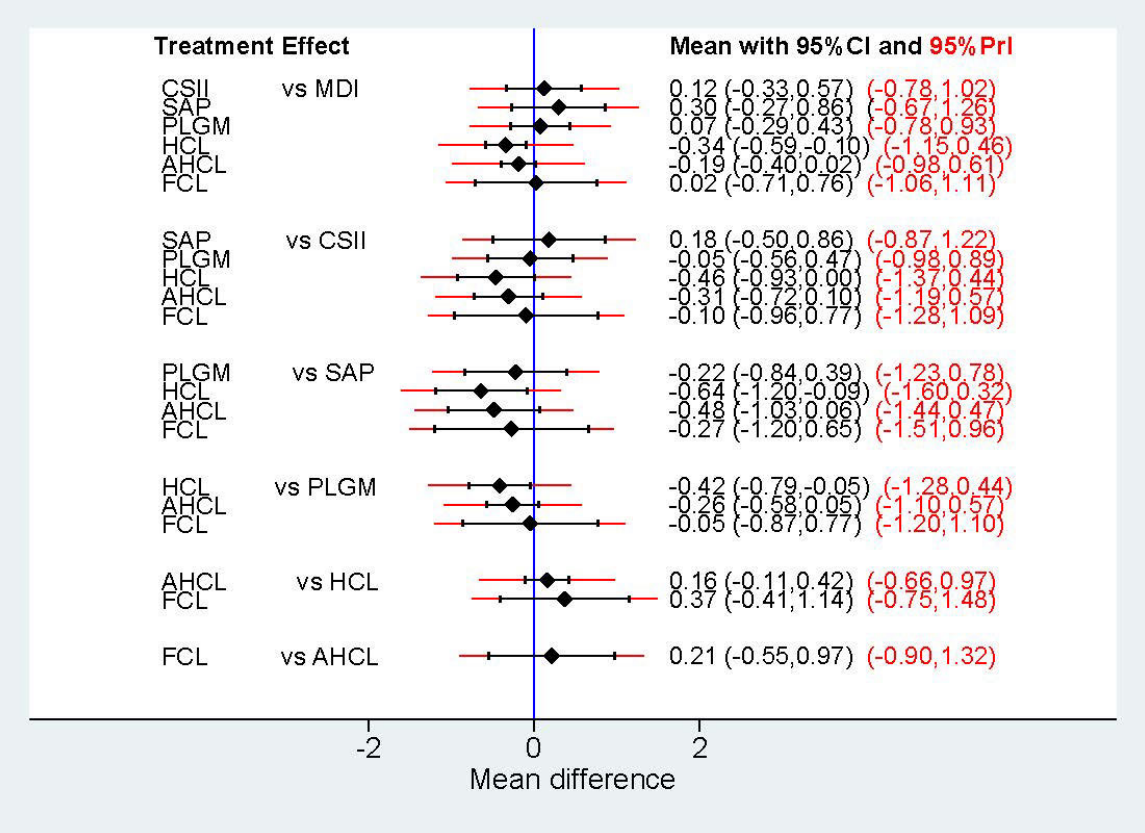

Figure G Predictive interval plot for the TBR <54 mg/dl network

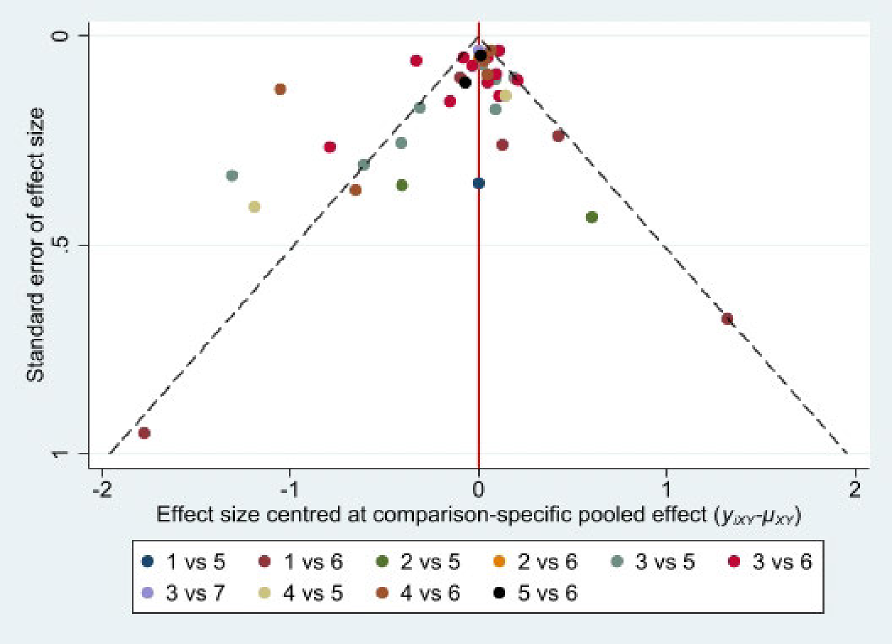

Coding	Treatment
1	MDI
2	CSII
3	SAP
4	PLGM
5	HCL
6	AHCL
7	FCL

Figure H Comparison-adjusted funnel plot for the TBR <54 mg/dl network

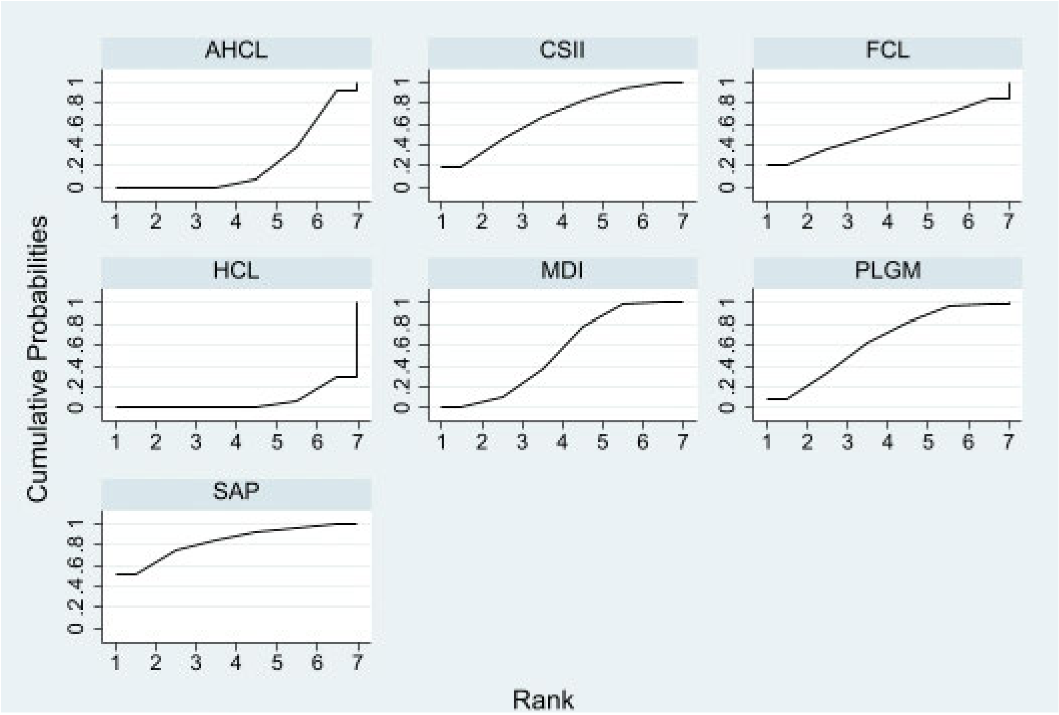

Figure I Plots of the surface under the cumulative ranking curves for all treatments in the TBR <54 mg/dl network

Table A League table of TBR <54 mg/dl network estimates

MDI	0.12 (-0.33; 0.57)	0.30 (-0.27; 0.86)	0.07 (-0.29; 0.43)	-0.34 (-0.59; -0.10)	-0.19 (-0.40; 0.02)	0.02 (-0.71; 0.76)
-0.12 (-0.57; 0.33)	CSII	0.18 (-0.50; 0.86)	-0.05 (-0.56; 0.47)	-0.46 (-0.93; -0.00)	-0.31 (-0.72; 0.10)	-0.10 (-0.96; 0.77)
-0.30 (-0.86; 0.27)	-0.18 (-0.86; 0.50)	SAP	-0.22 (-0.84; 0.39)	-0.64 (-1.20; -0.09)	-0.48 (-1.03; 0.06)	-0.27 (-1.20; 0.65)
-0.07 (-0.43; 0.29)	0.05 (-0.47; 0.56)	0.22 (-0.39; 0.84)	PLGM	-0.42 (-0.79; -0.05)	-0.26 (-0.58; 0.05)	-0.05 (-0.87; 0.77)
0.34 (0.10; 0.59)	0.46 (0.00; 0.93)	0.64 (0.09; 1.20)	0.42 (0.05; 0.79)	HCL	0.16 (-0.11; 0.42)	0.37 (-0.41; 1.14)
0.19 (-0.02; 0.40)	0.31 (-0.10; 0.72)	0.48 (-0.06; 1.03)	0.26 (-0.05; 0.58)	-0.16 (-0.42; 0.11)	AHCL	0.21 (-0.55; 0.97)
-0.02 (-0.76; 0.71)	0.10 (-0.77; 0.96)	0.27 (-0.65; 1.20)	0.05 (-0.77; 0.87)	-0.37 (-1.14; 0.41)	-0.21 (-0.97; 0.55)	FCL

Table B Direct estimates, indirect estimates, and differences between direct and indirect estimates of the outcome TBR <54 mg/dl

Side	Direct		Indirect		Difference			
	Estimate	SE	Estimate	SE	Estimate	SE	p	tau
HCL vs. MDI	-0.06	0.51	-0.57	0.27	0.51	0.58	0.379	0.38
HCL vs. CSII	-1.26	0.36	0.04	0.35	-1.31	0.50	0.010	0.33
HCL vs. SAP	-0.39	0.15	-0.24	0.23	-0.15	0.28	0.574	0.38
HCL vs. PLGM	-0.26	0.33	-0.50	0.23	0.24	0.40	0.546	0.38
AHCL vs. MDI	-0.39	0.23	0.12	0.53	-0.51	0.58	0.379	0.38
AHCL vs. CSII	0.11	0.33	-1.20	0.38	1.31	0.50	0.010	0.33
AHCL vs. SAP	-0.16	0.12	-0.31	0.25	0.15	0.27	0.574	0.38
AHCL vs. PLGM	-0.31	0.18	-0.07	0.36	-0.24	0.40	0.546	0.38
AHCL vs. HCL	-0.08	0.27	0.23	0.16	-0.31	0.31	0.317	0.38

Table C Rankogram for the outcome TBR <54 mg/dl

Treatment	P-score	SUCRA	Mean Rank
MDI	1.1	54.0	3.8
CSII	18.4	68.1	2.9
SAP	51.5	82.9	2.0
PLGM	9.0	63.2	3.2
HCL	0.0	5.9	6.6
AHCL	0.0	22.9	5.6
FCL	20.0	53.0	3.8

9.6 Network meta-analysis HbA1c

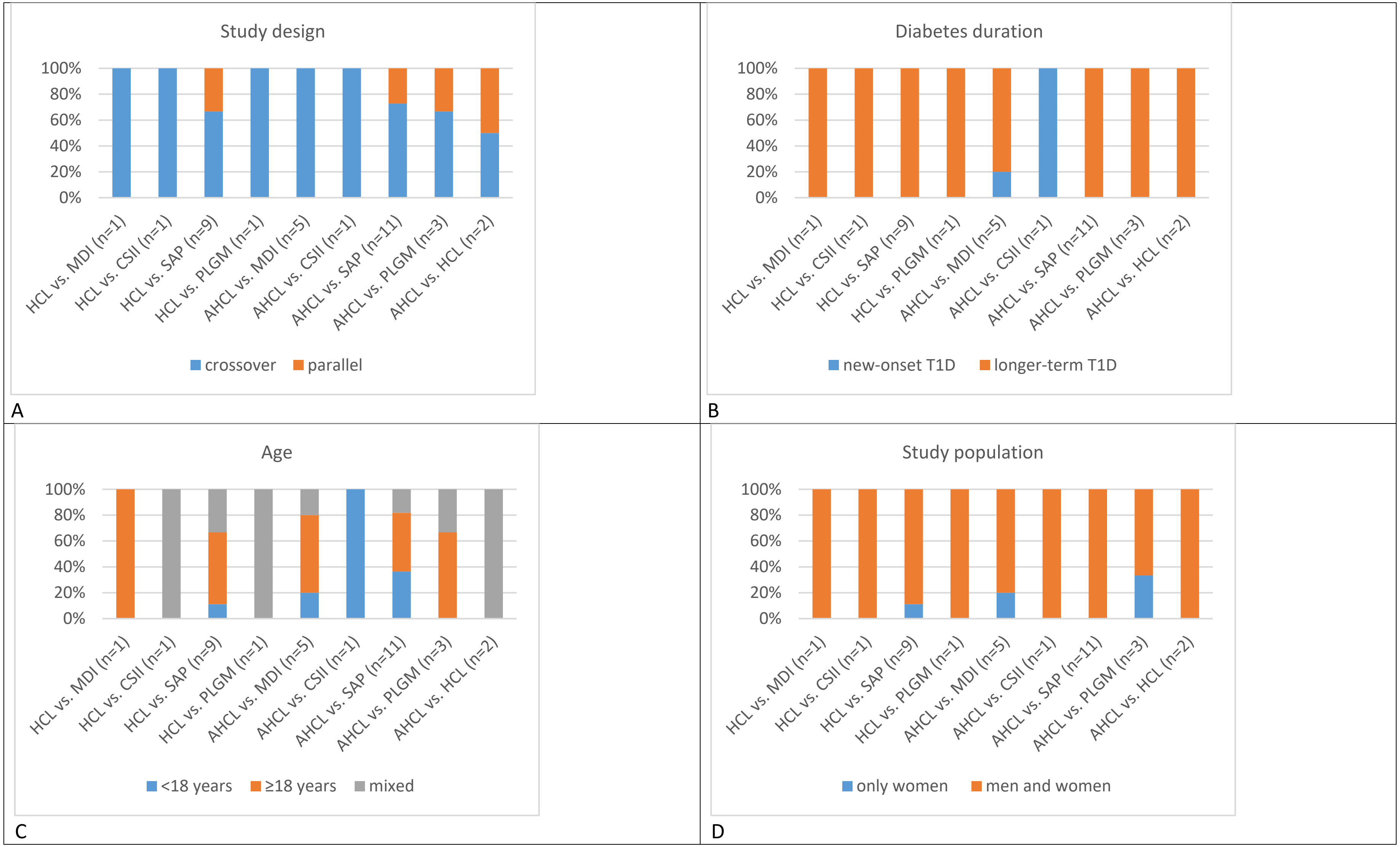

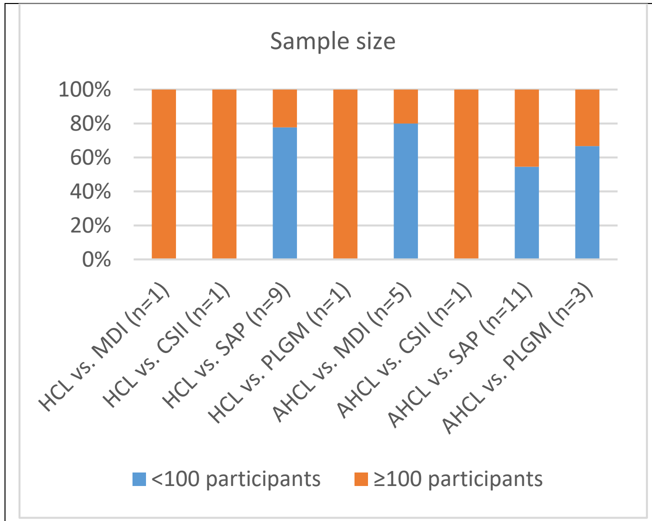

E

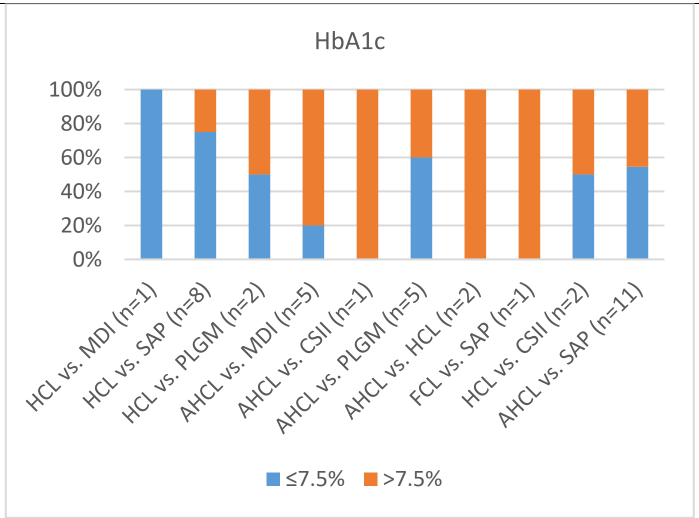

F

Figure A: Results of the transitivity assessment for HbA1c for (A) study design, (B) diabetes duration, (C) age, (D) study population, (E) sample size, and (F) baseline HbA1c.

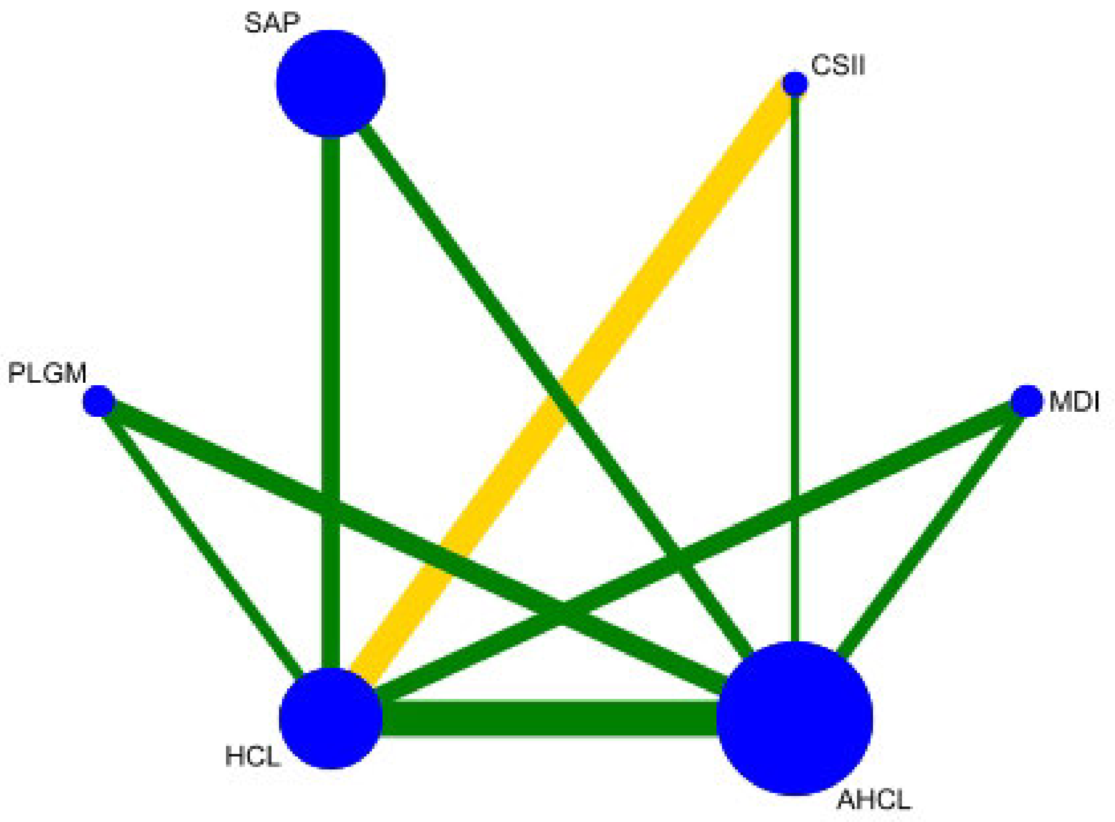

Number of studies	34
Number of treatments	6
Number of comparisons with direct evidence	9
Number of comparisons with indirect evidence	6
Density	0,6
Percentage of common comparators	100
Percentages of strong edges	55.6
Median thickness (IQR25; IQR75)	1 (1; 2.5)

Figure B Plot and metrics of the HbA1c network

The node size in the network graph represents the number of participants with the respective intervention. The line width of the edge represents the mean of inverse variances (precision) of the treatment effect of the studies on which the direct comparison is based. The colour of the edge corresponds to the average level of the RoB estimated as the precision-weighted mean of the trials of the direct comparison.

	Direct comparisons in the network								
	1vs5	1vs6	2vs5	2vs6	3vs5	3vs6	4vs5	4vs6	5vs6
**Network meta-analysis estimates — Mixed estimates**									
1vs5	72.4	11.4	1.2	1.2	2.3	2.3	1.4	1.4	6.6
1vs6	37.9	8.2	3.9	3.9	7.6	7.6	4.5	4.5	21.9
2vs5	0.7	0.7	63.8	15.2	3.2	3.2	1.9	1.9	9.3
2vs6	1.7	1.7	35.7	14.7	7.6	7.6	4.5	4.5	21.9
3vs5	1.7	1.7	3.9	3.9	28.5	31.7	4.5	4.5	21.7
3vs6	1.0	1.0	2.4	2.4	19.6	54.6	2.8	2.8	13.4
4vs5	1.5	1.5	3.6	3.6	6.9	6.9	24.1	32.0	19.9
4vs6	1.1	1.1	2.5	2.5	4.7	4.7	22.0	47.7	13.7
5vs6	2.9	2.9	6.9	6.9	13.2	13.2	7.9	7.9	38.2
**Indirect estimates**									
1vs2	41.0	6.9	38.1	9.9	0.7	0.7	0.4	0.4	1.9
1vs3	33.1	6.5	2.1	2.1	17.0	22.7	2.4	2.4	11.7
1vs4	32.6	6.3	1.9	1.9	3.7	3.7	16.1	22.8	10.8
2vs3	0.8	0.8	30.9	10.8	17.3	24.4	2.2	2.2	10.6
2vs4	0.7	0.7	30.5	10.5	3.4	3.4	16.6	24.4	9.8
3vs4	0.1	0.1	0.1	0.1	16.4	33.0	17.4	32.0	0.8
**Entire network**	15.6	3.5	15.1	6.0	10.0	14.3	8.8	12.9	13.7
**Included studies**	1	5	1	1	9	11	1	3	2

Coding	Treatment
1	MDI
2	CSII
3	SAP
4	PLGM
5	HCL
6	AHCL
7	FCL

Figure C Contribution matrix for the HbA1c network

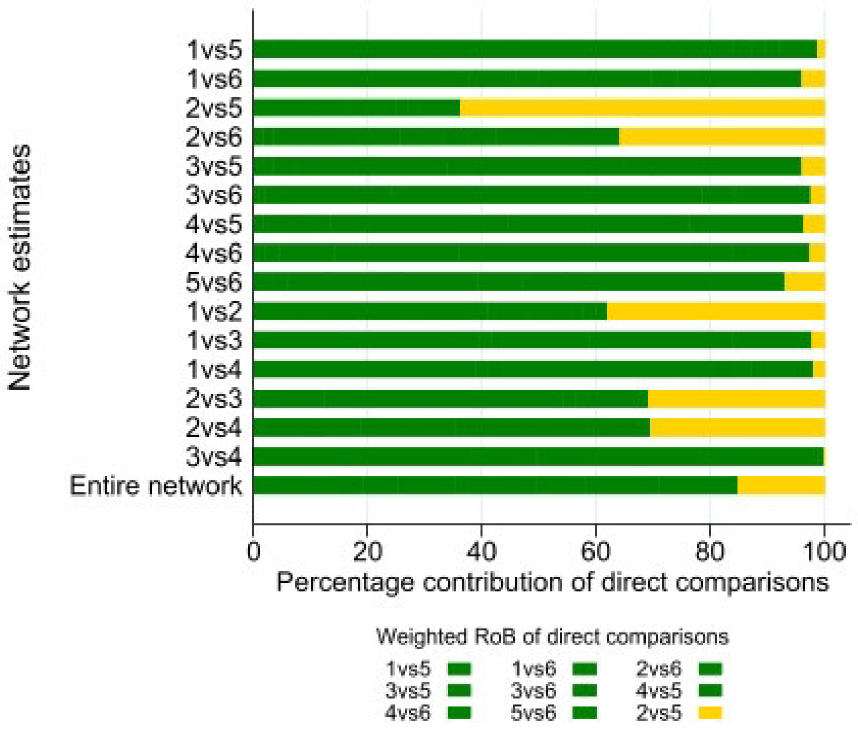

Coding	Treatment
1	MDI
2	CSII
3	SAP
4	PLGM
5	HCL
6	AHCL
7	FCL

Figure D Study limitations for each network estimate for pairwise comparisons in the HbA1c network

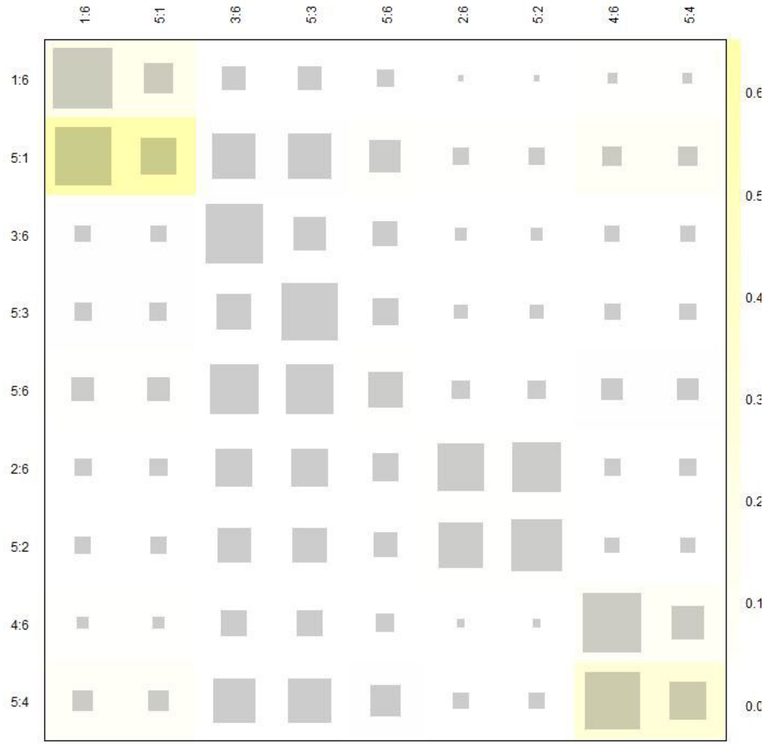

Coding	Treatment
1	MDI
2	CSII
3	SAP
4	PLGM
5	HCL
6	AHCL
7	FCL

Figure E Net heat plot

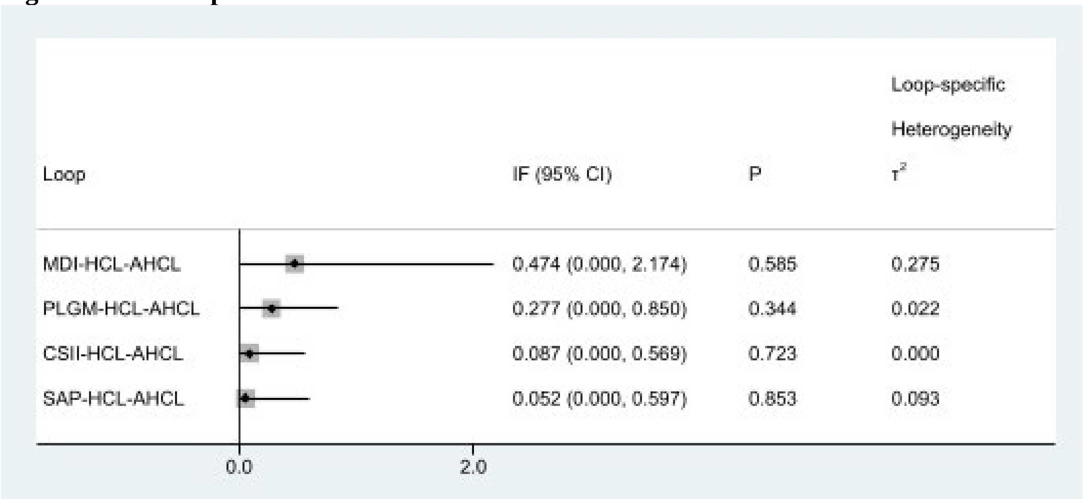

Figure F Inconsistency plot for the HbA1c network assuming loop-specific heterogeneity estimates

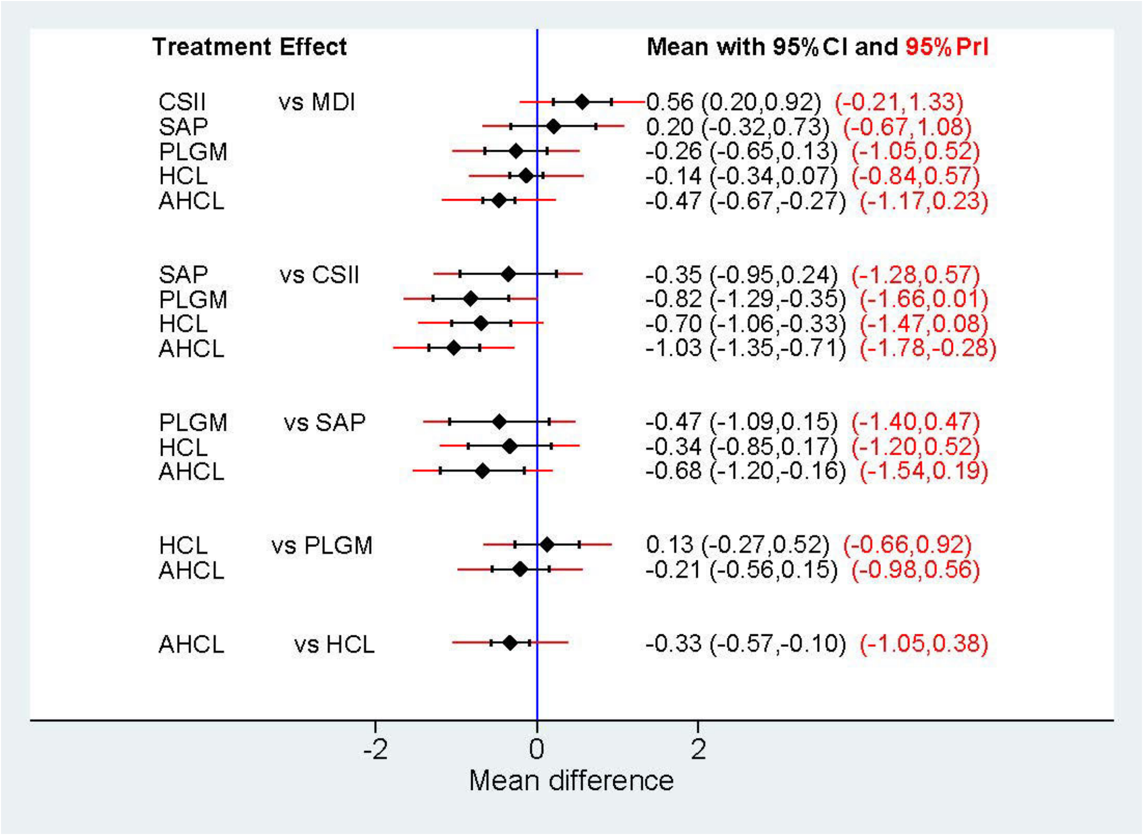

Figure G Predictive interval plot for the HbA1c network

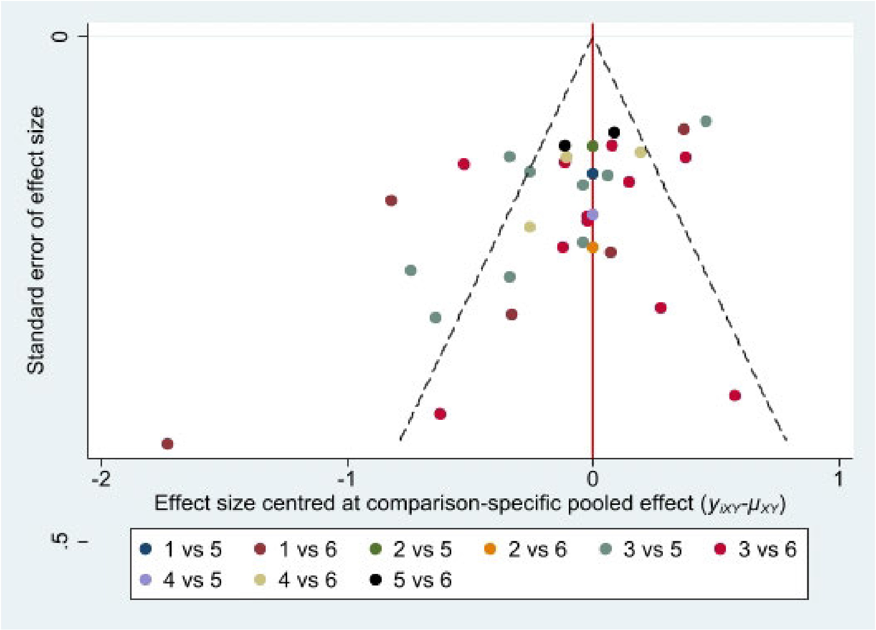

Coding	Treatment
1	MDI
2	CSII
3	SAP
4	PLGM
5	HCL
6	AHCL
7	FCL

Figure H Comparison-adjusted funnel plot for the HbA1c network

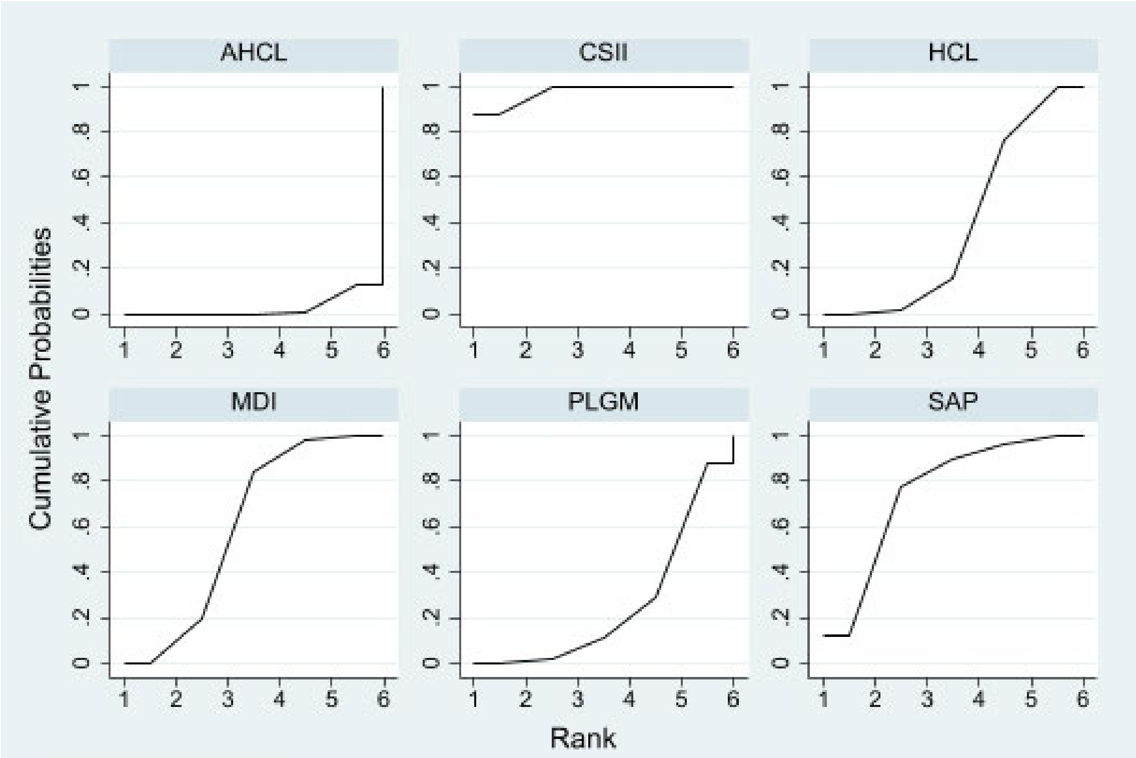

Figure I Plots of the surface under the cumulative ranking curves for all treatments in the HbA1c network

Table A League table of HbA1c network estimates

MDI	0.56 (0.20; 0.92)	0.20 (-0.32; 0.73)	-0.26 (-0.65; 0.13)	-0.14 (-0.34; 0.07)	-0.47 (-0.67; -0.27)
-0.56 (-0.92; -0.20)	CSII	-0.35 (-0.95; 0.24)	-0.82 (-1.29; -0.35)	-0.70 (-1.06; -0.33)	-1.03 (-1.35; -0.71)
-0.20 (-0.73; 0.32)	0.35 (-0.24; 0.95)	SAP	-0.47 (-1.09; 0.15)	-0.34 (-0.85; 0.17)	-0.68 (-1.20; -0.16)
0.26 (-0.13; 0.65)	0.82 (0.35; 1.29)	0.47 (-0.15; 1.09)	PLGM	0.13 (-0.27; 0.52)	-0.21 (-0.56; 0.15)
0.14 (-0.07; 0.34)	0.70 (0.33; 1.06)	0.34 (-0.17; 0.85)	-0.13 (-0.52; 0.27)	HCL	-0.33 (-0.57; -0.10)
0.47 (0.27; 0.67)	1.03 (0.71; 1.35)	0.68 (0.16; 1.20)	0.21 (-0.15; 0.56)	0.33 (0.10; 0.57)	AHCL

Table B Direct estimates, indirect estimates, network estimates and differences between direct and indirect estimates of the outcome HbA1c

Side	Direct		Indirect		Difference			
	Estimate	SE	Estimate	SE	Estimate	SE	p	tau
HCL vs. MDI	-0.40	0.36	-0.81	0.22	0.41	0.42	0.329	0.33
HCL vs. CSII	-0.40	0.35	-0.26	0.41	-0.14	0.54	0.792	0.33
HCL vs. SAP	-0.13	0.13	-0.15	0.21	0.02	0.25	0.931	0.33
HCL vs. PLGM*	-0.10	0.37	0.22	0.24	-0.32	0.44	0.468	0.33
AHCL vs. MDI	-1.11	0.18	-0.70	0.38	-0.41	0.42	0.329	0.33
AHCL vs. CSII	-0.60	0.39	-0.74	0.37	0.14	0.54	0.792	0.33
AHCL vs. SAP	-0.48	0.12	-0.45	0.22	-0.02	0.25	0.931	0.33
AHCL vs. PLGM	-0.14	0.21	-0.46	0.39	0.32	0.44	0.468	0.33
AHCL vs. HCL	-0.30	0.25	-0.35	0.14	0.05	0.29	0.867	0.33

Table C Rankogram for the outcome HbA1c

Treatment	P-score	SUCRA	Mean Rank
MDI	0.1	60.4	3.0
CSII	88.0	97.6	1.1
SAP	11.9	74.9	2.3
PLGM	0.0	25.9	4.7
HCL	0.0	38.6	4.1
AHCL	0.0	2.7	5.9
FCL	-	-	-

9.7 Global consistencies

Outcome	Global consistency	
	**Chi square**	**P value**
TIR	1.58	0.8121
TAR >180 mg/dl	0.32	0.9883
TAR >250 mg/dl	2.95	0.5655
TBR <70 mg/dl	5.81	0.2138
TBR <54 mg/dl	8.23	0.0835
HbA1c	1.32	0.8575

10 Supplement to GRADE CoE assessment from network meta-analysis

10.1 Applying GRADE to network meta-analysis

The GRADE rating of direct estimates to inform the rating of NMA estimates included RoB, inconsistency, indirectness, and publication bias. The starting point for the assessment of indirect estimates was the lowest of the preliminary certainty ratings for the direct comparisons that constitute the most dominant loop in terms of the number of studies and participants included. Serious intransitivity led to down rating by one level. The imprecision assessment of direct and indirect estimates was performed when direct and indirect were incoherent.

The certainty of network estimates was based on the respective certainty rating for the dominating direct or indirect estimate, and rated down if incoherence or imprecision was present.[7,53] All GRADE assess reasons for rating down are given in Appendix 1.4. When direct and indirect estimate were coherent, the NMA estimate was chosen as the best estimate. Otherwise, the estimate with the highest certainty was used.

10.2 CoE assessment TIR network meta-analysis

**Comparison**		**Direct estimate TIR**			**GRADE**						
Intervention	Control	Point estimate	CI lower limit	CI upper limit	Risk of bias	Inconsistency	Indirectness	Publication bias	**Rating for NMA**	Imprecision	**Final rating**
HCL	MDI	15.2	11.0	19.4	Not serious	Not serious	Serious	Not serious	MODERATE	Serious	LOW
HCL	CSII	12.0	9.7	14.2	Serious	Not serious	Not serious	Not serious	MODERATE	Serious	LOW
HCL	SAP	8.8	14.7	13.0	Not serious	Very serious	Not serious	Serious	VERY LOW	Very serious	VERY LOW
HCL	PLGM	8.2	4.2	12.2	Serious	Not serious	Not serious	Not serious	MODERATE	Very serious	VERY LOW
AHCL	MDI	25.3	17.8	32.8	Not serious	Serious	Not serious	Not serious	MODERATE	Serious	LOW
AHCL	CSII	14.0	7.6	20.1	Not serious	Not serious	Serious	Not serious	MODERATE	Serious	LOW
AHCL	SAP	11.4	9.2	13.6	Not serious	Serious	Not serious	Serious	LOW	Not serious	LOW
AHCL	PLGM	12.1	0.1	24.0	Not serious	Serious	Not serious	Not serious	MODERATE	Very serious	VERY LOW
AHCL	HCL	7.4	0.6	14.3	Not serious	Serious	Not serious	Not serious	MODERATE	Very serious	VERY LOW
FCL	MDI										
FCL	CSII										
FCL	SAP	13.8	7.7	19.9	Serious	Not serious	Not serious	Not serious	MODERATE	Serious	LOW
FCL	PLGM										
FCL	HCL										
FCL	AHCL										

**Comparison**		**Indirect estimate TIR**			**GRADE**				
Intervention	Control	Point estimate	CI lower limit	CI upper limit	Start rating	Intransitivity	**Rating for NMA**	Imprecision	**Final rating**
HCL	MDI	21.5	13.8	29.1	MODERATE	Serious	LOW	Not Serious	LOW
HCL	CSII	9.4	-4.6	23.4	MODERATE	Serious	LOW	Not Serious	LOW
HCL	SAP	5.7	-0.8	12.2	LOW	Serious	VERY LOW	Serious	VERY LOW
HCL	PLGM	7.9	1.1	14.7	MODERATE	Serious	LOW	Serious	VERY LOW
AHCL	MDI	19.1	6.3	31.9	MODERATE	Serious	LOW	Not Serious	LOW
AHCL	CSII	17.6	9.9	25.2	MODERATE	Serious	LOW	Not Serious	LOW
AHCL	SAP	14.7	8.1	21.4	VERY LOW	Serious	VERY LOW	Not Serious	VERY LOW
AHCL	PLGM	12.8	3.1	22.5	MODERATE	Serious	LOW	Not Serious	LOW
AHCL	HCL	3.6	-0.6	7.8	VERY LOW	Serious	VERY LOW	Serious	VERY LOW
FCL	MDI								
FCL	CSII								
FCL	SAP								
FCL	PLGM								
FCL	HCL								
FCL	AHCL								

Comparison		Network estimate TIR			GRADE					
Intervention	Control	Point estimate	CrI lower limit	CrI upper limit	Starting evidence	**Starting rating**	Incoherence	Imprecision	**Final rating**	**Best estimate**
HCL	MDI	8.0	4.7	11.2	Direct	MODERATE	Not serious	Serious	LOW	NMA
HCL	CSII	19.7	13.2	26.1	Direct	MODERATE	Not serious	Not Serious	MODERATE	NMA
HCL	SAP	12.3	6.4	18.2	Direct	VERY LOW	Not serious	Not Serious	VERY LOW	NMA
HCL	PLGM	8.1	2.8	13.4	Direct	MODERATE	Not serious	Serious	LOW	NMA
AHCL	MDI	12.4	9.3	15.4	Direct	MODERATE	Not serious	Not Serious	MODERATE	NMA
AHCL	CSII	24.1	18.2	29.9	Direct	MODERATE	Not serious	Not Serious	MODERATE	NMA
AHCL	SAP	16.7	10.1	23.3	Direct	LOW	Not serious	Not Serious	LOW	NMA
AHCL	PLGM	12.5	7.8	17.1	Direct	MODERATE	Not serious	Not Serious	MODERATE	NMA
AHCL	HCL	4.4	0.7	8.1	Direct	MODERATE	Not serious	Serious	LOW	NMA
FCL	MDI	13.8	0.9	26.7	Direct	HIGH	Not serious	Serious	MODERATE	NMA
FCL	CSII	25.5	11.1	39.9	Direct	HIGH	Not serious	Not Serious	HIGH	NMA
FCL	SAP	18.1	3.7	32.6	Direct	MODERATE	Not serious	Serious	LOW	NMA
FCL	PLGM	13.9	-0.1	27.9	Direct	HIGH	Not serious	Serious	MODERATE	NMA
FCL	HCL	5.8	-7.5	19.2	Direct	HIGH	Not serious	Serious	MODERATE	NMA
FCL	AHCL	1.5	-11.8	14.7	Direct	HIGH	Not serious	Serious	MODERATE	NMA

10.3 CoE assessment TAR >180 mg/dl network meta-analysis

**Comparison**		**Direct estimate TAR >180 mg/dl**			**GRADE**						
Intervention	Control	Point estimate	CI lower limit	CI upper limit	Risk of bias	Inconsistency	Indirectness	Publication bias	**Rating for NMA**	Imprecision	**Final rating**
HCL	MDI	-12.7	-17.2	-8.2	Not serious	Not serious	Serious	Not serious	MODERATE	Serious	LOW
HCL	CSII	-11.0	-17.9	-4.1	Serious	Not serious	Not serious	Not serious	MODERATE	Very serious	VERY LOW
HCL	SAP	-4.7	-2.2	-8.1	Serious	Very serious	Not serious	Serious	VERY LOW	Very serious	VERY LOW
HCL	PLGM	-7.0	-14.0	0.1	Serious	Not serious	Not serious	Not serious	MODERATE	Very serious	VERY LOW
AHCL	MDI	-20.3	-30.7	-10.0	Not serious	Serious	Not serious	Not serious	MODERATE	Serious	LOW
AHCL	CSII	-14.0	-20.4	-7.6	Not serious	Not serious	Serious	Not serious	MODERATE	Serious	LOW
AHCL	SAP	-9.8	-12.2	-7.4	Not serious	Serious	Not serious	Serious	LOW	Not serious	LOW
AHCL	PLGM	-10.9	-21.9	0.1	Not serious	Serious	Not serious	Not serious	MODERATE	Very serious	VERY LOW
AHCL	HCL	-6.9	-14.8	0.9	Not serious	Serious	Not serious	Not serious	MODERATE	Very serious	VERY LOW
FCL	MDI										
FCL	CSII										
FCL	SAP	-13.9	-20.2	-7.6	Serious	Not serious	Not serious	Not serious	MODERATE	Serious	LOW
FCL	PLGM										
FCL	HCL										
FCL	AHCL										

**Comparison**		**Indirect estimate TAR >180 mg/dl**				**GRADE**			
Intervention	Control	Point estimate	CI lower limit	CI upper limit	Start rating	Intransitivity	**Rating for NMA**	Imprecision	**Final rating**
HCL	MDI	-14.7	-22.1	-7.2	MODERATE	Serious	LOW	Not Serious	LOW
HCL	CSII	-8.5	-23.0	6.1	MODERATE	Serious	LOW	Serious	VERY LOW
HCL	SAP	-4.4	-11.3	2.5	LOW	Serious	VERY LOW	Serious	VERY LOW
HCL	PLGM	-5.5	-12.6	1.7	MODERATE	Serious	LOW	Serious	VERY LOW
AHCL	MDI	-17.9	-31.7	-4.2	MODERATE	Serious	LOW	Serious	VERY LOW
AHCL	CSII	-16.9	-26.4	-7.3	MODERATE	Serious	LOW	Not Serious	LOW
AHCL	SAP	-10.5	-17.6	-3.4	VERY LOW	Serious	VERY LOW	Serious	VERY LOW
AHCL	PLGM	-12.6	-22.9	-2.3	MODERATE	Serious	LOW	Serious	VERY LOW
AHCL	HCL	-5.0	-9.5	-0.5	VERY LOW	Serious	VERY LOW	Serious	VERY LOW
FCL	MDI								
FCL	CSII								
FCL	SAP								
FCL	PLGM								
FCL	HCL								
FCL	AHCL								

Comparison		Network estimate TAR >180 mg/dl			GRADE					
Intervention	Control	Point estimate	CrI lower limit	CrI upper limit	Starting evidence	**Starting rating**	Incoherence	Imprecision	**Final rating**	**Best estimate**
HCL	MDI	-4.7	-8.2	-1.3	Direct	MODERATE	Not serious	Serious	LOW	NMA
HCL	CSII	-14.2	-20.6	-7.8	Direct	MODERATE	Not serious	Not Serious	MODERATE	NMA
HCL	SAP	-10.6	-17.9	-3.2	Direct	VERY LOW	Not serious	Serious	VERY LOW	NMA
HCL	PLGM	-6.0	-11.6	-0.4	Direct	MODERATE	Not serious	Serious	LOW	NMA
AHCL	MDI	-10.1	-13.4	-6.9	Direct	MODERATE	Not serious	Not Serious	MODERATE	NMA
AHCL	CSII	-19.6	-25.1	-14.0	Direct	MODERATE	Not serious	Not Serious	MODERATE	NMA
AHCL	SAP	-15.9	-23.7	-8.1	Direct	LOW	Not serious	Not Serious	LOW	NMA
AHCL	PLGM	-11.4	-16.4	-6.5	Direct	MODERATE	Not serious	Not Serious	MODERATE	NMA
AHCL	HCL	-5.4	-9.3	-1.4	Direct	MODERATE	Not serious	Serious	LOW	NMA
FCL	MDI	-13.9	-27.5	-0.4	Direct	HIGH	Not serious	Serious	MODERATE	NMA
FCL	CSII	-23.3	-38.2	-8.4	Direct	HIGH	Not serious	Not Serious	HIGH	NMA
FCL	SAP	-19.7	-35.4	-4.1	Direct	MODERATE	Not serious	Serious	LOW	NMA
FCL	PLGM	-15.2	-29.8	-0.6	Direct	HIGH	Not serious	Serious	MODERATE	NMA
FCL	HCL	-9.2	-23.2	4.8	Direct	HIGH	Not serious	Serious	MODERATE	NMA
FCL	AHCL	-3.8	-17.7	10.2	Direct	HIGH	Not serious	Serious	MODERATE	NMA

10.4 CoE assessment TAR >250 mg/dl network meta-analysis

Comparison		Direct estimate TAR >250 mg/dl			GRADE						
Intervention	Control	Point estimate	CI lower limit	CI upper limit	Risk of bias	Inconsistency	Indirectness	Publication bias	**Rating for NMA**	Imprecision	**Final rating**
HCL	MDI	-3.4	-4.6	-2.2	Not serious	Not serious	Serious	Not serious	MODERATE	Serious	LOW
HCL	CSII	-4.8	-11.8	2.1	Serious	Not serious	Not serious	Not serious	MODERATE	Very serious	VERY LOW
HCL	SAP	-3.5	-6.7	-0.3	Serious	Very serious	Not serious	Not serious	VERY LOW	Very serious	VERY LOW
HCL	PLGM	-0.4	-2.3	1.5	Not serious	Not serious	Serious	Not serious	MODERATE	Very serious	VERY LOW
AHCL	MDI	-18.6	-30.8	-6.4	Not serious	Serious	Not serious	Not serious	MODERATE	Serious	LOW
AHCL	CSII	-6.0	-9.5	-2.5	Not serious	Not serious	Serious	Not serious	MODERATE	Serious	LOW
AHCL	SAP	-6.5	-8.4	-4.7	Not serious	Serious	Not serious	Serious	LOW	Not serious	LOW
AHCL	PLGM	-8.9	-20.6	2.8	Not serious	Serious	Not serious	Not serious	MODERATE	Very serious	VERY LOW
AHCL	HCL	-3.4	-8.1	1.4	Not serious	Serious	Not serious	Not serious	MODERATE	Very serious	VERY LOW
FCL	MDI										
FCL	CSII										
FCL	SAP	-10.7	-18.8	-2.6	Serious	Not serious	Not serious	Not serious	MODERATE	Serious	LOW
FCL	PLGM										
FCL	HCL										
FCL	AHCL										

Comparison		Indirect estimate TAR >250 mg/dl				GRADE			
Intervention	Control	Point estimate	CI lower limit	CI upper limit	Start rating	Intransitivity	**Rating for NMA**	Imprecision	**Final rating**
HCL	MDI	-13.8	-21.7	-5.9	MODERATE	Serious	LOW	Not Serious	LOW
HCL	CSII	-1.0	-13.1	11.2	MODERATE	Serious	LOW	Serious	VERY LOW
HCL	SAP	-1.4	-7.9	5.2	LOW	Serious	VERY LOW	Serious	VERY LOW
HCL	PLGM	-4.7	-12.3	2.8	MODERATE	Serious	LOW	Serious	VERY LOW
AHCL	MDI	-7.2	-18.3	3.9	MODERATE	Serious	LOW	Serious	VERY LOW
AHCL	CSII	-10.6	-20.8	-0.5	MODERATE	Serious	LOW	Serious	VERY LOW
AHCL	SAP	-9.6	-16.4	-2.8	VERY LOW	Serious	VERY LOW	Not Serious	VERY LOW
AHCL	PLGM	-4.7	-16.3	7.0	MODERATE	Serious	LOW	Serious	VERY LOW
AHCL	HCL	-5.2	-9.6	-0.7	VERY LOW	Serious	VERY LOW	Serious	VERY LOW
FCL	MDI								
FCL	CSII								
FCL	SAP								
FCL	PLGM								
FCL	HCL								
FCL	AHCL								

Comparison		Network estimate TAR >250 mg/dl			GRADE					
Intervention	Control	Point estimate	CrI lower limit	CrI upper limit	Starting evidence	**Starting rating**	Incoherence	Imprecision	**Final rating**	**Best estimate**
HCL	MDI	-3.1	-6.5	0.3	Direct	MODERATE	Not serious	Serious	LOW	DIRECT
HCL	CSII	-10.1	-16.5	-3.6	Direct	MODERATE	Not serious	Not Serious	MODERATE	NMA
HCL	SAP	-3.9	-11.1	3.4	Direct	VERY LOW	Not serious	Serious	VERY LOW	NMA
HCL	PLGM	-3.3	-9.4	2.8	Direct	MODERATE	Not serious	Serious	LOW	NMA
AHCL	MDI	-7.8	-10.9	-4.6	Direct	MODERATE	Not serious	Not Serious	MODERATE	NMA
AHCL	CSII	-14.8	-20.8	-8.8	Direct	MODERATE	Not serious	Not Serious	MODERATE	NMA
AHCL	SAP	-8.6	-16.0	-1.1	Direct	LOW	Not serious	Not Serious	LOW	NMA
AHCL	PLGM	-8.0	-13.5	-2.5	Direct	MODERATE	Not serious	Not Serious	MODERATE	NMA
AHCL	HCL	-4.7	-8.5	-0.9	Direct	MODERATE	Not serious	Serious	LOW	NMA
FCL	MDI	-10.7	-24.2	2.8	Direct	HIGH	Not serious	Serious	MODERATE	NMA
FCL	CSII	-17.7	-32.7	-2.8	Direct	HIGH	Not serious	Not Serious	HIGH	NMA
FCL	SAP	-11.5	-27.0	4.0	Direct	MODERATE	Not serious	Serious	LOW	NMA
FCL	PLGM	-10.9	-25.7	3.9	Direct	HIGH	Not serious	Serious	MODERATE	NMA
FCL	HCL	-7.6	-21.5	6.3	Direct	HIGH	Not serious	Not Serious	HIGH	NMA
FCL	AHCL	-2.9	-16.8	10.9	Direct	HIGH	Not serious	Serious	MODERATE	NMA

10.5 CoE assessment TBR <70 mg/dl network meta-analysis

**Comparison**		**Direct estimate TBR <70 mg/dl**			**GRADE**						
Intervention	Control	Point estimate	CI lower limit	CI upper limit	Risk of bias	Inconsistency	Indirectness	Publication bias	**Rating for NMA**	Imprecision	**Final rating**
HCL	MDI	-1.7	-2.6	-0.8	Not serious	Not serious	Serious	Not serious	MODERATE	Very serious	VERY LOW
HCL	CSII	-3.6	-4.8	-2.3	Serious	Not serious	Not serious	Not serious	MODERATE	Serious	LOW
HCL	SAP	-1.3	-1.9	-0.6	Not serious	Very serious	Not serious	Serious	VERY LOW	Very serious	VERY LOW
HCL	PLGM	-1.3	-4.6	1.9	Serious	Very serious	Not serious	Not serious	VERY LOW	Very serious	VERY LOW
AHCL	MDI	-0.7	-2.6	1.2	Not serious	Very serious	Not serious	Not serious	LOW	Very serious	VERY LOW
AHCL	CSII	0.4	-0.1	0.9	Not serious	Not serious	Serious	Not serious	MODERATE	Very serious	VERY LOW
AHCL	SAP	-1.1	-1.7	-0.5	Not serious	Serious	Not serious	Not serious	MODERATE	Serious	LOW
AHCL	PLGM	-1.1	-2.2	0.1	Not serious	Serious	Not serious	Not serious	MODERATE	Very serious	VERY LOW
AHCL	HCL	0.1	-0.1	0.4	Not serious	Not serious	Not serious	Not serious	HIGH	Very serious	LOW
FCL	MDI										
FCL	CSII										
FCL	SAP	0.6	0.0	1.2	Serious	Not serious	Not serious	Not serious	MODERATE	Very serious	VERY LOW
FCL	PLGM										
FCL	HCL										
FCL	AHCL										

**Comparison**		**Indirect estimate TBR <70 mg/dl**				**GRADE**			
Intervention	Control	Point estimate	CI lower limit	CI upper limit	Start rating	Intransitivity	**Rating for NMA**	Imprecision	**Final rating**
HCL	MDI	-1.1	-3.1	0.9	LOW	Serious	VERY LOW	Serious	VERY LOW
HCL	CSII	0.1	-2.5	2.7	MODERATE	Serious	LOW	Serious	VERY LOW
HCL	SAP	-2.0	-3.5	-0.5	MODERATE	Serious	LOW	Serious	VERY LOW
HCL	PLGM	-1.7	-3.3	-0.2	MODERATE	Serious	LOW	Serious	VERY LOW
AHCL	MDI	-1.2	-4.1	1.8	MODERATE	Serious	LOW	Serious	VERY LOW
AHCL	CSII	-3.4	-5.2	-1.6	MODERATE	Serious	LOW	Not Serious	LOW
AHCL	SAP	-0.5	-2.0	1.0	VERY LOW	Serious	VERY LOW	Serious	VERY LOW
AHCL	PLGM	-0.6	-2.9	1.7	VERY LOW	Serious	VERY LOW	Serious	VERY LOW
AHCL	HCL	0.7	-0.3	1.6	VERY LOW	Serious	VERY LOW	Serious	VERY LOW
FCL	MDI								
FCL	CSII								
FCL	SAP								
FCL	PLGM								
FCL	HCL								
FCL	AHCL								

**Comparison**		**Network estimate TBR <70 mg/dl**			**GRADE**					
Intervention	Control	Point estimate	CrI lower limit	CrI upper limit	Starting evidence	**Starting rating**	Incoherence	Imprecision	**Final rating**	**Best estimate**
HCL	MDI	-1.5	-2.2	-0.8	Direct	MODERATE	Not serious	Serious	LOW	NMA
HCL	CSII	-1.3	-2.8	0.3	Direct	MODERATE	Serious	Serious	VERY LOW	DIRECT
HCL	SAP	-2.7	-4.2	-1.2	Indirect	LOW	Not serious	Not Serious	LOW	NMA
HCL	PLGM	-1.5	-2.8	-0.3	Indirect	LOW	Not serious	Serious	VERY LOW	NMA
AHCL	MDI	-1.0	-1.7	-0.2	Direct	LOW	Not serious	Serious	VERY LOW	NMA
AHCL	CSII	-0.7	-2.2	0.7	Direct	MODERATE	Serious	Serious	VERY LOW	INDIRECT
AHCL	SAP	-2.1	-3.7	-0.5	Direct	MODERATE	Not serious	Serious	LOW	NMA
AHCL	PLGM	-1.0	-2.1	0.1	Direct	MODERATE	Not serious	Serious	LOW	NMA
AHCL	HCL	0.6	-0.3	1.4	Direct	HIGH	Not serious	Serious	MODERATE	NMA
FCL	MDI	0.6	-2.1	3.3	Direct	HIGH	Not serious	Serious	MODERATE	NMA
FCL	CSII	0.8	-2.3	3.9	Direct	HIGH	Not serious	Serious	MODERATE	NMA
FCL	SAP	-0.6	-3.7	2.6	Direct	MODERATE	Not serious	Serious	LOW	NMA
FCL	PLGM	0.6	-2.4	3.5	Direct	HIGH	Not serious	Serious	MODERATE	NMA
FCL	HCL	2.1	-0.7	4.9	Direct	HIGH	Not serious	Serious	MODERATE	NMA
FCL	AHCL	1.6	-1.2	4.3	Direct	HIGH	Not serious	Serious	MODERATE	NMA

10.6 CoE assessment TBR <54 mg/dl network meta-analysis

**Comparison**		**Direct estimate TBR <54 mg/dl**			**GRADE**						
Intervention	Control	Point estimate	CI lower limit	CI upper limit	Risk of bias	Inconsistency	Indirectness	Publication bias	**Rating for NMA**	Imprecision	**Final rating**
HCL	MDI	-0.1	-0.8	0.6	Not serious	Not serious	Serious	Not serious	MODERATE	Very serious	VERY LOW
HCL	CSII	-1.2	-2.2	-0.2	Serious	Not serious	Not serious	Not serious	MODERATE	Very serious	VERY LOW
HCL	SAP	-0.3	-0.6	-0.1	Not serious	Serious	Not serious	Not serious	MODERATE	Very serious	VERY LOW
HCL	PLGM	-0.4	-1.7	0.9	Serious	Very serious	Not serious	Not serious	VERY LOW	Very serious	VERY LOW
AHCL	MDI	-0.4	-0.9	0.0	Not serious	Very serious	Not serious	Not serious	LOW	Very serious	VERY LOW
AHCL	CSII	0.1	0.0	0.2	Not serious	Not serious	Serious	Not serious	MODERATE	Very serious	VERY LOW
AHCL	SAP	-0.2	-0.3	0.0	Not serious	Very serious	Not serious	Not serious	LOW	Very serious	VERY LOW
AHCL	PLGM	-0.3	-0.6	0.0	Not serious	Serious	Not serious	Not serious	MODERATE	Very serious	VERY LOW
AHCL	HCL	-0.1	-0.1	0.0	Not serious	Not serious	Not serious	Not serious	HIGH	Very serious	LOW
FCL	MDI										
FCL	CSII										
FCL	SAP	0.0	0.0	0.1	Serious	Not serious	Not serious	Not serious	MODERATE	Very serious	VERY LOW
FCL	PLGM										
FCL	HCL										
FCL	AHCL										

**Comparison**		**Indirect estimate TBR <54 mg/dl**				**GRADE**			
Intervention	Control	Point estimate	CI lower limit	CI upper limit	Start rating	Intransitivity	**Rating for NMA**	Imprecision	**Final rating**
HCL	MDI	-0.6	-1.1	0.0	LOW	Serious	VERY LOW	Serious	VERY LOW
HCL	CSII	0.0	-0.7	0.7	MODERATE	Serious	LOW	Serious	VERY LOW
HCL	SAP	-0.2	-0.7	0.2	LOW	Serious	VERY LOW	Serious	VERY LOW
HCL	PLGM	-0.5	-1.0	-0.1	MODERATE	Serious	LOW	Serious	VERY LOW
AHCL	MDI	0.1	-0.9	1.2	MODERATE	Serious	LOW	Serious	VERY LOW
AHCL	CSII	-1.2	-1.9	-0.5	MODERATE	Serious	LOW	Serious	VERY LOW
AHCL	SAP	-0.3	-0.8	0.2	MODERATE	Serious	LOW	Serious	VERY LOW
AHCL	PLGM	-0.1	-0.8	0.6	VERY LOW	Serious	VERY LOW	Serious	VERY LOW
AHCL	HCL	0.2	-0.1	0.5	LOW	Serious	VERY LOW	Serious	VERY LOW
FCL	MDI								
FCL	CSII								
FCL	SAP								
FCL	PLGM								
FCL	HCL								
FCL	AHCL								

**Comparison**		**Network estimate TBR <54 mg/dl**			**GRADE**					
Intervention	Control	Point estimate	CrI lower limit	CrI upper limit	Starting evidence	**Starting rating**	Incoherence	Imprecision	**Final rating**	**Best estimate**
HCL	MDI	-0.3	-0.6	-0.1	Direct	MODERATE	Not serious	Serious	LOW	NMA
HCL	CSII	-0.5	-0.9	0.0	Direct	MODERATE	Serious	Serious	VERY LOW	NMA
HCL	SAP	-0.6	-1.2	-0.1	Direct	MODERATE	Not serious	Serious	LOW	NMA
HCL	PLGM	-0.4	-0.8	-0.1	Direct	VERY LOW	Not serious	Serious	VERY LOW	NMA
AHCL	MDI	-0.2	-0.4	0.0	Direct	LOW	Not serious	Serious	VERY LOW	NMA
AHCL	CSII	-0.3	-0.7	0.1	Direct	MODERATE	Serious	Serious	VERY LOW	NMA
AHCL	SAP	-0.5	-1.0	0.1	Direct	LOW	Not serious	Serious	VERY LOW	NMA
AHCL	PLGM	-0.3	-0.6	0.1	Direct	MODERATE	Not serious	Serious	LOW	NMA
AHCL	HCL	0.2	-0.1	0.4	Direct	HIGH	Not serious	Serious	MODERATE	NMA
FCL	MDI	0.0	-0.7	0.8	Direct	HIGH	Not serious	Serious	MODERATE	NMA
FCL	CSII	-0.1	-1.0	0.8	Direct	HIGH	Not serious	Serious	MODERATE	NMA
FCL	SAP	-0.3	-1.2	0.7	Direct	MODERATE	Not serious	Serious	LOW	NMA
FCL	PLGM	-0.1	-0.9	0.8	Direct	HIGH	Not serious	Serious	MODERATE	NMA
FCL	HCL	0.4	-0.4	1.1	Direct	HIGH	Not serious	Serious	MODERATE	NMA
FCL	AHCL	0.2	-0.6	1.0	Direct	HIGH	Not serious	Serious	MODERATE	NMA

10.7 CoE assessment HbA1c network meta-analysis

**Comparison**		**Direct estimate HbA1c**			**GRADE**						
Intervention	Control	Point estimate	CI lower limit	CI upper limit	Risk of bias	Inconsistency	Indirectness	Publication bias	**Rating for NMA**	Imprecision	**Final rating**
HCL	MDI	-0.4	-0.7	-0.1	Not serious	Not serious	Serious	Not serious	MODERATE	Very serious	VERY LOW
HCL	CSII	-0.4	-0.6	-0.2	Serious	Not serious	Not serious	Not serious	MODERATE	Very serious	VERY LOW
HCL	SAP	-0.1	0.4	0.1	Not serious	Very serious	Not serious	Not serious	LOW	Very serious	VERY LOW
HCL	PLGM	-0.1	-0.4	0.2	Not serious	Not serious	Serious	Not serious	MODERATE	Very serious	VERY LOW
AHCL	MDI	-1.2	-1.8	-0.6	Not serious	Serious	Not serious	Not serious	MODERATE	Serious	LOW
AHCL	CSII	-0.6	-1.0	-0.2	Not serious	Not serious	Serious	Not serious	MODERATE	Very serious	VERY LOW
AHCL	SAP	-0.5	-0.7	-0.3	Not serious	Serious	Not serious	Not serious	MODERATE	Serious	LOW
AHCL	PLGM	-0.1	-0.4	0.1	Not serious	Serious	Not serious	Not serious	MODERATE	Very serious	VERY LOW
AHCL	HCL	-0.3	-0.5	-0.1	Not serious	Not serious	Not serious	Not serious	HIGH	Very serious	LOW
FCL	MDI										
FCL	CSII										
FCL	SAP										
FCL	PLGM										
FCL	HCL										
FCL	AHCL										

**Comparison**		**Indirect estimate HbA1c**			**GRADE**				
Intervention	Control	Point estimate	CI lower limit	CI upper limit	Start rating	Intransitivity	**Rating for NMA**	Imprecision	**Final rating**
HCL	MDI	-0.8	-1.2	-0.4	MODERATE	Serious	LOW	Serious	VERY LOW
HCL	CSII	-0.3	-1.1	0.5	MODERATE	Serious	LOW	Serious	VERY LOW
HCL	SAP	-0.2	-0.6	0.3	MODERATE	Serious	LOW	Serious	VERY LOW
HCL	PLGM	0.2	-0.3	0.7	MODERATE	Serious	LOW	Serious	VERY LOW
AHCL	MDI	-0.7	-1.4	0.0	MODERATE	Serious	LOW	Serious	VERY LOW
AHCL	CSII	-0.7	-1.5	0.0	MODERATE	Serious	LOW	Serious	VERY LOW
AHCL	SAP	-0.5	-0.9	0.0	LOW	Serious	VERY LOW	Serious	VERY LOW
AHCL	PLGM	-0.5	-1.2	0.3	MODERATE	Serious	LOW	Serious	VERY LOW
AHCL	HCL	-0.4	-0.6	-0.1	MODERATE	Serious	LOW	Serious	VERY LOW
FCL	MDI								
FCL	CSII								
FCL	SAP								
FCL	PLGM								
FCL	HCL								
FCL	AHCL								

Comparison		Network estimate HbA1c			GRADE					
Intervention	Control	Point estimate	CrI lower limit	CrI upper limit	Starting evidence	**Starting rating**	Incoherence	Imprecision	**Final rating**	**Best estimate**
HCL	MDI	-0.1	-0.3	0.1	Direct	MODERATE	Not serious	Serious	LOW	NMA
HCL	CSII	-0.7	-1.1	-0.3	Direct	MODERATE	Not serious	Serious	LOW	NMA
HCL	SAP	-0.3	-0.9	0.2	Direct	LOW	Not serious	Serious	VERY LOW	NMA
HCL	PLGM	0.1	-0.3	0.5	Direct	MODERATE	Not serious	Serious	LOW	NMA
AHCL	MDI	-0.5	-0.7	-0.3	Direct	MODERATE	Not serious	Serious	LOW	NMA
AHCL	CSII	-1.0	-1.4	-0.7	Direct	MODERATE	Not serious	Not Serious	MODERATE	NMA
AHCL	SAP	-0.7	-1.2	-0.2	Direct	MODERATE	Not serious	Serious	LOW	NMA
AHCL	PLGM	-0.2	-0.6	0.2	Direct	MODERATE	Not serious	Serious	LOW	NMA
AHCL	HCL	-0.3	-0.6	-0.1	Direct	HIGH	Not serious	Serious	MODERATE	NMA
FCL	MDI									
FCL	CSII									
FCL	SAP									
FCL	PLGM									
FCL	HCL									
FCL	AHCL									

11 References

1. Sherr JL, Heinemann L, Fleming GA, et al. Automated insulin delivery: benefits, challenges, and recommendations. A Consensus Report of the Joint Diabetes Technology Working Group of the European Association for the Study of Diabetes and the American Diabetes Association. *Diabetologia* 2023; **66**(1): 3-22.
2. Lynch J, Kanapka LG, Russell SJ, et al. The Insulin-Only Bionic Pancreas Pivotal Trial Extension Study: A Multi-Center Single-Arm Evaluation of the Insulin-Only Configuration of the Bionic Pancreas in Adults and Youth with Type 1 Diabetes. *Diabetes Technol Ther* 2022; **24**(10): 726-36.
3. Zeng B, Jia H, Gao L, Yang Q, Yu K, Sun F. Dual-hormone artificial pancreas for glucose control in type 1 diabetes: A meta-analysis. *Diabetes Obes Metab* 2022; **24**(10): 1967-75.
4. Deutsche Diabetes Gesellschaft (DDG). S3-Leitlinie Therapie des Typ-1-Diabetes. 2023.
5. Lameijer A, Fokkert MJ, Edens MA, Slingerland RJ, Bilo HJG, van Dijk PR. Determinants of HbA1c reduction with FreeStyle Libre flash glucose monitoring (FLARE-NL 5). *J Clin Transl Endocrinol* 2020; **22**: 100237.
6. Lenters-Westra E, Schindhelm R, Bilo H, Groenier K, Slingerland R. Differences in interpretation of haemoglobin A1c values among diabetes care professionals. *The Netherlands journal of medicine* 2014; **72**(9): 462-6.
7. Izcovich A, Chu DK, Mustafa RA, Guyatt G, Brignardello-Petersen R. A guide and pragmatic considerations for applying GRADE to network meta-analysis. *BMJ* 2023; **381**: e074495.
8. Abraham MB, de Bock M, Smith GJ, et al. Effect of a Hybrid Closed-Loop System on Glycemic and Psychosocial Outcomes in Children and Adolescents With Type 1 Diabetes: A Randomized Clinical Trial. *JAMA Pediatr* 2021; **175**(12): 1227-35.
9. Abraham MB, Smith GJ, Dart J, et al. Glycemic and Psychosocial Outcomes of Advanced Hybrid Closed-Loop Therapy in Youth With High HbA1c: A Randomized Clinical Trial. *Diabetes Care* 2025; **48**(1): 67-75.
10. Anderson SM, Buckingham BA, Breton MD, et al. Hybrid Closed-Loop Control Is Safe and Effective for People with Type 1 Diabetes Who Are at Moderate to High Risk for Hypoglycemia. *Diabetes Technol Ther* 2019; **21**(6): 356-63.
11. Bally L, Thabit H, Kojzar H, et al. Day-and-night glycaemic control with closed-loop insulin delivery versus conventional insulin pump therapy in free-living adults with well controlled type 1 diabetes: an open-label, randomised, crossover study. *The Lancet Diabetes & Endocrinology* 2017; **5**(4): 261-70.
12. Benhalima K, Beunen K, Van Wilder N, et al. Comparing advanced hybrid closed loop therapy and standard insulin therapy in pregnant women with type 1 diabetes (CRISTAL): a parallel-group, open-label, randomised controlled trial. *The Lancet Diabetes & Endocrinology* 2024; **12**(6): 390-403.
13. Benhamou PY, Franc S, Reznik Y, et al. Closed-loop insulin delivery in adults with type 1 diabetes in real-life conditions: a 12-week multicentre, open-label randomised controlled crossover trial. *Lancet Digit Health* 2019; **1**(1): e17-e25.
14. Benhamou PY, Lablanche S, Vambergue A, Doron M, Franc S, Charpentier G. Patients with highly unstable type 1 diabetes eligible for islet transplantation can be managed with a closed-loop insulin delivery system: A series of N-of-1 randomized controlled trials. *Diabetes Obes Metab* 2021; **23**(1): 186-94.
15. Bergenstal RM, Nimri R, Beck RW, et al. A comparison of two hybrid closed-loop systems in adolescents and young adults with type 1 diabetes (FLAIR): a multicentre, randomised, crossover trial. *Lancet* 2021; **397**(10270): 208-19.
16. Boucsein A, Zhou Y, Michaels V, et al. Automated Insulin Delivery for Young People with Type 1 Diabetes and Elevated A1c. *NEJM Evid* 2024; **3**(10): EVIDoa2400185.
17. Boughton CK, Allen JM, Ware J, et al. Closed-Loop Therapy and Preservation of C-Peptide Secretion in Type 1 Diabetes. *N Engl J Med* 2022; **387**(10): 882-93.

18. Boughton CK, Hartnell S, Thabit H, et al. Hybrid closed-loop glucose control compared with sensor augmented pump therapy in older adults with type 1 diabetes: an open-label multicentre, multinational, randomised, crossover study. *Lancet Healthy Longev* 2022; **3**(3): e135-e42.
19. Boughton CK, Hartnell S, Lakshman R, et al. Fully Closed-Loop Glucose Control Compared With Insulin Pump Therapy With Continuous Glucose Monitoring in Adults With Type 1 Diabetes and Suboptimal Glycemic Control: A Single-Center, Randomized, Crossover Study. *Diabetes Care* 2023; **46**(11): 1916-22.
20. Breton MD, Kanapka LG, Beck RW, et al. A Randomized Trial of Closed-Loop Control in Children with Type 1 Diabetes. *N Engl J Med* 2020; **383**(9): 836-45.
21. Brown SA, Kovatchev BP, Raghinaru D, et al. Six-Month Randomized, Multicenter Trial of Closed-Loop Control in Type 1 Diabetes. *N Engl J Med* 2019; **381**(18): 1707-17.
22. Brown SA, Beck RW, Raghinaru D, et al. Glycemic Outcomes of Use of CLC Versus PLGS in Type 1 Diabetes: A Randomized Controlled Trial. *Diabetes Care* 2020; **43**(8): 1822-8.
23. Burckhardt MA, Abraham MB, Dart J, et al. Impact of Hybrid Closed Loop Therapy on Hypoglycemia Awareness in Individuals with Type 1 Diabetes and Impaired Hypoglycemia Awareness. *Diabetes Technol Ther* 2021; **23**(7): 482-90.
24. Burnside MJ, Lewis DM, Crocket HR, et al. Open-Source Automated Insulin Delivery in Type 1 Diabetes. *N Engl J Med* 2022; **387**(10): 869-81.
25. Choudhary P, Kolassa R, Keuthage W, et al. Advanced hybrid closed loop therapy versus conventional treatment in adults with type 1 diabetes (ADAPT): a randomised controlled study. *The Lancet Diabetes & Endocrinology* 2022; **10**(10): 720-31.
26. Christensen MB, Ranjan AG, Rytter K, McCarthy OM, Schmidt S, Norgaard K. Automated Insulin Delivery in Adults With Type 1 Diabetes and Suboptimal HbA(1c) During Prior Use of Insulin Pump and Continuous Glucose Monitoring: A Randomized Controlled Trial. *J Diabetes Sci Technol* 2024: 19322968241242399.
27. Collyns OJ, Meier RA, Betts ZL, et al. Improved Glycemic Outcomes With Medtronic MiniMed Advanced Hybrid Closed-Loop Delivery: Results From a Randomized Crossover Trial Comparing Automated Insulin Delivery With Predictive Low Glucose Suspend in People With Type 1 Diabetes. *Diabetes Care* 2021; **44**(4): 969-75.
28. Donovan LE, Feig DS, Lemieux P, et al. A Randomized Trial of Closed-Loop Insulin Delivery Postpartum in Type 1 Diabetes. *Diabetes Care* 2023; **46**(12): 2258-66.
29. Garg SK, Grunberger G, Weinstock R, et al. Improved Glycemia with Hybrid Closed-Loop Versus Continuous Subcutaneous Insulin Infusion Therapy: Results from a Randomized Controlled Trial. *Diabetes Technol Ther* 2023; **25**(1): 1-12.
30. Kariyawasam D, Morin C, Casteels K, et al. Hybrid closed-loop insulin delivery versus sensor-augmented pump therapy in children aged 6-12 years: a randomised, controlled, cross-over, non-inferiority trial. *Lancet Digit Health* 2022; **4**(3): e158-e68.
31. Kim JY, Jin SM, Kang ES, et al. Comparison between a tubeless, on-body automated insulin delivery system and a tubeless, on-body sensor-augmented pump in type 1 diabetes: a multicentre randomised controlled trial. *Diabetologia* 2024; **67**(7): 1235-44.
32. Kovatchev B, Anderson SM, Raghinaru D, et al. Randomized Controlled Trial of Mobile Closed-Loop Control. *Diabetes Care* 2020; **43**(3): 607-15.
33. Kudva YC, Henderson RJ, Kanapka LG, et al. Automated Insulin Delivery in Older Adults with Type 1 Diabetes. *NEJM Evid* 2025; **4**(1): EVIDoa2400200.
34. Lee TTM, Collett C, Bergford S, et al. Automated Insulin Delivery in Women with Pregnancy Complicated by Type 1 Diabetes. *N Engl J Med* 2023; **389**(17): 1566-78.
35. Matejko B, Juza A, Kiec-Wilk B, et al. Transitioning of People With Type 1 Diabetes From Multiple Daily Injections and Self-Monitoring of Blood Glucose Directly to MiniMed 780G Advanced Hybrid Closed-Loop System: A Two-Center, Randomized, Controlled Study. *Diabetes Care* 2022; **45**(11): 2628-35.

36. McAuley SA, Lee MH, Paldus B, et al. Six Months of Hybrid Closed-Loop Versus Manual Insulin Delivery With Fingerprick Blood Glucose Monitoring in Adults With Type 1 Diabetes: A Randomized, Controlled Trial. *Diabetes Care* 2020; **43**(12): 3024-33.
37. McAuley SA, Trawley S, Vogrin S, et al. Closed-Loop Insulin Delivery Versus Sensor-Augmented Pump Therapy in Older Adults With Type 1 Diabetes (ORACL): A Randomized, Crossover Trial. *Diabetes Care* 2022; **45**(2): 381-90.
38. McVean J, Forlenza GP, Beck RW, et al. Effect of Tight Glycemic Control on Pancreatic Beta Cell Function in Newly Diagnosed Pediatric Type 1 Diabetes: A Randomized Clinical Trial. *Jama* 2023; **329**(12): 980-9.
39. Nanayakkara N, Sharifi A, Burren D, Elghattis Y, Jayarathna DK, Cohen N. Hybrid Closed Loop Using a Do-It-Yourself Artificial Pancreas System in Adults With Type 1 Diabetes. *J Diabetes Sci Technol* 2024; **18**(4): 889-96.
40. Pinsker JE, Dassau E, Deshpande S, et al. Outpatient Randomized Crossover Comparison of Zone Model Predictive Control Automated Insulin Delivery with Weekly Data Driven Adaptation Versus Sensor-Augmented Pump: Results from the International Diabetes Closed-Loop Trial 4. *Diabetes Technol Ther* 2022; **24**(9): 635-42.
41. Polsky S, Buschur E, Dungan K, et al. Randomized Trial of Assisted Hybrid Closed-Loop Therapy Versus Sensor-Augmented Pump Therapy in Pregnancy. *Diabetes Technol Ther* 2024; **26**(8): 547-55.
42. Renard E, Joubert M, Villard O, et al. Safety and Efficacy of Sustained Automated Insulin Delivery Compared With Sensor and Pump Therapy in Adults With Type 1 Diabetes at High Risk for Hypoglycemia: A Randomized Controlled Trial. *Diabetes Care* 2023; **46**(12): 2180-7.
43. Renard E, Weinstock RS, Aleppo G, et al. Efficacy and Safety of a Tubeless AID System Compared With Pump Therapy With CGM in the Treatment of Type 1 Diabetes in Adults With Suboptimal Glycemia: A Randomized, Parallel-Group Clinical Trial. *Diabetes Care* 2024; **47**(12): 2248-57.
44. Russell SJ, Beck RW, Damiano ER, et al. Multicenter, Randomized Trial of a Bionic Pancreas in Type 1 Diabetes. *N Engl J Med* 2022; **387**(13): 1161-72.
45. Stewart ZA, Wilinska ME, Hartnell S, et al. Day-and-Night Closed-Loop Insulin Delivery in a Broad Population of Pregnant Women With Type 1 Diabetes: A Randomized Controlled Crossover Trial. *Diabetes Care* 2018; **41**(7): 1391-9.
46. Tauschmann M, Allen JM, Wilinska ME, et al. Home Use of Day-and-Night Hybrid Closed-Loop Insulin Delivery in Suboptimally Controlled Adolescents With Type 1 Diabetes: A 3-Week, Free-Living, Randomized Crossover Trial. *Diabetes Care* 2016; **39**(11): 2019-25.
47. Tauschmann M, Thabit H, Bally L, et al. Closed-loop insulin delivery in suboptimally controlled type 1 diabetes: a multicentre, 12-week randomised trial. *Lancet* 2018; **392**(10155): 1321-9.
48. Thabit H, Tauschmann M, Allen JM, et al. Home Use of an Artificial Beta Cell in Type 1 Diabetes. *N Engl J Med* 2015; **373**(22): 2129-40.
49. von dem Berge T, Remus K, Biester S, et al. In-home use of a hybrid closed loop achieves time-in-range targets in preschoolers and school children: Results from a randomized, controlled, crossover trial. *Diabetes Obes Metab* 2022; **24**(7): 1319-27.
50. Wadwa RP, Reed ZW, Buckingham BA, et al. Trial of Hybrid Closed-Loop Control in Young Children with Type 1 Diabetes. *N Engl J Med* 2023; **388**(11): 991-1001.
51. Ware J, Boughton CK, Allen JM, et al. Cambridge hybrid closed-loop algorithm in children and adolescents with type 1 diabetes: a multicentre 6-month randomised controlled trial. *Lancet Digit Health* 2022; **4**(4): e245-e55.
52. Ware J, Allen JM, Boughton CK, et al. Randomized Trial of Closed-Loop Control in Very Young Children with Type 1 Diabetes. *N Engl J Med* 2022; **386**(3): 209-19.
53. Brignardello-Petersen R, Bonner A, Alexander PE, et al. Advances in the GRADE approach to rate the certainty in estimates from a network meta-analysis. *Journal of clinical epidemiology* 2018; **93**: 36-44.
